# Supplementary material for: A negative genetic interaction map in isogenic cancer cell lines reveals cancer cell vulnerabilities
Source: Mol Syst Biol. 2013 Oct 8;9:696. doi: 10.1038/msb.2013.54 (PMC3817404; doi:10.1038/msb.2013.54)
Supplement: Supplementary Information — Supplementary Table Legends S1–12, Supplementary Figures S1–14 [file msb201354-s1.pdf]

SUPPLEMENTAL INFORMATION FOR:

A Negative Genetic Interaction Map in Isogenic Cancer Cell Lines Reveals Cancer Cell Vulnerabilities

Franco J. Vizeacoumar[1,2,Φ], Roland Arnold[1Φ], Frederick S. Vizeacoumar[3], Megha Chandrashekhar[1], Alla Buzina[1], Jordan T.F. Young[3, 4], Julian H.M. Kwan[1, 4], Azin Sayad[1], Patricia Mero[1], Steffen Lawo[3, 4], Hiromasa Tanaka[1], Kevin R. Brown[1], Anastasia Baryshnikova[1,4], Anthony B. Mak[1], Yaroslav Fedyshyn[1], Yadong Wang[9], Glauber C. Brito[1], Dahlia Kasimer[1], Taras Makhnevych[1], Troy Ketela[1], Alessandro Datti[3], Mohan Babu[10], Andrew Emili[1,4], Laurence Pelletier[3,4], Jeff Wrana[3,4], Zev Wainberg[11], Philip M. Kim[1,4,6], Robert Rottapel[5], Catherine A. O'Brien[7,8,9], Brenda Andrews[1,4], Charles Boone[1,4] and *Jason Moffat[1,4]

[1]Donnelly Centre and Banting and Best Department of Medical Research, University of Toronto, 160 College St., Toronto, Ontario, Canada M5S 3E1
[2]Sakatchewan Cancer Agency, Department of Biochemistry, University of Saskatchewan, Saskatoon, Saskatchewan S7N 5E5, Canada
[3]Samuel Lunenfeld Research Institute, Mount Sinai Hospital, 600 University Avenue, Toronto, Ontario, Canada M5G1X5
[4]Department of Molecular Genetics, University of Toronto, 1 King's College Circle, Toronto, Ontario, Canada, M5S 1A8
[5]Department of Medical Biophysics, University of Toronto,
[6]Department of Computer Science, University of Toronto
[7]Department of Laboratory Medicine and Pathology and Department of Surgery, University of Toronto, Toronto, Ontario, Canada, M5L1F4
[8]Department of Surgery, University Health Network, Toronto, Ontario, Canada, M5G1L7
[9]Campbell Family Institute, Ontario Cancer Institute, Princess Margaret Hospital, University Health Network, Toronto, Ontario, Canada, M5G1L7
[10]Department of Biochemistry, Research and Innovation Centre, University of Regina, Regina, Saskatchewan, Canada
[11]Jonnson Comprehensive Cancer Center, Geffen School of Medicine, University of California at Los Angeles, Los Angeles, California, USA

[Φ]These authors contributed equally to this work.
*Correspondence:
Jason Moffat: j.moffat@utoronto.ca; Phone – 416-978-0336; FAX – 416-946-8253

SUPPLEMENTAL INFORMATION – TABLE OF CONTENTS

MATERIALS AND METHODS 3

Cell lines and tranfections 3
Pooled shRNA screening methods 3
Computational scoring of pooled screens 5
mRNA expression profiling 5
Quantitative PCR methods 6
Dual color HCS-based competition assay 7
Immunofluorescence 8
Time-lapse imaging 9
Cell synchronization 10
Epitope tagging and western blotting 11
Cetuximab validation assay 11
CellTiter-Glo® based luminescent cell viability assay 11
Rescue/complementation of TTC31 and CD83 knockdown phenotypes 12
In vivo xenograft experiments 14
Soft agar assays 15
Expression correlation analysis for (1) HKDC1 expression versus RAS signaling and (2) PTTG1 expression versus DHFR expression in methotrexate sensitive and resistant cell lines 16
Collection of negative genetic interaction data from model systems and other human screens 17
Orthology mapping using MP-eggNOG 18
Assessment of conservation of the negative genetic interactions 18
Construction and analysis of genetic sub-networks 19
Construction and analysis of genetic sub-networks 19
Assessment of human annotation status in the DiE network 20
Assessment of overlap of top delta-GARP scores in PTEN query with cancer essentials 20
Determining PTEN signature from non-isogenic lines 21
Precision-Recall Plots 21
Meta-analysis calculating the overlap of our study with other genomic datasets 22

ACCESS TO SUPPLEMENTAL TABLES 23
SUPPLEMENTAL TABLE LEGENDS 23
SUPPLEMENTAL FIGURE LEGENDS 26
SUPPLEMENTAL REFERENCES 32
SUPPLEMENTAL FIGURES 36

Materials and Methods

Cell lines and transfections.

A full description of each cell line used, their sources, and their culture method are detailed in Supplementary Table 10. Transfections were done using Fugene (Promega) as per the manufacturer's protocol.

Pooled shRNA screening methods.

Pooled *sh*RNA screens were done as described previously (Blakely et al, 2011; Ketela et al, 2011; Marcotte et al, 2012). All *sh*RNA pools and constructs in this study were derived from the RNAi Consortium lentiviral libraries available from Sigma-Aldrich (Moffat et al, 2006; Moffat & Sabatini, 2006; Root et al, 2006). Briefly, lentivirus pools were generated from pooled lentiviral plasmid DNA. 4×10^7 HCT116 cells per replicate were infected with 80 k lentiviral *sh*RNA pools at an MOI of 0.3-0.4. After two days of selection in media containing 2 μg/mL puromycin (Sigma-Aldrich) to eliminate uninfected cells, genomic DNA was prepared from *sh*RNA-infected cell populations (Blood Maxi prep kit, Qiagen). Therefore, each hairpin was represented >200 times in the screening populations . Half-hairpin barcodes were prepared from genomic DNA samples using using 30µg of DNA obtained from at least 5×10^6 infected cells, so that 60-70 fold representation was obtained from the starting amount of gDNA. A master mixture for each sample containing 30µg of template DNA, 2x PCR buffer, 2x enhancer solution, 300nM each dNTP, 900µM each oligonucleotide primer (PCR_BF 5'-Biotin-AATGGACTATCATATGCTTACCGTAACTTGAA-3' and PCR_R 5'-TGTGGATGAATACTGCCATTTGTCTCGAGGTC-3'), 50mM $MgSO_4$, 45 units of Platinum Pfx polymerase (Invitrogen), and water to 1200 µl was made and divided into 100 µl aliquots. The amplification reaction was performed by denaturing once at 94°C for 5 minutes, followed by (94°C for 15 seconds, 55°C for 15 seconds, 68°C for 20 seconds) x30, 68°C for 5 minutes, then cooling to 4°C. The PCR product (178bps) was run on a 2% agarose gel to make sure that the amplified *sh*RNA sequence does not form cruciform structure (225bps). PCR products are immediately purified using the QIAquick PCR purification kit (Qiagen) to avoid the conversion of linear product to cruciform DNA and immediately digested with XhoI (New England Biolabs) for 2 hours at 37°C to generate a thermo-stable half-hairpin probe (~106

bps). This is then gel purified and remaining salts were cleared using a PCR purification kit (Qiagen) with two elutions of 30 µl of EB buffer (Qiagen). An average yield of 3 to 3.5 µg of each sample was obtained from this procedure. Probe hybridization onto UT-GMAP 1.0 microarrays (Affymetrix Inc) was done as described previously (Blakely et al, 2011; Ketela et al, 2011).

The pooled shRNA screen to identify genetic interactions with Cetuximab/Erbitux (Bristol-Myers Squibb and Eli Lilly & Company) was performed similarly to the HCT116 screens described above. Briefly, Lim1215 colon cancer cells were grown to a density of ~$2x10^8$ cells in McCoy's 5A medium supplemented with10% FBS. Cells were washed with PBS, trypsinized, re-suspended in warm medium, and counted. A 20 mL aliquot of the 80k human shRNA lentivirus pool and 8 μg/mL polybrene (Sigma-Aldrich) were added such that an MOI of 0.3 was achieved, based on optimization experiments. Twenty-four hours post-infection, the medium was replaced with fresh medium containing 3 μg/mL puromycin and cells were incubated for an additional 48 hours to clear the uninfected cells. Cell populations were quantified and 3 aliquots of $1.6x10^7$cells were pelleted by centrifugation and frozen down to make genomic DNA representing time zero (ie. T0). Also, six replicate populations of $1.6x10^7$ cells were plated into six 4-level EasyFill 2528cm^2 Cell Factories (Nunc #140360). Twenty-four hours later, the medium in each of three of the six replicates was replaced with fresh medium containing 0.01 μg/mL Cetuximab; the media in three untreated controls was replaced with normal McCoy's 5A full medium without Cetuximab. Six days post treatment, three aliquots of $1.6x10^7$ cells from each replicate were removed, pelleted, and frozen (ie. T6 time point) while one aliquot of $1.6x10^7$ cells was re-plated for further growth. T12 and T18 time points were collected the same way as the T6 time point for the cells growing in the presence or the absence of Cetuximab. Genomic DNA was prepared from cell pellets using the QIAmp Blood Maxi kit (Qiagen), precipitated using ethanol and NaCl, and resuspended at 400 ng/mL in 10 mM Tris-HCl, pH 7.5. shRNA populations from cell lines were amplified via PCR and applied to UT-GMAP microarrays (Affymetrix) as described (Ketela et al, 2011; Marcotte et al, 2012).

Computational scoring of pooled screens.

Hairpin scoring was as described previously (Marcotte et al, 2012). Briefly, expression intensities from triplicate screens were averaged, and individual hairpin features were filtered from further consideration if the initial mean $\log_2$-expression intensity was below 7.5. Measurements collected over multiple time points were then integrated using the *sh*RNA Activity Ranking Profile score (shARP), as shown in Equation 1:

$$shARP = \frac{1}{n-1} \cdot \sum_{i=1}^{n} \left(\frac{\Delta y_i}{\Delta x_i} \right)$$

where *n* is the number of time points, Δy is the change in expression intensity at t_i relative to t_0, and Δx is the number of doublings for the cell line at t_i relative to t_0. shARP scores were determined for each of the 78,432 library hairpins, and were then used to calculate the Gene Activity Ranking Profile score (GARP) by averaging the two lowest shARP scores. A significance value was assigned to each GARP score through bootstrapping, where the shARP scores were randomLy permuted 1000 times, GARP scores recomputed, and a p-value determined by the frequency with which the actual GARP score was lower than the permuted GARP scores. To facilitate comparisons between screens, GARP scores were Z-score normalized. Finally, to obtain the top hits from the GARP score, we subtracted the GARP of the parental cell line from the GARP of the mutant (dGARP) on a gene-by-gene basis and this was sorted to obtain the rank order list of hits.

mRNA expression profiling

Transcriptome analysis for all the HCT116 cell lines screened were generated using Affymetrix Genechip® Human Gene 1.0 ST arrays. Total RNA was extracted from cells grown to 70% confluency using RNAeasy assay kit (Qiagen). One microgram of RNA for each sample was processed using the Affymetrix GeneChip® Whole Transcript Sense Target Labeling Assay. The array represents at least 26 probes spread across the entire gene sequence for every single 28,869 genes in the human genome. Hybridization targets were obtained following a double amplification procedure according to the manufacturer's instructions. The GeneChip® WT cDNA Synthesis Kit, WT cDNA Amplification Kit, and the WT Terminal Labeling Kit (Affymetrix, Inc., Santa Clara, CA) were used for the sample preparation. Hybridization cocktails were hybridized to the microarrays and incubated in a rotating hybridization oven at 45 °C and 60 rpm for 16 hours. Following hybridization, the

arrays were washed and stained on a GeneChip® Fluidics 450 workstation (Affymetrix, Santa Clara, USA). The arrays were scanned using the Affymetrix GeneChip® Scanner 3000 7G plus (Affymetrix, Santa Clara, USA). An example of the cumulative percentage of expressed genes, Affymetrix controls including expressed and non-expressed genes, as well as negative controls (e.g. intron sequences, miRNAs) for a given sample is shown in Supplementary Fig. 14A. Genes were filtered as not expressed if the mean $\log_2$-expression intensity was below 6.75. Using this cutoff, a Venn diagram was constructed to summarize the number of genes that were expressed in each of the HCT116 cell lines and also targeted by the TRC lentiviral pooled library that was used to screen these cell lines (Supplementary Fig. 14B).

Quantitative PCR methods.

Cells were plated into 24-well plates (5 x 10^4 cells/well) or 6-well plates (200,000 cells/well) and infected the following day with the appropriate *sh*RNA. Twenty-four hours post-infection, cells were selected with puromycin and allowed to grow for an additional 48 hours. cDNA samples were produced with RevertAid H Minus Reverse Transcriptase (Fermentas) or SSIII (invitrogen). From 1 ug of total RNA isolated with the RNeasy kit (Qiagen), transcript levels were measured with the ABI Prism 7300 Real-Time PCR System or Biorad CFX96, using the Power SYBR Green Master Mix (Applied Biosystems) or Platinum® SYBR® Green qPCR SuperMix-UDG (Invitrogen). Relative expression levels were calculated by cycle threshold (Ct) relative quantification (2-ddCt), using either glyceraldehyde-3-phosphate dehydrogenase or Actin as the endogenous control, at least in triplicate for each sample.

The detection primers used were

DHFR-Forward :AAATGAGCTCCTTGTGGAGG;

DHFR-Reverse :ACCTGGTTCTCCATTCCTGA;

ENO2-Forward :TGGTCAAATGGGTCCTCAAT;

ENO2-Reverse :CCTTCCCGATACATCA CTGG;

RASSF2-Forward :CACAGTCAGGGGCTTCAGA;

RASSF2-Reverse :AGATGCAGGATGACAACGAA;

HKDC1-Forward :GGGGAGGATTTGGAGACAAT;

HKDC1-Reverse :CCCA AGTACATCCCACTGGT;

GAPDH-Forward :GAGGTCAATGAAGGGGTCATTG;

GAPDH-Reverse :GGTGAAGGTC GGAGTCAACGG.

CD83-Forward :CCCTGCACAGCGTAAAGAAGA

CD83-Reverse :AAGCTCGTTCCATGCCAGC.

Actin-Forward :CACTCTTCCAGCCTTCCTTC

Actin-Reverse :GGATGTCCACGTCACACTTC.

Dual color HCS-based competition assay.

To validate the candidate negative genetic interactions or synthetic lethal interactions from the primary screens, we first selected a list of hits based on the GARP scores ($p<0.05$), SHARP scores ($p<0.01$), genes that had significant GARP scores and were also differentially expressed in the mutant lines versus the parental cells ($p<0.05$), and finally genes that had yeast orthologs with significant GARP scores ($p<0.05$)(Supplementary Figure 2B). In the selection of yeast orthologs, we included paralogs of human genes based on Inparanoid, P-POD, OrthoMCL and Genecards in order to maximize the number of candidate conserved genetic interactions in our validation list (Chen et al, 2006; Heinicke et al, 2007; Ostlund et al, 2010; Safran et al, 2010).

Dual color competition assays were developed (Torrance et al, 2001) for all five queries, where we monitored live cells by time-lapse imaging over a period of seven days following *si*RNA transfection against target genes listed in Supplemental Table 4. Briefly, red (mRFP)_ or green (EGFP) fluorescent proteins were integrated into each of the HCT116 cell lines that were screened using pLJM5 (RFP-hygro) or pLJM7 (GFP-hygro) lentiviral constructs, respectively (J. Moffat, unpublished). Stable cell lines expressing red or green fluorescent proteins were generated following a 2-week selection in 100μg/mL hygromycin and tested for growth rate either alone or in a mixing experiment. For the competition assays, mutant query cells that were green and parental cells that were red were seeded in 384-well plates for reverse transfection with siRNAs from an orthogonal RNA interference library (Dharmacon). Reverse transfections were done in duplicate using the BiomekFX liquid handling robot. Cells were seeded such that un-transfected cells could reach 90-100% confluence within seven days. Reverse transfection was done with Lipofectamine RNAiMax using Dharmacon SMARTpool *si*RNA reagents at a concentration of 30nM using liquid handling robots (Biomek FX). Twenty-four hours post-transfection (ie. day 1), plates were imaged using the Opera high-content automated imaging microscope (PerkinElmer) with a 4X objective to capture the entire well. Subsequently, plates were imaged each day at the same

time over the course of seven days. Cells from each image were segmented using Acapella™ (PerkinElmer) and the number of red and green cells was estimated using a spot detection algorithm in the Acapella software. Cell count was normalized for growth differences between the parental and mutant lines using un-transfected cell numbers and growth defects in the mutant cells compared to the parental cells were calculated at each time point as the ratio of red and green cells. Each secondary screen screen was formatted with 32 mock-transfected samples in each of the 384-well plates, which were subsequently used to develop reference features. The relative decrease in the number of mutant cells compared to parental cells may occur differentially for each putative negative genetic interaction, and knockdown of the target gene may decrease over the course of the seven day experiment, resulting in recovery of the mutant query cells relative to parental control cells. Therefore, the lowest cell count across these time points was used to compute the significance of the mutant population depletion compared to the non-targeting mock-transfected cells. We considered the validation experiment a success if the mutant cells grew a rate that was at least two standard deviations below the medium of the mock control distribution.

Immunofluorescence.

Immunofluorescence staining was typically done after infecting HCT116 cells with the appropriate lentiviruses and plating on laminin-coated glass bottomed plates (Sigma-L2020). For visualization of mitotic components, cells were fixed in ice-cold methanol at -20°C for 10 minutes. After blocking in either 1% goat serum in PBS or 0.2% gelatin from cold water fish (Sigma) in PBS (PBS/FSG) for 15 min, cover slips were incubated with primary antibodies in blocking solution at the following concentrations for 30 minutes to an hour: mouse anti-Centrin (gift from Jeff Salisbury) 1:1000, rabbit anti-Pericentrin (Abcam ab4448) 1:1000, mouse anti-NEDD1 (Abcam ab57336) 1:500, AuroraB (AIM1-611082) 1:500, Cenp-E (Sigma-C7488) 1:1000, BubR1 (Immunoquest-IQ199) 1:1000. After three washes with 0.2% PBS/FSG, cells were incubated with 1:500 dilutions of secondary antibodies for 30 min to an hour (anti-mouse conjugated to Alexa Fluor 488 and anti-rabbit conjugated to Alexa Fluor 594; Invitrogen Molecular Probes). Cells were then counterstained with 1 μg/mL Hoechst to visualize DNA. After washing with 0.2% PBS/FSG, cover slips were mounted on glass slides by inverting them into mounting solution (Prolong Gold antifade; Invitrogen Molecular Probes). For γH2AX immunostaining, HCT116 cells were seeded on coverslips and transfected with either non-

targeting siRNAs or siRNAs against specific targets (*TOPBP1*, *FANCD2, USP1* or *UAF1*), where the siRNAs were purchased from siGENOME SMARTpool *si*RNAs (Dharmacon). After 48 h, cells were fixed with 4% paraformaldehyde for 10 min followed by three washes with 1X PBS. Cells were permeabilized with 0.3% Triton X-100 for 10 min and blocked in 10% goat serum containing 0.5% NP-40 and 0.5% Saponin for 30 min. Cells were then immunostained with a rabbit anti-γH2AX (Ser139) antibody (#2577; Cell Signaling) at 1:500 for 1 h followed by incubation with a goat anti-rabbit IgG-Alexa Fluor 488 (Invitrogen Molecular Probes) secondary antibody at 1:1000 for 1 h. Nuclei were then stained with 1 μg/mL DAPI for 15 min and imaged. Three dimensional image datasets were acquired with either a DeltaVision CoreDV system (Applied Precision) equipped with an IX71 microscope (Olympus), a CCD camera (CoolSNAP 1024x1024; Roper Scientific) using a 60x NA 1.42 plan-Apochromat objective (1x1 binning) or a WaveFX spinning disc confocal system (Quorum Technologies) equipped with an ultra-cooled 512 back-thinned EM charge-coupled device camera (Hamamatsu). Images were acquired with a 63× HCX Plan-Apochromat oil objective with an NA of 1.4. Image stacks (0.2 μm apart for each section) were de-convolved using the softWoRx v4.5 software package (Applied Precision) and are shown as maximum intensity projections.

Time-lapse imaging.

HeLa cells stably expressing murine *NEDD1*::GFP from a bacterial artificial chromosome (Lawo et al, 2009) were infected with indicated *sh*RNAs and following two days of selection with Puromycin were seeded in Lab-TekII chambers (Nalge Nunc). Cells were imaged with a DeltaVision CoreDV system (Applied Precision) equipped with an IX71 microscope (Olympus), a CCD camera (CoolSNAP HQ2 512x512; Roper Scientific), 37°C and 5 % CO_2. Image stacks were acquired at 10 min intervals for 24 h with a 60X NA 1.42 plan-Apochromat objective using 2x2 binning. Image stacks (30 z-slices) were then de-convolved and are shown as maximum intensity projections.

Cell synchronization

HCT116-*PTTG1*$^{+/+}$ and HCT116-*PTTG1*$^{-/-}$ cells were grown in Mc Coy's 5A media (10% FBS, 1% Pen-Strep). At 30-40% confluency, cells were washed with 1X PBS and incubated in Mc Coy's 5A media containing 4mM thymidine for 16 hours to block the cells in S phase (first block). After the first thymidine block, cells were washed with 1X PBS to remove

thymidine and fresh media was added and incubated for 7.5 hours to release the cells. Following release, fresh media containing 2mM thymidine (to block cells in G1/S and S phase) or 200mM nocodazole (to block cells in G2/M and M) was added and cells were incubated for 16 hours (second block). After the second block, thymidine or nocodazole was removed by washing with 1X PBS and the cells were released into fresh media. Lysates were prepared at different times following release and were probed for different Cyclin proteins by western blotting using Cyclin Antibody Sampler Kit #9869 (Cell signaling).

Epitope tagging and western blotting.

TTC31 gene containing plasmid (HsCD00336882) was obtained from PlasmID in pBluescript vector and converted to Gateway-compatible construct by adding appropriate "att B1 and B2" sites. Forward primer GGGG ACA AGT TTG TAC AAA AAA GCA GGC TNN-ATG GCG CCG ATT CCA AAG and Reverse primer GGGGAC CAC TTT GTA CAA GAA AGC TGG GTN TCT GGC CTG AGA CAG ATG were used to amplify *TTC31* and clone into the pDONR221. The Gateway-compatible destination plasmids pLD-puro-CcVA or pLX304 were used to generate *TTC31* expression constructs with either a VA tag or a V5 tag, respectively (Mak et al, 2010; Yang et al, 2011). To construct *TUB3B*-GFP and Histone-BFP, we used the pLD-puro-Cc-tGFP. This destination plasmid was constructed using a turbo-GFP amplicon, cloned into pLD-puro-CcVA using the XbaI and BstBI sites using the primers XbaI-turboGFP (GATCGATCtctaga GAGAGCGACGAG AGCGGCCT) and BstBI-turboGFP-stop (GATCGATCttcgaa TTATTCTTCAC CGGCATCTGCATC). The turbo-GFP does not contain an ATG site, but includes a stop codon. HEK293T cells were then transfected along with lentivirus packaging plasmid and envelope plasmid (psPAX2 and pMD2.G) to make viral particles packaged with epitope-tagged *TTC31*. This lentivirus was used to infect target cells and expression was examined by western blotting. Briefly, cells were plated in 6-well plates (~1 x 10^5 cells/well) and infected with the appropriate virus. Twenty-four hours post-infection, virus was removed and cells were selected with puromycin for 48 hours. Cell lysates were harvested in RIPA buffer 4-5 days after infection. Western blot analysis was performed using antibodies directed against the Flag epitope (Abcam M2) or V5 (Abcam-ab9116) at a dilution of 1:5000.

For drug treatment assays (Fig. 6C) indicated cells were treated with 7.5 μg/mL Cisplatin or vehicle control for two hours and the next day cells were lysed in RIPA buffer with protease and phosphatase inhibitors.

Lysates prepared from knock down experiments in several instances, were blotted and probed with antibodies raised against ZC3H13 (Abcam-ab70802), YAP1 (Cell Signaling-4912), β-actin (Abcam-ab8226), PTEN (cell Signaling-9559), p-AKT (Cell Signaling-9275S), PTTG1 (Abcam-ab3305), ESPL1 (Abcam-ab3762), MUS81 (Abcam-ab14387), DHFR (Abcam-ab49881).

Cetuximab validation assay.

Lim1215 cells were infected in suspension with individual shRNA and plated in triplicates on 24-well plates ($5x10^3$ cells/well, 25ml virus/well, 8 mg/mL of polybrene). Twenty-four hours post-infection, the medium was replaced with fresh medium containing 3μg/mL puromycin. After 48 hours incubation, the medium was replaced with fresh full growth medium or the medium containing Cetuximab (0.01 μg/mL) and incubated for an additional four days. Culture dishes were washed with warm PBS to remove dead cells, and surviving cells were collected by trypsinization at 37°C and counted using a Hemocytometer. LacZ shRNA was used as a control.

CellTiter-Glo® based luminescent cell viability assay.

HCT116 cells were infected in suspension with individual shRNA and plated in triplicates on 96-well plates ($0.8\ x10^3$ cells/well, 5μl virus/well, 8μg/mL of polybrene). Twenty-four hours post-infection, the medium was replaced with fresh medium containing 3μg/mL puromycin. After 48 hours incubation, the medium was replaced with fresh full growth medium or the medium containing Cetuximab (0.01 μg/mL) and cells were incubated for an additional four days. CellTiter-Glo® Luminescent Cell Viability Assay was performed according to manufacture protocol. Briefly, CellTiter-Glo® Reagent was added directly to the wells containing cells cultured in full medium and luminescence was recorded 10 minutes after reagent addition using Synergy 2 plate reader (BioTek).

Rescue/complementation of TTC31 and CD83 knockdown phenotypes.

TTC31 gene containing plasmid (HsCD00336882) was obtained from PlasmID in pBluescript vector and converted to Gateway-compatible construct by adding appropriate "att

B1 and B2" sites and cloned into pLX304 plasmid to use as a wild type expression construct. Gene synthesis was used to introduce silent mutations in the ORF region of TTC31 corresponding to the binding sites of the two hairpins as shown below to make shRNA resistant (shR) constructs.

```
TTC31 Ref seq     : GAG GAT TAC GGC GAA GAG GAC ATA GTG
TRCN0000138173 :        GAT TAC GGC GAA GAG GAC ATA
Mutated on ORF    : GAG GAT TAC GGC GAG GAG GAT ATA GTG
```

```
TTC31 Ref seq     : GAG GAG CGC ATG AAA CAG AAA GCA GAG
TRCN0000137918 :        GAG CGC ATG AAA CAG AAA GCA
Mutated on ORF    : GAG GAG CGC ATG AAG CAG AAG GCA GAG
```

For CD83, human CD83 cDNA (IMAGE ID 4818856) was cloned into the destination plasmid pLD-puro-Cc-tGFP. CD83 shRNA resistant construct was created using GENEART Site-Directed Mutagenesis System (Invitrogen) with the following mutations:

```
CD83 Ref seq        :     TAT TCC CTG AAG ATC CGA AAC ACT ACC
TRCN0000056918  :            CC CTG AAG ATC CGA AAC ACT A
Mutated on ORF      :     TAT TCA TTA  AAA  ATT CGG AAT ACC ACC
```

Stables cell lines expressing both the wild type and the shRNA resistant constructs were generated as described above and infected with hairpins to examine whether or not the shR constructs could rescue the defects in proliferation. For example, HCT116 cells were infected with lentiviruses expressing WT-CD83-GFP or shR-CD83-GFP and stable lines were generated. GFP expressing cells were infected in suspension with individual lentiviruses: sh1CD83, shLacZ or shPSMD1 and plated into 12 well plates at 10^4cells/well, 50μl virus/well, 8μg/mL of polybrene. Twenty-four hours post-infection virus was removed and the medium was replaced with fresh medium. Six days later, cells were harvested by trypsinization, stained with 7-AAD (BioLegend) and counted using a BD FacsCalibur analyzer with CellQuest Pro software and analyzed using FlowJo (v.7.6.5). Each sample was run for 204.8 seconds at a constant flow rate of 35l/min and the number of live cells in GFP-positive populations was determined.

In vivo xenograft experiments.

NOD/LtSz-scid/scid (NOD/SCID) mice were maintained under defined conditions at the Ontario Cancer Institute under conditions approved by the Animal Care Committee of the Ontario Cancer Institute. Lim1215 cells were infected with individual shRNA lentiviruses sh1_CD83, sh2_CD83, or control shLacZ. Twenty-four hours post-infection, the medium was

replaced with fresh medium containing puromycin (3 μg/mL) and cells were incubated for an additional 48 hours. Puromycin resistant Lim1215 cells ($1x10^6$) were washed twice with PBS, resuspended in a 1:1 mixture of media and matrigel (BD Biosciences), and injected subcutaneously into the lower left and upper right flanks. After 10 days, when tumor size reached $0.2cm^3$, half (n=10) of the mice of every group (n=20) began receiving intra-peritoneal injection of Cetuximab (10 mg/kg) twice weekly, while the other half of the mice (n=10) were given mock injections of PBS on the same days. Tumor sizes were monitored on the injections days. Twenty-five days later mice were sacrificed, and tumors were measured and weighed.

A bootstrap approach was used to assess the significance of the difference in tumor growth as follows. Each tumor was associated with a treatment T (either Cetuximab or Mock) and a hairpin H (either Untransduced, shLacZ or sh1_CD83). The mean of tumor weights associated with each combination of treatment and hairpin was denoted $\overline{W}_{H,T}$. With this, the following quantities were defined

$$\text{Average Ratio of Weights for hairpin } H = WR_H = \frac{\overline{W}_{H,Cetuximab}}{\overline{W}_{H,Mock}}$$

$$\text{Relative Effect of hairpins } H_1 \text{ and } H_2 = RE_{H_1,H_2} = \frac{WR_{H_1}}{WR_{H_2}}$$

The analysis was designed to assess whether, as indicated by the synthetic lethal relationship between CD83 and Cetuximab observed in vitro, CD83-silenced tumors treated with Cetuximab exhibited greater relative decrease in weight when compared to CD83-normal tumors subjected to the same treatment. Expressed in terms of the quantities defined above, these questions were expressed as the alternative hypotheses $RE_{sh1CD83,Lac\ Z} < 1$ and $RE_{sh1CD83,Untransduced} < 1$. To ensure bootstrap distribution symmetry, $\log(RE_{sh1CD83,Lac\ Z}) < 0$ and $\log(RE_{sh1CD83,Untransduced}) < 0$ were used in the calculations instead. To test these hypotheses, 10^6 bootstrap samples were generated from the tumor weights data. The sets of weights associated with each treatment and hairpin pair were separately sampled with replacement and aggregated to form the bootstrap sample, in order to adequately represent the experimental structure as outlined in Efron and Tibshirani (Efron & Tibshirani, 1993). Symmetry of the distribution of log(RE) bootstrap estimates was verified using visual

inspection and Quantile-Quantile plots against the Normal distribution. Estimates of bootstrap bias for log(RE) estimates were less than 2.5% of estimates of log(RE) standard error in all cases. Using these bootstrap samples, single-tailed p-values were calculated for each hypothesis test following Efron and Tibshirani (Efron & Tibshirani, 1993). Displayed error bars were also estimated by bootstrap for each of the plotted bars representing the Weights Ratios $(WR_{Untransduced}, WR_{Lac\ Z}, WR_{sh1CD83})$.

Soft agar assay.

Soft agar assays were performed using Human fibroblast (BJs) stably expressing hTERT, SV40 Large and small T antigens and *HKDC1* (or the GFP control). Cells were maintained in DMEM supplemented with 10% FBS (Wisent). BJs cells expressing HTERT (BJ-hTERT) were generated in two steps. BJ-hTERT were transduced and selected to express SV40 Large and small T under the control of the CMV promoter. Lentiviral vector used for the stable expression of the SV40 large and small T were generated by BP and LR reactions (invitrogen) using pLenti CMV/TO SV40 small + Large T (Addgene plasmid 22298), pDONR 223 (invitrogen) and pLenti CMV Hygro DEST (Campeau et al, 2009). Cells were selected for one week with Hygromycin at 100 μg/mL. Following stable selection, established BJ hTERT-LTsT cells were transduced with *HKDC1* or GFP and stable populations were selected using puromycin at 2 μg/mL. The *HKDC1* lentiviral expression vector was generated by LR reaction using a sequenced verified entry clone from the ORFeome library v7.1 (Open Biosystems) and an N-terminal VAP tag destination vector (Mak et al. 2010, Mak and Moffat 2012). BJ-hTERT-LTsT cells were incubated with virus for 24 hours, selected for 2 days with puromycin and grown for 7 additional days prior to the colony formation assay. For the soft agar assay, type VII agarose (Sigma, A4018) was prepared as a 3% solution in water with 0.9g/L NaCl. A 1% bottom layer was prepared by diluting the agarose 1:3 with DMEM+10%FBS. 6-well plates were coated with a bottom layer of 1% agarose and allowed to solidify for 30 minutes at room temperature. The top layer was prepared by creating a 0.8% agarose suspension containing 12000cells/mL. 24000 cells were plated per well in duplicate. Cells were fed with DMEM containing 10% FBS once per week. After 3 weeks, cells were stained with 0.05% crystal violet, washed with PBS and photographed. Over-

expression of *HKDC1* was confirmed by Western blotting using anti-FLAG antibody (Abcam-ab49763).

Expression correlation analysis for (1) HKDC1 expression versus RAS signaling and (2) PTTG1 expression versus DHFR expression in methotrexate sensitive and resistant cell lines.

To examine the association between HKDC1 expression and RAS signaling dependence, two multi-tissue expression studies were used: the Cancer Cell Line Encyclopedia (CCLE, http://www.broadinstitute.org/ccle) of 807 cell lines, and the Expression Project For Oncology (EXPO) study of 2158 tumor samples (GEO: GSE2109, http://www.intgen.org). Both used the Affymetrix U133plus2.0 platform. CCLE data was obtained in RMA-processed form, while EXPO samples were processed using the Aroma Affymetrix R package (http://www.aroma-project.org). Probeset values were averaged per gene. Using the expression data, a RAS pathway dependency signature (Loboda et al, 2010) was used to score each sample in the CCLE and EXPO datasets, following the procedure detailed elsewhere (Loboda et al, 2009). Briefly, 147 gene symbols from the *RAS* signature were matched against genes profiled on the U133plus2 platform, yielding expression profiles for 143 signature genes. Each study was scored separately. The signature's two branches reflect genes up- and down-regulated in a *RAS* signaling-dependent state. The *RAS* dependence score for each sample S was calculated as follows:

$X_{S,G}$ - Expression of gene G in sample S

$\overline{X_G}$ - Averaged expression of gene G across all samples in study

N_{UP} and N_{DOWN} - Number of genes in the up and down branches of the signature

$$RAS-dependence_S = \frac{1}{N_{UP}}\left[\sum_{\substack{\text{U gene in}\\ \text{UP branch}}} \log 10\left(\frac{X_{S,U}}{\overline{X_U}}\right)\right] - \frac{1}{N_{DOWN}}\left[\sum_{\substack{\text{D gene in}\\ \text{DOWN branch}}} \log 10\left(\frac{X_{S,D}}{\overline{X_D}}\right)\right]$$

Higher scores suggest greater reliance of the tumor or cell line on *RAS* pathway signaling, and potential decrease in proliferation rate and viability should this signaling be inhibited.

Examination of the *RAS* pathway dependence scores obtained for each study revealed bimodal score distributions, with one major peak on either side of zero. These score peaks

were modeled using a two-component Gaussian mixture model fitted to the score distribution using the R package mixtools (http://www.jstatsoft.org/v32/i06/), with each study was analyzed separately. The model provided a sample-level probability of being in the low or high *RAS* dependence groups. Each sample was assigned to the more probable group. *HKDC1* expression levels were observed to be low for a large fraction of samples in both studies. Probeset-level present/absent calls available for the CCLE data suggested that log_2 *HKDC1* probeset expression values of approximately four were at the array background signal level for this dataset. However, similar present/absent calls were not available for the EXPO dataset. To address this, a Gaussian mixture approach was used to model *HKDC1* expression values in each study as present or absent based on intensity. With the above classifications, we checked for potential enrichment of samples with clear *HKDC1* expression and high *RAS* signaling dependence. For both the CCLE and EXPO, a strongly significant enrichment of samples with clear *HKDC1* expression and high *RAS* dependence was observed (Fisher's Exact Test, CCLE: $p < 2.2 \times 10^{-16}$, Odds Ratio 11.25; EXPO: $p < 2.2 \times 10^{-16}$, Odds Ratio 5.51). In addition, considering just numeric *HKDC1* expression values, a consistent positive correlation with RAS dependence score was observed (Spearman Rank Correlation, CCLE: $\rho = 0.51$, $p < 2.2 \times 10^{-16}$; EXPO: $\rho = 0.50$, $p < 2.2 \times 10^{-16}$).

Separately, we examined the possible association between *PTTG1* and *DHFR* expression. For this, the GSK300 panel of 318 cell lines was obtained in MAS5-processed form from ArrayExpress (http://www.ebi.ac.uk/arrayexpress). The data was quantile-normalized, replicates averaged, log_{10}-transformed, and each gene Z-scored across cell lines, followed by Pearson correlation of *PTTG1* and *DHFR*. An additional question of interest was the relative expression of *DHFR*, *PTTG1* and other genes in methotrexate resistant versus sensitive lines. For this, the GEO dataset GSE16648, described earlier, was used. Data was quantile-normalized, replicates averaged, and the log_2 ratio of resistant over sensitive expression was calculated for each paired cell line. This was performed for genes *PTTG1*, *DHFR*, *ESPL1*, *FPGS*, *GGH* and *SLC19A1*.

Collection of negative genetic interaction data from model systems and other human screens.

To identify interactions which directly (i.e. human interactions) or indirectly (i.e. interactions of orthologs in other species) support the relationships uncovered in our screen,

we collected an exhaustive dataset of known genetic and physical interactions from human, yeast, worm, fly, and mouse by combining data from the iRefWeb (Turner et al, 2010) and BioGrid (Stark et al, 2011). Both resources curate interactions from various primary sources and thus comprise the largest collection of interaction data available. We also integrated data from a recent *KRAS* synthetic lethal screen (Luo et al, 2009), and an updated set of genetic interaction data from yeast (Costanzo et al, 2010), neither of which was available in the curated databases. For the yeast genetic interaction data, we chose a cut-off for the absolute genetic interaction score at 0.08, which refers to a p-value of 0.05 (Costanzo et al, 2010; Koh et al, 2010). For *KRAS*, we used yeast genetic interactions of *IRA1* and *IRA2* as deletion of these mutants mimic constitutively active form of KRAS (Tanaka et al, 1989; Tanaka et al, 1990a; Tanaka et al, 1990b). We then mapped the set of the most significant genetic interactions to these databases using the MP-eggNOG procedure described below.

Orthology mapping using MP-eggNOG.

The existing orthologous mapping methods such as eggNOG (Muller et al, 2010), OrthoMCL (Chen et al, 2006), or P-POD (Heinicke et al, 2007) comprise many paralogs. However, these orthologous groupings contain many-to-many mappings between genes that do not necessarily reflect meaningful relationships (e.g. divergent paralogs with distinct functions). Orthologous group based methods are more sensitive for large evolutionary distances (e.g. between yeast and human) compared to methods based on bi-directional best BLAST hits such as Inparanoid (Ostlund et al, 2010). We implemented a stringent approach based on eggNOG 2.0 resulting in a mapping of the most probable ortholog for each human gene, which we describe here. We adapted an initial ortholog mapping using a combination of eukaryotic and general orthologous groups (euNOGs, COGs, and KOGs in the eggNOG terminology) from the eggNOG database (Muller et al, 2010). These orthologous groups contain orthologous pairs of the same genes in different species, but might also comprise paralogs and their respective orthologs. Consequently, we determined for each human gene the most probable orthologous gene in the species of the evolutionary older, less complex clades. To do this, we detected for each human gene the most similar sequence for each species of interest within the largest group it has been assigned to (by measuring similarity using the Smith-Waterman algorithm with BLOSUM62 substitution matrix, gap opening penalty 2, extension penalty 1), since the largest group should be most sensitive in picking up

distant relationships. As a consequence, most human genes have been assigned to exactly one gene in each of these species where several human genes may target the same ortholog (e.g. certain kinases that have undergone gene duplication events leading to multiple paralogs/co-orthologs will have the same yeast gene as an ortholog). Our goal was to report only highly likely orthologous relationships, so we further pruned potential false positive relationships based on shared domains. Thus, we removed all pairs of the detected *bona fide* orthologs with a sequence identity <25% over the complete sequence and these forming alignments with less than 75% coverage for any of the two sequences. This procedure is represented in Supplementary Figure 3. For the orthologs used as supporting evidence of a genetic interaction in our dataset (see below), we manually inspected the orthology assignment and removed further false positive ortholog relationships if contradictory annotations were present (e.g. molecular function) of the sequences. The sequence alignment for each ortholog pair can be found in Supplementary Table 11.

Assessment of conservation of the negative genetic interactions.

While genes themselves may be conserved, there is conflicting evidence whether the corresponding genetic interactions are conserved over large evolutionary distances (Byrne et al, 2007; Tarailo et al, 2007; Tischler et al, 2008). Therefore, we tested the significance of the compiled supporting evidence as follows. First, we calculated empirical p-values by permuting the experimentally determined human genetic interaction network 500 times, and determined the probability of obtaining a greater or equal amount of evidence for each of the query genes. Permutations were carried out by randomly assigning one of the ~16,000 genes represented in the shRNA library to each node in the network, while retaining the evidence networks, which simulates random *sh*RNA experiments equivalent to the one we performed. Due to the bi-partite nature of the network, this is equivalent to an edge re-wiring strategy. Overall, the overlap between the human and model organism networks is not significant using this approach. The results of this analysis and the individual evidences uncovered are summarized in the Supplementary Table 7.

Construction of a high confidence network.

The high-confidence differential essentiality or DiE network (Figure 2) comprises all interactions which tested positively in the secondary screen at the 80% confidence level and all hits in the top 5% (p-value <0.05) that have any evidence in form of a human genetic or

physical interaction, or in the respective orthologous networks in mouse, fly, worm, or yeast. This high confidence network comprises 264 genes connected by 291 interactions and is represented in Figure 2.

Construction and analysis of genetic sub-networks.

In order to investigate the relationships between the genes that have been found genetically interacting to the same query gene, we created sub-networks by gathering interactions between them in human as well as in model organisms. For the latter, we used MP-eggNOG. Negative genetic interactions as observed in other model systems tend to be more related to each other compared to a random selection of genes, indicating that the experimentally-determined interactions tend to share complexes or functional modules (Costanzo et al, 2010). We tested this by assessing the significance of the observed structure within each sub-network as measured by their average clustering coefficient (Barabasi & Oltvai, 2004). The sub-networks were compared against a random model generated by permutations of the evidence networks (i.e. the physical and genetic interaction networks from fly, worm, mouse, yeast, human). In order to avoid reporting structural features of these input networks alone which will be reflected in the projected sub-networks, we applied a conservative random model: for each input network, we generated 500 random networks with the same structure as the original network (ie. retaining same degree and clustering coefficient distribution) by node shuffling. We then selected all interactions that are shared by nodes in the top 5% of each screen for the original (constituting the sub-network) and the randomized networks (constituting the random background networks). We integrated each type of interaction (genetic and physical interactions from each species) into a unified sub-network for each query gene (*BLM*, *MUS81*, *PTEN*, *PTTG1*, *KRAS*). We then computed the average clustering coefficient of the original sub-network and of the sub-networks of the random trials. We determined empirical p-values reflecting the increase of cross-talking genes picked up by each screen against the background as the empirical probability of observing the same or higher average clustering coefficients. We found a statistically significant increase in the average clustering coefficient for several of the sub-networks (Supplementary Table 8). Networks were visualized using Cytoscape (Cline et al, 2007).

Assessment of human annotation status in the DiE network.

We assessed the annotation status of our top dGARP hits using GO annotations obtained from the UniProt-GOA consortium (Dimmer et al, 2012) downloaded at the 10th of January 2013. Each gene that has at least one annotation that had not been electronically transferred (ignoring all entries with GO IEA evidence code) was counted as 'annotated'. In total, we found that 23% of the genes picked up in our screen lack any experimentally derived evidence.

Assessment of overlap of top delta-GARP scores in PTEN query with cancer essentials.

In order to assess the general applicability of the digenic relationships derived from the isogenic colon cancer cells for other non-isogenic cancer types, we systematically compared data generated from cancer-specific essentiality screens from breast, ovarian, and pancreatic cancers to the data from our isogenic screens (Marcotte et al, 2012). As a proof of principle, we chose to study this effect in PTEN/PI3K-dependent versus PTEN/PI3K-independent cell lines. In order to first classify the non-isogenic lines into two groups, those that are dependent on the PTEN/PI3K pathway or not, we used the CCLE database (Barretina et al, 2012) as downloaded on the 20th of October 2012, and assessed the mutation status of PTEN and PIK3CA genes in cell lines evaluated in the Marcotte et al. screens (Marcotte et al, 2012). Each mutation was classified as relevant if it was found in the literature or in UniProt (UniProt, 2012) described as an either PTEN inactivating or PIK3CA activating mutation. Coding mutations in close proximity to these described mutations have been categorized as *bona fide* equivalent mutations. We also searched the literature and annotated additional cell lines not known to carry these mutations (e.g. SW1990 and ASPC1), but that display constitutively active AKT due to some other mechanism (Cheng et al, 1996; Halilovic et al, 2010). In total, PTEN or PIK3CA status for 48 cell lines was uncovered (Supplementary Table 12) including 26 PTEN/PIK3CA-dependent cell lines and 22 PTEN/PIK3CA-independent cell lines, hereafter referred to as “PTEN*” and “WT”, respectively. We determined the overlap of the genes with the “m” most significant dGARP scores from the PTEN screen (the DiE profile) with the “n” top cancer essentials from non-isogenic lines by computing the Jaccard index (Jaccard, 1901). The Jaccard index is defined as the intersection between “m” and “n” divided by their union. We then sorted each set due to the resulting value from high to low and plotted them as shown in Figure 7B for m=n=750 which represents genes with dGARP/GARP score

with a p-value <0.05. We tested several cut-offs for n and m, resulting in a consistently similar scenarios as represented by an example in Figure 7B: the top PTEN* cell-lines exhibit higher Jaccard indices on average. We tested the significance of the difference between the two set as their shift in the mean of the Jaccard distributions (with n=750, m=750) using a one-sided t-Test (p-value=0.0002). A box-plot summarizing this data has been created in R using the ggplot2 package. From this we determined the signature of PTEN dependency using genes that are more frequent within the top 5% of the PTEN* lines compared to WT lines (Fig. 7C).

Determining PTEN signature from non-isogenic lines.

An alternative approach to determine PTEN-dependency signature would be to asses the amount of genes that behave differentially between PTEN* lines and WT non-isogenic cell lines using data from Marcotte et al (Marcotte et al, 2012). We compared the Z-normalized GARP distributions between the PTEN* lines and WT lines and collected genes that exhibit a significant shift to more severe scores in the PTEN* lines (p-value ≤ 0.05, using a one sided student t-test). This procedure resulted in 1147 genes. To assess the significance of this amount of genes, we repeated this procedure 500 times on randomized data (while shuffling the cell-line labels). On average, the random data yield ~750 genes with a significant behavior resulting in an empirical p-value of 0.05. An overview of this procedure can be found in Supplementary Figure 13A. While this procedure may capture genes independent of the GARP score (since their shift but not the actual essentiality is evaluated), the resulting genes have a small but significant tendency to be more essential in the PTEN* lines than in the WT lines. This is pictured in the box plot in Supplementary Figure 13B. We then assessed the overlap of the genes derived from this procedure to our top 5% hits of the DiE profile of PTEN using a Fisher exact test and found the two sets significantly overlap (102 instances, p-value 5e-10). We further investigated this enrichment using different fractions of our PTEN DiE profile. The most significant enrichment was discovered using a cut-off of ~750 top-genes, consistent with the applied cut-off of top 5% used throughout this study (Supplementary Figure 13C).

Precision-Recall Plots.

Precision-recall plots were computed to show that high scoring digenic pairs are more likely functionally related to each other than less significant ones. We defined a true positive prediction if the interacting gene shares one or more functional categories with the query

gene. These functional categories have been manually assigned based on the publicly available GO annotation and expert knowledge. We restricted this analysis to *BLM* and *MUS81* as query genes as they have very clear and distinct roles in DNA damage and nucleic acid metabolism. Recall was computed as TP/(FN+TP) and Precision as TP/(TP+FP) where TP is the amount of true positive (interacting and sharing an annotation term), FP the amount of false positives (interacting but not sharing annotation), and FN the amount of false negatives (not interacting but sharing annotation). Interacting genes have been defined as genes with a GARP score smaller (more aggravating) as a certain cut-off. By varying the cut-off from low (strongly aggravating) to high (more neutral) GARP scores, the plots were then generated. The ROCR package was used to generate the plots (Sing et al, 2005). We computed p-values for the observation of the area under the precision-recall curve (pAUC), which is higher when more signals are recovered on the left part. We empirically computed the p-value by randomizing the annotation 500 times while retaining the frequency of each annotation term as well as the frequency of term co-occurrence. None of the random run revealed a AUC as high as we found for the original data, resulting in p-values <1/500 in both cases.

Meta-analysis calculating the overlap of our study with other genomic datasets.

In order to assess the significance of the DiE genes with respect to other screens, we computed the frequency of the gene-sets in the DiE profile with gene from other indicated datasets. In case of enrichment in the DiE genes, we computed a p-value using a one-sided Fisher exact test (Supplementary Figure 12).

ACCESS TO SUPPLEMENTARY TABLES

All supplementary tables can be accessed using one of the following links.
http://moffatlab.ccbr.utoronto.ca/resources.php
http://moffatlab.ccbr.utoronto.ca/publications.html
http://kimLab1.ccbr.utoronto.ca/projects/cancer_essential/

SUPPLEMENTARY TABLE LEGENDS

Supplementary Table 1. Complete list of single hairpin (shARP) score across all the screens. The table lists the shARP score calculated based on the drop out signal across different time points. The TRC consortium number of each hairpin, Gene ID, and the score in independent screens are listed. To get the top hits from each screen, sort the column ascending.

Supplementary Table 2. Complete list of dGARP score across all screens. The table lists gene level dGARP score calculated as described in the methods section. The Gene ID, and the score in independent screens are listed. To get the top negative genetic interactions from each screen, sort the column ascending.

Supplementary Table 3. Gene expression profile for all HCT116 lines with different genetic backgrounds. Expression profile generated using the Affymetrix platform is given in this table. Note that the fold changes in expression compared to the parental lines are also indicated.

Supplementary Table 4. Validation data by secondary screen. List of hits tested in all the screens are given along with a reason for selection as illustrated in Supplementary Figure 2B. Individual sheets in this table represent the hits validated for each query. The confidence interval columns show three levels of confidence to confirm the hits as validated. In addition, the rank order of each hit in the primary screen, expression status and the fold change in expression are included for each gene.

Supplementary Table 5. Summary of the secondary validation screen. Sheet 1 lists the summary of the hits validated for each screen. Based on the hits validated with 80% confidence, around 200 negative genetic interactions are validated. Sheet 2 provides the raw data used to compute the hits for the secondary validation screen.

Supplementary Table 6. The complete database of cross-species ortholog mapping. This table lists ortholog relationships detected by the eggnog-based procedure to find the

most probable ortholog for each human gene. The data is mapped to Uniprot-identifier as used in the iRef-Web resource. For each gene and species (human yeast, fly, mouse, worm), the Uniprot gene name and gene names are listed. N/A indicates the absence of a common gene name for a gene (only Uniprot name available) and '-' denotes the absence of an ortholog.

Supplementary Table 7. List of genes represented in the high confidence differential essentiality network. Sheet1 of this table lists all the genes that are represented in Figure 2. Interaction pairs that were conserved in other species are also included with their published references. Sheet 2 of this table shows the statistical significance of the conserved interactions.

Supplementary Table 8. Supporting evidence for sub-networks using a cross-species analytical approach. This table lists the supporting evidences that connect the genes within each screen ($p<0.05$). These supporting evidences comprises of known genetic interactions and physical interactions that resemble the genetic interaction in human, or between any pair of orthologs in yeast, fly, worm, or mouse. The first sheet lists all of them together and the subsequent sheets lists separately for each screen for convenience. The last sheet provides information on the non-random structure of the resulting sub-networks as measured by the average clustering coefficient. P-values to find higher clustering-coefficients have been computed from 500 rounds of randomized data.

Supplementary Table 9. Genes that constitute PTEN-signature to determine PTEN genetic dependency. List of genes represented in Figure 7D. The alternative signatures derived from non-isogenic cell lines are listed in Sheet2. Sheet3 lists the intersection of signature genes from the *PTEN* signature derived from HCT116-$PTEN^{-/-}$ versus HCT116-$PTEN^{+/+}$ screening results and the *PTEN*/*PI3K* signature derived from non-isogenic cell lines .

Supplementary Table 10. Cell lines used in this study.

Supplementary Table 11. Sequence alignment of the gene pairs recovered as supporting interactions. Alignments of all genes establishing orthologs relationships that actually contributed to the main network evidences.

Supplementary Table 12. Classification of the cell lines based on PI3K/PTEN mutation status. The status column gives the classification of each cell line where “WT” denotes wild-type (not driven by PTEN loss or PI3K pathway), “PTEN” denotes cell lines mutated in PTEN,

“PIK3CA” denotes activating mutation in PI3K pathway, “PIK3CA dependency” denotes cell lines that have activated PI3K pathway but no detected mutations in the PI3K3CA gene. Mutation information has been derived from the CCLE database. Reference/Mutation lists the actual mutations or provides references to relevant publications describing the cell line.

SUPPLEMENTARY FIGURE LEGENDS

Supplementary Figure 1. Primary screening results. (A) Unsupervised clustering of correlation coefficients derived from the raw intensity values for each replicate and each time points across all cell lines screened. The replicates are represented as R1, R2 and R3 appended with the name of the screen. The time points are represented as Tx, where x denotes the day of harvest after infection. Red represents a high correlation and blue represents a lower correlation. (B-F) Density plot of raw intensity signal from different time points for each screen. The time points T0, T10 and T14 represent the time of harvesting in days relative to the first harvest (ie. T0). The distributions from each of the three replicates have a high degree of correlation ($R>0.90$) and are represented in the same color. (G-K) Examples of drop out trends for differentially essential genes between query and parental cell lines showing how different hairpins targeting the same gene behave in evolving screening populations. Each of the shRNAs are indicated by their respective TRC numbers in the legend and the trend lines represent the average of the three replicate screens. (L-P) Frequency distribution of dGARP score highlighting the top SSL hits with p-value <0.05 in black bars from each screen.

Supplementary Figure 2. Negative genetic interaction network of differentially essential genes and experimental setup for secondary validation screens. (A) Genetic interaction network for the genes identified in *PTTG1* (S), *MUS81* (M), *BLM* (B), *PTEN* (P) and *KRAS* (R) primary screens. Candidate hits that passed secondary validation are highlighted with blue nodes. The edges are color-coded based on cross-species evidence of genetic (solid lines) or protein (dotted lines) interactions. Triangular nodes indicate genes that have been annotated as having drugs that target them. (B) Flow chart representation of the selection process used to generate the list of hits to be validated using the HCS based co-culture assay. The table on the right shows the number of hits chosen in each category. (C-G) Growth patterns of color-coded parental and mutant cells in co culture assay. 100 replicates of mock-transfected cells were monitored for consistency for a period of seven days and the normalized values are plotted as percentage of red and green cells for each day from the multiple replicates. (H-L) Bar graph showing differential growth rates in the co-culture experiment after infection with corresponding *si*RNA as examples of validation. Fold change in the number of mutant cells compared to the parental cells after the depletion of corresponding gene products at 24hr interval is shown. (H-K) Time points showing specificity of the secondary validation as assessed for selected hits across multiple cell lines showing hits that were identified in both the primary screen and validated in a secondary screen (H

and I); a hit identified in primary screen but not validated in secondary screen (J) and a hit identified in one of the primary screen but validated in another screen (K).

Supplementary Figure 3. Orthology Mapping using MP-eggNOG. (A) Principle of the MP-eggNOG ortholog mapping approach. For each human gene, the orthologous group is split up into unique relationships between human genes and their most likely counterpart in the specified model organisms. For details please see the supplementary materials. (B) Distribution of mapping frequencies of one-to-many relationships from each species is represented. (C) Sensitivity of different ortholog detection methods including MP-eggNOG, the most probable ortholog based on eggNOG v2.0 mapping; Inparanoid; and BBH or the bi-directional best hit method using Smith-Waterman searches in eggNOG clusters. (D) Coverage of ortholog mappings with respect to human and different model organisms. "# in species" is the number of genes in the respective species that have a human ortholog assigned. "# in human" is the number of human genes which have an ortholog assigned in the respective model species. "Ratio" is the ratio of # in species to # in human. (E) Schematic representation highlighting conserved genetic interactions with KRAS using evidence from other model systems or independent screens from other groups in mammalian cells.

Supplementary Figure 4. Conserved negative genetic interaction between *PTTG1* and *ESPL1*. (A) Left: negative genetic interaction of two independent shRNAs targeting ESPL1 as assessed by proliferation assay. Middle: Western blot showing the levels of PTTG1 in total cell lysates in HCT116-*PTTG1*$^{+/+}$ and HCT116-*PTTG1*$^{-/-}$ cell lines. Right: anti-ESPL1 (Abcam) Western blot showing ESPL1 knockdown with two independent shRNAs targeting ESPL1. (B) Left: Percentage of HCT116-*PTTG1* $^{+/+}$ and HCT116-*PTTG1* $^{-/-}$ cells with a congression defect when transduced with constitutively expressed *sh*RNAs against LacZ or two independent shRNAs targeting *ESPL1*, measured in cells with spindle poles more than 6µm in distance as determined by NEDD1 staining. Middle: Percentage of HCT116-*PTTG1*$^{+/+}$ and HCT116-*PTTG1*$^{-/-}$ cells with lagging chromosomes when transduced with *sh*RNAs against LacZ or *ESPL1*, measured as cells were progressing through anaphase. Right: percentage of HCT116-PTTG1+/+ and HCT116-PTTG1-/- cells expressing a negative control shRNA targeting LacZ (shControl) or the same two independent shRNAs targeting ESPL1 as described above, in different phases of mitosis including prometaphase, metaphase and anaphase (n=3). ** represents $p<0.01$; *** represents $p<0.001$, calculated using chi-square test.

Supplementary Figure 5. Conserved negative genetic interaction between *TOPBP1* and *BLM*. (A) HCT116-*BLM*$^{+/+}$ and HCT116-*BLM*$^{-/-}$ cells were transfected with *si*RNAs that target *TOPBP1* (si*TOPBP1*) or a negative control (siCTRL) and then fixed and stained using

an antibody against γ-H2AX. (B) Percentage of HCT116-*BLM*$^{+/+}$ or HCT116-*BLM*$^{-/-}$ cells with greater than 5 foci by analyzing at least 100 cells following knockdown of TOPBP1 (n=3). **p-value <0.01 and *p-value=0.02 calculated using the Student's t-test. Scale bar is 12 μm. (C) Target validation of si*TOPBP1* by Western blot showing the levels of TOPBP1 in total cell lysates in the HCT116-*BLM*$^{+/+}$ cell line. (D) Model for the synthetic lethal relationship between *TOPBP1* and *BLM*. Loss of *BLM* results in replication fork stalling at aberrant DNA structures that are usually unwound by *BLM*. In the presence of *TOPBP1* this would result in *ATR* activation followed by cell cycle arrest, giving the cell time to resolve these inhibitory DNA structures. However, in the absence of *TOPBP1*, *ATR* signaling cannot be initiated resulting in non-replicated DNA entering mitosis causing excessive genome instability and subsequent lethality.

Supplementary Figure 6. Cross-species analyses of negative genetic interactions from the differential essentiality network with emerging functional modules. (A) Sub-network analysis of genes that exhibited negative genetic interaction with *BLM*$^{-/-}$ showing extensive interactions with one another as projected by cross-species analysis. A zoomed version of a subset of interactions more relevant to the function of *BLM* is shown. (B) Sub-network analysis of genes that that exhibited negative genetic interaction with *PTTG1*$^{-/-}$ showing extensive interactions with one another as projected by cross-species analysis. A zoomed version of a subset of interactions more relevant to the function of *PTTG1* is shown. (C) Sub-network analysis of genes that exhibited negative genetic interaction with *PTEN*$^{-/-}$ showing extensive interactions with one another as projected by cross-species analysis. A zoomed version of a subset of interactions more relevant to the function of *PTEN* is shown. (D) Sub-network analysis of genes that exhibited negative genetic interaction with *KRAS*^{G13D} showing extensive interactions with one another as projected by cross-species analysis. A zoomed version of a subset of interactions more relevant to the function of *KRAS* is shown. Note that we included *KRAS* itself as one of the nodes since it displayed several conserved interactions from other species. (E) Sub-network analysis of genes that exhibited negative genetic interactions with *MUS81*$^{-/-}$ showing extensive interactions with one another as projected by cross-species analysis. A zoomed version of a subset of interactions more relevant to the function of *MUS81* is shown.

Supplementary Figure 7: Novel negative genetic interaction between *PTTG1* and the uncharacterized gene *TTC31*. (A) Immunofluorescence of HCT116-*PTTG1*$^{+/+}$ cells depleted for TTC31 and stained to monitor the localization pattern of Aurora B. HCT116-*PTTG1*$^{+/+}$ cells infected with either a negative control shRNA (shLacZ) or shRNA targeting *TTC31* (shTTC31) were stained with antibodies against Aurora B as well as DAPI (n=3). Scale bar is 5 μm. (B

and C) Quantitation graph and representative images of HCT116-*PTTG1*$^{-/-}$ cells infected with sh*TTC31* or shRFP (ie. negative control) showing heterogeneity in nuclear morphology. Cells were stained for DNA (blue), Pericentrin (red) and NEDD1 (Green). Scale bar is 15 μm. (D) Representative images of *TTC31* knockdown in HeLa cells showing multiple centrosomes. Cells were stained for DNA (blue), Pericentrin (red) and Nedd1 (Green). Scale bar is 10 μm.

Supplementary Figure 8. Negative genetic interaction between *PTTG1* and *ZC3H13.* (A) Western blot analysis showing the levels of ZC3H13 and PTTG1 across multiple pancreatic cancer lines. (B) Homologous human genes of yeast *PDS1* as reported by SIMAP (Rattei et al, 2010). Instances of the same gene are summarized by color code. (C) Smith-Waterman sequence alignment of *PDS1* with *ZC3H13*. Alignment parameters are given on top of the alignment. The used substitution matrix is Blosum62. (D) Simplified model showing the role of ZC3H13 in chromosome segregation.

Supplementary Figure 9: Negative genetic interaction between *PTTG1* and *DHFR.* (A) Growth curve of wildtype (left) or *pds1-128* (middle) yeast cells expressing empty control vector or wild-type yeast cells over-expressing *PDS1* (right) grown in the presence of methotrexate at semi-permissive temperature (35°C)(OE denotes over expressing *PDS1*). (B) Confirmation of DHFR knockdown using two independent shRNAs targeting *DHFR* by western blot and qPCR in HCT116-*PTTG1*$^{+/+}$ cells. Representative images from Hela cells and quantitation graph of DHFR knockdown cells showing bi- and multi-nucleated cells. Quantitation was done in both HCT116 cells (*PTTG1*$^{+/+}$ and *PTTG1*$^{-/-}$) and HeLa cells and represented in the graph. Cells were stained for DNA (blue), Pericentrin (red) and Nedd1 (Green). Scale bar is 10 μm. (C) Correlation plot of *PTTG1* and *DHFR* expression across 300 different cancer cell lines (see Methods for details of analysis).

Supplementary Figure 10: Negative genetic interaction between *PTEN* and either *RASSF2* or *ENO2.* (A) Relative transcript levels of *RASSF2* in HCT116-*PTEN*$^{+/+}$ and HCT116-*PTEN*$^{-/-}$ cells. (B) A model for synthetic lethal relationship between *PTEN* and *RASSF2*. (C) Western blot showing the level of PTEN and phospho-*AKT* in HCT116-*PTEN*$^{+/+}$ and HCT116-*PTEN*$^{-/-}$ cells and relative transcript levels of *RASSF2* in *RASSF2* knockdown cells. (D) Relative transcript levels of *ENO2* in HCT116-*PTEN*$^{+/+}$ and HCT116-*PTEN*$^{-/-}$ cells. (E) Western blot analysis of protein expression levels in HCT116 cells after knocking down *ENO2* with two independent hairpins. shLacZ was used as a negative control. (F) Bar graph of protein levels of phospho-*AKT*, Pan-*AKT* and *ENO2* in HCT116-*PTEN*$^{+/+}$ and HCT116-*PTEN*$^{-/-}$ cell lines. Protein levels were normalized based on actin levels. *p-value < 0.05 and

**p-value < 0.01 calculated by Student t-test. (G) Model for the feed back mechanism between the *PTEN* and the Glycolytic pathway in an *ENO2* dependent fashion.

Supplementary Figure 11. Negative genetic interaction between *KRAS^{G13D}* and *HKDC1.* (A) Relative transcript levels of *HKDC1* in HCT116-*KRAS*$^{+/-}$ and HCT116-*KRAS*$^{+/G13D}$ cells as evaluated by quantitative PCR. (B) Phylogenetic tree showing HKDC1 in relation to the rest of the hexokinase family members. Average distance trees calculated using the UPGMA method for HKDC1 orthologs and paralogs using the sum of BLOSUM 62 scores for similarity calculation. Numbers on tree branches represent distances as reported by the Unweighted Pair Group Method with Arithmetic Mean (UPGMA) method. Phylogenetic tree was calculated using Jalview,version 2.6.1. The bottom panel shows the hexokinase domain positions as derived from Pfam. (C) Sorted heat map of normalized zGARP score across 78 screens in 4 different cancer types. The scale indicates the level of essentiality across these screens. Relative positions of *KRAS*, *PLK1*, *HKDC1* and other hexokinases are highlighted. (D) Proliferation assay from three independent replicates after infecting the HCT116-*KRAS*$^{+/-}$ and HCT116-*KRAS*$^{+/G13D}$ cells with two independent *sh*RNAs targeting *HKDC1* are shown. (E) Relative transcript levels in *HKDC1* knockdown cells using two independent shRNAs targeting *HKDC1*. (F) *RAS* signaling dependence score versus *HKDC1* expression in EXPO datasets (see Methods for details). Vertical line separates high and low *RAS* signaling dependence whereas the horizontal line separates *HKDC1* "expressed" and "expression uncertain". p-value $< 2.2 \times 10^{-16}$ calculated using Fisher Exact Test. (G) Distribution of RAS signaling dependence scores and *HKDC1* expression values in CCLE and EXPO studies. Overlaid are fitted Gaussian Mixture Models used to classify RAS scores as high/low, and *HKDC1* expression as expressed/uncertain. Dotted lines indicate thresholds derived from these models.

Supplementary Figure 12. Meta-analysis of comparable screens from curated literature sources . (A) Meta-analysis of our primary screening data generated from the *PTTG1*$^{-/-}$ screen and previously published mitotic screens represented as a binary heat map. The names of the screens are number coded. 1represents rank in the primary screen; 2 represents fold change in expression in *PTTG1*$^{-/-}$ cells compared to *PTTG1*$^{+/+}$ cells; 3, 4, 5, 6 and 7 are references from (Andersen et al, 2003; Hutchins et al, 2010; Kittler et al, 2004; Moffat et al, 2006; Neumann et al, 2010) (B) Meta-analysis of the primary and secondary data generated from the *MUS81*$^{-/-}$, *BLM*$^{-/-}$ and *KRAS*$^{G13D/+}$ screens and previously published screens. The previously published screens are represented by the Pubmed IDs. A parallel screen done in PSNG13 cell line that is deficient in *BLM* is also included to compute the overlap.. (C-D) Bar graph measuring the amount nuclear area covered by foci formation for

different *si*RNAs selected after confirming the SSL interaction with either *BLM*$^{-/-}$ or *MUS81*$^{-/-}$. (E-F) Precision recall plots of *BLM* and *MUS81*. X-axis represents the Recall computed as TP/(TP+FN) and y-axis represents the Precision computed as TP/(TP+FP). The color code encodes the absolute value of the GARP-score, sorted from left to right with the most aggravating interactions on the left. For comparison, a run with randomized annotation is plotted in black. (G) Overlaps among *PTEN*, *BLM* and *MUS81* screens are represented as a Venn diagram. (H) Phylogenetic tree showing *TTC31* and *STI1* are closely positioned within the same dendogram. Average distance trees calculated using the Unweighted Pair Group Method with Arithmetic Mean (UPGMA) method for *TTC31* orthologs and paralogs using the sum of BLOSUM 62 scores for similarity calculation. Numbers on tree branches represent distances as reported by the UPGMA method. Phylogenetic tree was calculated using Jalview, version 2.6.1.

Supplementary Figure 13. Alternative PTEN signature. (A) Schematic overview to derive genes that exhibit a significant shift to lower GARP scores in PTEN* vs WT lines in non-isogenic lines. (B) Box plot of GARP distributions of the genes constituting the PTEN signature derived from non-isogenic lines. (C) Exploration of the optimal DiE profile size to recover the PTEN signature. Y-axis represents the fraction of overlap between PTEN DiE profile and the PTEN signature derived from non-isogenic lines. X-axis represents the staggered size of the gene sets based on dGARP. Note that the fraction of overlap peaks with a DiE profile size of 750 as indicated on the x-axis. (D) The Jaccard index distribution for both the PTEN-dependent and PTEN-independent sets as negative controls from dGARP values from MUS81, KRAS, PTTG1 and BLM are plotted as box-plot. (E) Jaccard Index for negative controls showing poor classification of those lines that are dependent on PTEN/PI3K pathway.

Supplementary Figure 14. Expression Analyses. (A) Determination of the threshold value for mRNA expression levels in the cell lines studied using genome-wide microarray expression profiling. The plot shows the cumulative percentage of log_2-expression on Affymetrix Gene 1.0ST arrays for expressed genes (ie. Refseqs), Affymetrix controls including expressed and non-expressed genes, as well as negative controls (e.g. intron sequences, miRNAs) for a given sample.. (B) Venn diagram showing the number of expressed genes that overlap with the pooled shRNA lentiviral library used in this study for each of the six isogenic cell lines screened. (C) Summary statistics from expression profiling.

SUPPLEMENTAL REFERENCES

Andersen JS, Wilkinson CJ, Mayor T, Mortensen P, Nigg EA, Mann M (2003) Proteomic characterization of the human centrosome by protein correlation profiling. *Nature* **426:** 570-574

Barabasi AL, Oltvai ZN (2004) Network biology: understanding the cell's functional organization. *Nat Rev Genet* **5:** 101-113

Barretina J, Caponigro G, Stransky N, Venkatesan K, Margolin AA, Kim S, Wilson CJ, Lehar J, Kryukov GV, Sonkin D, Reddy A, Liu M, Murray L, Berger MF, Monahan JE, Morais P, Meltzer J, Korejwa A, Jane-Valbuena J, Mapa FA et al (2012) The Cancer Cell Line Encyclopedia enables predictive modelling of anticancer drug sensitivity. *Nature* **483:** 603-607

Blakely K, Ketela T, Moffat J (2011) Pooled lentiviral shRNA screening for functional genomics in mammalian cells. *Methods Mol Biol* **781:** 161-182

Byrne AB, Weirauch MT, Wong V, Koeva M, Dixon SJ, Stuart JM, Roy PJ (2007) A global analysis of genetic interactions in Caenorhabditis elegans. *J Biol* **6:** 8

Campeau E, Ruhl VE, Rodier F, Smith CL, Rahmberg BL, Fuss JO, Campisi J, Yaswen P, Cooper PK, Kaufman PD (2009) A versatile viral system for expression and depletion of proteins in mammalian cells. *PLoS One* **4:** e6529

Chen F, Mackey AJ, Stoeckert CJ, Jr., Roos DS (2006) OrthoMCL-DB: querying a comprehensive multi-species collection of ortholog groups. *Nucleic Acids Res* **34:** D363-368

Cheng JQ, Ruggeri B, Klein WM, Sonoda G, Altomare DA, Watson DK, Testa JR (1996) Amplification of AKT2 in human pancreatic cells and inhibition of AKT2 expression and tumorigenicity by antisense RNA. *Proc Natl Acad Sci U S A* **93:** 3636-3641

Cline MS, Smoot M, Cerami E, Kuchinsky A, Landys N, Workman C, Christmas R, Avila-Campilo I, Creech M, Gross B, Hanspers K, Isserlin R, Kelley R, Killcoyne S, Lotia S, Maere S, Morris J, Ono K, Pavlovic V, Pico AR et al (2007) Integration of biological networks and gene expression data using Cytoscape. *Nature protocols* **2:** 2366-2382

Costanzo M, Baryshnikova A, Bellay J, Kim Y, Spear ED, Sevier CS, Ding H, Koh JL, Toufighi K, Mostafavi S, Prinz J, St Onge RP, VanderSluis B, Makhnevych T, Vizeacoumar FJ, Alizadeh S, Bahr S, Brost RL, Chen Y, Cokol M et al (2010) The genetic landscape of a cell. *Science* **327:** 425-431

Dimmer EC, Huntley RP, Alam-Faruque Y, Sawford T, O'Donovan C, Martin MJ, Bely B, Browne P, Mun Chan W, Eberhardt R, Gardner M, Laiho K, Legge D, Magrane M, Pichler K, Poggioli D, Sehra H, Auchincloss A, Axelsen K, Blatter MC et al (2012) The UniProt-GO Annotation database in 2011. *Nucleic Acids Res* **40:** D565-570

Efron B, Tibshirani R (1993) *An introduction to the bootstrap*, New York: Chapman & Hall.

Halilovic E, She QB, Ye Q, Pagliarini R, Sellers WR, Solit DB, Rosen N (2010) PIK3CA mutation uncouples tumor growth and cyclin D1 regulation from MEK/ERK and mutant KRAS signaling. *Cancer Res* **70:** 6804-6814

Heinicke S, Livstone MS, Lu C, Oughtred R, Kang F, Angiuoli SV, White O, Botstein D, Dolinski K (2007) The Princeton Protein Orthology Database (P-POD): a comparative genomics analysis tool for biologists. *PLoS One* **2:** e766

Hutchins JR, Toyoda Y, Hegemann B, Poser I, Heriche JK, Sykora MM, Augsburg M, Hudecz O, Buschhorn BA, Bulkescher J, Conrad C, Comartin D, Schleiffer A, Sarov M, Pozniakovsky A, Slabicki MM, Schloissnig S, Steinmacher I, Leuschner M, Ssykor A et al (2010) Systematic analysis of human protein complexes identifies chromosome segregation proteins. *Science* **328:** 593-599

Jaccard P (1901) Étude comparative de la distribution florale dans une portion des Alpes et des Jura. *Bulletin de la Société Vaudoise des Sciences Naturelles* **37:** 547–579

Ketela T, Heisler LE, Brown KR, Ammar R, Kasimer D, Surendra A, Ericson E, Blakely K, Karamboulas D, Smith AM, Durbic T, Arnoldo A, Cheung-Ong K, Koh JL, Gopal S, Cowley GS, Yang X, Grenier JK, Giaever G, Root DE et al (2011) A comprehensive platform for highly multiplexed mammalian functional genetic screens. *BMC Genomics* **12:** 213

Kittler R, Putz G, Pelletier L, Poser I, Heninger AK, Drechsel D, Fischer S, Konstantinova I, Habermann B, Grabner H, Yaspo ML, Himmelbauer H, Korn B, Neugebauer K, Pisabarro MT, Buchholz F (2004) An endoribonuclease-prepared siRNA screen in human cells identifies genes essential for cell division. *Nature* **432:** 1036-1040

Koh JL, Ding H, Costanzo M, Baryshnikova A, Toufighi K, Bader GD, Myers CL, Andrews BJ, Boone C (2010) DRYGIN: a database of quantitative genetic interaction networks in yeast. *Nucleic Acids Res* **38:** D502-507

Lawo S, Bashkurov M, Mullin M, Ferreria MG, Kittler R, Habermann B, Tagliaferro A, Poser I, Hutchins JR, Hegemann B, Pinchev D, Buchholz F, Peters JM, Hyman AA, Gingras AC, Pelletier L (2009) HAUS, the 8-subunit human Augmin complex, regulates centrosome and spindle integrity. *Curr Biol* **19:** 816-826

Loboda A, Nebozhyn M, Cheng C, Vessey R, Huang P, Dai H, Watters JW (2009) Biomarker discovery: identification of a growth factor gene signature. *Clinical pharmacology and therapeutics* **86:** 92-96

Loboda A, Nebozhyn M, Klinghoffer R, Frazier J, Chastain M, Arthur W, Roberts B, Zhang T, Chenard M, Haines B, Andersen J, Nagashima K, Paweletz C, Lynch B, Feldman I, Dai H, Huang P, Watters J (2010) A gene expression signature of RAS pathway dependence

predicts response to PI3K and RAS pathway inhibitors and expands the population of RAS pathway activated tumors. *BMC medical genomics* **3:** 26

Luo J, Emanuele MJ, Li D, Creighton CJ, Schlabach MR, Westbrook TF, Wong KK, Elledge SJ (2009) A genome-wide RNAi screen identifies multiple synthetic lethal interactions with the Ras oncogene. *Cell* **137:** 835-848

Mak AB, Ni Z, Hewel JA, Chen GI, Zhong G, Karamboulas K, Blakely K, Smiley S, Marcon E, Roudeva D, Li J, Olsen JB, Wan C, Punna T, Isserlin R, Chetyrkin S, Gingras AC, Emili A, Greenblatt J, Moffat J (2010) A lentiviral functional proteomics approach identifies chromatin remodeling complexes important for the induction of pluripotency. *Mol Cell Proteomics* **9:** 811-823

Marcotte R, Brown KR, Suarez F, Sayad A, Karamboulas K, Krzyzanowski PM, Sircoulomb F, Medrano M, Fedyshyn Y, Koh JL, van Dyk D, Fedyshyn B, Luhova M, Brito GC, Vizeacoumar FJ, Vizeacoumar FS, Datti A, Kasimer D, Buzina A, Mero P et al (2012) Essential gene profiles in breast, pancreatic, and ovarian cancer cells. *Cancer Discov* **2:** 172-189

Moffat J, Grueneberg DA, Yang X, Kim SY, Kloepfer AM, Hinkle G, Piqani B, Eisenhaure TM, Luo B, Grenier JK, Carpenter AE, Foo SY, Stewart SA, Stockwell BR, Hacohen N, Hahn WC, Lander ES, Sabatini DM, Root DE (2006) A lentiviral RNAi library for human and mouse genes applied to an arrayed viral high-content screen. *Cell* **124:** 1283-1298

Moffat J, Sabatini DM (2006) Building mammalian signalling pathways with RNAi screens. *Nat Rev Mol Cell Biol* **7:** 177-187

Muller J, Szklarczyk D, Julien P, Letunic I, Roth A, Kuhn M, Powell S, von Mering C, Doerks T, Jensen LJ, Bork P (2010) eggNOG v2.0: extending the evolutionary genealogy of genes with enhanced non-supervised orthologous groups, species and functional annotations. *Nucleic Acids Res* **38:** D190-195

Neumann B, Walter T, Heriche JK, Bulkescher J, Erfle H, Conrad C, Rogers P, Poser I, Held M, Liebel U, Cetin C, Sieckmann F, Pau G, Kabbe R, Wunsche A, Satagopam V, Schmitz MH, Chapuis C, Gerlich DW, Schneider R et al (2010) Phenotypic profiling of the human genome by time-lapse microscopy reveals cell division genes. *Nature* **464:** 721-727

Ostlund G, Schmitt T, Forslund K, Kostler T, Messina DN, Roopra S, Frings O, Sonnhammer EL (2010) InParanoid 7: new algorithms and tools for eukaryotic orthology analysis. *Nucleic Acids Res* **38:** D196-203

Rattei T, Tischler P, Gotz S, Jehl MA, Hoser J, Arnold R, Conesa A, Mewes HW (2010) SIMAP--a comprehensive database of pre-calculated protein sequence similarities, domains, annotations and clusters. *Nucleic Acids Res* **38:** D223-226

Root DE, Hacohen N, Hahn WC, Lander ES, Sabatini DM (2006) Genome-scale loss-of-function screening with a lentiviral RNAi library. *Nat Methods* **3:** 715-719

Safran M, Dalah I, Alexander J, Rosen N, Iny Stein T, Shmoish M, Nativ N, Bahir I, Doniger T, Krug H, Sirota-Madi A, Olender T, Golan Y, Stelzer G, Harel A, Lancet D (2010) GeneCards Version 3: the human gene integrator. *Database (Oxford)* **2010:** baq020

Sing T, Sander O, Beerenwinkel N, Lengauer T (2005) ROCR: visualizing classifier performance in R. *Bioinformatics* **21:** 3940-3941

Stark C, Breitkreutz BJ, Chatr-Aryamontri A, Boucher L, Oughtred R, Livstone MS, Nixon J, Van Auken K, Wang X, Shi X, Reguly T, Rust JM, Winter A, Dolinski K, Tyers M (2011) The BioGRID Interaction Database: 2011 update. *Nucleic Acids Res* **39:** D698-704

Tanaka K, Matsumoto K, Toh EA (1989) IRA1, an inhibitory regulator of the RAS-cyclic AMP pathway in Saccharomyces cerevisiae. *Mol Cell Biol* **9:** 757-768

Tanaka K, Nakafuku M, Satoh T, Marshall MS, Gibbs JB, Matsumoto K, Kaziro Y, Toh-e A (1990a) S. cerevisiae genes IRA1 and IRA2 encode proteins that may be functionally equivalent to mammalian ras GTPase activating protein. *Cell* **60:** 803-807

Tanaka K, Nakafuku M, Tamanoi F, Kaziro Y, Matsumoto K, Toh-e A (1990b) IRA2, a second gene of Saccharomyces cerevisiae that encodes a protein with a domain homologous to mammalian ras GTPase-activating protein. *Mol Cell Biol* **10:** 4303-4313

Tarailo M, Tarailo S, Rose AM (2007) Synthetic lethal interactions identify phenotypic "interologs" of the spindle assembly checkpoint components. *Genetics* **177:** 2525-2530

Tischler J, Lehner B, Fraser AG (2008) Evolutionary plasticity of genetic interaction networks. *Nat Genet* **40:** 390-391

Torrance CJ, Agrawal V, Vogelstein B, Kinzler KW (2001) Use of isogenic human cancer cells for high-throughput screening and drug discovery. *Nat Biotechnol* **19:** 940-945

Turner B, Razick S, Turinsky AL, Vlasblom J, Crowdy EK, Cho E, Morrison K, Donaldson IM, Wodak SJ (2010) iRefWeb: interactive analysis of consolidated protein interaction data and their supporting evidence. *Database (Oxford)* **2010:** baq023

UniProt C (2012) Reorganizing the protein space at the Universal Protein Resource (UniProt). *Nucleic Acids Res* **40:** D71-75

Yang X, Boehm JS, Yang X, Salehi-Ashtiani K, Hao T, Shen Y, Lubonja R, Thomas SR, Alkan O, Bhimdi T, Green TM, Johannessen CM, Silver SJ, Nguyen C, Murray RR, Hieronymus H, Balcha D, Fan C, Lin C, Ghamsari L et al (2011) A public genome-scale lentiviral expression library of human ORFs. *Nat Methods* **8:** 659-661

A

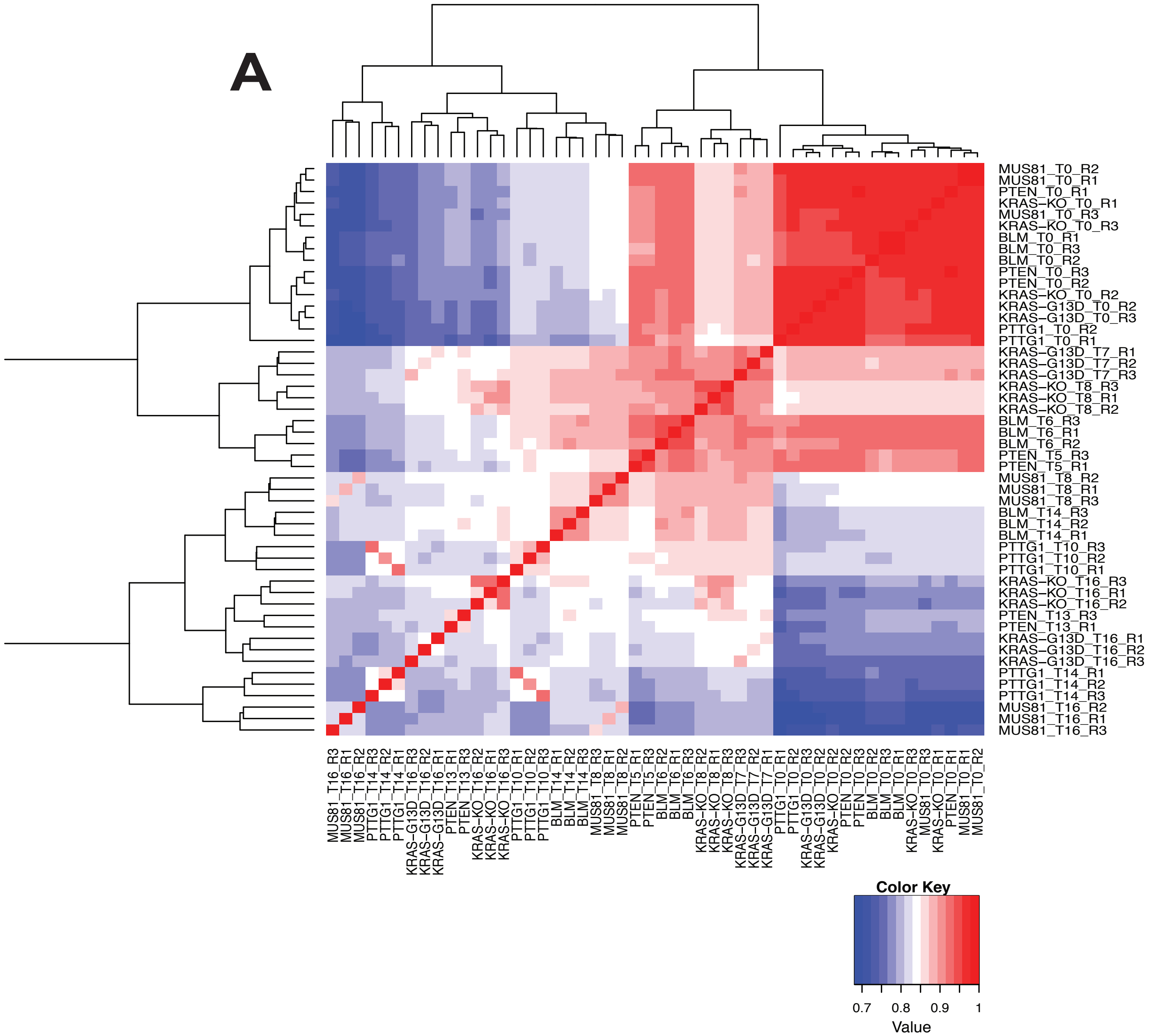

Supplementary Fig. 1

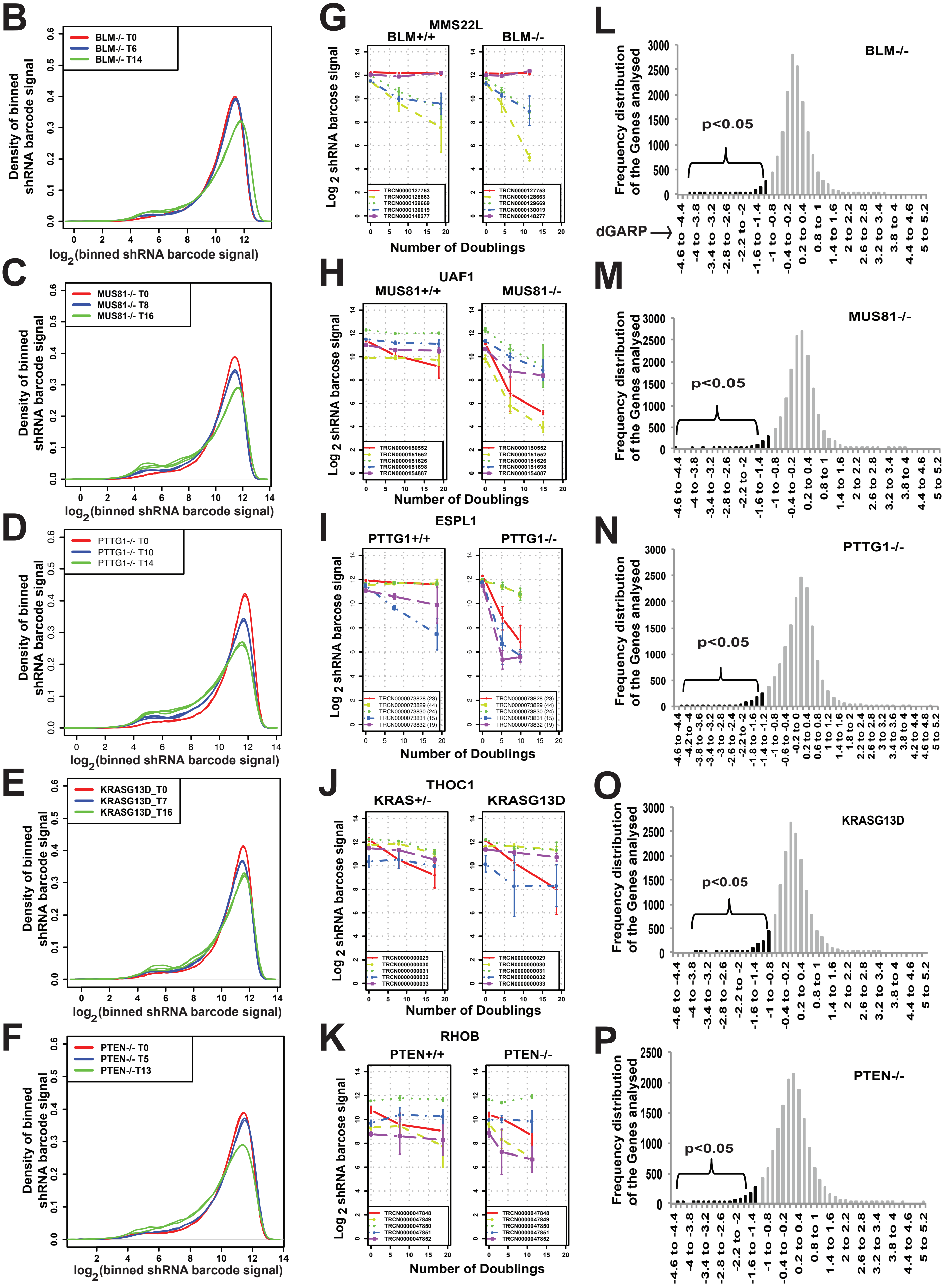

Supplementary Fig. 1cont'd

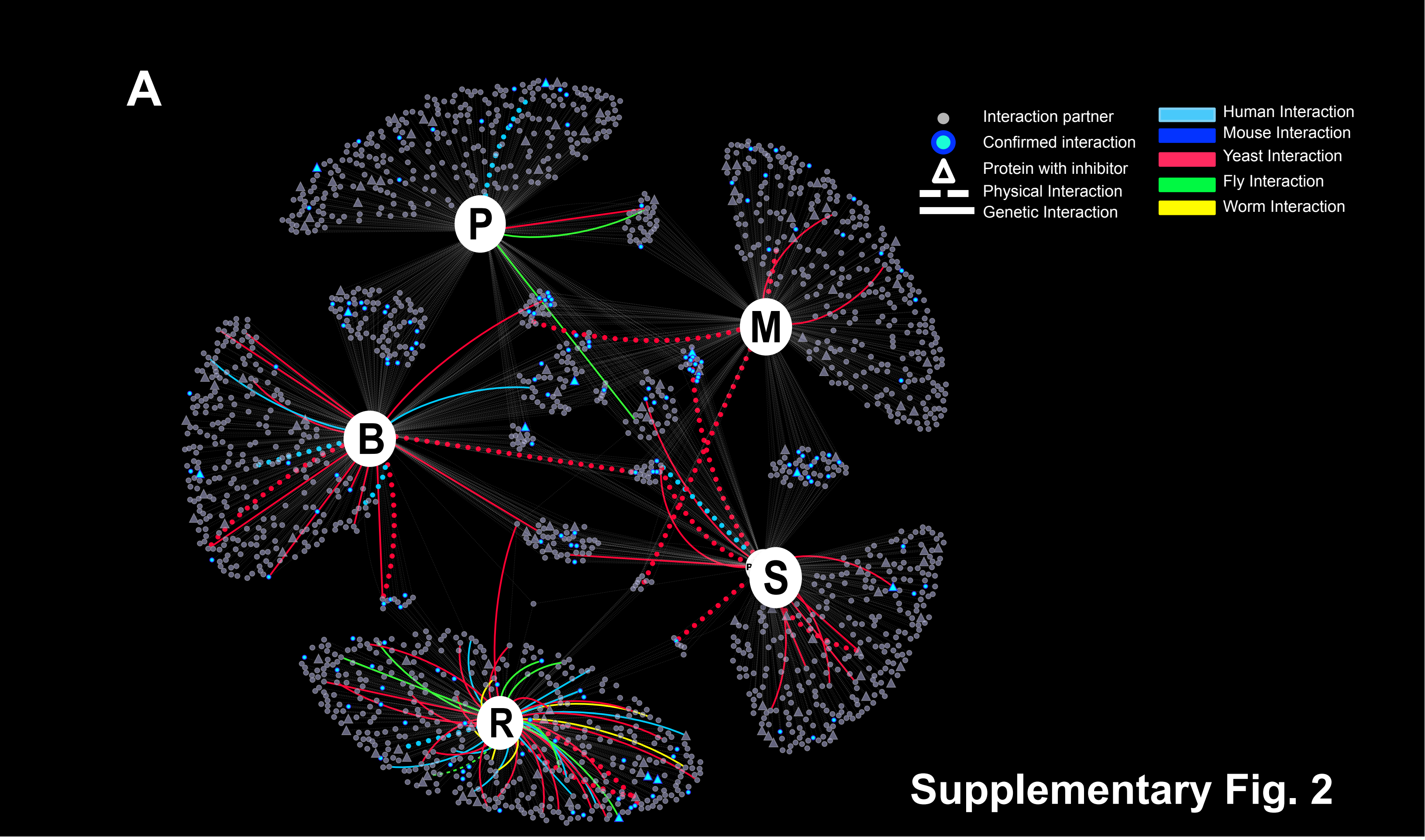

Supplementary Fig. 2

B

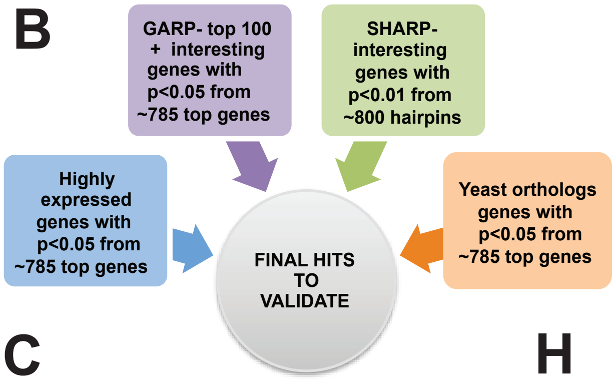

	Expression-based hits	GARP-based hits	shARP-based hits	Yeast orthology-based hits	Selected for validation	Actually tested (filtered for expression)
PTTG1	5	218	33	18	274	201
BLM	3	186	11	30	230	173
MUS81	2	233	15	6	256	205
PTEN	4	162	0	36	202	154
KRAS	2	111	0	30	143	93

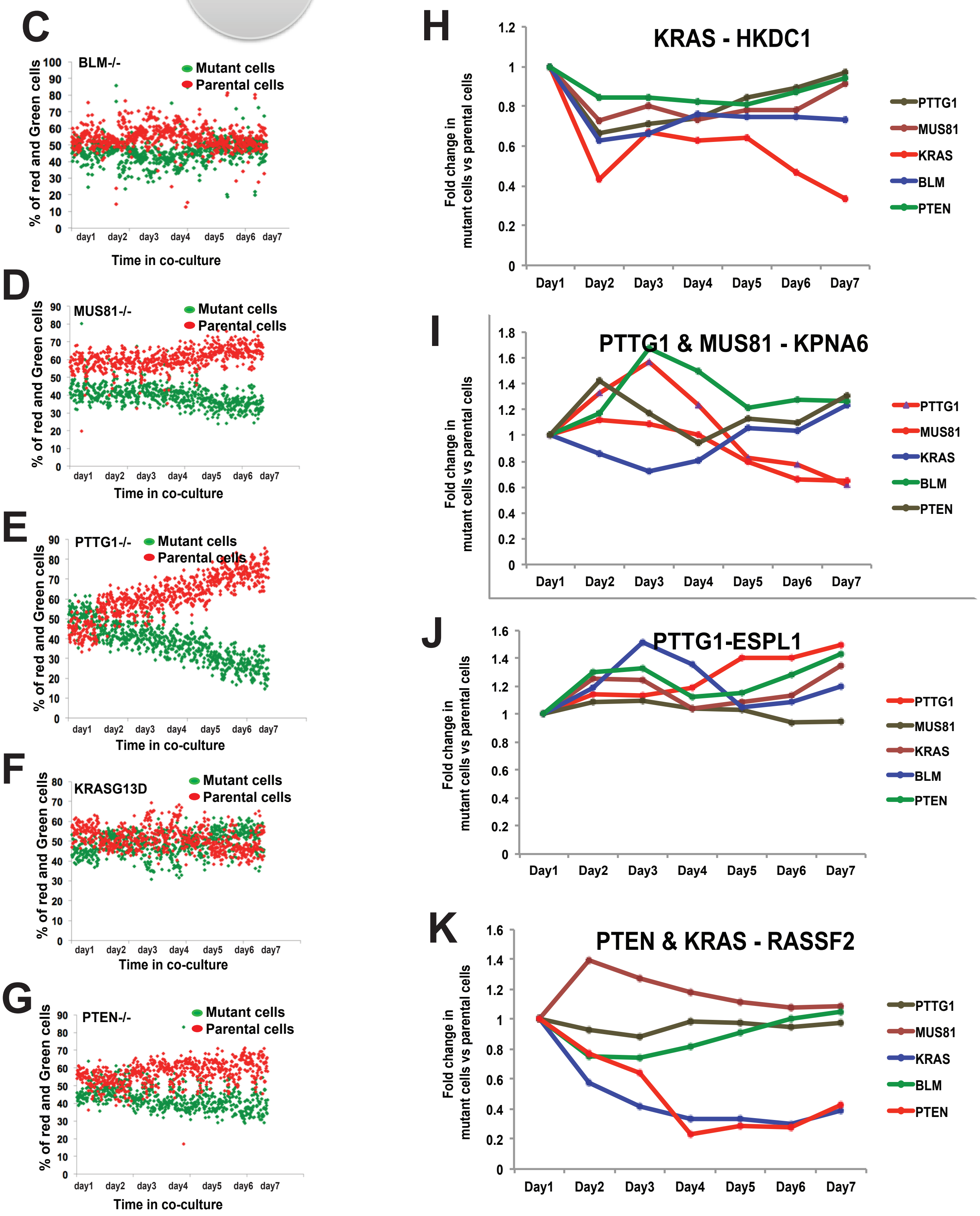

Supplementary Fig. 2cont'd

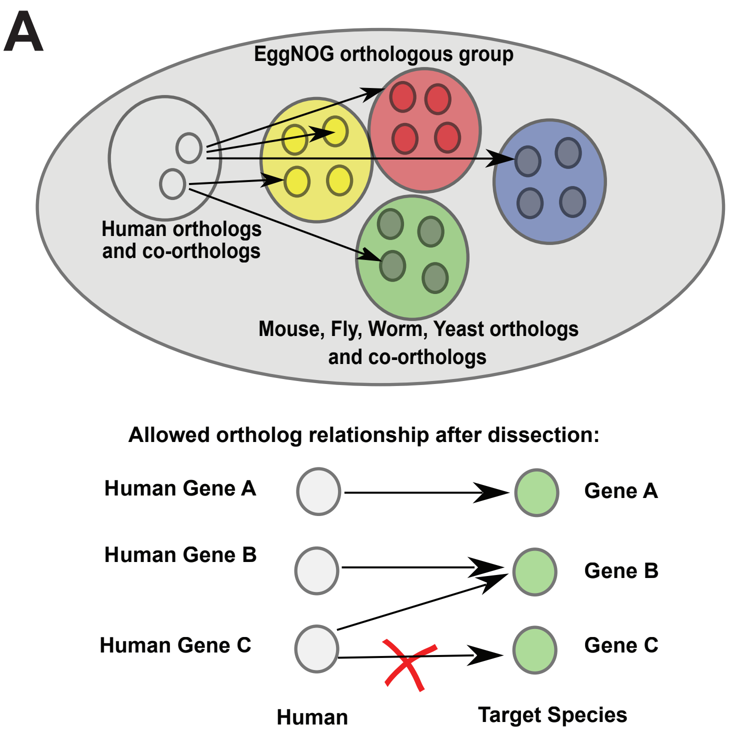

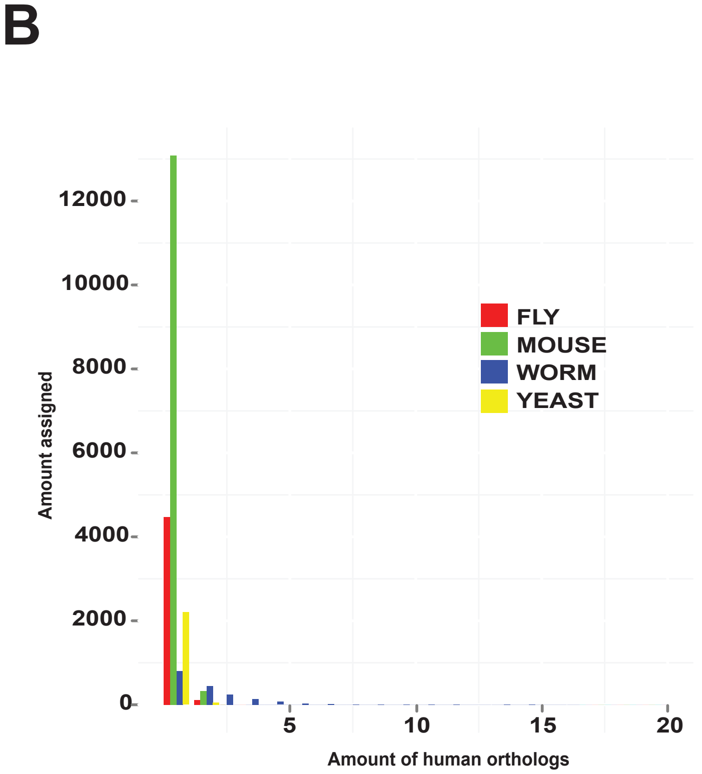

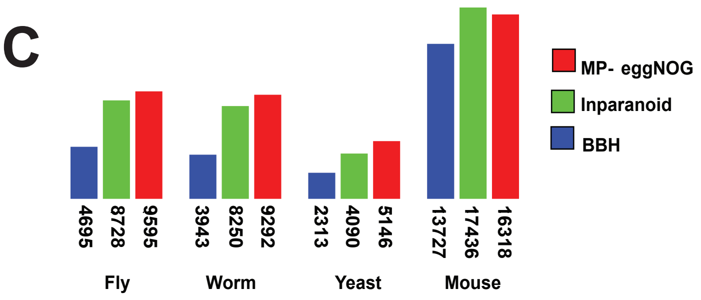

D

	Mouse	Fly	Worm	Yeast
# in species	14215	5180	4558	2440
# in human	16318	9595	9292	5146
ratio	1:1.1	1:1.8	1:2.0	1:2.1

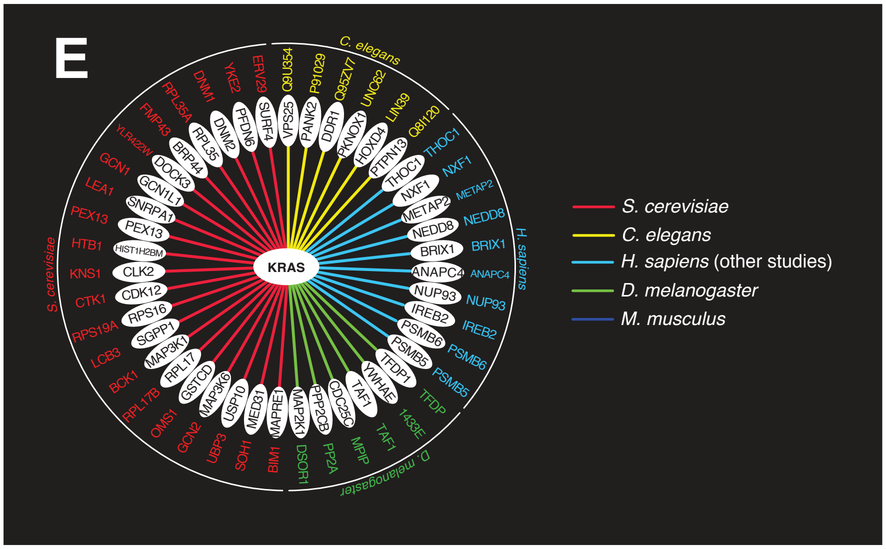

Supplementary Fig. 3

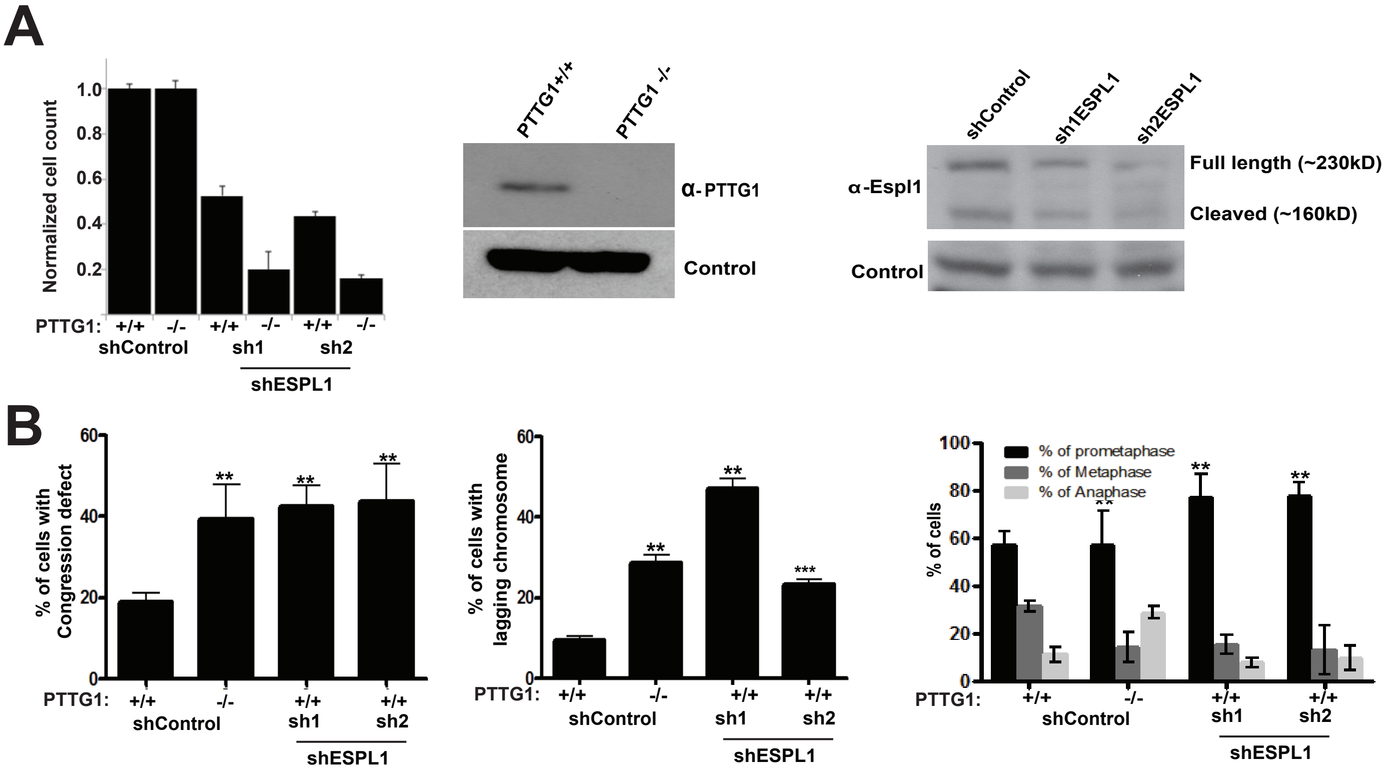

Supplementary Fig. 4

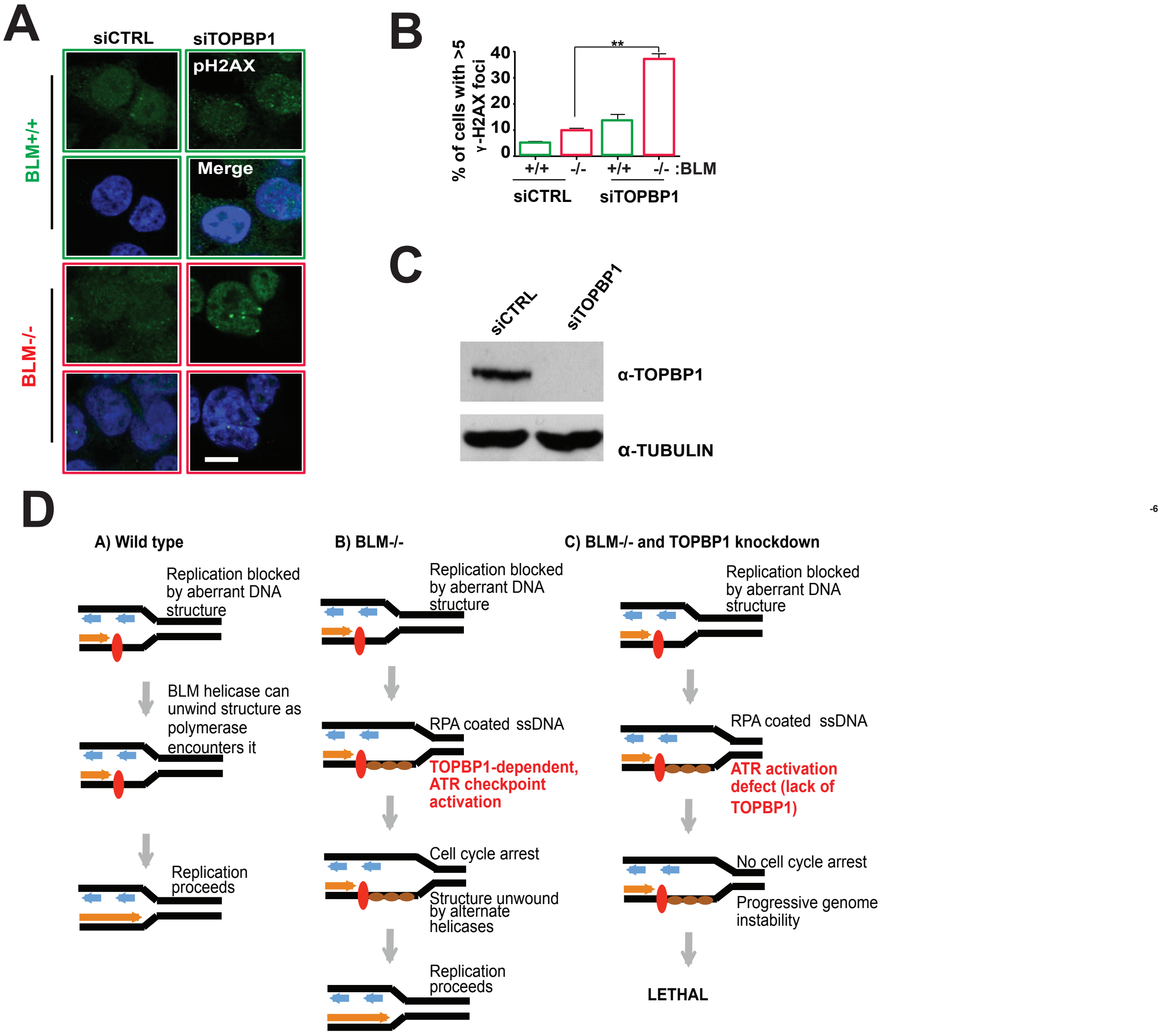

Supplementary Fig. 5

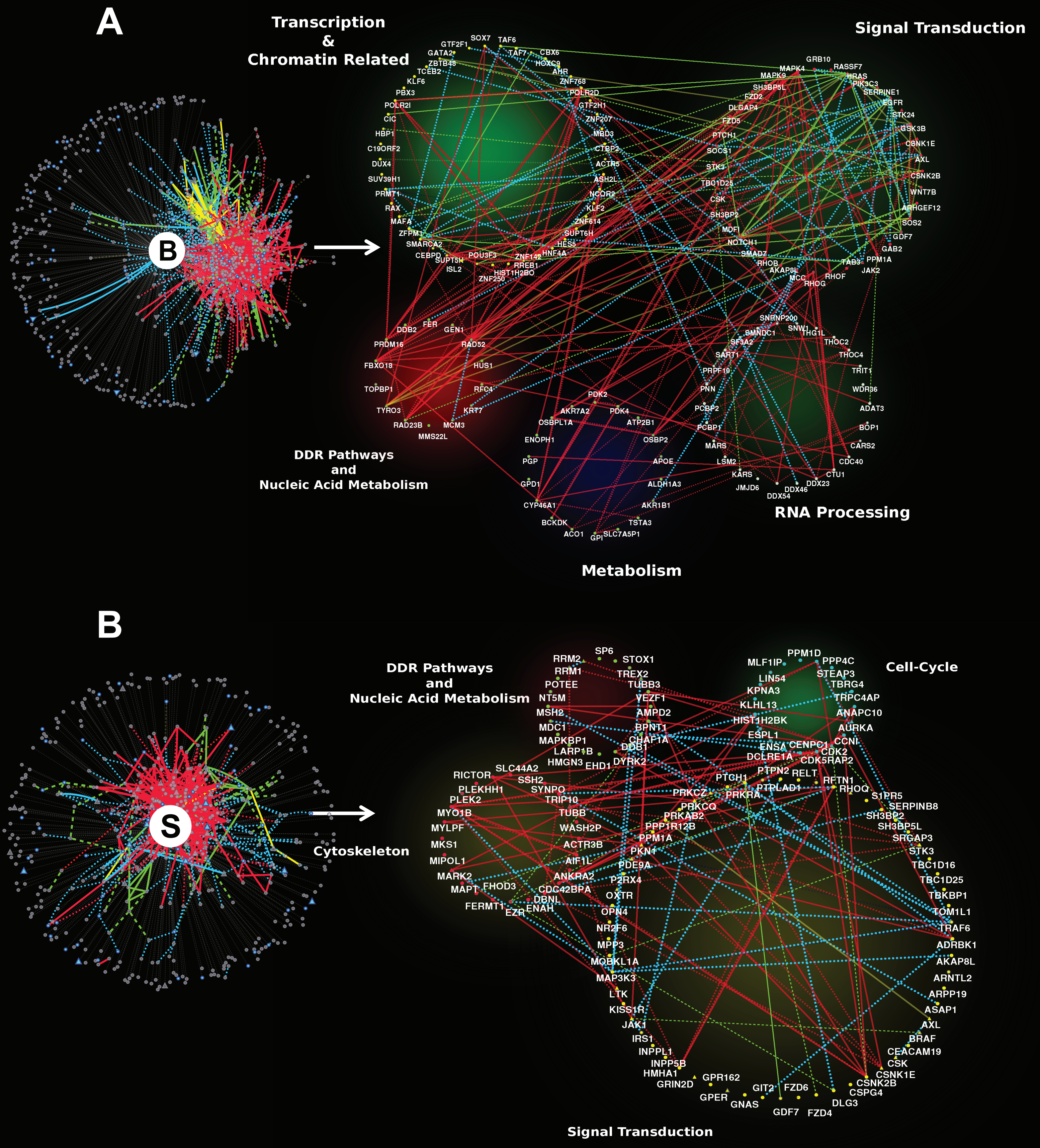

Supplementary Fig. 6

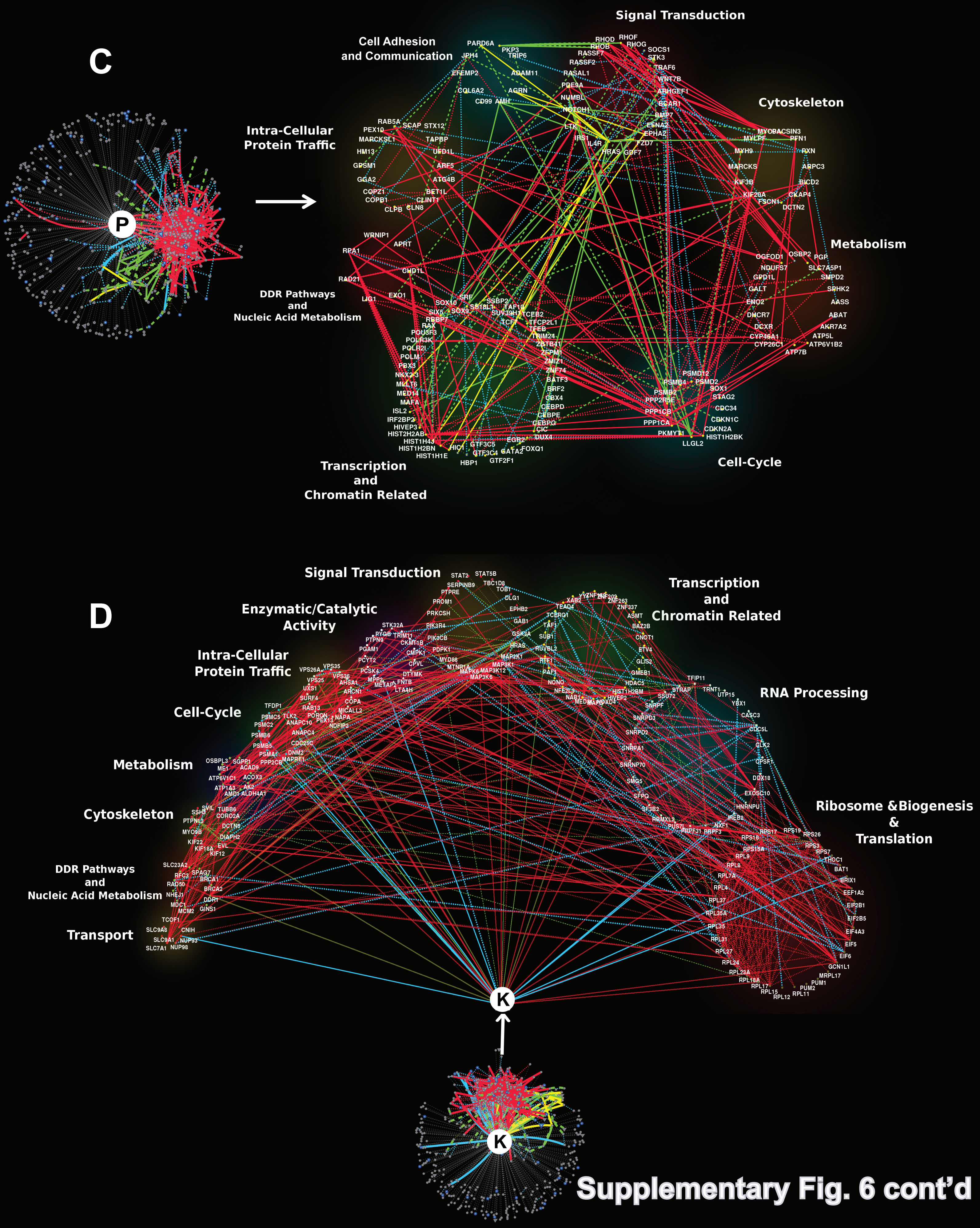

Supplementary Fig. 6 cont'd

E

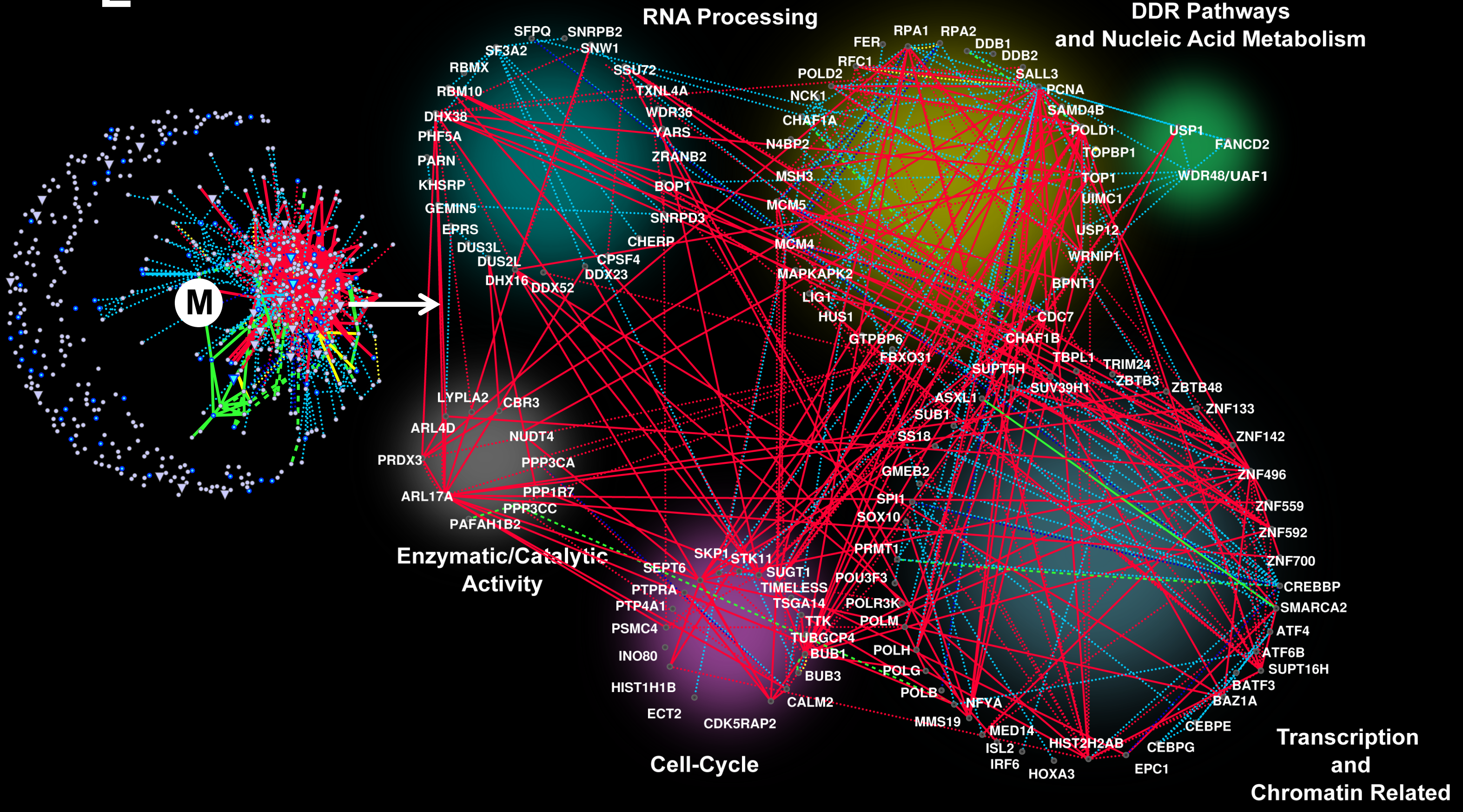

Supplementary Fig. 6 cont'd

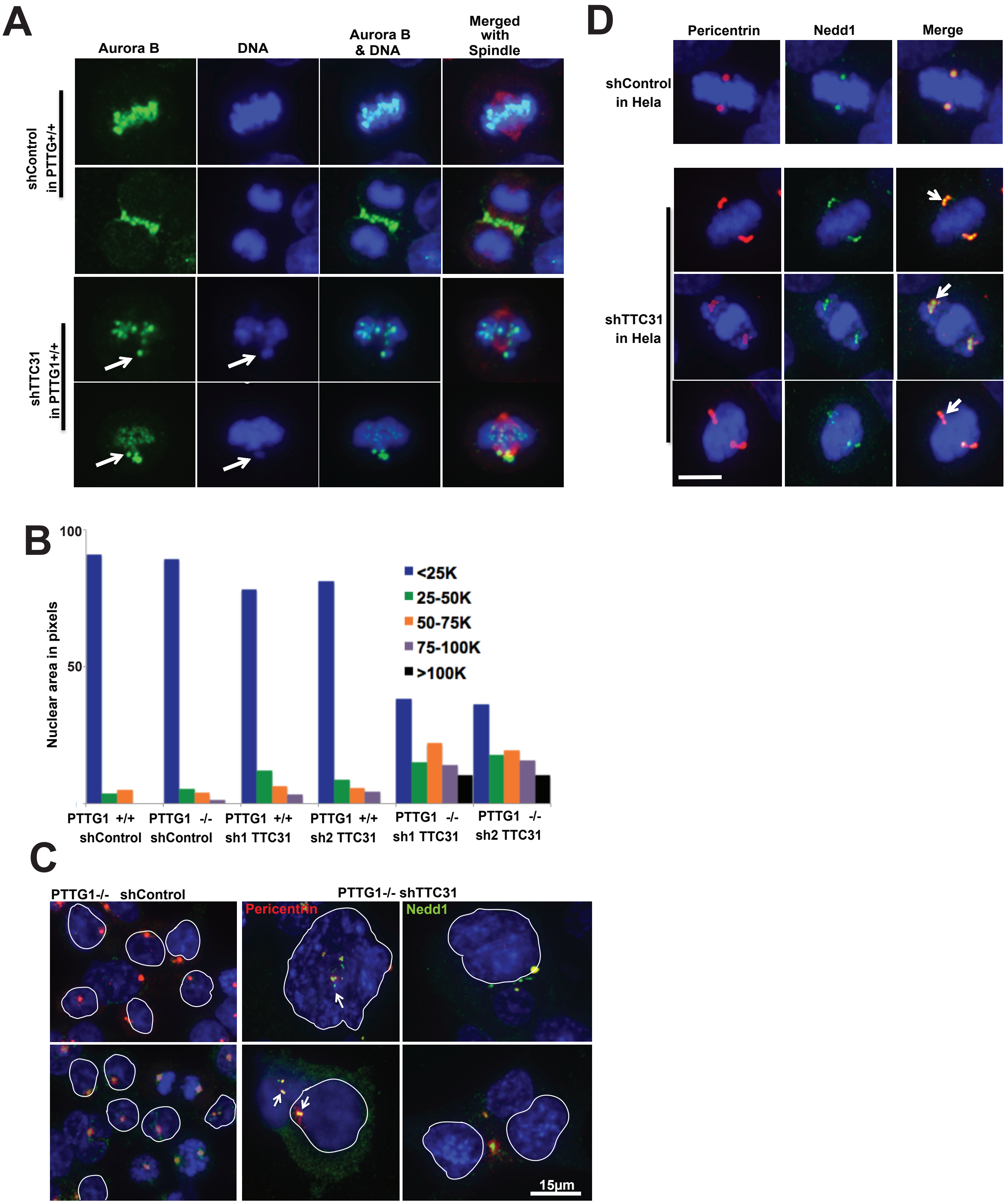

Supplementary Fig. 7

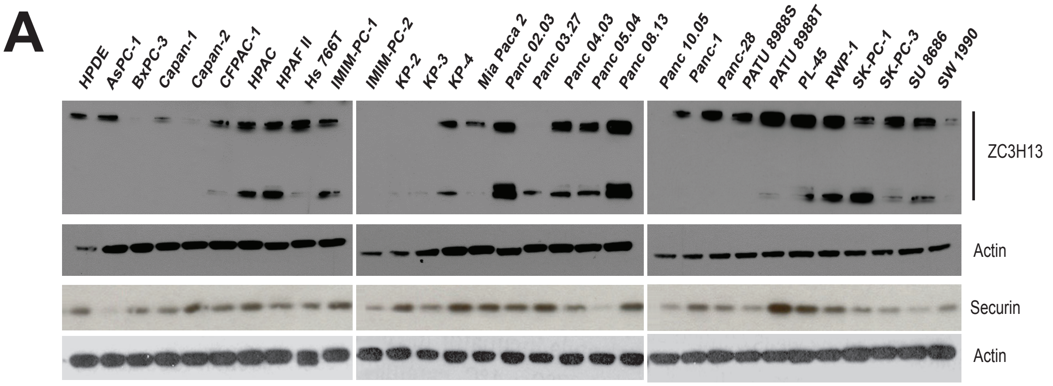

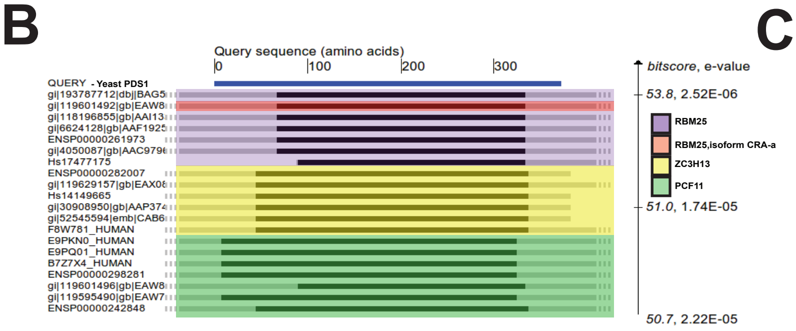

C

```
Length sequence 1: 373
Length sequence 2: 1668
Sequence #1: YEAST_PDS1
Sequence #2: HUMAN_ZC3H13
Length #1: 465
Length #2: 465
Matrix: BLOSUM62
Gap open: 5.0
Gap extend: 1.0
Length: 465
Identity: 116/465 (24.95%)
Similarity: 192/465 (41.29%)
Gaps: 141/465 (30.32%)
Score: 230.00
YEAST_PDS1         3 PANEDKENNIVYTGNESS-------GINFPQTPAHL-LK-RSHSNILKPP      43
                     |.|  ..|| .: .||.|       .:...:|| |: :: ..|.   ||
HUMAN_ZC3H13    1143 PTN--TTNN-TF-ANEDSHRKCHRTRVEKVETP-HVTIEDAQHR---KP-    1183

YEAST_PDS1        44 VRLDQLKRDAN--SNNGNTLKYIQGGKEVSPTK-RLHTHA-QQQGR---L      86
                       :|| ||.::  ||..|  :....|:..||:. ..|... .|.||    |
HUMAN_ZC3H13    1184 --MDQ-KRSSSLGSNRSN--RSHTSGRLRSPSNDSAHRSGDDQSGRKRVL    1228

YEAST_PDS1        87 PLAAKDNNRSKSF-IFPE-TS--NQSKDADLPQLQNTLSIRKNDQ---LR     129
                     ...::|..::||. |..| .|  :|.|..: |. ::|.|.|::.:   .|
HUMAN_ZC3H13    1229 HSGSRDREKTKSLEITGERKSRIDQLKRGE-PS-RSTSSDRQDSRSHSSR    1276

YEAST_PDS1       130 KLS-----QI-SR-----SRSRANHNDLLSNSRKLQK-----YGSVLGYN     163
                     :.|     |: ||     ||.|....|...:.|:.::     .......:
HUMAN_ZC3H13    1277 RSSPESDRQVHSRSGSFDSRDRLQERDRYEHDRERERERRDTRQREWDRD    1326

YEAST_PDS1       164 A---LPKMKS---L---------VLKDL--------ADSGKNEESSDDD     189
                     |   .|:.:.   |          ..:||        :||.:.:...|.|
HUMAN_ZC3H13    1327 ADKDWPRNRDRDRLRERERERERDKRRDLDRERERLISDSVERDRDRDRD    1376

YEAST_PDS1       190 ---EGNE-DS----ESKL-G---KKLQSALLKQDS--SDGENELNGGLGL     225
                        |.:: :|    |:|| |   :.|:|.  .:||  .|.| .::..||
HUMAN_ZC3H13    1377 RTFESSQIESVKRCEAKLEGEHERDLEST--SRDSLALDKE-RMDKDLG-    1422

YEAST_PDS1       226 FNEQGGLQQLIKNSTKNEQKTKNDKSDKTDD-YDIEIAPQRQEPLPYVPE     274
                      :.| |.::..| |.:.|....:|:| |.|| :.:...         ..|
HUMAN_ZC3H13    1423 -SVQ-GFEETNK-SERTESLEGDDES-KLDDAHSLGSG---------AGE    1459

YEAST_PDS1       275 GYSPFQQDDIEKLKTFNSPYKLDLEDEDDTPDKVDLLPLEQIDEEG--EK     322
                     ||.|...|:::::...::..:.|.:||:..||.:|::   .:|..|  .|
HUMAN_ZC3H13    1460 GYEPISDDELDEILAGDAEKREDQQDEEKMPDPLDVI---DVDWSGLMPK    1506

YEAST_PDS1       323 DETECITRNQEEGAAL----P----L---LSKNF--KEV-AAV-PTME-L     356
                     ...|  .|  |.||||    |    |   :||..  .|: |.| .|.: |
HUMAN_ZC3H13    1507 HPKE--PR--EPGAALLKFTPGAVMLRVGISKKLAGSELFAKVKETCQRL    1552

YEAST_PDS1       357 VYSEEGLDPEELEDL    371
                     :  |:   |::.::|
HUMAN_ZC3H13    1553 L--EK---PKDADNL   1562
```

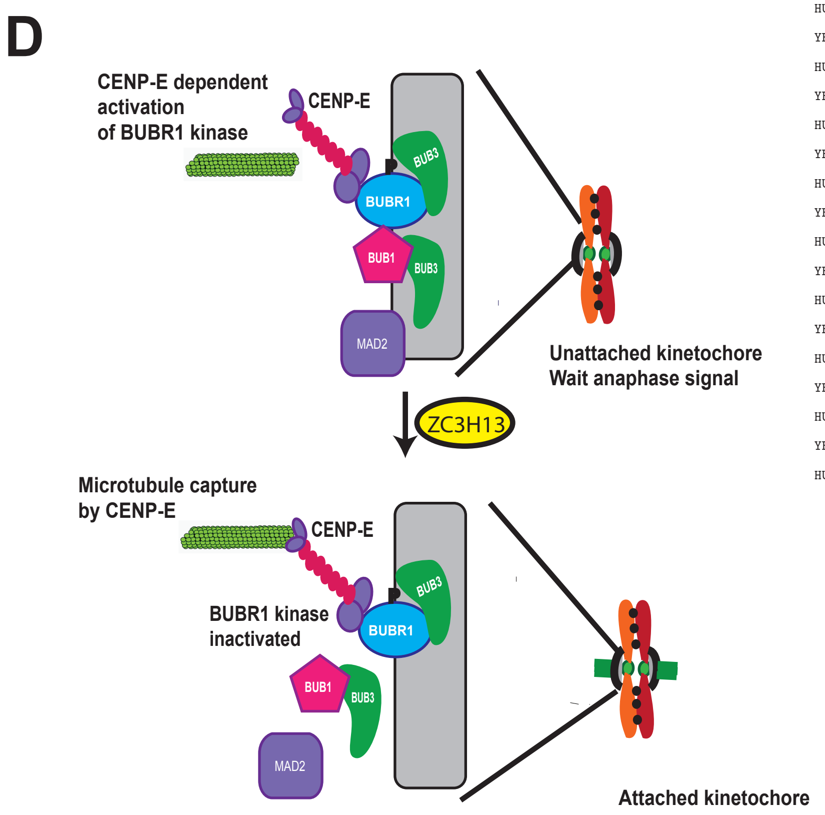

Supplementary Fig. 8

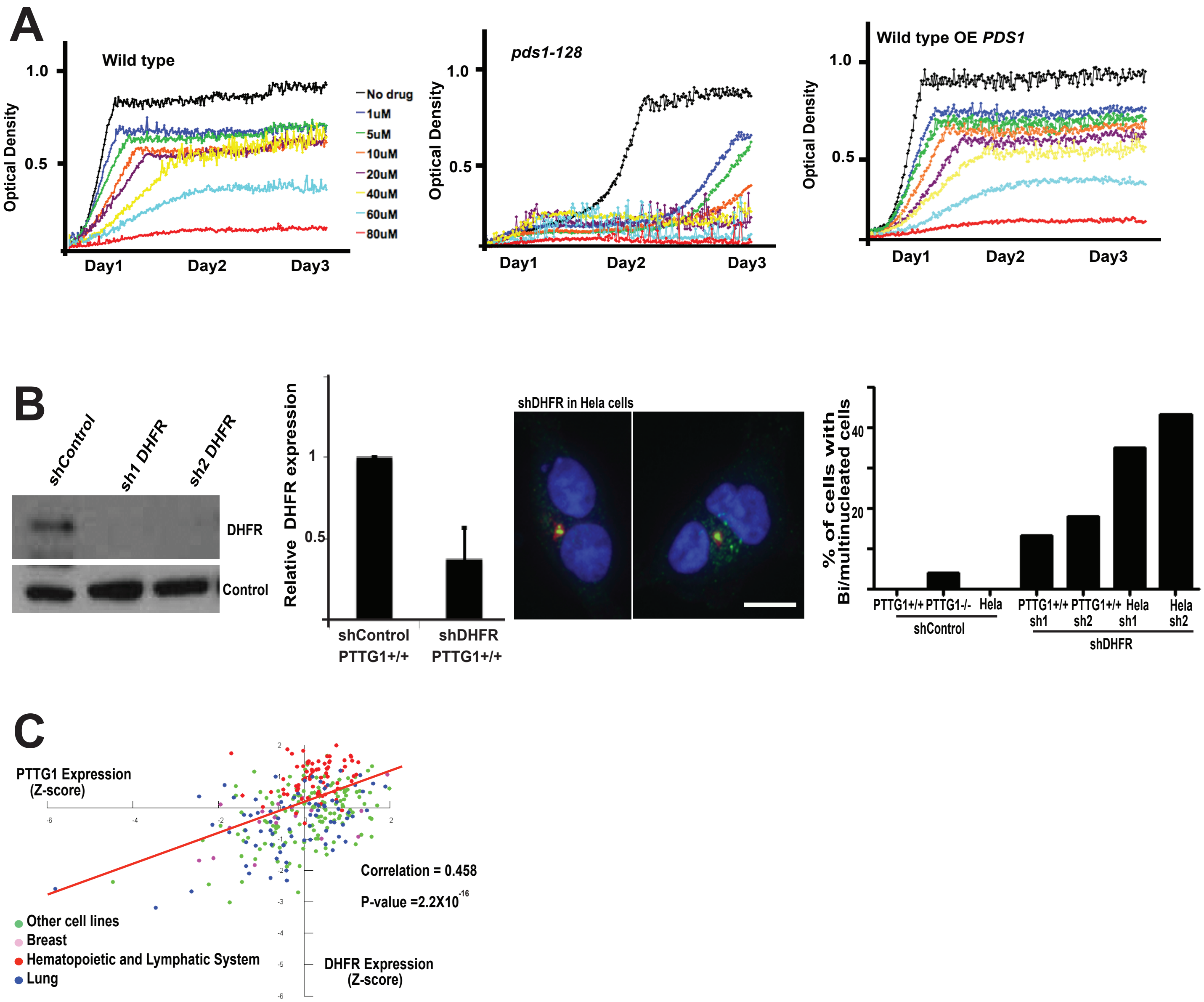

Supplementary Fig. 9

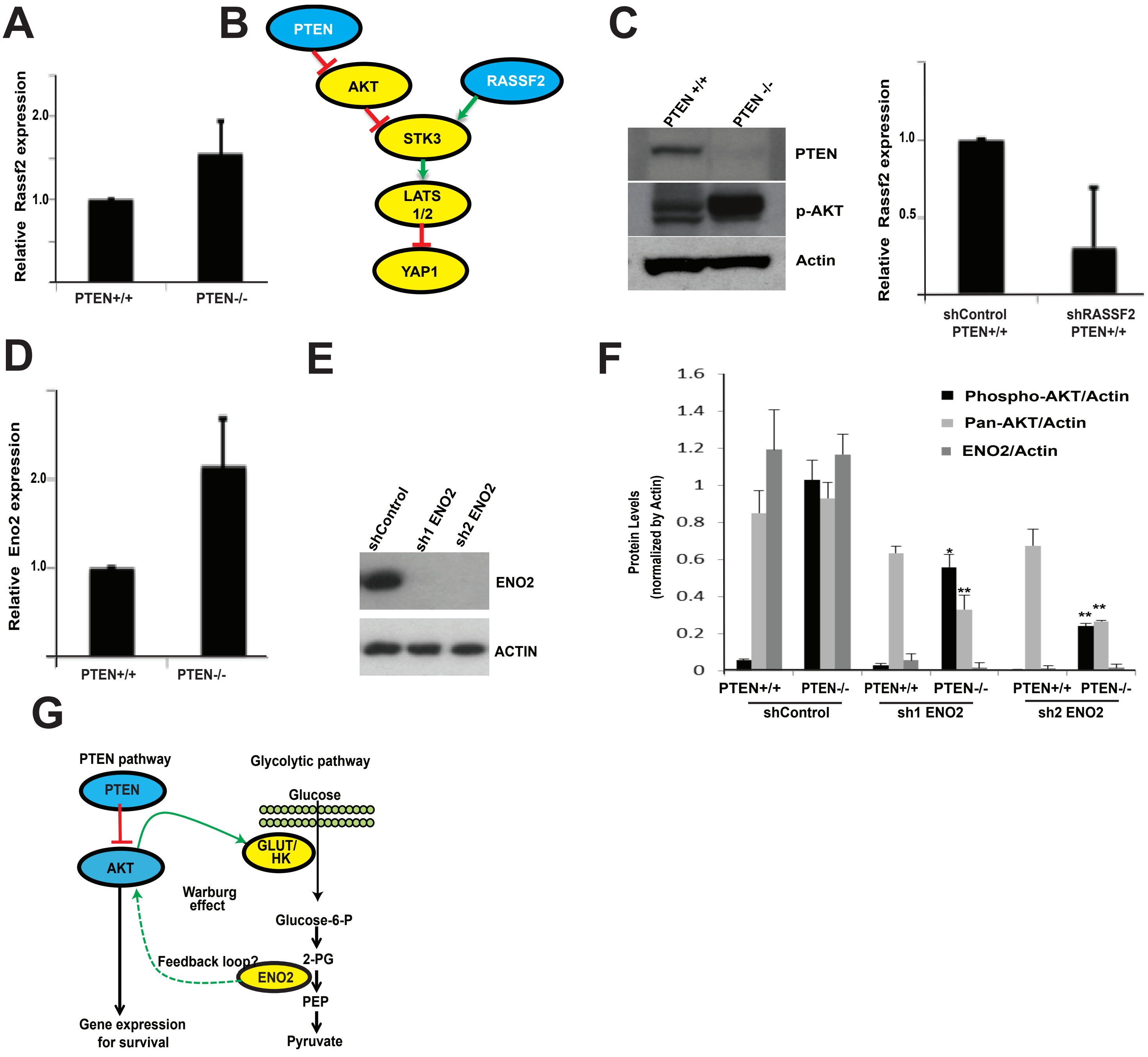

Supplementary Fig. 10

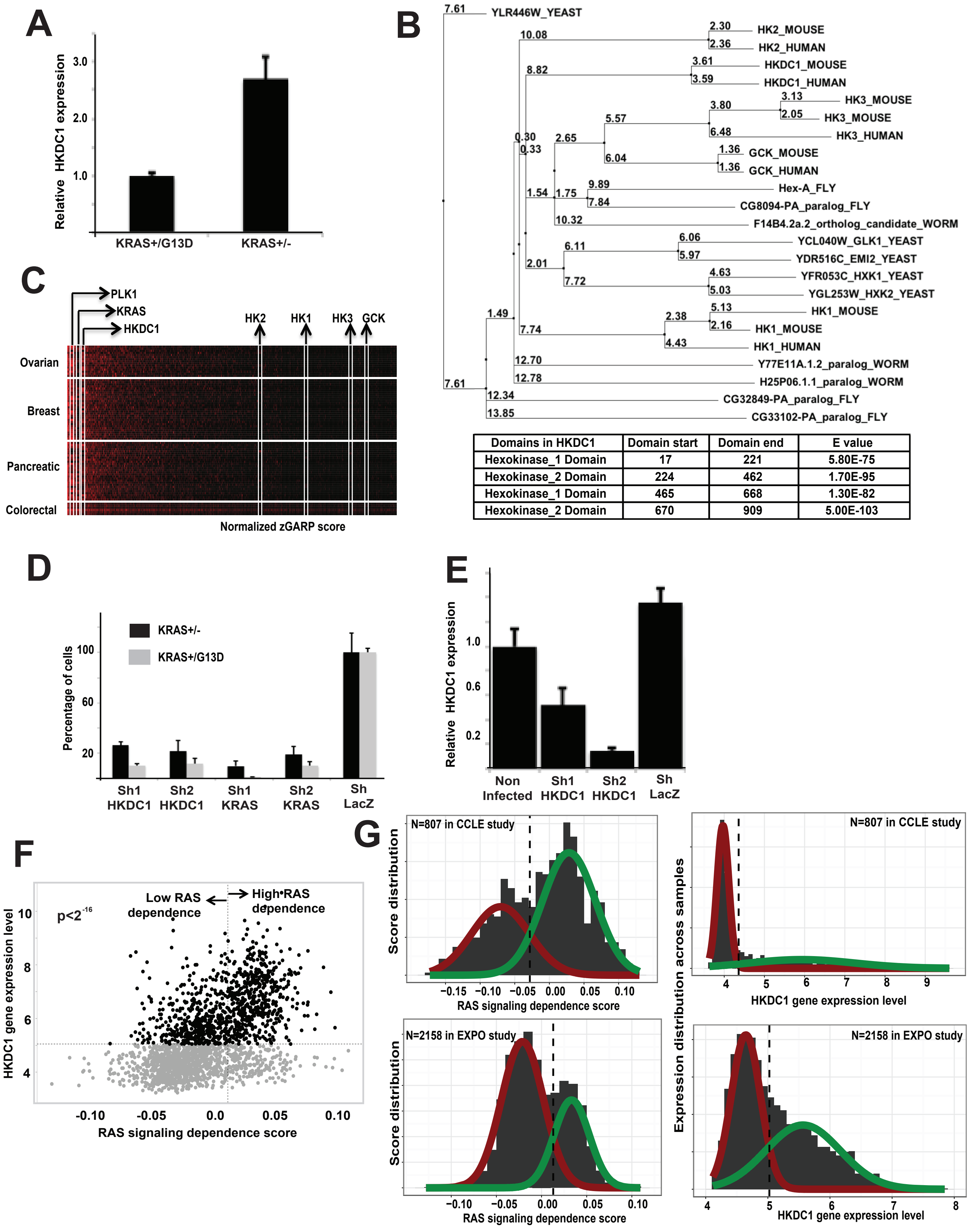

Domains in HKDC1	Domain start	Domain end	E value
Hexokinase_1 Domain	17	221	5.80E-75
Hexokinase_2 Domain	224	462	1.70E-95
Hexokinase_1 Domain	465	668	1.30E-82
Hexokinase_2 Domain	670	909	5.00E-103

Supplementary Fig. 11

A

	1	2	3	4	5	6	7
SNW1	171	0.899					
ESPL1	2	1.364					
DDB1	132	1.037					
CDK5RAP2	315	1.128					
ANAPC10	323	0.897					
CSK	443	1.109					
CSNK1E	547	1.099					
AURKA	629	1.297					
STK3	47	0.975					
STEAP3	101	0.882					
LIN54	175	0.975					
EZR	220	0.712					
AIF1L	303	1.11					
STK16	418	0.654					
PINK1	438	0.737					
COPZ1	476	0.921					
ITGA6	513	0.947					
CYC1	526	0.902					
PPIH	544	1.652					
FABP3	577	1.067					
B3GNT4	596	1.068					
HIST1H2BK	635	1.858					
ALDH18A1	664	1.222					
PA2G4	678	1.114					
SFRS12	6	0.943					
TRAF6	7	0.937					
CLEC4C	13	0.977					
CCDC21	14	1.191					
CHAF1A	27	1.315					
ZNF142	32	1.039					
SHC4	33	0.878					
MARK2	43	1.183					
DYRK2	49	0.903					
TRPC4AP	62	1.067					
LILRA3	68	0.91					
WASH2P	78	0.942					
F2RL2	106	1.13					
CENPC1	107	1.16					
FAM71D	109	1.028					
PDK2	111	1.653					
AXL	113	0.974					
UBASH3B	120	1.735					
CDK2	122	1.377					
ZNF358	127	1.305					
SLTM	137	0.808					
CDNF	144	0.817					
NUP188	145	1.196					
COL25A1	151	0.907					
SERPINB6	166	0.898					
SS18	173	0.919					
SFRS6	177	0.942					
CSNK2B	179	1.509					
CLDN10	180	0.954					
ENPP5	185	0.85					
PPL	186	0.592					
JAK1	195	0.626					
ASAP1	197	1.094					
ENAH	202	0.871					
PPP1R12B	212	1.13					
MOBKL1A	214	0.855					
PEX3	221	1.129					
CNOT6L	225	0.85					
S1PR5	230	1.058					
CPSF6	235	1.138					
CD248	238	0.961					
PRKRA	242	1.085					
NPR2	253	0.84					
LTK	281	0.941					
FKBP3	289	0.905					
RRM1	299	1.572					
OR1D2	308	1.387					
VEZF1	309	0.986					
AKNA	318	0.638					
GRHL3	330	1.026					
CDC42BPA	339	0.788					
ALMS1	342	1.118					
GNAS	346	1.032					
CCRL2	355	0.886					
FBXW2	356	1.009					
AKAP8L	359	0.728					
ERC1	390	0.733					
DAP3	391	0.901					
AMPD3	396	0.928					
SEZ6L2	398	0.95					
ZNF165	403	0.655					
AMPD2	404	1.047					
MLF1IP	423	1.193					
GABRA6	425	0.991					
MSH2	439	1.462					
MOGS	445	0.682					
ZNF362	447	0.942					
PTX3	458	0.958					
MELK	460	1.174					
RRBP1	461	1.134					
TTR	462	1.017					
NUP88	466	0.772					
GTF3C4	485	1.07					
SYF2	487	0.861					
ZFP42	490	0.933					
DPP7	491	1.077					
PAFAH1B2	492	1.419					
CXCR7	505	1.081					
PRKCQ	512	1.033					
SARS2	514	1.113					
TUBB	515	1.334					
SRRT	516	1.161					
TMED7	518	#N/A					
C22orf32	525	#N/A					
RGS11	527	0.853					
CBX7	533	1.055					
MAPK10	541	0.947					
CFB	545	1.45					
RRM2	550	1.257					
KPNA3	554	1.116					
SYT11	556	1.194					
GTF3C5	565	1.216					
TUBB3	568	2.198					
ATP11C	579	0.919					
ZC4H2	586	0.223					
RNF41	588	0.935					
RPN2	591	1.008					
PAFAH1B3	599	2.569					
RGPD4	611	0.83					
C14orf145	617	0.966					
MTMR1	624	1.034					
KIAA0586	625	0.954					
FAM98A	626	1.064					
PDK4	628	1.026					
AZGP1	646	0.938					
TJP2	648	0.644					
ACTR3B	652	0.896					
HNF4A	655	0.543					
DMD	673	1.015					
PPP4C	674	1.022					
ADRBK1	679	1.14					
CLIP4	680	0.632					
MAP3K3	683	1.567					
DARC	684	1.221					
AP1G1	691	1.071					
ZC3H13	707	1.011					
RSC1A1	715	#N/A					
GLIPR1L2	718	1.034					
ARSE	721	0.976					
ERBB4	732	0.917					
FBXL7	735	0.924					
RNF141	740	0.725					

1- Ranking in the SSL screen
2- Differential expression
3- PMID: 15616564 (Nature 2003)
4- PMID: 20360735 (Nature 2010)
5- PMID: 20360068 (Science 2010)
6- PMID: 16564017 (Cell 2006)
7- PMID: 14654843 (Nature 2003)

Supplementary Fig. 12

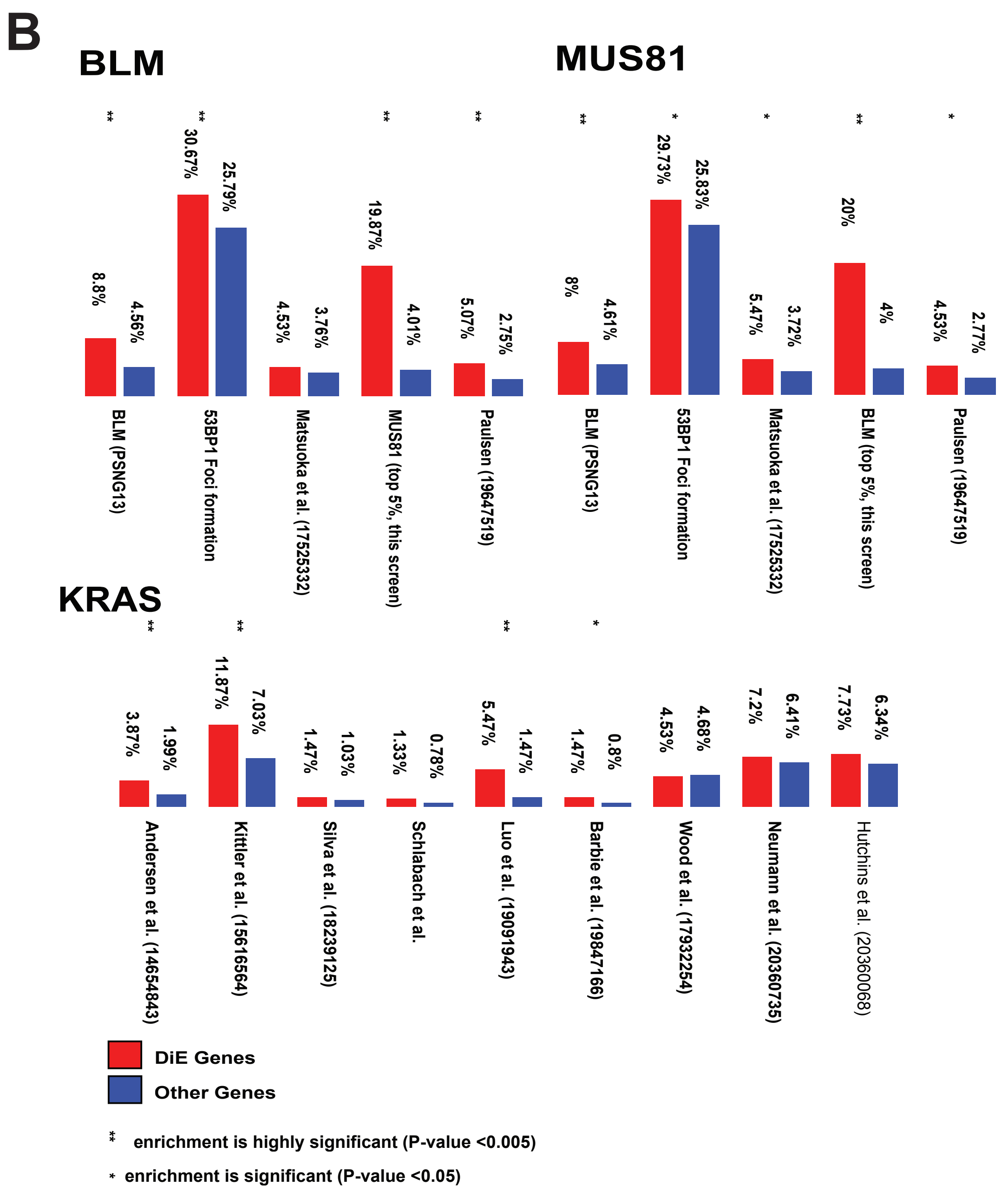

Supplementary Fig. 12 cont'd

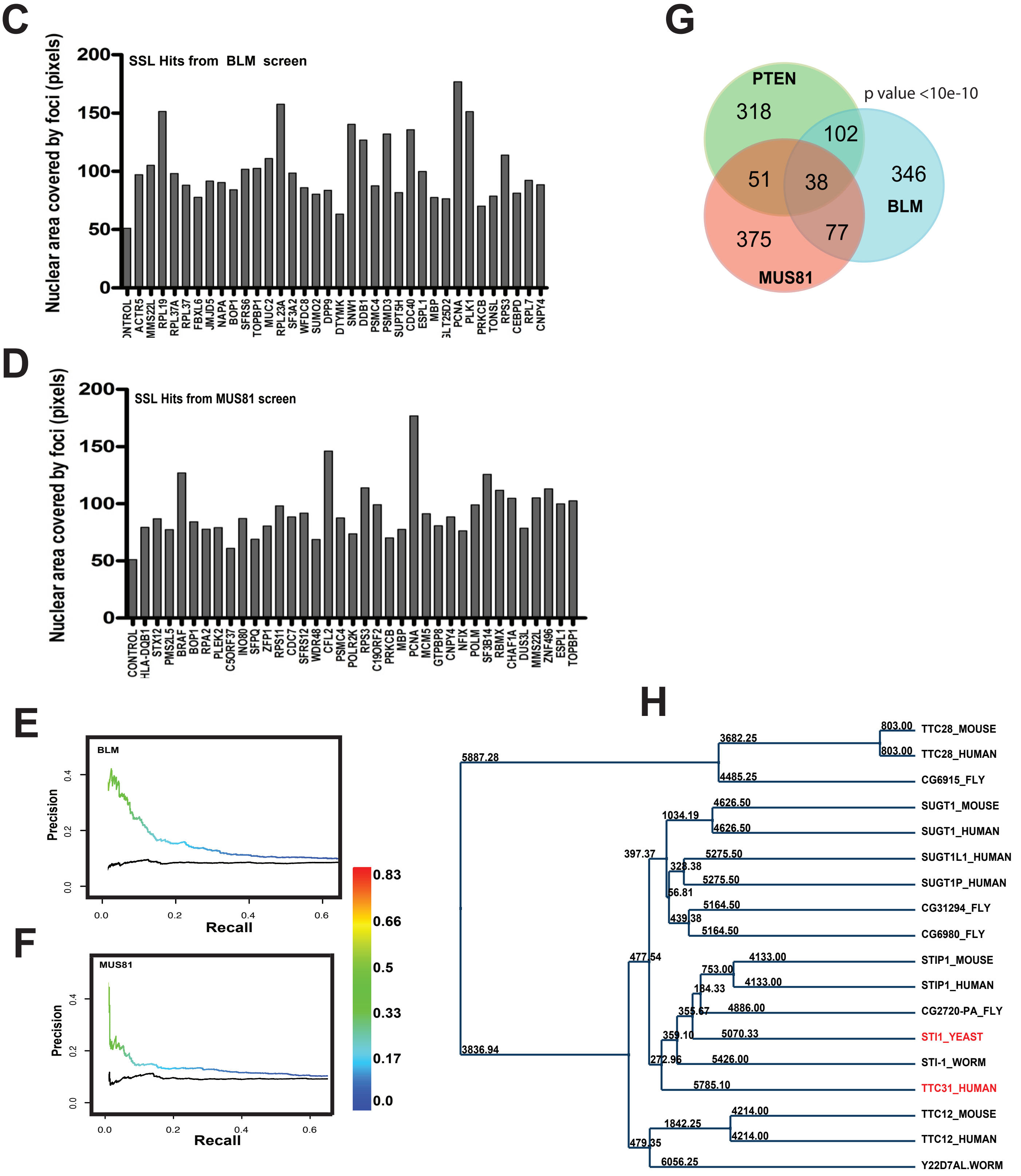

Supplementary Fig. 12 Cont'd

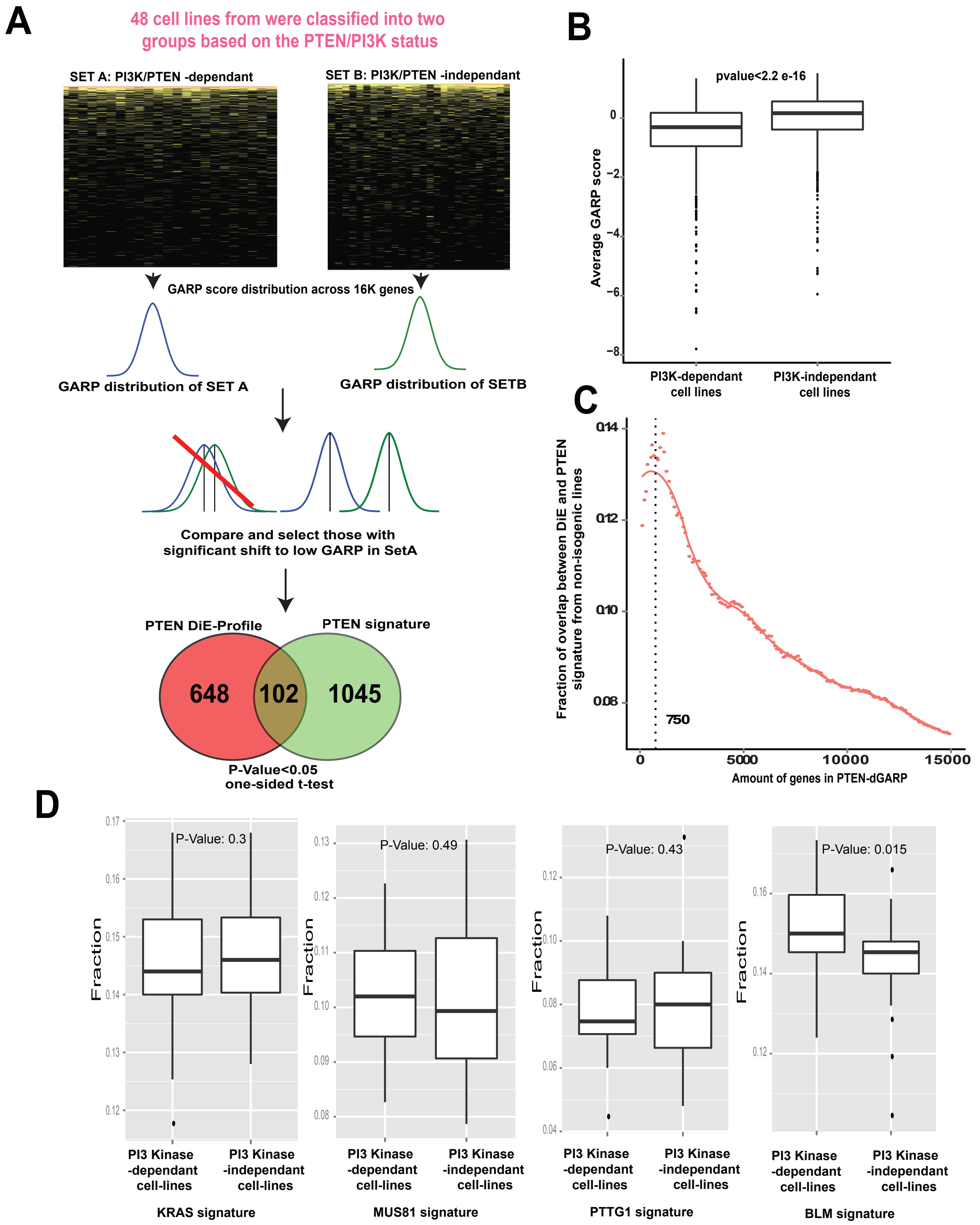

Supplementary Figure 13

E

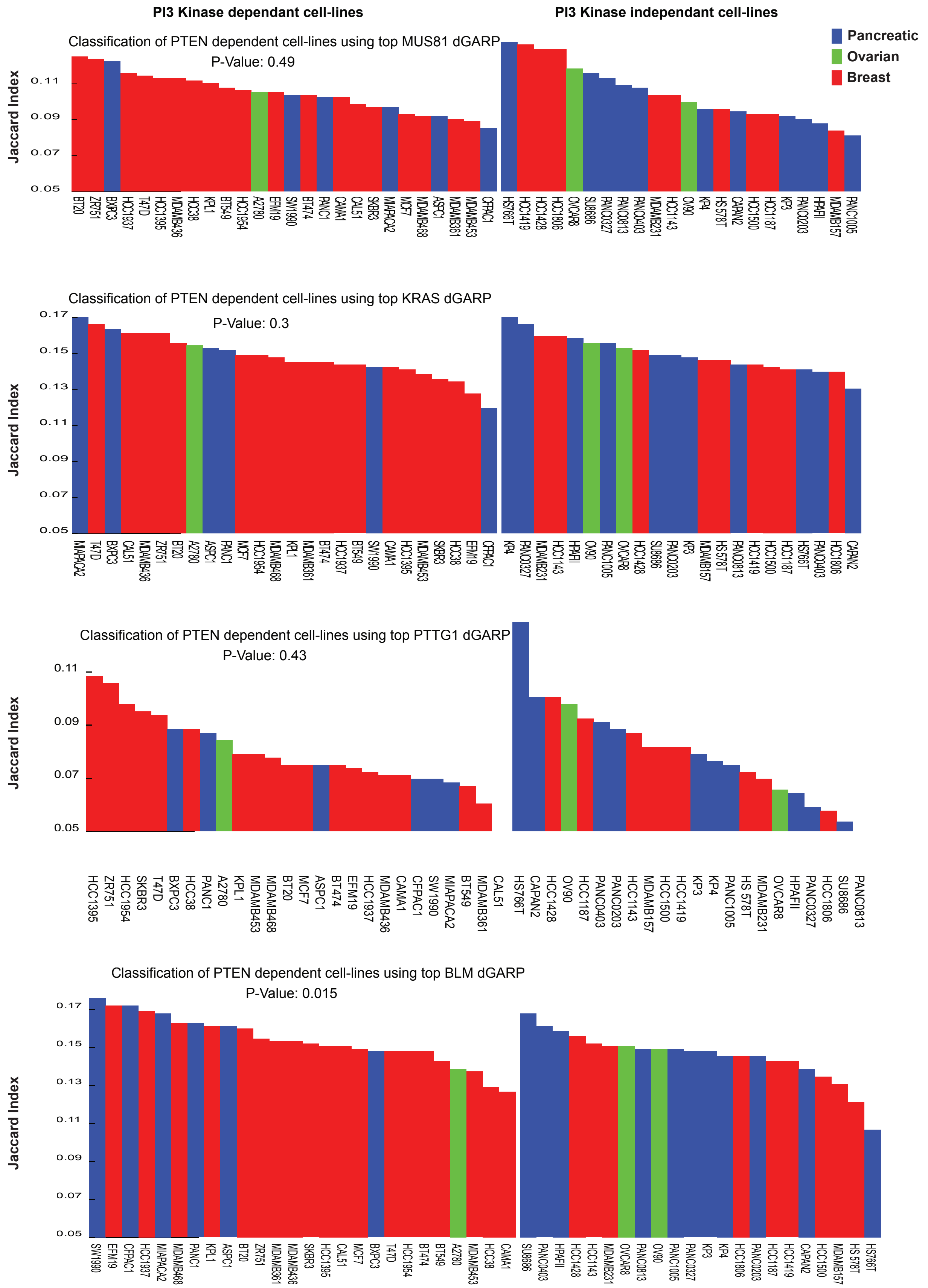

Supplementary Figure 13 Cont'd

A

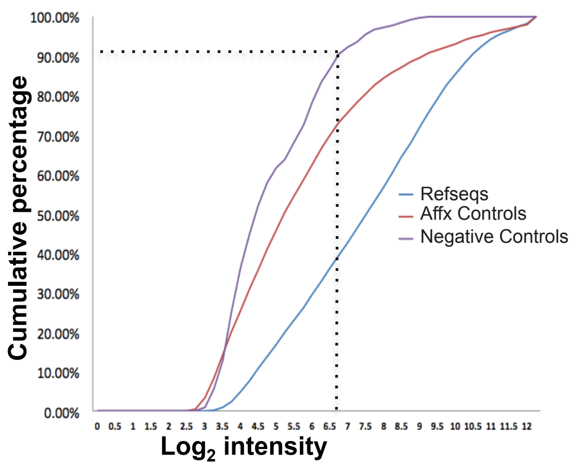

B

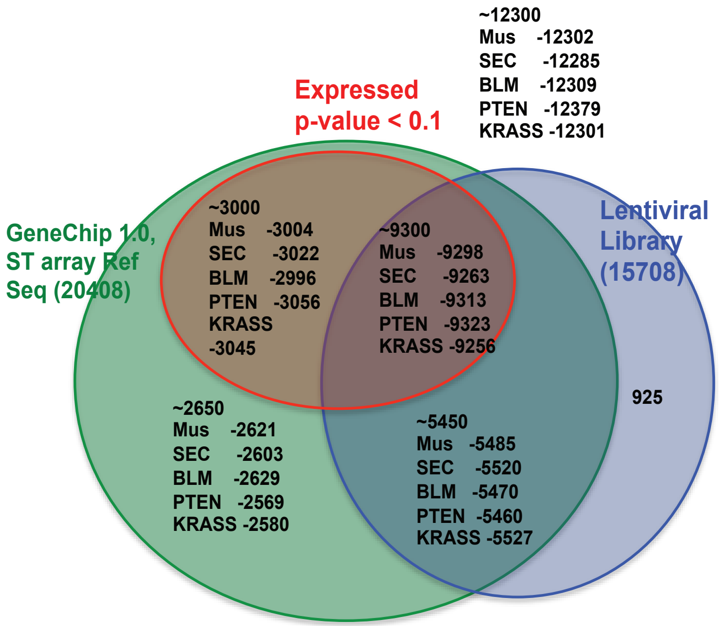

C

	Refseqs						Affx Controls						Negative Controls					
	BLM	MUS	WT	PTEN	KRAS	SEC	BLM	MUS	WT	PTEN	KRAS	SEC	BLM	MUS	WT	PTEN	KRAS	SEC
Mean	7.414	7.419	7.411	7.410	7.408	7.405	7.194	6.890	6.993	6.890	7.245	7.023	5.748	5.750	5.739	5.656	5.781	5.914
Standard Error	0.015	0.015	0.015	0.015	0.015	0.015	0.376	0.347	0.367	0.351	0.395	0.364	0.042	0.042	0.043	0.042	0.044	0.043
Median	7.403	7.396	7.388	7.379	7.365	7.387	6.839	6.333	6.515	6.626	6.665	6.433	5.223	5.273	5.217	5.115	5.149	5.502
Standard Deviation	2.257	2.262	2.241	2.243	2.215	2.260	2.837	2.621	2.770	2.651	2.985	2.748	2.287	2.276	2.295	2.285	2.353	2.324
Sample Variance	5.093	5.118	5.022	5.032	4.908	5.109	8.048	6.872	7.673	7.028	8.910	7.550	5.229	5.181	5.266	5.223	5.538	5.399
Minimum	2.873	2.796	2.771	2.799	2.864	2.816	2.845	2.626	2.805	2.757	2.969	2.740	2.600	2.496	2.496	2.554	2.559	2.571
Maximum	14.117	14.035	14.095	14.009	14.124	14.037	13.708	13.110	13.479	13.312	13.978	13.346	14.088	14.006	14.052	13.960	14.231	14.134
Count	21574	21574	21574	21574	21574	21574	57	57	57	57	57	57	2904	2904	2904	2904	2904	2904
Confidence Level (95.0%)	0.096	0.097	0.096	0.096	0.095	0.096	2.356	2.177	2.301	2.202	2.479	2.282	0.266	0.265	0.267	0.266	0.274	0.270

Supplementary Fig. 14
