# Supplementary material for: A negative genetic interaction map in isogenic cancer cell lines reveals cancer cell vulnerabilities
Source: Mol Syst Biol. 2013 Oct 8;9:696. doi: 10.1038/msb.2013.54 (PMC3817404; doi:10.1038/msb.2013.54)
Supplement: Supplementary Table 11 [file msb201354-s12.pdf]

```
Name 1: CDK12_HUMAN
Name 2: CTK1_YEAST

Cyclin-dependent kinase 12 OS=Homo sapiens GN=CDK12 PE=1 SV=2
CTD kinase subunit alpha OS=Saccharomyces cerevisiae (strain ATCC 204508 / S288c
) GN=CTK1 PE=1 SV=1

Needed for following interaction(s):
yeast   genetic RASK_HUMAN      CDK12_HUMAN     KRAS    CDK12   IRA2_YEAST
CTK1_YEAST

Length sequence 1:      1490
Length sequence 2:      528
Sequence #1: CDK12_HUMAN
Sequence #2: CTK1_YEAST
Length #1: 612
Length #2: 612
Matrix: BLOSUM62
Gap open: 2.0
Gap extend: 1.0
Length: 612
Identity: 220/612 (35.95%)
Similarity: 318/612 (51.96%)
Gaps: 176/612 (28.76%)
Score: 979.00

CDK12_HUMAN      538 PL-PTTTPPPQTPPLPPLPPIPALPQQPPL-PPSQPAFSQVPASSTSTLP    585
                     || ...:|.||:  | ..|| |  |::  : ..|  .: |   |:...:
CTK1_YEAST        20 PLFGKRSPNPQS--L-ARPP-P--PKR--IRTDS--GY-Q---SNMDNI-     54

CDK12_HUMAN      586 PSTHSKTSAVSSQANSQP-PVQVSVK--TQVS-V--TAAIPHLKTSTLPP    629
                      |:| :   |:|  |.|| ..: | :  ..:| .  |:    .:||:
CTK1_YEAST        55 -SSH-R---VNS--NDQPGHTK-S-RGNNNLSRYNDTS----FQTSS---     88

CDK12_HUMAN      630 LPLPPLLPGD--DDMDSPKETLPSKPVKKEKEQRTRHLLTDLPLPPELPG    677
                          ...|.  ::.::..|..| |.:|:: |  |:   .:. | ..||.
CTK1_YEAST        89 -----RYQGSRYNNNNTSYENRP-KSIKRD-E--TK---AEF-L-SHLPK    124

CDK12_HUMAN      678 GDLSPPDSPEPKA-I-----TP-P-QQPY---K----K----R----PKI    704
                     |    |.|.| |: .     |. . :..|   |    |    |    .|:
CTK1_YEAST       125 G----PKSVE-KSRYNNSSNTSNDIKNGYHASKYYNHKGQEGRSVIAKKV    169

CDK12_HUMAN      705 CCPRYG---ERRQTESDWGKRCVDKFDI-IGI-IGEGTYGQVYKAKDKDT    749
                       | ..   ::|.| |      |  : : | : :||||||:|||||:.:|
CTK1_YEAST       170 --P-VSVLTQQRST-S------V--Y-LRI-MQVGEGTYGKVYKAKNTNT    205

CDK12_HUMAN      750 GE-LVALKKVRLDNEKEGFPITAIREIKILRQLI-HRSVVNMKEIVTDKQ    797
                      | ||||||:||..|:||||||:|||||:| |.. |.:|..:|||:.:.|
CTK1_YEAST       206 -EKLVALKKLRLQGEREGFPITSIREIKLL-QSFDHPNVSTIKEIMVESQ    253

CDK12_HUMAN      798 DALDFKKDKGAFYLVFEYMDHDLMGLL---ESGL-V-HFSE-DHIKSFMK    841
                             | ..|::|||.|:||.|||   |  : : | |: .|:  | |
CTK1_YEAST       254 --------K-TVYMIFEYADNDLSGLLLNKE--VQISH-SQCKHL--F-K    288

CDK12_HUMAN      842 QLMEGLEYCH-KKNFLHRDIKCSNILLNNSGQIKLADFGLAR-LYNSEES    889
                     ||:.|:||.| .| .||||:|.||||::|.|.:|:.|||||| : |   |
CTK1_YEAST       289 QLLLGMEYLHDNK-ILHRDVKGSNILIDNQGNLKITDFGLARKM-N---S    333

CDK12_HUMAN      890 R-PYTNKVITLWYRPPELLLGEERY-TPAIDVWSCGCILGELFTKKPIFQ    937
                     | .|||:||||||||||||||...| | .:|:|.|||:|.|||.|..|||
CTK1_YEAST       334 RADYTNRVITLWYRPPELLLGTTNYGT-EVDMWGCGCLLVELFNKTAIFQ    382

CDK12_HUMAN      938 -ANLELAQLELISRLCGSPCPAV--WPDVIKLP-YFNTMKPKK--QYRRR    981
                      :| ||.|:|.|.::.|:  |.:  ||.:..:| :|..| |::  :|...
CTK1_YEAST       383 GSN-ELEQIESIFKIMGT--PTINSWPTLYDMPWFFMIM-PQQTTKYVNN    428

CDK12_HUMAN      982 LREEF-SFIPSA-ALDL-LDHMLTLDPSKRCTAEQTLQSDFLKDVELSKM   1028
                     ..|:| |.:||: .|.| : ::|..|.:||.:|.:.||||:.|: | .|
CTK1_YEAST       429 FSEKFKSVLPSSKCLQLAI-NLLCYDQTKRFSATEALQSDYFKE-E-PK-    474

CDK12_HUMAN     1029 AP-P---D-LPHWQDCHELWS-KKRRRQRQSGVVVEEPPPS-KTSRKETT   1071
                      | |   | |   ..||| :. |..|:|::..::      | .|:.|  .
CTK1_YEAST       475 -PEPLVLDGL---VSCHE-YEVKLARKQKRPNIL------STNTNNK--G    511
```

```
CDK12_HUMAN     1072 SGTSTEPVKNSS   1083
                     :|.|..  .|::
CTK1_YEAST       512 NGNSNN--NNNN    521

--------------------------------------------
Name 1: PCNA_HUMAN
Name 2: PCNA_YEAST

Proliferating cell nuclear antigen OS=Homo sapiens GN=PCNA PE=1 SV=1
Proliferating cell nuclear antigen OS=Saccharomyces cerevisiae (strain ATCC 2045
08 / S288c) GN=POL30 PE=1 SV=1

Needed for following interaction(s):
yeast   genetic MUS81_HUMAN     PCNA_HUMAN      MUS81   PCNA    MUS81_YEAST
PCNA_YEAST

Length sequence 1:      261
Length sequence 2:      258
Sequence #1: PCNA_HUMAN
Sequence #2: PCNA_YEAST
Length #1: 280
Length #2: 280
Matrix: BLOSUM62
Gap open: 2.0
Gap extend: 1.0
Length: 280
Identity: 107/280 (38.21%)
Similarity: 175/280 (62.50%)
Gaps: 43/280 (15.36%)
Score: 565.00

PCNA_HUMAN         1 MFEARLVQGSILKKVLEALKD---LIN-EACWDISSSGVNL-QSMDSSHV     45
                     |.||:..:.|:.|::::..||   |:| : |   ...|: : |::|.|.|
PCNA_YEAST         1 MLEAKFEEASLFKRIIDGFKDCVQLVNFQ-C---KEDGI-IAQAVDDSRV     45

PCNA_HUMAN        46 SLVQLTLRSEGFDTYRCDRNLAMGVNLTSMSKILKCAGNE-DIITLRAED     94
                     .||.|.:..|.|..||||..:.:|::|||:||||:| ||. |.:||.| |
PCNA_YEAST        46 LLVSLEIGVEAFQEYRCDHPVTLGMDLTSLSKILRC-GNNTDTLTLIA-D     93

PCNA_HUMAN        95 NA-DTLALVFEAPNQEKVSDYEMKLMDLDVEQLGIPEQ-EY-SCVVKMPS    141
                     |. |::.|:||...::::::|.:||||:|.:.|.| |: :| | .:.:||
PCNA_YEAST        94 NTPDSIILLFEDTKKDRIAEYSLKLMDIDADFLKI-EELQYDS-TLSLPS    141

PCNA_HUMAN       142 GEFARICRDLSHIGDAV-V-ISCAKDGVKFSASGELGNGN--IK-LSQTS    186
                     .||::|.||||.:.|:: : |:  |:.:||.|.|::|:|:  || .
PCNA_YEAST       142 SEFSKIVRDLSQLSDSINIMIT--KETIKFVADGDIGSGSVIIKPF----    185

PCNA_HUMAN       187 NVDKE--EEAVTIEMNEPVQLTF-ALRY-LNFFTKATPLSSTVTLSMSAD    232
                      ||.|  |.::.:||::||.||| | :| |:.. |.:.||..|.:.:|::
PCNA_YEAST       186 -VDMEHPETSIKLEMDQPVDLTFGA-KYLLDII-KGSSLSDRVGIRLSSE    232

PCNA_HUMAN       233 VP-LVVEYKIADM--GHLKYYLAPKIEDEE    259
                     .| |   ::. |:  |.|:::||||..|||
PCNA_YEAST       233 APAL---FQF-DLKSGFLQFFLAPKFNDEE    258

--------------------------------------------
Name 1: PRDX3_HUMAN
Name 2: TSA1_YEAST

Thioredoxin-dependent peroxide reductase, mitochondrial OS=Homo sapiens GN=PRDX3
 PE=1 SV=3
Peroxiredoxin TSA1 OS=Saccharomyces cerevisiae (strain ATCC 204508 / S288c) GN=T
SA1 PE=1 SV=3

Needed for following interaction(s):
yeast   genetic PRDX3_HUMAN     MUS81_HUMAN     PRDX3   MUS81   TSA1_YEAST
MUS81_YEAST

Length sequence 1:      256
Length sequence 2:      196
Sequence #1: PRDX3_HUMAN
Sequence #2: TSA1_YEAST
Length #1: 198
Length #2: 198
```

```
Matrix: BLOSUM62
Gap open: 2.0
Gap extend: 1.0
Length: 198
Identity: 119/198 (60.10%)
Similarity: 149/198 (75.25%)
Gaps: 9/198 (4.55%)
Score: 619.00

PRDX3_HUMAN       64 A-VTQ-HAPYFKGTAVVNGEFKD-LSLDDFKGKYLVLFFYPLDFTFVCPT    110
                     | | | .||.||.||||:|.| | :|||.:||||:||.|.||.|||||||
TSA1_YEAST         3 AQV-QKQAPTFKKTAVVDGVF-DEVSLDKYKGKYVVLAFIPLAFTFVCPT     50

PRDX3_HUMAN      111 EIVAFSDKANEFHDVNCEVV-AVSVDSHFSHLAWINTPRKNGGLGHMNIA    159
                     ||:|||:.|.:|.:...:|: | |.||.:|.|||.|.|||.||||.:||.
TSA1_YEAST        51 EIIAFSEAAKKFEEQGAQVLFA-STDSEYSLLAWTNIPRKEGGLGPINIP     99

PRDX3_HUMAN      160 LLSDLTK-QISRDYGVLLEGSGLALRGLFIIDPNGVIKHLSVNDLPVGRS    208
                     ||:| |. .:|||||||:|..|:||||||||||.|||:|:::|||||||:
TSA1_YEAST       100 LLAD-TNHSLSRDYGVLIEEEGVALRGLFIIDPKGVIRHITINDLPVGRN    148

PRDX3_HUMAN      209 VEETLRLVKAFQYVETHGEVCPANWTPDSPTIKPSPAASKEYFQKVNQ    256
                     |:|.||||:|||:.:.:.:|.|.|.|.||||.:.||||:...|||||:..|:
TSA1_YEAST       149 VDEALRLVEAFQWTDKNGTVLPCNWTPGAATIKPTVEDSKEYFEAANK    196

--------------------------------------------
Name 1: CDC23_HUMAN
Name 2: CDC23_YEAST

Cell division cycle protein 23 homolog OS=Homo sapiens GN=CDC23 PE=1 SV=3
Anaphase-promoting complex subunit CDC23 OS=Saccharomyces cerevisiae (strain ATC
C 204508 / S288c) GN=CDC23 PE=1 SV=1

Needed for following interaction(s):
yeast   genetic CDC23_HUMAN     PTTG1_HUMAN     CDC23   PTTG1   CDC23_YEAST
SECU_YEAST
yeast   physical        CDC23_HUMAN     PTTG1_HUMAN     CDC23   PTTG1   CDC23_YE
AST     SECU_YEAST

Length sequence 1:      597
Length sequence 2:      626
Sequence #1: CDC23_HUMAN
Sequence #2: CDC23_YEAST
Length #1: 688
Length #2: 688
Matrix: BLOSUM62
Gap open: 2.0
Gap extend: 1.0
Length: 688
Identity: 246/688 (35.76%)
Similarity: 361/688 (52.47%)
Gaps: 214/688 (31.10%)
Score: 1062.00

CDC23_HUMAN       22 INSDFS-D--LREIKKQLLLIAGLTRE--RGLLH-SSKWSAE-LAFSL-P     63
                     :|.| | |  :.:|:.||.. |. | |  |..|: ||||:|| || .| .
CDC23_YEAST        1 MNDD-SQDKIIHDIRIQLRK-AA-T-ELSRWKLYGSSKWAAEALA-GLAE     45

CDC23_HUMAN       64 AL------PLA-E--L---Q--PP-----PP----ITE-EDAQDMDAYTL     89
                     |:      .|| |  |   |  |.     |.    ::| |  .|:  |.|
CDC23_YEAST       46 AIDVDQTHSLADESPLRNKQGVPKQMFEIPQNGFGLSETE--YDL--YLL     91

CDC23_HUMAN       90 AKAYFDVKEYDRAAHFLHG-CNSKKAY--FLYMYSRYLSGEKK-------    129
                     ....||.||:||...||.. .|   .|  ||.:||::||.:||
CDC23_YEAST       92 GSTLFDAKEFDRCVFFLKDVTN---PYLKFLKLYSKFLSWDKKSQESMEN    138

CDC23_HUMAN      130 -----K-DDE--TV----D-SLG--PL-EKG-Q---VK---NE-------    149
                          | .||  ..     | | |  .: :.| |   :|   ||
CDC23_YEAST      139 ILTTGKFTDEMYRANKDGDGS-GNEDINQSGHQRANLKMVSNEHESQSNI    187

CDC23_HUMAN      150 -A-LRELR--VELS-K-K---HQARELDGFG-LYLY--GVVLR--KLDLV    185
                      : |:|:.  :| | : |   .:| :| |.. || |  ||:|:  | ::.
CDC23_YEAST      188 SSILKEINTFLE-SYEIKIDDDEA-DL-GLALLY-YLRGVILKQEK-NIS    232

CDC23_HUMAN      186 KEAIDVFVEATHVLPLHWGAWLEL--CNL--ITD----KEML------KF    221
                     | |:..|:::......:|..||||  | |  :.|    ...|      ||
CDC23_YEAST      233 K-AMSSFLKSLSCYSFNWSCWLELMDC-LQKVDDALLLNNYLYQNFQFKF    280

CDC23_HUMAN      222 LS--L-PD-T--W--M-KEFF-LAHIYTEL--QLIE---EALQKYQNLID    256
                      |  | .. |  :  | | || | .::.||  || |   |.|: :  |:.
CDC23_YEAST      281 -SENLGSQRTIEFNIMIK-FFKL-KVFEELNGQL-EDYFEDLE-F--LLQ    323

CDC23_HUMAN      257 V--GFS--KSSYIVSQIA-VAYHNIRD-IDKALSIFNELRKQDPYRIEN-    299
                     |  .|:  | :|  :  | ::|:|: | : .|.|.|:::.||||||: |
CDC23_YEAST      324 VFPNFTFLK-AY--N--ATISYNNL-DYV-TAESRFDDIVKQDPYRL-ND    365

CDC23_HUMAN      300 MDTFSNLLYVRSM-K-SELSYLAHNLCEIDKYRVETCCVIGNYYSLRSQH    347
                     ::|:||:|||  | | |:|:|||..:.:||::|.||||:|.||||.|.:|
CDC23_YEAST      366 LETYSNILYV--MQKNSKLAYLAQFVSQIDRFRPETCCIIANYYSARQEH    413

CDC23_HUMAN      348 EKAALYFQRALKLNPRYLGAWTLMGHEYMEMKNTSAAIQAYRHAIEVNKR    397
                     ||:.:||:|||.|:.:...||||||||::|:.|:.|||:.||.|:::..|
CDC23_YEAST      414 EKSIMYFRRALTLDKKTTNAWTLMGHEFVELSNSHAAIECYRRAVDICPR    463

CDC23_HUMAN      398 DYRAWYGLGQTYEILKMPFYCLYYYRRAHQLRPNDSRM-LVALGECYEKL    446
                     |::||:||||.|.:|.|..|.|||:::|..|:|.|.|: .| |||||.|.
CDC23_YEAST      464 DFKAWFGLGQAYALLDMHLYSLYYFQKACTLKPWDRRIWQV-LGECYSKT    512

CDC23_HUMAN      447 -NQLVEAKKCYWRAY-A---VGDVEKMAL-VKLAKLHEQLTESE-QAAQC    489
                      |: |||.|||.|:. |    | | :..:: .:||:|:|:| | : |  :|
CDC23_YEAST      513 GNK-VEAIKCYKRSIKASQTV-D-QNTSIYYRLAQLYEEL-E-DLQ--EC    555

CDC23_HUMAN      490 --Y-IKYIQDIYSCGEIVEHL--EEST--AFR-YLAQYYF--KC---KL-    525
                       : :|.: |: .  |::|.:  :| |  | | :||  .|  |.   :|
CDC23_YEAST      556 KKFMMKCV-DV-E--ELLEGIVTDE-TVKA-RLWLA--IFEIKAGNYQLA    597

CDC23_HUMAN      526 WDEA----S-TCAQKCCAFNDTREEGKALLRQILQLRN    558
                     :|.|    | | :|      :. ||.:.|.|: .: |:
CDC23_YEAST      598 YDYAMGVSSGT-SQ------EI-EEARMLARE-CR-RH    625

--------------------------------------------
Name 1: PANK2_HUMAN
Name 2: P91029_CAEEL

Pantothenate kinase 2, mitochondrial OS=Homo sapiens GN=PANK2 PE=1 SV=3
Pantothenate kinase protein 1, isoform a, confirmed by transcript evidence OS=Ca
enorhabditis elegans GN=pnk-1 PE=2 SV=2

Needed for following interaction(s):
worm    genetic RASK_HUMAN      PANK2_HUMAN     KRAS    PANK2   LET60_CAEEL
P91029_CAEEL

Length sequence 1:      570
Length sequence 2:      449
Sequence #1: PANK2_HUMAN
Sequence #2: P91029_CAEEL
Length #1: 604
Length #2: 604
Matrix: BLOSUM62
Gap open: 2.0
Gap extend: 1.0
Length: 604
Identity: 209/604 (34.60%)
Similarity: 290/604 (48.01%)
Gaps: 214/604 (35.43%)
Score: 827.00

PANK2_HUMAN       22 GLHRLLFLRGTRIPSSTTLSPPRHDSLSLDGGTVNPPRVREPTGREAFGP     71
                     | |       |.:.||..:| ||        |  :||.:|. |      |
P91029_CAEEL       2 G-H-------TSVTSSRRIS-PR--------G--SPPNLRR-T------P     25

PANK2_HUMAN       72 SPASSDWLPARWR----NGRGGRPRARLCSGWTAA-EEARRNPTLGGLLG    116
                      |  .  :  | :       |  ..||       || |:  :      ::
P91029_CAEEL      26 -P--K--I--R-KVLVL--R--EKRA-------AATED--Q------IM-     47

PANK2_HUMAN      117 RQRLLLRMGGGRLGAPMERHGRA-SAT-SVSSAGEQ--AAGD--PEGRRQ    160
                      ..|||   ..:|     :  |. ||| |||   .:  || |  |   ..
P91029_CAEEL      48 -DDLLL---SSKL-----K--RVDSATGSVS---PRVCAA-DALP---TT     79
```

```
PANK2_HUMAN      161 EPLRRRASSASVPAVGASAEGTRR-DRLGSY--SGPTSVSRQRVESLRK-    206
                     .|    |  ||. |:   .: |:: .:. .|  . |.|   ...|..||
P91029_CAEEL      80 TP----A--AST-AL---RQ-TQKAPKT-PYVIL-PAS---DTFEKFRKT    113

PANK2_HUMAN      207 KRPLFPWFGLDIGGTLVKLVYFEPKDITAEEEEEEVESLKS-IRKYLTSN    255
                     .| :    .:||||||:|:||   ..: .:||..| |||.. .|||
P91029_CAEEL     114 SR-V----SVDIGGTLIKVVY---SSV-MDEELPE-ESLNGHTRKY----    149

PANK2_HUMAN      256 VAYGSTGIRDVHLELKDLTLCGRKGNL-HFIRFPTHDMPAFI----QMGR    300
                      ||          |  |    |:: .| :|.:| | ||..||    ::..
P91029_CAEEL     150 -AY----------E--D----GKR-VLVNFKKF-T-DMDRFINFLKEVWT    179

PANK2_HUMAN      301 D-KNFSS-LHTVFCATGGGAYKF-E---QDFLTIG-DLQLCKLDELDCLI    343
                     | |. .. :|   | ||||:||: |   :: |  | .||  :.||:..||
P91029_CAEEL     180 DRKR-GDVIH---C-TGGGSYKYSEIIMKE-L--GVRLQ--RTDEMRSLI    219

PANK2_HUMAN      344 KGI--LY---ID-SVGFNGRSQCYYFENPA-DSEKCQ-KLPF--DLKNPY    383
                     .|:  |.   :| |  |   :  |:.:  | ...|.| : |.  ||  .|
P91029_CAEEL     220 FGVNFLLSTNVDES--F---T--YHHD--AIGRNKFQYR-PIAADL--IY    257

PANK2_HUMAN      384 PLLLVNIGSGVSILAVYSKDNYKRVTGTSLGGGTFFGLCCLLTGCTTFEE    433
                     |.||||||:|:|||.|.|..:|:||.|:|:|||||.||..|||....|:|
P91029_CAEEL     258 PFLLVNIGTGISILKVDSPTSYERVGGSSMGGGTFMGLGSLLTPAQNFDE    307

PANK2_HUMAN      434 ALEMASRGDSTKVDKLVRDIYGGDYERFGLPGWA--VASSFGNMMSK---    478
                     .||||:|||...|||||.|||||.|:..||.  |  :|   |: |:|
P91029_CAEEL     308 LLEMANRGDHRNVDKLVCDIYGGAYDELGLK--ADLIA---GS-MAKCNR    351

PANK2_HUMAN      479 --E--KREAVSK-EDLARATLITITNNIGSIARMCA-L--NE-NINQVVF    519
                       |  |::. .| ||:|::.|:.::||||   :| | |  .. |:.::.|
P91029_CAEEL     352 FEETTKKQQ-HKPEDIAKSLLLMVSNNIG---QM-AYLYGTRYNLKRIYF    396

PANK2_HUMAN      520 VGN-FLRINTIAMRLLAYALDYWSKGQLKALFSEHEGYFGAVGALL-E--    565
                      |. |:|.:.|.||.|::|::|||||:::||:.:||||.||:|:.| |
P91029_CAEEL     397 -GGYFIRQDPITMRTLSFAINYWSKGEIEALYLKHEGYLGAMGSFLDEDG    445

PANK2_HUMAN      566 LLKI    569
                       |:
P91029_CAEEL     446 --KL    447

-------------------------------------------
Name 1: H15_HUMAN
Name 2: H1_YEAST

Histone H1.5 OS=Homo sapiens GN=HIST1H1B PE=1 SV=3
Histone H1 OS=Saccharomyces cerevisiae (strain ATCC 204508 / S288c) GN=HHO1 PE=1
 SV=1

Needed for following interaction(s):
yeast   physical        H15_HUMAN       MUS81_HUMAN     HIST1H1B        MUS81
H1_YEAST        MUS81_YEAST

Length sequence 1:      226
Length sequence 2:      258
Sequence #1: H15_HUMAN
Sequence #2: H1_YEAST
Length #1: 281
Length #2: 281
Matrix: BLOSUM62
Gap open: 2.0
Gap extend: 1.0
Length: 281
Identity: 99/281 (35.23%)
Similarity: 130/281 (46.26%)
Gaps: 81/281 (28.83%)
Score: 344.00

H15_HUMAN          1 MSETAP--AETATPAPVE-KSPA--K-K-KATKKAA-GAGAAKR-KATGP     41
                     |   ||  :.|.|.:  : |.||  | | |:|.||| ....||: :|:..
H1_YEAST           1 M---APKKSTTKTTS--KGKKPATSKGKEKSTSKAAIKKTTAKKEEASSK     45

H15_HUMAN         42 PVS--ELITKAVAASKERNGLSLAALKK------AL--AAGGYDVEKNNS     81
                       |  |||.:.:.|.|||.|.|..||||      .:  :|..:|:..||:
H1_YEAST          46 --SYRELIIEGLTALKERKGSSRPALKKFIKENYPIVGSASNFDLYFNNA     93

H15_HUMAN         82 RIKLGLKSLVSKGTLVQTKGTGASGSFKLNKKAASGEAKPKAKKAGAAKA    131
                      ||   |. |..|...|.||. | |:.||.|| .|.|.| |.|:. :.|.
H1_YEAST          94 -IK---KG-VEAGDFEQPKGP-A-GAVKLAKK-KSPEVK-KEKEV-SPKP    133

H15_HUMAN        132 KKPA---GATPKKAKKAAG---A-KKAV-KKTP----KKAKKPAAAGVKK    169
                     |:.|   .||..|| |||.   | ||.| ||:|    |||..|::...|:
H1_YEAST         134 KQAATSVSATASKA-KAASTKLAPKKVVKKKSPTVTAKKASSPSSLTYKE    182

H15_HUMAN        170 -VAKS-PK----K--AKAAAKPK--KATKSPAKPK-----------AVKP    198
                      :.|| |:    |  ::...| |  |.|.| :|.|           |:|
H1_YEAST         183 MILKSMPQLNDGKGSSRIVLK-KYVKDTFS-SKLKTSSNFDYLFNSAIK-    229

H15_HUMAN        199 KAAK-PKAAKPKAAKPKA-AKAKKAAAKK-K    226
                     |..: .:..:||.  |.. .|..|   || |
H1_YEAST         230 KCVENGELVQPKG--PSGIIKLNK---KKVK    255

-------------------------------------------
Name 1: UBP10_HUMAN
Name 2: UBP3_YEAST

Ubiquitin carboxyl-terminal hydrolase 10 OS=Homo sapiens GN=USP10 PE=1 SV=2
Ubiquitin carboxyl-terminal hydrolase 3 OS=Saccharomyces cerevisiae (strain ATCC
 204508 / S288c) GN=UBP3 PE=1 SV=1

Needed for following interaction(s):
yeast   genetic RASK_HUMAN      UBP10_HUMAN     KRAS    USP10   IRA2_YEAST
UBP3_YEAST

Length sequence 1:      798
Length sequence 2:      912
Sequence #1: UBP10_HUMAN
Sequence #2: UBP3_YEAST
Length #1: 1031
Length #2: 1031
Matrix: BLOSUM62
Gap open: 2.0
Gap extend: 1.0
Length: 1031
Identity: 302/1031 (29.29%)
Similarity: 452/1031 (43.84%)
Gaps: 357/1031 (34.63%)
Score: 1100.00

UBP10_HUMAN        1 MALHSPQYIFGDFSPDE-FNQFFVTPR-SSVELPPYSGTVLCGTQAVDKL     48
                     |.:   |    |.:.:| ::.:   |: ||   || ..|   .|. : ::
UBP3_YEAST         1 MNM---Q----DANKEESYSMY---PKTSS---PP-PPT---PTN-M-QI     31

UBP10_HUMAN       49 PDGQEYQR-IE-FGVDEVIEPSDTL-PRT--PSYS---ISSTLN-P---Q     86
                     |   .||. :: :|..:.  |  .| | |  |:||   ::.  | |   |
UBP3_YEAST        32 P---IYQAPLQMYGYTQA--P--YLYP-TQIPAYSFNMVNQ--NQPIYHQ     71

UBP10_HUMAN       87 --APEFI-----L--GCTA--SKIT-----PDGIT-KEASYGSIDCQ---    116
                       :|..:     :  |.|.  :.|.     .:||| ...|.|:   |
UBP3_YEAST        72 SGSPHHLPPQNNINGGSTTNNNNINKKKWHSNGITNNNGSSGN---QGAN    118

UBP10_HUMAN      117 YPGSALALDGS-------SN--VE--AEVLENDGVS-GGLGQRE-RKK--    151
                     ..||.::.:.|       ||  :.  |.  .|.| | .|: ::: ...
UBP3_YEAST       119 SSGSGMSYNKSHTYHHNYSNNHIPMMAS--PNSG-SNAGM-KKQTNSSNG    164

UBP10_HUMAN      152 --KKKRPPGY--Y---S-Y-L-K-DGGDDSISTEALVNGHANSA--V--P    186
                       .....|.|  |   | | | | |     : |: |.|...||:  :  |
UBP3_YEAST       165 NGSSATSPSYSSYNSSSQYDLYKFD-----V-TK-LKNLKENSSNLIQLP    207

UBP10_HUMAN      187 ---NSVSAEDAEFMGDMPPSVTPR--------TCNSP--QNSTDSV--SD    221
                        |  :.| |||..   .|| .|        ..||.  :||  ||  |.
UBP3_YEAST       208 LFIN--TTE-AEFAA---ASV-QRYELNMKALNLNSESLENS--SVEKSS    248

UBP10_HUMAN      222 I---VPD-SPFPGALGSD--TRTAGQPEGGPGA-DFGQSCFPAEAGRDT-    263
                     .   ... | .| ....:  |.|.|:.|   .| |  :.  | .|.:|.
UBP3_YEAST       249 AHHHTKSHS-IP-KHNEEVKTETHGEEE---DAHD--KK--P-HASKDAH    288

UBP10_HUMAN      264 -L-SRTAGAQPCV-GTDTTENLGVANGQILESSGEGTATNGVELHTTESI    310
```

```
                       | .:|    :  | ..|..::   .|.::::   |..||  | |     .:
UBP3_YEAST       289 ELKKKT---E--VKKEDAKQD---RNEKVIQ---EPQAT--V-L----PV    320

UBP10_HUMAN      311 DLDPTK-P-ESASPPADGTGSASGTLPVSQP--KSWASLFHD---SKPSS    353
                      :| .| | ||..   :.|...|...| |.|  |||:::..|    |:.:|
UBP3_YEAST       321 -VD-KKEPEESVE---ENTSKTSSPSP-SPPAAKSWSAIASDAIKSRQAS    364

UBP10_HUMAN      354 ----S-S-----PV----AYV-ETKYSPPA-I----SPLVSEKQVEVKE-    382
                         | |      |:    |.| .|..: .| |    |||:| ||.:.|:
UBP3_YEAST       365 NKTVSGSMVTKTPISGTTAGVSSTNMA-AATIGKSSSPLLS-KQPQKKDK    412

UBP10_HUMAN      383 GLVPVS-E--DP---VAIK-------IAELLEN--VTLIHK-PV-SLQPR    415
                     ..||.| :  :|   :|::        |:.:|.|  |.  :| || |:.||
UBP3_YEAST       413 KYVPPSTKGIEPLGSIALRMCFDPDFISYVLRNKDVE--NKIPVHSIIPR    460

UBP10_HUMAN      416 GLINKGNWCYINATLQALVACPPMYHLMKFIPLYS-K-V-QRPCTST-PM    461
                     |:||:.|.|::::.||.|:.|.|...::. : | | : . .|..||: .:
UBP3_YEAST       461 GIINRANICFMSSVLQVLLYCKPFIDVIN-V-L-STRNTNSRVGTSSCKL    507

UBP10_HUMAN      462 IDSFVRL-M-NEFTNMPVPPKPR--Q-ALGD--KIVR-DIRPGA---AFE    500
                     :|: . | | .:| :.....| :  : | .|  |... |.:..:   :|:
UBP3_YEAST       508 LDA-C-LTMYKQF-DKETYEK-KFLENA-DDAEKTTESDAKKSSKSKSFQ    552

UBP10_HUMAN      501 ---------P-TYIYRLL-TVNK-SSLSEKGRQEDAEEYLGFILNGLHEE    538
                              | .: |:.| |:.| ..| :.|.||||||:|..:|:.||||
UBP3_YEAST       553 HCATADAVKPDEF-YKTLSTIPKFKDL-QWGHQEDAEEFLTHLLDQLHEE    600

UBP10_HUMAN      539 M------L--N-LKKLLSPS-N-EKL-T--ISN----GP----KNHSVNE    566
                     :      |  | ::.:|. | | |:| .  |.|     |.    ||.|...
UBP3_YEAST       601 LISAIDGLTDNEIQNMLQ-SINDEQLKVFFIRNLSRYGKAEFIKNASPRL    649

UBP10_HUMAN      567 EEQ-EEQG---EGS-EDE-WEQV-G--PR-NKTSVT---RQADFVQTPIT    603
                     :|. |:.|   :.| |:. |.:| |  .| .||. |   |..:.|.:||:
UBP3_YEAST       650 KELIEKYGVINDDSTEENGWHEVSGSSKRGKKTK-TAAKRTVEIVPSPIS    698

UBP10_HUMAN      604 GIFGGHIRSV--VYQQSSKES-A-TLQPFFTLQLDIQSDKIRTVQDALES    649
                     .:|||..|||  :  .::||| : ||.||.|:|||| ||  ..|.| ||:
UBP3_YEAST       699 KLFGGQFRSVLDI--PNNKESQSITLDPFQTIQLDI-SD--AGVND-LET    742

UBP10_HUMAN      650 LVA-RE-SVQGYTT---KTK--QEVEISRRVT-LEKLPPVLVLHLKR--F    689
                       | :: | : |..   |:.  .:|| :::.| ::|||.||::..||  |
UBP3_YEAST       743 --AFKKFS-E-YELLPFKSSSGNDVE-AKKQTFIDKLPQVLLIQFKRFSF    787

UBP10_HUMAN      690 V--YEK----T-----GG-CQKLI-KNIEYPVDLEISKELLSPGV--KNK    724
                     :  ..|     |      .| .:| | |.|:|..:|.|.:|.:| .:  ||
UBP3_YEAST       788 INNVNKDNAMTNYNAYNGRIEK-IRKKIKYGHELIIPEESMS-SITLKN-    834

UBP10_HUMAN      725 N---FKCHRTYRLFAVVYHHGNSATGGHYTTDVFQIGLNGWLRIDDQTVK    771
                     |   .. .|.|:|..|:||||.|:.|||||.||:....|.|.||||  |.
UBP3_YEAST       835 NTSGID-DRRYKLTGVIYHHGVSSDGGHYTADVYHSEHNKWYRIDD--VN    881

UBP10_HUMAN      772 VINQYQ---VVK--PTA-E-RTAY-LLYYRR    794
                       |.:.:   |:|  ..| : |||| |:|.:|
UBP3_YEAST       882 -ITELEDDDVLKGGEEASDSRTAYILMYQKR    911

--------------------------------------------

Name 1: GCN1L_HUMAN
Name 2: GCN1_YEAST

Translational activator GCN1 OS=Homo sapiens GN=GCN1L1 PE=1 SV=6
Translational activator GCN1 OS=Saccharomyces cerevisiae (strain ATCC 204508 / S
288c) GN=GCN1 PE=1 SV=1

Needed for following interaction(s):
yeast   genetic GCN1L_HUMAN     RASK_HUMAN      GCN1L1  KRAS    GCN1_YEAST
IRA2_YEAST

Length sequence 1:      2671
Length sequence 2:      2672
Sequence #1: GCN1L_HUMAN
Sequence #2: GCN1_YEAST
Length #1: 3101
Length #2: 3101
Matrix: BLOSUM62
```

```
Gap open: 2.0
Gap extend: 1.0
Length: 3101
Identity: 1023/3101 (32.99%)
Similarity: 1618/3101 (52.18%)
Gaps: 880/3101 (28.38%)
Score: 4239.00

GCN1L_HUMAN        1 MAA-----DTQVSETLKRFAGKVTTAS-VKER----REILSELGKCVAGK     40
                     |.|      |  :|..|::  |  |..| |.:|     ::| |:|   |. :
GCN1_YEAST         1 MTAILNWED--ISPVLEK--G--TRESHVSKRVPFLQDI-SQL---VR-Q     39

GCN1L_HUMAN       41 D-L--P---EGA-VKGLCKLFCLTLHRYRDAASR-RALQAAIQQLAE---     79
                     : |  |   |.| |  |...|  |:  |.|  :| ::|..:|  |.:
GCN1_YEAST        40 ETLEKPQLSEIAFV--LLNTF--TI--YED--NRSKSLVTSI--LLDILN     79

GCN1L_HUMAN       80 AQPEATAKNLLHSLQSS-GIGSKAGVP-SKSSGSAA--L-LALTWTCLLV    124
                     .:| ...:|.:.: |. .| |.   | :|   :.|  | | |.|     :
GCN1_YEAST        80 LEP-CLLENFIRFI-SDVVI-SN---PATK---AVADYLNL-LDW----I    115

GCN1L_HUMAN      125 R--IVFPSRAKRQGDIWNKLVE--V-QCLLL--------LE-VL-G---G    156
                     .  ::|.|     ...  | |.|  : : ||:          :| :| .    |
GCN1_YEAST       116 NSFLIFVS----HNS--N-LFEEYIPK-LLVAHSYATFGVETILDNQEEG    157

GCN1L_HUMAN      157 --S------H-K---HAV-DGAVKK-LTK-LWKENPGLVEQYLS-AILS-    189
                       |       | |   :.: ...||. | | | |:|...: .::. :|.:
GCN1_YEAST       158 KKSQDKQNQHRKRIRYCIFQTTVKAFL-KCL-KDNDDSI-SFMKISIKTV    204

GCN1L_HUMAN      190 LEPNQNYA-------G--M-LGLLVQFCTSHKEMDVVS-Q---H---K--    220
                     ||   :|:         |  | :|.|.|  .:   :.::| |    |    |
GCN1_YEAST       205 LE---SYSKLKITSVGVVMIMGALTQ--AA---LQLLSRQPALHSVLKEN    246

GCN1L_HUMAN      221 SALLD-F--YM-KNILMSKVKPP-KYLLD-SCAPLLR-YLSHSE-F-KDL    261
                     ||  : :   |: |.:.:.| .|| .:.|: ...|.|: ::| .| | | .
GCN1_YEAST       247 SA--EKYCEYLGKEVFLGK-NPPSSFCLEIGLKPFLKEFVS-QELFIK-F    291

GCN1L_HUMAN      262 ILPTIQKSLLRSPE---NVI-E--T-IS----SLL---ASVTLDLSQY--    295
                     .:|.|:|::|||||   ::: |  . :|     :||   ||..| ::||
GCN1_YEAST       292 FIPNIEKAVLRSPEVGFSILSELYAGVSPEKVNLLNAFASSKL-INQYFS    340

GCN1L_HUMAN      296 ----AMDIVKGLAGHLKSNSPRLMDEAVLALRNLARQCSDSSAMESLTK-    340
                         :.::|:.::  |:|      |  .:| ||.::.  :|:: :|.|||
GCN1_YEAST       341 SFKSSKEVVRSVS--LQS-----M--IIL-LRKISN--TDTT-LEDLTKL    377

GCN1L_HUMAN      341 --HLFA-I---LGGSEGKLTVVAQKMSVLSGIGSV--SHHVVSGPSSQVL    382
                       .:|. |    | .::.| :::: |  :|  | .:  :|:.|    |.::.
GCN1_YEAST       378 IDEIFKNIKSNL-NADYK-SLIS-K--IL--I-EIPLTHYEV---SEKIC    416

GCN1L_HUMAN      383 NGIVAELFIPFLQQEVHEG--TL------VHAVSV---LA-LWCNR----    416
                      .|: :     |::.:|.:|.  ||       ||..|:    :. |  ::
GCN1_YEAST       417 KGL-S----PYIGKEGNEAALTLMLNAFFVHYFSLGKPIEDL--DKIISA    459

GCN1L_HUMAN      417 -FTMEVP--KKLTEWFKKAFSLKTSTSAVRHAYLQCMLASYRGDTLLQAL    463
                       |..:.|  ||.  || .|| |  :.|    :|        || . :.:|..:
GCN1_YEAST       460 GFADKKPALKKC--WF-AAF-L--NNS---NA------AS-E-EVILNFI    492

GCN1L_HUMAN      464 DLLPLLIQTVEKAASQSTQVPTI---TEGVAAALL--L-L--KLSVA-DS    504
                     |  ..| :.| |    .|  :  |    |.| .|.:|  : .  |: :| |:
GCN1_YEAST       493 D--GCL-EFV-K---DS--I--IHYQTHG-HACILASIEFTNKI-LALDN    529

GCN1L_HUMAN      505 QAEAKLSS-FWQLIVDEKKQVFT-SEKFLVMAS-EDALCT-VLHLTERLF    550
                      .|  |:. ..|||  |      | .|.    :| .||:.| .|. || |.
GCN1_YEAST       530 -TE--LNDRVMQLI--E-----TLPEN----SSIGDAILTAALS-TE-LS    563

GCN1L_HUMAN      551 LDHP-HRLTGNKVQQ--YHR-ALV--AVL--LSRTWHVR-R-Q---AQQ-    586
                     :::. |.:  |.:|:  |.:  ..:  :|:  :.|    | | |   .||
GCN1_YEAST       564 IENRIHAV--NLLQELFYKKPEFIGFSVIDAIER----RMRVQELIPQQN    607

GCN1L_HUMAN      587 TVRKLLSSLGGFKLAHGLLEELKTVLSSHKVLPLEALVTDAGEVTEAGKA    636
                     |       |   ||  :          | :|  || | | :|      :|
GCN1_YEAST       608 T------S---FK--Y--------V-TS--VL-L-A-IT-----SE----    623

GCN1L_HUMAN      637 YVPPRVLQEALCVISGVPGLKGDVTDTEQL--AQEMLIISHHPSLVAVQS    684
                      :|.:   ||     |     :|  |      |  |   |:|:.. ::.:::
```

```
GCN1_YEAST        624 -LPDK---EA----S----IK--V-----LINA---LVIAQW-NIFNIKN    650

GCN1L_HUMAN       685 GLWPA-LLARMKIDP-EAFITRH----LDQIIPRMTTQS-P-LNQSSM-N    725
                      | | | |:.|.::|| |. :..|     :::|: .: |.| . ::  :: .
GCN1_YEAST        651 G-W-AGLVLRARLDPAEV-VKEHASVIMEKIL-EI-TGSCEWID--TIYG    693

GCN1L_HUMAN       726 AMGSL------SVLSPDRVLPQLISTITASVQNPA--L-RLVTREEFAIM    766
                      |.| |       :.:.|:...|.|..||.|.:  .|  . || :.|:|.|.
GCN1_YEAST        694 ACG-LQAAAYAAFIQPNEFTPILCKTIEADL--TADDFSRL-SEEDFEIF    739

GCN1L_HUMAN       767 QTPAGE---L-YDKSIIQSAQQDSI-KK-ANMKREN-KAYSFKEQII-EL    808
                          |||    | .|  ::     ::|: || :| |  | |.|   |.:: |.
GCN1_YEAST        740 ---AGEEGVLVVD--VL----EESMNKKLSN-K--NSKEY---ETLMWEQ    774

GCN1L_HUMAN       809 EL-KEEIKK--KKGI-KEEVQ-LTSKQ--KE-MLQAQLDREAQVR-RR-L    848
                      :: ||:.||  || : ||| | |.::|  || .::::.:. |...| :| :
GCN1_YEAST        775 KIRKEQAKKNVKK-LSKEE-QELVNEQLAKESAVRSHVS-EISTRLKRGI    821

GCN1L_HUMAN       849 QELDGEL-EAALGLLDIILAKNPSGL-TQYIPVLVDSFLPL-LKS-P-LA    893
                      : |..|| :||  .|  :  :|  |: | :.|:.|.. | |.| | ::
GCN1_YEAST        822 R-LVSELSKAA--CL--V--QN--GIAT-WFPLAVTK-L-LYLCSEPNIS    859

GCN1L_HUMAN       894 --APRIKNPFLSLAACVMPS-RL---KA-LGTLVSHVTLRLLKPECVLDK    936
                        ...:.|.||.|:..|  | ||    :. || |    .|||:.....: .:
GCN1_YEAST        860 KLTEDVNNVFLQLSQNV--SERLGNIRLFLG-L---ATLRVHNANGI-SQ    902

GCN1L_HUMAN       937 SWCQEELSVAVKRAVMLLHTHTITSRVGK---GEPGAAPLSAPAFSL--V    981
                      .:.:||.|   |:     || |. :..|: |    .:   || :.  :.|| :
GCN1_YEAST        903 DYLQEPL---VE----LL-TR-VLFRI-KFVSNQ--AA-ID--SISLTYI    937

GCN1L_HUMAN       982 FPFL-------KMV-L--TEMP----HH-SE-EEEE--WMAQILQILTVQ   1013
                      .|.|         |.: |  .:.|     .. .| ||||  .:|  ::|::|.
GCN1_YEAST        938 LPLLINVLEKGKAIALKNADKPVVKAEFVEEDEEEEHLLLA--MEIISVH    985

GCN1L_HUMAN      1014 AQLRASPNTPPGRVDENGPELLPRVAMLR-LLTWVIGTGS-P-RLQVLAS   1060
                      |:  |        . |: |. :||::::. ||: ::   | | :.:: |.
GCN1_YEAST        986 AE--A--------F-ED-PS-IPRISIVEVLLS-LL---SLPSKAKI-AK   1017

GCN1L_HUMAN      1061 DTLTTLCASSSGDDGCAFAEQEEVDVLLCALQSPCAS--VRETVLRGLME   1108
                      |....||.|.|     .| ..||::|::|..|.||  :  ||.|:|     |
GCN1_YEAST       1018 DCFNALCQSIS----VA-PNQEDLDMILSNLLSP--NQFVRSTIL----E   1056

GCN1L_HUMAN      1109 LHMVLPAPDTD-EKNGLN-LLR---RLWVVKFDKEEEIRKLAERLW--S-   1150
                         .|   |.: |   |. .::    .:::.:||.:...|::|:.:|  :
GCN1_YEAST       1057 ---TL---DNEFE---LEPFMKYSPEVFICRFDSDPSNREIADFIWEFNK   1097

GCN1L_HUMAN      1151 -MMGLD-LQPDLCSL--LI--DD--V-IYHEAAVRQA-GAEAL--SQAVA   1188
                        ::. | |   |.||  |.   ||  : ::  ||...| ||.:|  |:
GCN1_YEAST       1098 FVVN-DEL---LKSLFPLFNQDDSGLRLF--AANAYAFGAVSLFTSE---   1138

GCN1L_HUMAN      1189 RYQRQAAEVMGRLMEIYQEKLYRP-PPVLDALGRV-ISES-PPDQWEARC   1235
                         :..:.:.:..|:..|:|. :| .|:||..|.| :|.| ..|.|:.|.
GCN1_YEAST       1139 --ENSSKDYLNDLLNFYKEKA-KPLEPILDQFGLVLVSASEQKDPWQGRS   1185

GCN1L_HUMAN      1236 GLALALNK-LSQ-Y-L-DSSQVKPLFQFFVPD-ALNDRHPDVRKCMLDAA   1280
                      .:|:.| | ::: : . |.:.|. :.:|.|.| .|.||.|.||:.|.:|.
GCN1_YEAST       1186 TVAITL-KIMAKAFSAEDDTVVN-IIKFLVDDGGLVDREPIVRQEMKEAG   1233

GCN1L_HUMAN      1281 --LATLNTHGKENVNSLLPVFEEFLKNAPNDASYDAVRQSVVVLMGSLAK   1328
                        |.||  ||.:|...|:|:|||.|.:: .| |  |::::|::|.|:||:
GCN1_YEAST       1234 VELITL--HGSQNSKDLIPIFEEALSSS-TD-S--ALKENVIILYGTLAR   1277

GCN1L_HUMAN      1329 HLDKSDPKVKPIVAKLIAALSTPSQQVQESVASCLPPLVPAIKEDAG---   1375
                      ||.:||.::..|:.:|::.|.|||..:|::|::|:.|||...|:..|
GCN1_YEAST       1278 HLQQSDARIHTIIERLLSTLDTPSADIQQAVSACIAPLVFQFKQKVGDYL   1327

GCN1L_HUMAN      1376 GMIQRLMQQLLESDKYAE--RKGAAYGLAGLVKGLGILS-LKQQEMMAAL   1422
                      | |  ||::|| :...|.  |||||:|:||||||.|| | |.:.:::..|
GCN1_YEAST       1328 G-I--LMEKLL-NPTVASSMRKGAAWGIAGLVKGYGI-SALSEFDIIRNL   1372

GCN1L_HUMAN      1423 TDAIQDKKNFRRRE--GALFAFEMLCTMLGKLFEPYVVHVLPHLL--LCF   1468
                      .:|.:|||..:|||  |  |.|:.|...|||.|||||:.:||::|  |
GCN1_YEAST       1373 IEAAEDKKEPKRRESVG--FCFQYLSESLGKFFEPYVIEILPNILKNL--   1418
```

```
GCN1L_HUMAN      1469 GDG-NQYVREA-ADDCA-KAVMSNLSAHGV-KLVLPSLLA-A-LEEESWR   1512
                      ||. .: ||:| |.  | ||:|::.:.:|| ||: |  :| : |:|.:||
GCN1_YEAST       1419 GDAVPE-VRDATAR--ATKAIMAHTTGYGVKKLI-P--VAVSNLDEIAWR   1462

GCN1L_HUMAN      1513 TKAGSVELLGAMAYCAPKQLSSCLPNIVPKLTEVLTDSHVK-VQKAGQQA   1561
                      ||.|||:|||.|||..|.|||:.|..|||::..||.||| | |:||..::
GCN1_YEAST       1463 TKRGSVQLLGNMAYLDPTQLSASLSTIVPEIVGVLNDSH-KEVRKAADES   1511

GCN1L_HUMAN      1562 LRQIGSVIRNPEI--LAIAPVLLDALTDPSRK-TQKCLQTLLDTKFVHFI   1608
                      |::.|.||||||| | : ||||.|:.||: | |::.|.:|:.|:|||:|
GCN1_YEAST       1512 LKRFGEVIRNPEIQKL-V-PVLLQAIGDPT-KYTEEALDSLIQTQFVHYI   1558

GCN1L_HUMAN      1609 DAPSLALIMPIVQRAFQDRSTD-TRKMAAQIIGNMYS-LTDQKDLAPYLP   1656
                      |.|||||:.|:.|...|||.: .|| |.:|:||| : |.|.|||.|||.
GCN1_YEAST       1559 DGPSLALIIHIIHRGMHDRSANIKRK-ACKIVGNM-AILVDTKDLIPYLQ   1606

GCN1L_HUMAN      1657 SVTPGLKASLLDPVPEVR-TVSAKALGAMVKGMGESCFEDLLPWLMETLT   1705
                      .:...::.:::||||..| | :|:||||:|:.:||..|.||:|.|:::||:
GCN1_YEAST       1607 QLIDEVEIAMVDPVPNTRAT-AARALGALVERLGEEQFPDLIPRLLDTLS   1655

GCN1L_HUMAN      1706 YEQS-SVDRSGAAQGLAEVMAGLGVEKLEKLMPEIVA--TAS-KVDIAPH   1751
                       ::| |.||.|:||.||||::|||:.||::::|.|:|  | : :   | :
GCN1_YEAST       1656 -DESKSGDRLGSAQALAEVISGLGLTKLDEMLPTILAGVT-NFR---A-Y   1699

GCN1L_HUMAN      1752 VRDGY--IMMFNYLPITFGDKFTPYVGPII-PCILKALA--DENEFVRDT   1796
                      :|:|:  :::|  ||:.||.:|.||:..|| | ||..||  |||  :|||
GCN1_YEAST       1700 IREGFMPLLLF--LPVCFGSQFAPYINQIIQP-ILSGLADNDEN--IRDT   1744

GCN1L_HUMAN      1797 ALRAGQRVISMYAET-AIALLLPQLEQGLFD--DLWRIRFSSVQLLGDLL   1843
                      ||:||:.::..|| | |:.||||:||:|:|| |  |||.|||||.|:|||
GCN1_YEAST       1745 ALKAGKLIVKNYA-TKAVDLLLPELERGMFDEND--RIRLSSVQLTGELL   1791

GCN1L_HUMAN      1844 FHISGVTGKMTTETASED-D-NFGTAQ-SNKAIITALGVERRNRVLAGLY   1890
                      |.::|::.:  .|.:.|| | | |  : |.| ::.:.||.:||:|:||.|:
GCN1_YEAST       1792 FQVTGISSR--NEFSEEDGDHN-G--EFSGK-LVDVLGQDRRDRILAALF   1835

GCN1L_HUMAN      1891 MGRSDTQLVVRQA-SLHVWKIVVSNTPRTLREILPTLFGLLLGFLASTCA   1939
                      :.|:||..:|| | ::.:||.:|.||||.::||||||.|:::..|||: :
GCN1_YEAST       1836 VCRNDTSGIVR-ATTVDIWKALVPNTPRAVKEILPTLTGMIVTHLASS-S   1883

GCN1L_HUMAN      1940 D-KRTIAARTLGDLVRKLGEKILPEIIPILEEGL-RSQKSDERQGVCIGL   1987
                      : .|.|||:|||||||::|...|.:::|.|||.| .:..||.||||||.|
GCN1_YEAST       1884 NVLRNIAAQTLGDLVRRVGGNALSQLLPSLEESLIETSNSDSRQGVCIAL   1933

GCN1L_HUMAN      1988 SEIMKSTSRDAVLYFSES-LVPTARKALCDPLEE--VREAAAKTFE--QL   2032
                      .|:::|.|.:.:..| :| :|...|.||.| |.  ||||||.:|:  |
GCN1_YEAST       1934 YELIESASTETISQF-QSTIVNIIRTALID--ESATVREAAALSFDVFQ-   1979

GCN1L_HUMAN      2033 HSTIGHQALEDILPFLLKQLD--DEEVSEFALDGLKQVMAIKSRVVLPYL   2080
                         ..:|..|::::||:||..|:  |.  |:|||.||:::|:.||.|:.|.|
GCN1_YEAST       1980 -DVVGKTAVDEVLPYLLHMLESSDN--SDFALLGLQEIMSKKSDVIFPIL   2026

GCN1L_HUMAN      2081 VPKLTTPPVNT-R--VLAFLSSVAGDALTRHLGVILPAVML-ALKEKLGT   2126
                      :|.|..||::. |   .|..|:.|||.||.:.:.|:.:|:.| :: |:   :||
GCN1_YEAST       2027 IPTLLAPPIDAFRASALGSLAEVAGSALYKRLSIIINA-LVDAI---IGT   2072

GCN1L_HUMAN      2127 -PDE--Q--LEMANCQAVILSVEDDTG-HRIIIEYLLEATRSPEVGMRQA   2170
                        .||  :  ||:| ...|.|||.||.| |. :::.::...:|..:..|
GCN1_YEAST       2073 SEDESTKGALELA-LDRVFLSVNDDEGLHP-LLQQIMSLLKSDNIEKR--   2118

GCN1L_HUMAN      2171 AAII--L-NIYCSRSKADYTSHLRSLVS-GLIRLFNDSSP-VVLEESWDA   2215
                      .|::  | |.: .::..|:..::.:.:|| .::.| :|...  || ..:::|
GCN1_YEAST       2119 IAVLERLPNFF-DKTVLDFDVYIPNFVSHAILSL-DDEDQRVV-NGNFNA   2165

GCN1L_HUMAN      2216 LNAITKKLDAGNQLALIEEL---HKE-IRLIGNESKGEHVPGFCLPKKGV   2261
                      |:.:.||:|   :...|   .|: :.|.|.:  |:.|..|.|| :|.
GCN1_YEAST       2166 LSTLLKKVD---KPTL-EKLVKPAKQSLALTGRQ--GQDVAAFKLP-RGP   2208

GCN1L_HUMAN      2262 TSILPV-LREGVLTGSPEQKEEAAKALG-LVIRLTSADA-LRPSVVS-IT   2307
                      ..:||: | .|::.||.:::||:|.|:. :|.: |.| | |: |:|| ||
GCN1_YEAST       2209 NCVLPIFL-HGLMYGSNDEREESALAIADVVSK-TPA-ANLKP-FVSVIT   2254

GCN1L_HUMAN      2308 GPLIRILGDRFSWNVKAALLETLSLLLAKVGIALKPFLPQLQTTFTKALQ   2357
                      |||||::|:|||.::|||:|..|::|..|:.:.|:||||.||.|:|.
```

```
GCN1_YEAST      2255 GPLIRVVGERFSSDIKAAILFALNVLFIKIPMFLRPFIPQLQRTFVKSLS   2304

GCN1L_HUMAN     2358 D-SNRGVRLKAADALGKLISIH-IKVDPLFTELLNGIR-AMEDPGVRDT-   2403
                     | :|..:||:||.|||.||. | .:||||..||:.|.: | .|.||: |
GCN1_YEAST      2305 DATNETLRLRAAKALGALIE-HQPRVDPLVIELVTGAKQA-TDEGVK-TA   2351

GCN1L_HUMAN     2404 MLQA-LRFVIQGAGAKV--DAVIRK-NIVSLL-LSMLGHDEDNTRISSA-   2447
                     ||:| |. ||..||:|:  ::   | |||:|: ..||| ..|...::.|
GCN1_YEAST      2352 MLKALLE-VIMKAGSKLNENS---KTNIVNLVEEEMLG-SNDKLAVAYAK   2396

GCN1L_HUMAN     2448 --GCLGELCAFLTEEELSA--VLQQCLL-ADVSGIDWMVRHGRSLALSVA   2492
                       |.|.|:   |:.:|  |  :||..:| ||:.| :  .  |: .|: :.
GCN1_YEAST      2397 LIGSLSEI---LSNDE--AHKILQDKVLNADLDG-E--T--GK-FAI-LT   2434

GCN1L_HUMAN     2493 VN--V--APGRLCAGRYSSD-VQE-M--ILSSATADRIP-I--AVSG-V-   2529
                     :|  :  ||..:    :::. :.| :  || :|.  |.| :  ..:| :
GCN1_YEAST      2435 LNSFLKDAPTHI----FNTGLIDEFVSYIL-NAI--RSPDVYFGENGTIA   2477

GCN1L_HUMAN     2530 RG-MGFLM----RH-HIETGGGQLPAKL---S-SLFV----KC-LQNP-S   2563
                     .| : .|:    |. .::....: |.|:   : :|.:    |. || | |
GCN1_YEAST      2478 AGKL-LLLEGEKRSPFVKKDAAE-PFKIGDENINLLINELSKAVLQ-PAS   2524

GCN1L_HUMAN     2564 -S-DIR---LV-----AE-KM---I--WWANKDPL-PP----L-DPQAIK   2591
                      | |:|   ||     |. |.   |  ::   |.: |.    | || .|
GCN1_YEAST      2525 NSTDVRRLALVVIRTLARFKFDECIKQYF---DVVGPSVFSCLRDP-VI-   2569

GCN1L_HUMAN     2592 PILKALLDNTKDKNTVVRAYSDQAIVNLLKMRQGEE---VFQS-LSKILD   2637
                     || |  |  ..:|     ||    :. |.|:.: |:   .|.. .:||.|
GCN1_YEAST      2570 PI-K--L--AAEK-----AY----LA-LFKLVE-EDDMHTFNEWFAKISD   2603

GCN1L_HUMAN     2638 VA-SLE-V------LNEV---NRRSL-KKLASQADSTEQV----D-DTIL   2670
                     .. |:| |      |..|   .:| : |:||: .: .|::    | :|:.
GCN1_YEAST      2604 RGNSIETVTGTTIQLRSVGDYTKR-VGKRLAN-VE-RERIAAGGDAETMF   2650

GCN1L_HUMAN     2671 T   2671
                     :
GCN1_YEAST      2651 S   2651
```

Name 1: KCC1G_HUMAN
Name 2: RAD53_YEAST

Calcium/calmodulin-dependent protein kinase type 1G OS=Homo sapiens GN=CAMK1G PE=1 SV=3
Serine/threonine-protein kinase RAD53 OS=Saccharomyces cerevisiae (strain ATCC 204508 / S288c) GN=RAD53 PE=1 SV=1

Needed for following interaction(s):
yeast   genetic KCC1G_HUMAN   PTTG1_HUMAN   CAMK1G PTTG1   RAD53_YEAST   SECU_YEAST

Length sequence 1:   476
Length sequence 2:   821
Sequence #1: KCC1G_HUMAN
Sequence #2: RAD53_YEAST
Length #1: 660
Length #2: 660
Matrix: BLOSUM62
Gap open: 2.0
Gap extend: 1.0
Length: 660
Identity: 214/660 (32.42%)
Similarity: 305/660 (46.21%)
Gaps: 227/660 (34.39%)
Score: 855.00

```
KCC1G_HUMAN        4 K---EED--D-C-SSWK---K--Q---T--T--N-I-RKT--F-IF--M-     26
                     |   |::  | . |:.|   |  .   |  |  : : .||  | .|  :
RAD53_YEAST      152 KQCLEQNKVDRIRSNLKNTSKIASPGLTSSTASSMVANKTGIFKDFSIID    201

KCC1G_HUMAN       27 EVLGSGAFSEVFLVK--QRLTGKLFALKCIKKSPAF--RDSSLENEIAVL     72
                     ||:|.|||:.|  .|  :|.|||.||:|.|.|....  .| .:..|:.||
RAD53_YEAST      202 EVVGQGAFATV--KKAIERTTGKTFAVKIISKRKVIGNMD-GVTRELEVL    248

KCC1G_HUMAN       73 KKIKHENIVTLEDIYESTTHYYLVMQLVSGGELFDRILERG-VYTEKDAS    121
                     :|:.|..||.|:..||.|..||:||:.||||:|.|.:...| | .| ||.
RAD53_YEAST      249 QKLNHPRIVRLKGFYEDTESYYMVMEFVSGGDLMDFVAAHGAV-GE-DAG    296

KCC1G_HUMAN      122 LVI-QQVLSAVKYLHENGIVHRDLKPENLLYLTPEENSKIM--ITDFGLS    168
                     ..| :|:|:|:||:|..||.||||||:|:| :  |::..::  ||||||:
RAD53_YEAST      297 REISRQILTAIKYIHSMGISHRDLKPDNIL-I--EQDDPVLVKITDFGLA    343

KCC1G_HUMAN      169 KMEQ-NG-IMSTACGTPGYVAPEVLAQK-----P--------YSKAVDCW    203
                     |: | || .|.|.|||..||||||:..|     |        ||..||.|
RAD53_YEAST      344 KV-QGNGSFMKTFCGTLAYVAPEVIRGKDTSVSPDEYEERNEYSSLVDMW    392

KCC1G_HUMAN      204 SIGVITYILLCGYPPFYEETESKLFEKIKEG-YYEFESPFWDD--ISESA    250
                     |:|.:.|::|.|:.||...|:.:|:::|..| |:  |.|. .|  |||.|
RAD53_YEAST      393 SMGCLVYVILTGHLPFSGSTQDQLYKQIGRGSYH--EGPL-KDFRISEEA    439

KCC1G_HUMAN      251 KDFICHLLEKDPNERYTCEKALSHPWI--D--GNTALHRDIYPS-VSL-Q    294
                     :|||..||:.|||.|.|..|||:||||  .  |:.: :.| : | :|| |
RAD53_YEAST      440 RDFIDSLLQVDPNNRSTAAKALNHPWIKMSPLGSQS-YGD-F-SQISLSQ    486

KCC1G_HUMAN      295 -I--QK---N-------FAKSKWR--QAFNAAAVVHHM----R--KLH-M    322
                      :  ||   |       |.|:: |  | ....  :...     :  |:. .
RAD53_YEAST      487 SLSQQKLLENMDDAQYEFVKAQ-RKLQ-MEQQ--LQEQDQEDQDGKIQGF    532

KCC1G_HUMAN      323 NL--HSPGVR---P---EVENRPPETQ----ASETSR-PSSPE------I    353
                     .:  |:| :|   |   |.|.|  | |    ::.|.. .||.:      :
RAD53_YEAST      533 KIPAHAP-IRYTQPKSIEAETR--E-QKLLHSNNTENVKSSKKKGNGRFL    578

KCC1G_HUMAN      354 TITEAPVL-DHSVAL-PALTQLPCQHGRRP-TAPGGRS--LNCLV--NG-    395
                     |: : | | | |: : .:| ::  |.|..| ..  |||  .||.:  |.
RAD53_YEAST      579 TL-K-P-LPD-SI-IQESL-EI--QQGVNPFFI--GRSEDCNCKIEDNRL    618

KCC1G_HUMAN      396 S-LH--ISSSLVPMHQ-G-SL----AAG--P---C--GCCS-SCLN----    424
                     | :|  |...   .|. | |:    |.|  .   |  | .: |.||
RAD53_YEAST      619 SRVHCFIFKK---RHAVGKSMYESPAQGLDDIWYCHTG-TNVSYLNNNRM    664

KCC1G_HUMAN      425 I-GSK-----G--------KSS-----Y---CSEPT---------L----    439
                     | |:|     |        |::     :   .::.|         |
RAD53_YEAST      665 IQGTKFLLQDGDEIKIIWDKNNKFVIGFKVEINDTTGLFNEGLGMLQEQR    714

KCC1G_HUMAN      440 --LKK-ANKKQNF--K-SEVMV------PVKASGSS-HCR---AGQT---    470
                       ||: |.:| :.  | :::|.      | .||.|| ..:   ...|
RAD53_YEAST      715 VVLKQTAEEK-DLVKKLTQMMAAQRANQP-SASSSSMSAKKPPVSDTNNN    762

KCC1G_HUMAN      471 G---VC--LI    475
                     |   |.  |:
RAD53_YEAST      763 GNNSVLNDLV    772
```

Name 1: APC10_HUMAN
Name 2: APC10_YEAST

Anaphase-promoting complex subunit 10 OS=Homo sapiens GN=ANAPC10 PE=1 SV=1
Anaphase-promoting complex subunit DOC1 OS=Saccharomyces cerevisiae (strain ATCC 204508 / S288c) GN=DOC1 PE=1 SV=2

Needed for following interaction(s):
yeast   physical   APC10_HUMAN   PTTG1_HUMAN   ANAPC10 PTTG1   APC10_YEAST   SECU_YEAST

Length sequence 1:   185
Length sequence 2:   250
Sequence #1: APC10_HUMAN
Sequence #2: APC10_YEAST
Length #1: 227
Length #2: 227
Matrix: BLOSUM62
Gap open: 2.0
Gap extend: 1.0
Length: 227
Identity: 75/227 (33.04%)
Similarity: 112/227 (49.34%)
Gaps: 84/227 (37.00%)
Score: 341.00

```
APC10_HUMAN        5 NKTPPGADPKQLE---RTGTVREI-GS-Q-------AVWSLSSCKP-GF-     40
                     |   |  .|:.|:   ..| : || .| :       |:|     || .|
APC10_YEAST       59 N---P--TPENLQHMFHQG-I-EILDSARMINVTHLALW-----KPSSFK     96

APC10_HUMAN       41 -G--VD-QLRDDNLETYWQSDGSQPH-LVNIQF-RRKTTVKTLCIYADYK     84
                      |  || .| |||.:|:|||||.||| | :|.| :|   : .:|:.|.:.
APC10_YEAST       97 LGNPVDFAL-DDNYDTFWQSDGGQPHQL-DIMFSKR---M-DICVMAIFF    140

APC10_HUMAN       85 S---DESYTPSKISVRV--GNN-----FHNLQEIRQLELVEPSGWIHVPL    124
                     |   ||||.|| : |:|  |::     |:.:.|:|.   |  :||  |.|
APC10_YEAST      141 SMIADESYAPS-L-VKVYAGHSPSDARFYKMLEVRN---V--NGW--VAL    181

APC10_HUMAN      125 --TDNHKKPTRTFMI--Q-IAVL--ANHQNGRDTHMRQIKIYTPV-E---    163
                       .|| ::..:  ::  | |.:|  .||:||:|||:|.|::|.|. |
APC10_YEAST      182 RFLDN-REDDQ--LLKCQFIRLLFPVNHENGKDTHLRGIRLYVPSNEPHQ    228

APC10_HUMAN      164 ---E--SSIGKFPRCT-TI--DFMMYR    182
                        |  .::   |. | .:  |.:: |
APC10_YEAST      229 DTHEWAQTL---PE-TNNVFQDAIL-R    250

--------------------------------------------
Name 1: MPIP3_HUMAN
Name 2: MPIP_DROME

M-phase inducer phosphatase 3 OS=Homo sapiens GN=CDC25C PE=1 SV=2
M-phase inducer phosphatase OS=Drosophila melanogaster GN=stg PE=1 SV=2

Needed for following interaction(s):
fly     genetic RASK_HUMAN      MPIP3_HUMAN     KRAS    CDC25C  RAS1_DROME
MPIP_DROME

Length sequence 1:      473
Length sequence 2:      479
Sequence #1: MPIP3_HUMAN
Sequence #2: MPIP_DROME
Length #1: 547
Length #2: 547
Matrix: BLOSUM62
Gap open: 2.0
Gap extend: 1.0
Length: 547
Identity: 195/547 (35.65%)
Similarity: 266/547 (48.63%)
Gaps: 161/547 (29.43%)
Score: 793.00

MPIP3_HUMAN        1 MSTELFS-STREEGSS-G--SGPSFRSNQRKMLNLL-LER-DTSF---TV     41
                     |...: | :| ...|| .  || | | ..|:.|.|: ::: :.||   .|
MPIP_DROME        14 MDCNI-SNNT-SSSSSINKMSG-S-R-RARRSLELMSMDQEELSFYDDDV     58

MPIP3_HUMAN       42 CP-DVPR--TP--VGKFL---GDSA---NL----SIL-S-G--G--TPKR     70
                     .| |..|  :|  :| .|   | |.   .:    .|| : |  .  ||.|
MPIP_DROME        59 VPQDQQRSASPELMG-LLSPEG-SPQRFQIVRQPKILPAMGVSSDHTPAR    106

MPIP3_HUMAN       71 CLDLSN-LSSGEITATQLTTSADLDETGH--L---DS-SGLQEVHL---A    110
                     ...:.| |||   |.: :.:|.| ||  :  |   :| |  |:..|   :
MPIP_DROME       107 SFRIFNSLSS---TCS-MESSMD-DE--YMELFEMESQS--QQTALGFPS    147

MPIP3_HUMAN      111 GMN---HDQHLMKCSPAQLLCSTPNGLD-RGHR---KRDAMCSSSANKEN    153
                     |:|   ..|  :|..||   ..:|.||. |  |   :|   |.|..  |:
MPIP_DROME       148 GLNSLISGQ--IKEQPA---AKSPAGLSMR--RPSVRR---CLSMT--ES    185

MPIP3_HUMAN      154 DNGNLVDSEMKYLGSPITTVPKLDKNPNLGEDQAEEISDELMEFSLKDQE    203
                      |.|          |  ||.|. .|.|   | .|.   |  . |  |..|
MPIP_DROME       186 -NTN----------S--TTTPP-PKTP---E-TAR---D--C-F--KRPE    209

MPIP3_HUMAN      204 AKVSRSGLYRSPSMPENLNRPRLKQVEKFKDN-TIPDKVKKKYFSGQGKL    252
                     ...| :..  || : :: .|.|...||  |:| ..|..:.:...|....|
MPIP_DROME       210 PPAS-ANC--SP-I-QS-KRHRCAAVE--KENCPAPSPLSQVTISHPPPL    251

MPIP3_HUMAN      253 RKGLCLKKTVSLCDITITQM--L--EEDSNQGHLIGDFSKVCALPTVSGK    298
                     ||  |:    ||.|..|  |  |  .|:.|:..|||||||..|||.:.|:
MPIP_DROME       252 RK--CM----SLNDAEI--MSALARSENRNEPELIGDFSKAYALPLMEGR    293
```

```
MPIP3_HUMAN      299 HQDLKYVNPETVAALLSGKFQGLIEKF--Y-VIDCRYPYEYLGGHIQGAL    345
                     |:|||.::.||||.||.|:|.   :|.  | :||||||||:.||||:||.
MPIP_DROME       294 HRDLKSISSETVARLLKGEFS---DKVASYRIIDCRYPYEFEGGHIEGAK    340

MPIP3_HUMAN      346 NLYSQEELFNFFL--KKPIVPLDTQ-------KRIIIVFHCEFSSERGPR    386
                     |||:.|::.:.||  ::  ..|..|       ||.||:|||||||||||:
MPIP_DROME       341 NLYTTEQILDEFLTVQQ--TELQQQQNAESGHKRNIIIFHCEFSSERGPK    388

MPIP3_HUMAN      387 MCRCLR--EEDRSLNQYPALYYPELYILKGGYRDFFPE-YMELCEPQSYC    433
                     |.|.||  :.:|:.|.||||:|||:|:|..||::|| | ::|||||.:|.
MPIP_DROME       389 MSRFLRNLDRERNTNAYPALHYPEIYLLHNGYKEFF-ESHVELCEPHAYR    437

MPIP3_HUMAN      434 PMHHQD--HKTELLR-CRSQSKVQEGE-------RQL---REQIALL    467
                     .|  .|  : .|..| .|::||...|:       | |   |.:: :|
MPIP_DROME       438 TM--LDPAY-NEAYRHFRAKSKSWNGDGLGGATGR-LKKSRSRL-ML    479

--------------------------------------------
Name 1: DYN2_HUMAN
Name 2: DNM1_YEAST

Dynamin-2 OS=Homo sapiens GN=DNM2 PE=1 SV=2
Dynamin-related protein DNM1 OS=Saccharomyces cerevisiae (strain ATCC 204508 / S
288c) GN=DNM1 PE=1 SV=1

Needed for following interaction(s):
yeast   genetic DYN2_HUMAN      RASK_HUMAN      DNM2    KRAS    DNM1_YEAST
IRA2_YEAST

Length sequence 1:      870
Length sequence 2:      757
Sequence #1: DYN2_HUMAN
Sequence #2: DNM1_YEAST
Length #1: 842
Length #2: 842
Matrix: BLOSUM62
Gap open: 2.0
Gap extend: 1.0
Length: 842
Identity: 349/842 (41.45%)
Similarity: 490/842 (58.19%)
Gaps: 191/842 (22.68%)
Score: 1592.00

DYN2_HUMAN         1 MGNRGMEELIPLVNKLQDA-FSSIG-QSCHLDLPQI-AVVGGQSAGKSSV     47
                     |.:  :|:|||.||||||. :.| | .:  |||| | ||||.||:||||:
DNM1_YEAST         1 MAS--LEDLIPTVNKLQDVMYDS-GIDT--LDLP-ILAVVGSQSSGKSSI     44

DYN2_HUMAN        48 LENFVGRDFLPRGSGIVTRRPLILQL--I------F-----S--------     76
                     ||..|||||||||:||||||||:|||  |      .     |
DNM1_YEAST        45 LETLVGRDFLPRGTGIVTRRPLVLQLNNISPNSPLIEEDDNSVNPHDEVT     94

DYN2_HUMAN        77 K-------T--------E--HA----EFLHCKSKKFTDFDEVRQEIEAET    105
                     |       |        |  ||    ||||...|:|.|||::::|||.||
DNM1_YEAST        95 KISGFEAGTKPLEYRGKERNHADEWGEFLHIPGKRFYDFDDIKREIENET    144

DYN2_HUMAN       106 DRVTGTNKGISPVPINLRVYSPHVLNLTLIDLPGITKVPVGDQPPDIEYQ    155
                     .|:.|.:||||.:||||:|:|||||||||:|||||||||:|:||||||.|
DNM1_YEAST       145 ARIAGKDKGISKIPINLKVFSPHVLNLTLVDLPGITKVPIGEQPPDIEKQ    194

DYN2_HUMAN       156 IKDMILQFISRESSLILAVTPANMDLANSDALKLAKEVDPQGLRTIGVIT    205
                     ||::||.:|:..:.|||||:|||:||.||::||||:||||||.|||||||
DNM1_YEAST       195 IKNLILDYIATPNCLILAVSPANVDLVNSESLKLAREVDPQGKRTIGVIT    244

DYN2_HUMAN       206 KLDLMDEGTDARDVLENKLLPLRRGYIGVVNRSQKDIEGKKDIRAALAAE    255
                     ||||||.||:|.|:|..|:.||:.|::|||||||:||:..|.:..:|..|
DNM1_YEAST       245 KLDLMDSGTNALDILSGKMYPLKLGFVGVVNRSQQDIQLNKTVEESLDKE    294

DYN2_HUMAN       256 RKFFLSHPAYRHMADRMGTPHLQKTLNQQLTNHIRESLPALRSKLQSQLL    305
                     ..:|..||.||.::.:.||.:|.|.|||.|.:|||:.||.:::||.: |:
DNM1_YEAST       295 EDYFRKHPVYRTISTKCGTRYLAKLLNQTLLSHIRDKLPDIKTKLNT-LI    343

DYN2_HUMAN       306 S-LEKEVEEYKNFRPDDPTRKTKA-L-LQMVQQFGVDFEKRIEG-SGDQV    351
                     | .|:|:..|...  ...|.:::| | ||::.:|..:|...|:| |.| :
```

```
DNM1_YEAST       344 SQTEQELARYGGV--GATTNESRASLVLQLMNKFSTNFISSIDGTSSD-I    390

DYN2_HUMAN       352 DTLELSGGARI----NRIF-HE-R-F-P-FEL-VKMEFDEKDLRREISYA    391
                     :|.||.|||||    |.:| :. : . | ..| | :     |:|   : |
DNM1_YEAST       391 NTKELCGGARIYYIYNNVFGNSLKSIDPTSNLSV-L-----DVR---T-A    430

DYN2_HUMAN       392 IKNIHGVR-TGLFTPDLAFEAIVKKQVVK-LKEPCLKCVDLVIQELINTV    439
                     |:|..|.| | ||.|:|||:.:||.| :| |.||..:||:||.:||:. :
DNM1_YEAST       431 IRNSTGPRPT-LFVPELAFDLLVKPQ-IKLLLEPSQRCVELVYEELMK-I    477

DYN2_HUMAN       440 -RQC-TSKLSSYPRLREE-TERIVTTYIRER-EGRTKDQILLLIDIEQSY    485
                      .:| :::|:.||:|:.. .| :::..:||| : .|:..:..||||.::|
DNM1_YEAST       478 CHKCGSAELARYPKLKSMLIE-VISELLRERLQ-PTRSYVESLIDIHRAY    525

DYN2_HUMAN       486 INTNHEDFIGFA-NAQQ--RSTQLNKKRAIPNQGEIL---VIRR--GWLT    527
                     |||||.:|:. | .|..  ..|:  :||   || |:|   :.::  | .|
DNM1_YEAST       526 INTNHPNFLS-ATEAMDDIMKTR--RKR---NQ-ELLKSKLSQQENG-QT    567

DYN2_HUMAN       528 INNISLMKGGSKEYWFVLTAESLSWYK-DEEE-KEKKYMLPLDNLKIRDV    575
                      |.|:    |         |: |:| .. |::. |...|  . |: .| |.
DNM1_YEAST       568 -NGIN----G--------TS-SIS-SNIDQDSAKNSDY--D-DD-GI-DA    597

DYN2_HUMAN       576 E----KG-FMSNKHVFAIFNTEQRN--VY--KD-LRQIELACDSQ-EDVD    614
                     |    |. |: | : |  |..:::.  |:  .| .|.|  |.|.. ||..
DNM1_YEAST       598 ESKQTKDKFL-N-Y-F--FGKDKKGQPVFDASDKKRSI--AGDGNIEDFR    640

DYN2_HUMAN       615 SWKAS-F-LRAG-VYPEKDQAENEDGAQENTFSMDPQL-ER-QV--ETIR    657
                     :.:.| | |  | :    |..||    | |       |.| || ::  |.|:
DNM1_YEAST       641 NLQISDFSL--GDI----DDLEN---A-E------PPLTEREELECELIK    674

DYN2_HUMAN       658 NLVDSYVAIINKSIRDLMPKTIMHLMINNTK-AFIHHELLAYLYSSADQS    706
                     .|:.||..||.:.|.|.:||.:|.|::|..| : :.:.|:..||    :.
DNM1_YEAST       675 RLIVSYFDIIREMIEDQVPKAVMCLLVNYCKDS-VQNRLVTKLY----KE    719

DYN2_HUMAN       707 SLMEE--SADQ--AQRRDDM----LRMYHALKEA--L--NII    736
                     :|.||  ..||  ||.| ::    |.:|   |:|  |  ||:
DNM1_YEAST       720 TLFEELLVEDQTLAQDR-ELCVKSLGVY---KKAATLISNIL    757

--------------------------------------------
```

Name 1: TTC31_HUMAN
Name 2: STI1_YEAST

Tetratricopeptide repeat protein 31 OS=Homo sapiens GN=TTC31 PE=2 SV=3
Heat shock protein STI1 OS=Saccharomyces cerevisiae (strain ATCC 204508 / S288c) GN=STI1 PE=1 SV=1

Needed for following interaction(s):
yeast genetic PTTG1_HUMAN TTC31_HUMAN PTTG1 TTC31 SECU_YEAST STI1_YEAST

Length sequence 1: 519
Length sequence 2: 589
Sequence #1: TTC31_HUMAN
Sequence #2: STI1_YEAST
Length #1: 665
Length #2: 665
Matrix: BLOSUM62
Gap open: 2.0
Gap extend: 1.0
Length: 665
Identity: 168/665 (25.26%)
Similarity: 267/665 (40.15%)
Gaps: 253/665 (38.05%)
Score: 513.00

```
TTC31_HUMAN        2 A-PIPKTVGRIKLDCSLRPSC--PL-EVA-A---APKLC-KEFGP---ED     39
                     | .:.:|...: | .|.|.:|  .| :.: |   |.: | | ..|   :.
STI1_YEAST        31 AIEVSETPNHV-L-YSNRSACYTSLKKFSDALNDANE-CVK-INPSWSKG     76

TTC31_HUMAN       40 Y---G-EE----DIVD-----FLRR-L-VE-SD---PQGLHRIH-VDGSS     69
                     |   | ..    |: |     : :: | :: |:   .:||.::| .  ..
STI1_YEAST        77 YNRLGAAHLGLGDL-DEAESNY-KKALELDASNKAAKEGLDQVHRT--QQ    122

TTC31_HUMAN       70 GRLQLWHHDYLLG--HL--D-D--EG--KS--TGQ--SD-----R--G-K     98
                     .| |. ..|  ||  .|  | :  |.  |:  |.:  .|     :  | |
STI1_YEAST       123 AR-QA-QPD--LGLTQLFADPNLIENLKKNPKTSEMMKDPQLVAKLIGYK    168

TTC31_HUMAN       99 -G--AEG--------L----GTYCG--L-----RKS-FLYP--PQ--ES-    120
                      .  |.|        |     .|..|  |      .:| .: |  |:  :|
STI1_YEAST       169 QNPQAIGQDLFTDPRLMTIMATLMGVDLNMDDINQSNSM-PKEPETSKST    217

TTC31_HUMAN      121 E---PC-PQSPSASATFPSVSDSLLQVAMPQKLLVTEEEANRLAE--ELV    164
                     |    .. ||| . |.|  |..:|   ..| |||      ||:.. :|  | |
STI1_YEAST       218 EQKKDAEPQS-D-STT--SKENS--SKA-PQK-----EESKE-SEPME-V    253

TTC31_HUMAN      165 AEEE-RM---KQKAE-KKRLKKKRQ--K--E--RKRQERLEQ---YCGEP    200
                     .|:: ::    |:|||  .| ..|.||  :  |   .|..| |.:   |...
STI1_YEAST       254 DEDDSKIEADKEKAEGNK-FYKARQFDEAIEHYNKAWE-LHKDITYLNN-    300

TTC31_HUMAN      201 KASTTSD-GD-ESPPSSPGN-PVQGQCGEE--ED-SLDLSSTFVSL--AL    242
                     :|:...: |: |:..|:. | .|: | |.|  .| .: :|.:|..:  |.
STI1_YEAST       301 RAAAEYEKGEYETAISTL-NDAVE-Q-GREMRADYKV-ISKSFARIGNAY    346

TTC31_HUMAN      243 RKVGDWPLSARR--E---KGLNQEPQGRGLA--LQKMGQEEESPPRE-ER    284
                     .|:||  |  ::  |    |.|. | . | .|  |.|: :..|   :| ::
STI1_YEAST       347 HKLGD--L--KKTIEYYQKSLT-E-H-R-TADILTKL-RNAE---KELKK    384

TTC31_HUMAN      285 PQ-QSPKVQASPGLLAAALQQSQELAKL-GTS-FAQNGFYHEAVVLFTQA    331
                     .: :: .|  :|        ::::| |:| |.. |.::. :..||..:|:.
STI1_YEAST       385 AEAEA-YV--NP-------EKAEE-ARLEGKEYFTKSD-WPNAVKAYTEM    422

TTC31_HUMAN      332 LKLNPQDHRLFG--NRSFCHERL-GQPAWALADAQVALTLR-PGWPRGLF    377
                     :|..|:|.|  |  ||:....:| ..|. |:||...|:. : |.:.|...
STI1_YEAST       423 IKRAPEDAR--GYSNRAAALAKLMSFPE-AIADCNKAIE-KDPNFVRAYI    468

TTC31_HUMAN      378 RLGKALMGLQ-RFRE-AAAVFQETL---R-------GGSQPDAARELRSC    415
                     |  ||.  .| ..:| |:|:  |||   |        .||   :|||:..
STI1_YEAST       469 R--KAT--AQIAVKEYASAL--ETLDAARTKDAEVNNGS---SAREIDQ-    508

TTC31_HUMAN      416 LLHLTLQGQRGGICAPPLSPGALQPLPHAELAPSGLPSLRCPRSTALRSP    465
                     |.:...| ||       ..||.    .: | .|.  .:.:  |  |::.|
STI1_YEAST       509 LYYKASQ-QR-------FQPGT----SN-E-TPE--ETYQ--R--AMKDP    538

TTC31_HUMAN      466 GLSPLLHYP---S-CHRSHPNQP--LSQTQSRRPHPLKPQDPS--KGWDI    507
                     .::.::..|   | ..::..| |  | | :    | :|  :|.  |  .|
STI1_YEAST       539 EVAAIMQDPVMQSILQQAQQN-PAAL-Q-E----H-MK--NPEVFK--KI    576

TTC31_HUMAN      508 --L-GLGLQHLSQAR    519
                       | ..|:  :...|
STI1_YEAST       577 QTLIAAGI--IRTGR    589

--------------------------------------------
```

Name 1: TFDP1_HUMAN
Name 2: TFDP_DROME

Transcription factor Dp-1 OS=Homo sapiens GN=TFDP1 PE=1 SV=1
Transcription factor Dp OS=Drosophila melanogaster GN=Dp PE=1 SV=2

Needed for following interaction(s):
fly genetic RASK_HUMAN TFDP1_HUMAN KRAS TFDP1 RAS1_DROME TFDP_DROME

Length sequence 1: 410
Length sequence 2: 445
Sequence #1: TFDP1_HUMAN
Sequence #2: TFDP_DROME
Length #1: 489
Length #2: 489
Matrix: BLOSUM62
Gap open: 2.0
Gap extend: 1.0
Length: 489
Identity: 216/489 (44.17%)
Similarity: 273/489 (55.83%)
Gaps: 123/489 (25.15%)
Score: 946.00

```
TFDP1_HUMAN        1 MA-KDAGLI---EAN-------GEL-KVFID--QNLSP---GKGVVSLV-     32
```

```
                     || ...|.:   |.|       |:: |: :.  ||.|.   || .|::|
TFDP_DROME         1 MAHSTGGTVKTDEVNFFFRNEHGQISKM-LKPAQNKSEMEGGK-PVAVVY     48

TFDP1_HUMAN       33 AV-----HP-S-T-V-NPLGKQLLPKTFGQSNVNIAQ-QVV-I---G-T-     66
                     |.     :. | : : | :|:  : ..|.|  :. :| |.: :   | :
TFDP_DROME        49 ATGSSARNSGSASGIGN-VGR--M-GAFSQ--MG-SQGQFIRLQDNGLSI     91

TFDP1_HUMAN       67 PQRPAASN-TLV-------VGS-----P-----H---TP------S-THF     88
                     |:..|.:. |.|       .||     |     :   ||      | .|
TFDP_DROME        92 PKTEAGTTYTTVSAQKTSGAGSGHYDLPLKGDRYVKFTPNPIKMKSKLH-    140

TFDP1_HUMAN       89 ASQ-N---QPSDSSPWSAGKRNRKGEKNGKGLRHFSMKVCEKVQRKG-TT    133
                     |.| |   ..|.||  |:.:|.||.:|.|||||||||||||||:.|| ||
TFDP_DROME       141 AIQSNSLHSMSASS--SSVQRKRKPDKAGKGLRHFSMKVCEKVEEKGKTT    188

TFDP1_HUMAN      134 SYNEVADELVA-EF--SAADNHILPNESAYDQKNIRRRVYDALNVLMAMN    180
                      ||||||:||: |.  :|.||    |  . ||||||||||||||||||:|
TFDP_DROME       189 -YNEVADDLVSEEMKNNAYDN----N--C-DQKNIRRRVYDALNVLMAIN    230

TFDP1_HUMAN      181 IISKEKKEIKWIGLPTNSAQEC-QNLEVER-QRRLERIKQKQSQLQELIL    228
                     :|||:||||:|||||.||. |. ..||.|. ||| ||||||...|:|:|:
TFDP_DROME       231 VISKDKKEIRWIGLPANST-ETFLALEEENCQRR-ERIKQKNEMLREMIM    278

TFDP1_HUMAN      229 QQIAFKNLVQRN-RHAEQQASRPPPPN-SVIHLPFIIVNTSKKTVIDCSI    276
                     |.:|||.||:|| |: |.|.. .|.|| | |.||||||||.|.|.|:||:
TFDP_DROME       279 QHVAFKGLVERNKRN-ESQGV-VPSPNAS-IQLPFIIVNTHKSTKINCSV    325

TFDP1_HUMAN      277 SNDKFEYLFNFDNTFEIHDDIEVLKRMGMACGLESGSCSAEDLKMARSLV    326
                     :|||.||:|.||.|||:|||||||||||...||:.|.|:.|:::..:|.|
TFDP_DROME       326 TNDKSEYIFKFDKTFEMHDDIEVLKRMGFLLGLDKGECTPENIERVKSWV    375

TFDP1_HUMAN      327 PKALEPYVTEMAQGTVGGVFITTAGSTSNGTRFSASDLTNGADGML--AT    374
                     |..|..|| | |.||  |   .| |  .|  .:.:.|..|..:|.|  |.
TFDP_DROME       376 PPNLAKYV-E-AYGT--G---KT-G--EN--MYESDDEDNEFNGYLESAN    413

TFDP1_HUMAN      375 SSNG-SQYSGSRVETPVSYVGEDD-EEDDDFNENDED-D    410
                     .|.| :|:| :: .| ..  ||.. |.|||  |.|:| |
TFDP_DROME       414 ESQGFAQHS-AQ-HT-TD--GEFKLEMDDD--ELDDDID    445
```

Name 1: SURF4_HUMAN
Name 2: ERV29_YEAST

Surfeit locus protein 4 OS=Homo sapiens GN=SURF4 PE=1 SV=3
ER-derived vesicles protein ERV29 OS=Saccharomyces cerevisiae (strain ATCC 20450
8 / S288c) GN=ERV29 PE=1 SV=1

Needed for following interaction(s):
yeast   genetic RASK_HUMAN   SURF4_HUMAN   KRAS   SURF4   IRA2_YEAST
ERV29_YEAST

Length sequence 1:   269
Length sequence 2:   310
Sequence #1: SURF4_HUMAN
Sequence #2: ERV29_YEAST
Length #1: 307
Length #2: 307
Matrix: BLOSUM62
Gap open: 2.0
Gap extend: 1.0
Length: 307
Identity: 113/307 (36.81%)
Similarity: 167/307 (54.40%)
Gaps: 72/307 (23.45%)
Score: 520.00

```
SURF4_HUMAN        3 QN-DLMG-TAE---DFADQFL-RV---T--------KQYLPHVARLCLIS     35
                     || ...| ..:   .| ::|. |:   |        |.|:|.::|..:::
ERV29_YEAST       32 QNQSTSGILKQWKHSF-EKFASRIEGLTDNAVVYKLKPYIPSLSRFFIVA     80

SURF4_HUMAN       36 TFLEDGIRMWFQWSEQRDYIDTTW-NCGYLLASSF--VFLNL--LGQLTG     80
                     ||.||..|:..|||:|..|:: .| :..|.    |  |||.:  :..|.|
ERV29_YEAST       81 TFYEDSFRILSQWSDQIFYLN-KWKHYPYF----FVVVFLVVVTVSMLIG    125

SURF4_HUMAN       81 C-VLVLSRNFVQ--YACFG-LFG-IIALQTIAYSILW-DLKFLMRNLA-L    123
                     . :||| |.  |  ||. | |.. :|: |.:.|. |: ...|::||.: :
ERV29_YEAST      126 ASLLVL-RK--QTNYAT-GVLCACVIS-QALVYG-LFTGSSFVLRNFSVI    169

SURF4_HUMAN      124 GGGLLLLLAESRS--EGKSMFAG-VPTMRESSPK-Q-YMQL-GGRVLLVL    167
                     ||   ||:|.|.|  :.|:.| | :|.:...:.| : |: | .||:|:||
ERV29_YEAST      170 GG---LLIAFSDSIVQNKTTF-GMLPELNSKNDKAKGYL-LFAGRILIVL    214

SURF4_HUMAN      168 MFM--TLLHFDASFFSIVQNIVGTALMILVAIGFKTKLAALTL-VVWL-F    213
                     ||:  |   |..|:|::|..|:||   |..|||:|||.|::.| :: | |
ERV29_YEAST      215 MFIAFT---FSKSWFTVVLTIIGT---ICFAIGYKTKFASIMLGLI-LTF    257

SURF4_HUMAN      214 AINVYFNAFWTIPVY---KPMHDFLKYDFFQTMSVIGGLLLVVALGPGGV    260
                      .|:..|.:|   .|   |  .|||||:|:|.:|:|||||||...|.|.:
ERV29_YEAST      258 -YNITLNNYW---FYNNTK--RDFLKYEFYQNLSIIGGLLLVTNTGAGEL    301

SURF4_HUMAN      261 SMDEKKK    267
                     |:|||||
ERV29_YEAST      302 SVDEKKK    308
```

Name 1: YMEL1_HUMAN
Name 2: YME1_YEAST

ATP-dependent zinc metalloprotease YME1L1 OS=Homo sapiens GN=YME1L1 PE=1 SV=2
Mitochondrial inner membrane i-AAA protease supercomplex subunit YME1 OS=Sacchar
omyces cerevisiae (strain ATCC 204508 / S288c) GN=YME1 PE=1 SV=1

Needed for following interaction(s):
yeast   genetic BLM_HUMAN   YMEL1_HUMAN   BLM   YME1L1 SGS1_YEAST
YME1_YEAST

Length sequence 1:   773
Length sequence 2:   747
Sequence #1: YMEL1_HUMAN
Sequence #2: YME1_YEAST
Length #1: 853
Length #2: 853
Matrix: BLOSUM62
Gap open: 2.0
Gap extend: 1.0
Length: 853
Identity: 351/853 (41.15%)
Similarity: 486/853 (56.98%)
Gaps: 217/853 (25.44%)
Score: 1544.00

```
YMEL1_HUMAN        1 MFSLSS-TVQPQVTVPLSHLINAFHTPKNTSV-SLSGVSVSQNQHRDVVP     48
                     | ::|. .|.|.||   ::::..| .|:   : .: |.|:       :|
YME1_YEAST         1 M-NVSKILVSPTVT---TNVLRIF-APR---LPQI-GASL-------LV-     33

YMEL1_HUMAN       49 EHE-APSSECMFSDFLT-KLNIVSIG----KGKIFEGYRSMFMEPAKRMK     92
                     :.: |..|: .|..|.: | |  | |    | |  |. .|    ..|...
YME1_YEAST        34 QKKWALRSK-KFYRFYSEK-N--S-GEMPPK-K--EA-DS----SGKASN     70

YMEL1_HUMAN       93 KS-LDTTDNWHIRPEPFSLSIPPSLNLRDLGLSELKIGQIDQLVENLLPG    141
                     || :.:.||  .:| |     ||| |..|    :.|     |  .|:
YME1_YEAST        71 KSTISSIDN--SQP-P-----PPS-NTND----KTK-----Q--ANV---     97

YMEL1_HUMAN      142 FCKGKNISSHWHTSHVSA--QSFFE-NKYGNLDIFSTLRS----SCLYRH    184
                        .  :|   | :.::.  |   | ||    |:  |  |    :..|:
YME1_YEAST        98 ---A--VS---H-AMLATREQ---EANK----DL--T--SPDAQAAFYK-    126

YMEL1_HUMAN      185 HSRALQSICSDLQYWPVFIQSRGFKTLKSRTRRLQSTSERLAETQNIAPS    234
                       ..|||     .| |.::.|| |:| ..    :.|:.|.: |   :
YME1_YEAST       127 --LLLQS-----NY-PQYVVSR-FET-PG----IASSPECM-E---L---    155

YMEL1_HUMAN      235 FVKGFLLRDR-G--S--D-V-ESLDKLMKTK----NIPE-AHQDAFKTGF    272
                     :::.  |: | |  |  | | ::|  |..:.    | |. |...:.::|:
YME1_YEAST       156 YMEA--LQ-RIGRHSEADAVRQNL--LTASSAGAVN-PSLASSSSNQSGY    199

YMEL1_HUMAN      273 AEG-F--LKAQAL--TQK------TNDSLRRT-RLI-LFV--LLLFGI--    305
                      .| |  :.: .|  ::|      .::|   | .:: .:|  ||:|||
YME1_YEAST       200 -HGNFPSMYS-PLYGSRKEPLHVVVSES---TFTVVSRWVKWLLVFGILT    244
```

```
YMEL1_HUMAN      306 YGLLKNPFLSVRFR--T--TTGL-------DSAVDPVQMK-NVTFEHVKG    343
                     |    : | |..|:  |  || |       |.:|| | .| ||.|:.|.|
YME1_YEAST       245 Y----S-F-SEGFKYITENTT-LLKSSEVADKSVD-V-AKTNVKFDDVCG    285

YMEL1_HUMAN      344 VEEAKQELQEVVEFLKNPQKFTILGGKLPKGILLVGPPGTGKTLLARAVA    393
                     .:||:.||:|:|:|||:|.|:..||||||||:||.|||||||||||||.|
YME1_YEAST       286 CDEARAELEEIVDFLKDPTKYESLGGKLPKGVLLTGPPGTGKTLLARATA    335

YMEL1_HUMAN      394 GEADVPFYYASGSEFDEMFVGVGASRIRNLFREAKANAPCVIFIDELDSV    443
                     |||.|.|::.|||||||::|||||.|||:||.:|::.||.:|||||||::
YME1_YEAST       336 GEAGVDFFFMSGSEFDEVYVGVGAKRIRDLFAQARSRAPAIIFIDELDAI    385

YMEL1_HUMAN      444 GGKRIESPM-HPYSRQTINQLLAEMDGFKPNEGVIIIGATNFPEALDNAL    492
                     ||||  :|. ..|::||:||||.|:|||....|:|||||||||||||.||
YME1_YEAST       386 GGKR--NPKDQAYAKQTLNQLLVELDGFSQTSGIIIIGATNFPEALDKAL    433

YMEL1_HUMAN      493 IRPGRFDMQVTVPRPDVKGRTEILKWYLNKIKF-DQSVDPEIIARGTVGF    541
                     .||||||..|.|..|||:||.:|||.::.||.. | :|||.||||||.|.
YME1_YEAST       434 TRPGRFDKVVNVDLPDVRGRADILKHHMKKITLAD-NVDPTIIARGTPGL    482

YMEL1_HUMAN      542 SGAELENLVNQAAL----KAAVDGKEMVTMKELEFSKDKILMGPERRS-V    586
                     |||||.|||||||:    |.||.    |.|...|::|||||||.||:: |
YME1_YEAST       483 SGAELANLVNQAAVYACQKNAVS----VDMSHFEWAKDKILMGAERKTMV    528

YMEL1_HUMAN      587 EI-DNKNKTITAYHESGHAIIAYYTKDAMPINKATIMPRGPTLGHVSL-L    634
                      : |...|. ||:||:||||:|.||..|.|:.||||:|||..|| ::. |
YME1_YEAST       529 -LTDAARKA-TAFHEAGHAIMAKYTNGATPLYKATILPRGRALG-ITFQL    575

YMEL1_HUMAN      635 PENDRWNET-R-AQLLAQMDVSMGGRVAEELIFGTDHITT-GASSDFDNA    681
                     ||.|:.:.| | .|  |::||.|||::|||||:|.|: || |..||..:|
YME1_YEAST       576 PEMDKVDITKRECQ--ARLDVCMGGKIAEELIYGKDN-TTSGCGSDLQSA    622

YMEL1_HUMAN      682 TKIAKRMVTKFGMSEKLG-V-MT--Y-SDTGKLSPETQSAIEQEIRI-LL    725
                     |..|:.|||::|||:.:| | ::  : |.:.|:. :.  | :.|: | ||
YME1_YEAST       623 TGTARAMVTQYGMSDDVGPVNLSENWESWSNKIR-DI--A-DNEV-IELL    667

YMEL1_HUMAN      726 RDSYERAKHILKTHAK--E-HKNLAEALLTYETLDAKEI-QIVLEGKKLE    771
                     :||.|||:.:| | .|  | |: ||:.|:.||||||.|| | |.:|:||:
YME1_YEAST       668 KDSEERARRLL-T-KKNVELHR-LAQGLIEYETLDAHEIEQ-VCKGEKLD    713

YMEL1_HUMAN      772 -VR    773
                      ::
YME1_YEAST       714 KLK    716
```

Name 1: BR44_HUMAN
Name 2: FMP43_YEAST

Brain protein 44 OS=Homo sapiens GN=BRP44 PE=1 SV=1
UPF0041 protein FMP43 OS=Saccharomyces cerevisiae (strain ATCC 204508 / S288c) GN=FMP43 PE=1 SV=1

Needed for following interaction(s):
yeast genetic RASK_HUMAN BR44_HUMAN KRAS BRP44 IRA2_YEAST FMP43_YEAST

Length sequence 1: 127
Length sequence 2: 146
Sequence #1: BR44_HUMAN
Sequence #2: FMP43_YEAST
Length #1: 138
Length #2: 138
Matrix: BLOSUM62
Gap open: 2.0
Gap extend: 1.0
Length: 138
Identity: 61/138 (44.20%)
Similarity: 78/138 (56.52%)
Gaps: 33/138 (23.91%)
Score: 280.00

```
BR44_HUMAN         1 MSAAGARGLRATYHRLLDKVELMLPEKLRPLYNHPAGPRTVFFWAPIMKW     50
                     |||: |... | : |             | .:|...||:||.||||.:||
FMP43_YEAST        1 MSAS-AFNF-A-F-R-------------R-FWNSETGPKTVHFWAPTLKW     32

BR44_HUMAN        51 GLVCAGLADMARPAEKLSTAQSAVLMATGFIWSRYSLVIIPKNWSLFA-V     99
                     |||.|||.|:.||.||:|.||:..|:||..||:|:|.||.|||: |.| |
FMP43_YEAST       33 GLVFAGLNDIKRPVEKVSGAQNLSLLATALIWTRWSFVIKPKNY-LLASV     81

BR44_HUMAN       100 NFFVG-AAG--ASQL--FRIWRYN-QELKAKA-H---K    127
                     |||:| .||  .:::  ||| | | ...| :. |   |
FMP43_YEAST       82 NFFLGCTAGYHLTRIANFRI-R-NGDSFK-QVIHYIIK    116
```

Name 1: BICD2_HUMAN
Name 2: SMC3_YEAST

Protein bicaudal D homolog 2 OS=Homo sapiens GN=BICD2 PE=1 SV=1
Structural maintenance of chromosomes protein 3 OS=Saccharomyces cerevisiae (strain ATCC 204508 / S288c) GN=SMC3 PE=1 SV=1

Needed for following interaction(s):
yeast genetic BICD2_HUMAN BLM_HUMAN BICD2 BLM SMC3_YEAST SGS1_YEAST

Length sequence 1: 824
Length sequence 2: 1230
Sequence #1: BICD2_HUMAN
Sequence #2: SMC3_YEAST
Length #1: 1187
Length #2: 1187
Matrix: BLOSUM62
Gap open: 2.0
Gap extend: 1.0
Length: 1187
Identity: 333/1187 (28.05%)
Similarity: 508/1187 (42.80%)
Gaps: 489/1187 (41.20%)
Score: 995.00

```
BICD2_HUMAN        2 SAPSEEEEYARL-VME----AQP-E-WLRAEVKRLSHELAETTREKIQA-     43
                     :|  :::|  || ::|    |:. | .|:|.:|::  |  ||.::|||.
SMC3_YEAST       156 NA--KDKE--RLQLLEDVVGAKSFEVKLKASLKKM--E--ETEQKKIQIN    197

BICD2_HUMAN       44 AEYG-L-AVL-E-EKHQLK-LQ-FEELE----VDYE-AI--RSEM-E---     76
                     .|.| | :.| | |:.: | |: :.|||    : |: .:  | |: |
SMC3_YEAST       198 KEMGELNSKLSEMEQER-KELEKYNELERNRKI-YQFTLYDR-ELNEVIN    244

BICD2_HUMAN       77 QLKEAF-GQAHTNHKKVAADGESREESLIQESASK-E----QYYV-RKV-    118
                     |: |.. |. : |: .|.:   | |: .||| ..| |     |  | :|:
SMC3_YEAST       245 QM-ERLDGD-Y-NN-TVYS---S-EQ-YIQE-LDKREDMIDQ--VSKKLS    282

BICD2_HUMAN      119 -LE--LQ----TELKQ--LR--NV---LTNT--------QS-E-NE--R-    141
                      :|  |:    |:|:|  ||  .:   |||.        |. | ||  |
SMC3_YEAST       283 SIEASLKIKNATDLQQAKLRESEISQKLTNVNVKIKDVQQQIESNEEQRN    332

BICD2_HUMAN      142 LASVAQELKEINQN-VEIQR----GR-L-R-DDI-KE---YKFREA----    175
                     |.| | .|||| :: :| ||     .: | | .:: ||   ||.:.|
SMC3_YEAST       333 LDS-A-TLKEI-KSIIE-QRKQKLSKILPRYQELTKEEAMYKLQLASLQQ    378

BICD2_HUMAN      176 --R-L-LQ--DY----SELEE-EN-I-S-LQ--KQVSVLRQNQV-EFEG-    207
                       | | |:  :|    |: :| :. | | ::  |. |:  || : |.|.
SMC3_YEAST       379 KQRDLILKKGEYARFKSK-DERDTWIHSEIEELKS-SI--QN-LNELESQ    423

BICD2_HUMAN      208 LKHE---I-KR---LEEETEYL----N-----SQLE--DA--IRLKE-IS    236
                     |:.:   : |:   ::||.|.|    |     .|||  |:  |.||: :|
SMC3_YEAST       424 LQMDRTSLRKQYSAIDEEIEELIDSINGPDTKGQLEDFDSELIHLKQKLS    473

BICD2_HUMAN      237 E----R-QL---EEALET-LKT---E--REQKN-------SL-----R-K    259
                     |    | :|   |:.|:| |:|   :  :.|:|       ||     . |
SMC3_YEAST       474 ESLDTRKELWRKEQKLQTVLETLLSDVNQNQRNVNETMSRSLANGIINVK    523

BICD2_HUMAN      260 ELSHYMSIN-DS-FYT-SHL-HVSLDGLKFSDDAAE---PNN------DA    296
                     |::..:.|: :| |.| ..| .|: |  |:. ..||   .|:       |.
SMC3_YEAST       524 EITEKLKISPESVFGTLGELIKVN-D--KYK-TCAEVIGGNSLFHIVVDT    569

BICD2_HUMAN      297 E--A-LV-NGFE-H---GG------LAKLPLD-------NKTS----TP-    320
```

```
                    |  | |: |  | :   ||      |.:|.||       |.|:    ||
SMC3_YEAST       570 EETATLIMN--ELYRMKGGRVTFIPLNRLSLDSDVKFPSNTTTQIQFTPL    617

BICD2_HUMAN      321 -KK---EGLAPPSPSLVSDLLSE-LNISEI-QKLKQQLMQMEREKAGLLA    364
                      ||   |   |.....|..:..: :.:.:: |.||  |  .::.|  |.|
SMC3_YEAST       618 IKKIKYE---PRFEKAVKHVFGKTIVVKDLGQGLK--L--AKKHK--LNA    658

BICD2_HUMAN      365 -TLQ-D-TQKQ--LEHTRGSLSEQQEKVTRLTENLSALR--RLQASK--E    405
                      ||. | ..|:  |  |.|.| :|.:: ||| |:|..|.  |.|..|  |
SMC3_YEAST       659 ITLDGDRADKRGVL--TGGYL-DQHKR-TRL-ESLKNLNESRSQHKKILE    703

BICD2_HUMAN      406 RQTALD---NE-KDRDSHE-D---GDYYEVDINGPE-ILA-CK-YHVAV-    443
                      :  ||   || .|.|: : |   |:..:|. |..| :|. .: |..::
SMC3_YEAST       704 -E--LDFVRNELNDIDT-KIDQVNGNIRKVS-NDRESVLTNIEVYRTSLN    748

BICD2_HUMAN      444 AEAGE---LREQLKA--LR----STHEARE-AQHAEEK-GRYE----AE-    477
                     .:..|   |.|.|.|  |:    :|:  |. ||   || ..:|     .|
SMC3_YEAST       749 TKKNEKLILEESLNAIILKLEKLNTN--RTFAQ---EKLNTFENDLLQEF    793

BICD2_HUMAN      478 -GQALT--EK--V-SLL-E-KA--SR----QDR-E-----L--L-ARLE-    503
                      .: |:  ||  : ||. | .|  ::     .|. |      :  | |.||
SMC3_YEAST       794 DSE-LSKEEKERLESLTKEISAAHNKLNITSDALEGITTTIDSLNAELES    842

BICD2_HUMAN      504 K----E--LK-KVSDV--A---G------ETQGSL---SV-AQ-D----E    526
                     |     |  |: |:|:|  |   |       |.|  |   || .| :    |
SMC3_YEAST       843 KLIPQENDLESKMSEVGDAFIFGLQDELKELQ--LEKESVEKQHENAVLE    890

BICD2_HUMAN      527 LVTFS-E-E-L-A----N--LYHHVCMCNNETPNRVM---LDYYREGQGG    563
                     |.|.. | | | |     |  |..   ..||:  .|::   ||.:   |..
SMC3_YEAST       891 LGTVQREIESLIAEETNNKKLLE---KANNQ--QRLLLKKLDNF---QKS    932

BICD2_HUMAN      564 AGRTS-PGGRTS-----PEARGR-RSPILLPKGLLAPEAGRADGGTGD-S    605
                     ..:|. .  :|:      .|.:.| |. |     ||| ||    |....| |
SMC3_YEAST       933 VEKTMIK--KTTLVTRREELQQRIRE-I----GLL-PE----DALVNDFS    970

BICD2_HUMAN      606 S-PSPGSSLPSPLSDPRRE--PM-NIYNLIAIIRDQIKHLQAAVDRTTEL    651
                     . .|  ..|...|:|...|  .: |: |..|.  :..|...   :|..:|
SMC3_YEAST       971 DITS--DQLLQRLNDMNTEISGLKNV-NKRAF--ENFKKFN---ERRKDL   1012

BICD2_HUMAN      652 SRQRIASQELGPAVDKDKEALMEE-ILKLK----SLL-ST--K-RE--Q-    689
                     : :| || ||     |:.|::: :: |:|||     :.: ||  | .|  :
SMC3_YEAST      1013 A-ER-AS-EL----DESKDSI-QDLIVKLKQQKVNAVDSTFQKVSENFEA   1054

BICD2_HUMAN      690 I-TTL--R-T---VL--K---AN--KQTAEVAL-A--NLKS---KYENEK    719
                     : ..|  | |   ::  |   ||  .::.:|.: |  | :|   | ::|
SMC3_YEAST      1055 VFERLVPRGTAKLIIHRKNDNANDHDESIDVDMDAESN-ESQNGK-DSE-   1101

BICD2_HUMAN      720 AMVT--E-TM---MKLRNE-L--KALKEDAATFSSLRAM-FATR-CD--E    756
                     .|.|  . ::   .| :|| |  :.|.....|..:: |: .|.: .|  .
SMC3_YEAST      1102 IMYTGVSISVSFNSK-QNEQLHVEQLSGGQKTVCAI-ALILAIQMVDPAS   1149

BICD2_HUMAN      757 -YITQLDEMQRQLAAAEDEK-KT-LNSLLR-M---AIQQKLALTQRLELL    799
                      |:  .||:.   ||. |:: :| :.:||: :    |  |.:..|.|.::|
SMC3_YEAST      1150 FYL--FDEID---AAL-DKQYRTAVATLLKELSKNA--QFICTTFRTDML   1191

BICD2_HUMAN      800 EL-D------HE---QT-----RR---G--R-A-K-A    813
                     :: |       :|   .|      |.   |  | : | |
SMC3_YEAST      1192 QVADKFFRVKYENKISTVIEVNREEAIGFIRGSNKFA   1228
```

Name 1: TAF1_HUMAN
Name 2: TAF1_DROME

Transcription initiation factor TFIID subunit 1 OS=Homo sapiens GN=TAF1 PE=1 SV=2
Transcription initiation factor TFIID subunit 1 OS=Drosophila melanogaster GN=Taf1 PE=1 SV=3

Needed for following interaction(s):
fly genetic RASK_HUMAN TAF1_HUMAN KRAS TAF1 RAS1_DROME TAF1_DROME

Length sequence 1: 1872
Length sequence 2: 2129

```
Sequence #1: TAF1_HUMAN
Sequence #2: TAF1_DROME
Length #1: 2207
Length #2: 2207
Matrix: BLOSUM62
Gap open: 2.0
Gap extend: 1.0
Length: 2207
Identity: 1067/2207 (48.35%)
Similarity: 1373/2207 (62.21%)
Gaps: 487/2207 (22.07%)
Score: 5198.00

TAF1_HUMAN        21 M-SD-TDSDEDSAGGGPFSLAGFLFGNINGAGQL----EGE---SV-LDD     60
                     | || :| ||.|.|.| ..|.|.|||||:..|:|     :||   .. .|.
TAF1_DROME         3 MESDNSD-DEGSIGNG-LDLTGILFGNIDSEGRLLQDDDGEGRGGTGFDA     50

TAF1_HUMAN        61 ECKKHLAG-LGALGLGSLITE---L-TA-----NEE--------LTGTDG     92
                     |.:::: | |..|||.|::.|   | .|      :||          ::.::|
TAF1_DROME        51 ELRENI-GSLSKLGLDSMLLEVIDLKEAEPPSDDEEEEDARPSAVSASEG     99

TAF1_HUMAN        93 -----AL---V-NDEGWVRSTEDAVDYSDINEVAEDESRRYQQTMGSLQP    133
                          ||   | .::|.|::.||:|||||.|::|| ..|   |     .|
TAF1_DROME       100 MSAFDALKAGVKREDGAVKAQDDAIDYSDITELSED-CPR---T----PP    141

TAF1_HUMAN       134 LCH--SDYDED-DYDA------DC---EDIDCK-LMPPPPPPPGPMKKDK    170
                       .  |.|| | : ||       :.   :| | | ||   |||..||:...
TAF1_DROME       142 --EETSTYD-DLE-DAIPASKVEAKLTKD-D-KELM---PPPSAPMRSGS    182

TAF1_HUMAN       171 DQDSITGE--KV-DFSSSSDSESEMGPQEATQAESEDG--KL-TLPLAGI    214
                     . ..|. |  |. |.||.|| :|      ::|  :|:|.  || | |||
TAF1_DROME       183 G-GGIE-EPAKSNDASSPSD-DS-----KST--DSKDADRKLDT-PLA--    219

TAF1_HUMAN       215 MQHDATKLLPS------VTELFPEFRPGKVLRFLRLFGPGK--NVPSVWR    256
                        |   :|||       |.||||:|||.|||||.|||||||  ::|.:||
TAF1_DROME       220 ---D---ILPSKYQNVDVRELFPDFRPQKVLRFSRLFGPGKPTSLPQIWR    263

TAF1_HUMAN       257 SARRKRKKKHRELIQ-EEQIQEVEC--SVES----EVSQK---SLWNYDY    296
                     .. |||::| |.  | .:| :....  | :|     |..:|    ||  : |
TAF1_DROME       264 HV-RKRRRK-RN--QSRDQ-KTTNTGGS-DSPSDTEEPRKRGFSL--H-Y    304

TAF1_HUMAN       297 APPPPPEQCLSDDEITMMAPVESKF-SQST---G-DID-KVTDTKPRVAE    340
                     |..|.|.:|:||||..::.  :  | |:..    | | : :.:|.||:||:
TAF1_DROME       305 AAEPTPAECMSDDEDKLLG--D--FNSEDVRPEGPD-NGENSDQKPKVAD    349

TAF1_HUMAN       341 WRYGPARLWYDMLGVPEDGSGFDYGFKLR--KTEHEPVIKSRMIEEFRKL    388
                     ||:|||:|||||.||:.|.||:|||||.:  .|..:|.:|    :| |::
TAF1_DROME       350 WRFGPAQIWYDMLEVPDSGEGFNYGFKTKAASTSSQPQLK----DE-RRV    394

TAF1_HUMAN       389 E--ENNGTD-LLADENFLMVTQLHWEDDIIWDGEDVK----HK-GTKPQR    430
                     :  |::.|  .:||:.|||:|||||||:|:|||.|:|     .| .:| ..
TAF1_DROME       395 KSPEDDVEDPSIADDAFLMVSQLHWEDDVVWDGNDIKAKVLQKLNSK-TN    443

TAF1_HUMAN       431 ASLAGWLPSSMTRNAMAYN--------V-----QQGF-AAT--LDDD---    461
                     |  |||||||.:|.|.|.|:         |      :||. |::  ...:
TAF1_DROME       444 A--AGWLPSSGSRTAGAFSQPGKPSMPVGSGSSKQGSGASSKKAQQNAQA    491

TAF1_HUMAN       462 KP-------WYSIFPIDNEDLVYGRWEDNIIWDAQAMPRLLEP-P-VLTL    502
                     ||       |||:||::||:|:|:|||.:|||||.:.:  | | ||||
TAF1_DROME       492 KPAEAPDDTWYSLFPVENEELIYYKWEDEVIWDAQQVSKV--PKPKVLTL    539

TAF1_HUMAN       503 DPNDENLILEIPDE----K-EEATSNSPSKESK-KESSLKKSRILLGKTG    546
                     ||||||:||.|||:     | .::| ..|.| .| ....:|||:|||||.|
TAF1_DROME       540 DPNDENIILGIPDDIDPSKINKST-GPPPK-IKIPHPHVKKSKILLGKAG    587

TAF1_HUMAN       547 VIK---EE--PQQNMSQPEVKDPWNLSNDEYYYPK-QQGLRGTFGGNIIQ    590
                     ||.    |:  |....| |: :||:|:|||.||.|| :..||...|||:||
TAF1_DROME       588 VINVLAEDTPPPPPKS-PD-RDPFNISNDTYYTPKTEPTLRLKVGGNLIQ    635

TAF1_HUMAN       591 HSIPAVELRQPFFPTHMGPIKLRQFHRPPLKKYSFGALSQPG-PHSVQPL    639
                     ||.|.||||.||.||||||:|||.|||||||||.|.::| . ||.|.||
TAF1_DROME       636 HSTPVVELRAPFVPTHMGPMKLRAFHRPPLKKYSHGPMAQ-SIPHPVFPL    684

TAF1_HUMAN       640 LKHIKKKAKMREQERQASGGGEMFFMRTPQDLTGKDGDLILAEYSEENGP    689
```

```
                      ||.|.||||.||.||.|||||::||||.|:||:|:|||::|||:.||:.|
TAF1_DROME        685 LKTIAKKAKQREVERIASGGGDVFFMRNPEDLSGRDGDIVLAEFCEEHPP    734

TAF1_HUMAN        690 LMMQVGMATKIKNYYKRKPGKDPGAP-DCKYGETVYCHTSPFLGSLHPGQ    738
                      |:.||||.:|||||||||..||.| | |..|||..:.|||||||.|||||
TAF1_DROME        735 LINQVGMCSKIKNYYKRKAEKDSG-PQDFVYGEVAFAHTSPFLGILHPGQ    783

TAF1_HUMAN        739 LLQAFENNLFRAPIYLHKMPETDFLIIRTRQGYYIRELVD-IFVVGQQCP    787
                      .:||.|||::|||||.|||...|||:||||..|:||. |: |:.|||:||
TAF1_DROME        784 CIQAIENNMYRAPIYPHKMAHNDFLVIRTRNNYWIRS-VNSIYTVGQECP    832

TAF1_HUMAN        788 LFEVPGPNSKRAN--THIRDFLQVFIYRLFWKSKDRPRRIRMEDIKKAFP    835
                      |:|||||||||||  |  |||||||||||||||:|.||||||:|||:|||
TAF1_DROME        833 LYEVPGPNSKRANNFT--RDFLQVFIYRLFWKSRDNPRRIRMDDIKQAFP    880

TAF1_HUMAN        836 SHSESSIRKRLKLCADFKRTGMDSNWWVLKSDFRLPTEEEIRAMVSPEQC    885
                      :||||||||||.||||||||||||||||:|.:||||:|||||||||||||
TAF1_DROME        881 AHSESSIRKRLKQCADFKRTGMDSNWWVIKPEFRLPSEEEIRAMVSPEQC    930

TAF1_HUMAN        886 CAYYSMIAAEQRLKDAGYGEKSF-FAPEEENEEDFQMKIDDEVRTAPWNT    934
                      |||:||||||||||||||||| | |||:|:::|:.|:|:||||:.|||||
TAF1_DROME        931 CAYFSMIAAEQRLKDAGYGEK-FLFAPQEDDDEEAQLKLDDEVKVAPWNT    979

TAF1_HUMAN        935 TRAFIAAMKGKCLLEVTGVADPTGCGEGFSYVKIPNKPTQQKDDKEPQPV    984
                      |||:|.||:|||||:::|.|||||||||||||::||||||.|:::|.||
TAF1_DROME        980 TRAYIQAMRGKCLLQLSGPADPTGCGEGFSYVRVPNKPTQTKEEQESQP-   1028

TAF1_HUMAN        985 KKTVTGTDADLRRLSLKNAKQLLRKFGVPEEEIKKLSRWEVIDVVRTMST   1034
                      |::||||||||||.|:.||:|||:|.||||||||||||||||||||||:||
TAF1_DROME       1029 KRSVTGTDADLRRLPLQRAKELLRQFKVPEEEIKKLSRWEVIDVVRTLST   1078

TAF1_HUMAN       1035 EQARSG-EGPMSKFARGSRFSVAEHQERYKEECQRIFDLQNKVLSSTEVL   1083
                      |:|::| || |.||:||:|||:||||||||||||||||||||:||:|:|||
TAF1_DROME       1079 EKAKAGEEG-MDKFSRGNRFSIAEHQERYKEECQRIFDLQNRVLASSEVL   1127

TAF1_HUMAN       1084 STD-TDSSSAEDSDFEEMGKNIENMLQNKKTSSQLSREREEQERKELQRM   1132
                      ||| .:||::|:||.||:|||:||||.|||||:||||||||.||:||.|.
TAF1_DROME       1128 STDEAESSASEESDLEELGKNLENMLSNKKTSTQLSREREELERQELLRQ   1177

TAF1_HUMAN       1133 L------LAAGS-AASGNNHRDDDTASVTSLNSSATGRCLKIYRTFRDEE   1175
                      |       .:|| .|.|...:||....:.:.|:.  ||.|:|.||||..:
TAF1_DROME       1178 LDEEHGG-PSGSGGAKGAKGKDDPGQQMLATNNQ--GRILRITRTFRGND   1224

TAF1_HUMAN       1176 GKEYVRCETVRK-PAVIDAYVRIRTTKDEEFIRKFA-LFDEQHREEMRKE   1223
                      ||||.|.||||: | |||||::|||||||:||::|| | |||.:|||::|
TAF1_DROME       1225 GKEYTRVETVRRQP-VIDAYIKIRTTKDEQFIKQFATL-DEQQKEEMKRE   1272

TAF1_HUMAN       1224 RRRIQEQLRRLKRNQEKEKL-------K----G-P-----P---------   1247
                      :|||||||||:|||||:|:|       |    | |     |
TAF1_DROME       1273 KRRIQEQLRRIKRNQERERLAQLAQNQKLQPGGMPTSLGDPKSSGGHSHK   1322

TAF1_HUMAN       1248 EK----K---P--KKMKERPDLKLKCGACGAIGHMRTNKFCPLY--YQTN   1286
                      |:    |   |  ||.|.:|||||||||||.:|||||||.||||  .|::
TAF1_DROME       1323 ERDSGYKEVSPSRKKFKLKPDLKLKCGACGQVGHMRTNKACPLYSGMQSS   1372

TAF1_HUMAN       1287 APPSNP-VA--MTEEQEEELEKTVIHNDNEELIKVEGTKIVLGKQLIE--   1331
                      ...|||  :|  . :||.|: |.|:   |:::|:.|:|||:.|..::::
TAF1_DROME       1373 LSQSNPSLADDF-DEQSEK-EMTM---DDDDLVNVDGTKVTLSSKILKRH   1417

TAF1_HUMAN       1332 SADE-VRR---KS-LVLKFPKQQLPPKKKRRVGTTVHCDYLNRPH-KSIH   1375
                      ..|: .||    .| ..||.|:..: .|||||||..:|||||.| | |:.:
TAF1_DROME       1418 GGDDGKRRSGSSSGFTLKVPRDAM-GKKKRRVGGDLHCDYLQR-HNKTAN   1465

TAF1_HUMAN       1376 RRRTDPMVTLSSILESII-NDMRDLPNTYPFHTPVNAKVVKDYYKIITRP   1424
                      ||||||:|.|||||| || |::|.:|:..||..||:||.|.|||:::|:|
TAF1_DROME       1466 RRRTDPVVVLSSILE-IIHNELRSMPDVSPFLFPVSAKKVPDYYRVVTKP   1514

TAF1_HUMAN       1425 MDLQTLRENVRKRLYPSREEFREHLELIVKNSATYNGPKHSLTQI-SQSM   1473
                      |||||:||.:|:|.|.|||.|.|.|.|:.||.||..||||:.:.| : :|.|
TAF1_DROME       1515 MDLQTMREYIRQRRYTSREMFLEDLKQIVDNSLIYNGPQSAYT-LAAQRM   1563

TAF1_HUMAN       1474 LDLCDEKLKEKEDKLARLEKAINPLLDDDDQVAFSFILDNIVTQ-KMMAV   1522
                      ...|.|.|.|:||||.|||||||||||||||||.|||.|.:.:| |.:
TAF1_DROME       1564 FSSCFELLAEREDKLMRLEKAINPLLDDDDQVALSFIFDKLHSQIKQL--   1611

TAF1_HUMAN       1523 PDSWPFHHPVNKKFVPDYYKVIVNPMDLETIRKNISKHKYQSR-ESFLDD   1571
                      |:||||..|||||.|.|||.||..|||||||.|||..|:|.|| | :|.|
TAF1_DROME       1612 PESWPFLKPVNKKQVKDYYTVIKRPMDLETIGKNIEAHRYHSRAE-YLAD   1660

TAF1_HUMAN       1572 VNLILA-NSVKYNGPESQYTKTAQEIVNVCY-QT-LTEYDEHLTQLEKDI   1618
                      :.|| | |..:|||.:::|||.:::|:.  | || |.|:.||..|||.:|
TAF1_DROME       1661 IELI-ATNCEQYNGSDTRYTKFSKKILE--YAQTQLIEFSEHCGQLENNI   1707

TAF1_HUMAN       1619 CTAK--EAALEEA-EL-ESL--DP--MTPGPYTPQP-PDLY-D-------   1651
                        ||  |.|.|.| |. |:.  |.  ...|.....| .| | |
TAF1_DROME       1708 --AKTQERARENAPEFDEAWGNDDYNFDRGSRASSPGDD-YIDVEGHGGH   1754

TAF1_HUMAN       1652 ---TNTSL--SMSRDA-SVFQDESNM--SVLDIPSATP---E--------   1682
                         :| |:  ||..:| |      |:.  :|.. | |.|    |
TAF1_DROME       1755 ASSSN-SIHRSMGAEAGS-----SHTAPAVRK-P-APPGPGEVKRGRGRP   1796

TAF1_HUMAN       1683 -KQ---VTQE--GED----GDG----D-LADEEEGTVQQPQASVLY---E   1714
                       ||   | :|  .::     |.|     | ||.....|     ||   |   |
TAF1_DROME       1797 RKQRDPV-EEVKSQNPVKRGRGRPRKDSLASNMSHT----QA---YFLDE   1838

TAF1_HUMAN       1715 DLLMS-EGEDD--EED----------AGS--DEEGD--N-P-------F-   1738
                      ||..| :.|||  |||          |.|  | :|:  | |       |
TAF1_DROME       1839 DLQCSTDDEDDDEEEDFQEVSEDENNAASILD-QGERINAPADAMDGMFD   1887

TAF1_HUMAN       1739 -----SAI-----QLSES--G-SDS----D--V---GSGGIRPKQPRMLQ   1766
                           :.|     |::|.  | .||    :  |   |.||...:| :  |
TAF1_DROME       1888 PKNIKTEIDLEAHQMAEEPIGEDDSQQVAEAMVQLSGVGGYYAQQ-Q--Q   1934

TAF1_HUMAN       1767 ENTRMDME-N-EES--M-MSY-EGDG-GEASHGLED--SNISYGSYEEPD   1807
                      :.: ||:: | :.|  : | : :... ||.| .|:.  :|  :.|:|:.|
TAF1_DROME       1935 DES-MDVDPNYDPSDFLAM-HKQRQSLGEPS-SLQGAFTN--FLSHEQDD   1979

TAF1_HUMAN       1808 --PKSN-TQ-DTSFSSIG---GYEVS-------E-----------EEE-   1830
                        | .| .: .||.:| |   |.:.|       |           :::
TAF1_DROME       1980 NGP-YNPAEASTSAAS-GADLGMDASMAMQMAPEMPVNTMNNGMGIDDDL   2027

TAF1_HUMAN       1831 D--EEEEEQRSGPSVLSQVHLSEDE--EDSEDF---H-SI---AGDSDL-   1868
                      |  |.:||. .|    |:|.:.: |  :|. |:   | .:   |..|.:
TAF1_DROME       2028 DISESDEED-DG----SRVRIKK-EVFDDG-DYALQHQQMGQAASQSQIY   2070

TAF1_HUMAN       1869 --D-SDE   1872
                        | |:|
TAF1_DROME       2071 MVDSSNE   2077

--------------------------------------------
Name 1: M3K1_HUMAN
Name 2: BCK1_YEAST

Mitogen-activated protein kinase kinase kinase 1 OS=Homo sapiens GN=MAP3K1 PE=1
SV=4
Serine/threonine-protein kinase BCK1/SLK1/SSP31 OS=Saccharomyces cerevisiae (str
ain ATCC 204508 / S288c) GN=BCK1 PE=1 SV=1

Needed for following interaction(s):
yeast   genetic M3K1_HUMAN      RASK_HUMAN      MAP3K1  KRAS    BCK1_YEAST
IRA2_YEAST

Length sequence 1:      1512
Length sequence 2:      1478
Sequence #1: M3K1_HUMAN
Sequence #2: BCK1_YEAST
Length #1: 1774
Length #2: 1774
Matrix: BLOSUM62
Gap open: 2.0
Gap extend: 1.0
Length: 1774
Identity: 503/1774 (28.35%)
Similarity: 767/1774 (43.24%)
Gaps: 699/1774 (39.40%)
Score: 1704.00

M3K1_HUMAN        113 P--HGAASRGGAHLTESVAAPDSGASSPAAAEPGEKRAP----AAEPSPA    156
```

```
                     |  ...|  |.|| |.| .: ||. ||   .:.|.:  |    |:..|.:
BCK1_YEAST         2 PFLRKIA--GTAH-THS-RS-DSN-SS---VKFGHQ--PTSSVASTKSSS    40

M3K1_HUMAN       157 AAPAGREMENKETLKGLHKMDDRPEERMIREKL-KATCMP-AWKHEWLER   204
                     .:|  | ..::   |.::  ||       ||.:. ..|  | :...::.|
BCK1_YEAST        41 KSP--R-ATSR---KSIY--DD------IRSQFPNLT--PNSTSSQFYE-    73

M3K1_HUMAN       205 RNRRGPVVVKPIP-VKGDGSEMNHLAA---ESPGEVQASAASPAS-KGRR   249
                      :.  ||:.:... .. |    :|::|   |:|   .|..: :| |...
BCK1_YEAST        74 -ST--PVIEQSFNWTT-D----DHISAGTLENP----TSFTN-SSYKNDN   110

M3K1_HUMAN       250 SPSPGN-SPSGRTVKSESPGVRRKRVSPVPFQSGR-ITPPRRAPSPDGFS   297
                      .||  : |.| |  || |.|    ..|.:.:.|.  : |.      |.|
BCK1_YEAST       111 GPS--SLSDS-R--KS-SGG---NSVNSLSFD--KLIL------SWD---   140

M3K1_HUMAN       298 PYSPEE-TNRRV-N--KVMR--ARLYLL----QQI-GPNSF--LIGGDSP   334
                      |..|:| |..|| :  | ..  ...::|     .|: | :.|  |:   :.
BCK1_YEAST       141 PTDPDEWTMHRVTSWFK-FHDFPESWILFFKKHQLFG-HRFIKLL---AY   185

M3K1_HUMAN       335 DN--KYRVFIGPQNCSCARGTFCIHLLFVMLRVFQLEPSDPMLWRKTL-K   381
                     ||  .|..:: ||. ..|  ::         .| || :    :| :||: |
BCK1_YEAST       186 DNFAVYEKYL-PQT-KTA--SY--------TR-FQ-Q----LL-KKTMTK   216

M3K1_HUMAN       382 NFEV-ESLFQKYHSR-RS-SRIKAPSRN---TIQKFVSRMSNSHT---LS   422
                     |  | .|      |.| :| |::|: ||:   :| |  |::.||.:   :
BCK1_YEAST       217 N--VTNS-----HIRQKSASKLKS-SRSSSESI-K--SKLKNSKSQEDI-   254

M3K1_HUMAN       423 SSSTSTSSSENSIKDEEEQMCPICLLGMLDEESLTVCEDGCRNKLH----   468
                     |:|.||  ||:::.        | ...|    .|.|   |. :|.||
BCK1_YEAST       255 SNSRST--SESALS-------P-TKSG----PSKT---DE-KNFLHSTST   286

M3K1_HUMAN       469 HH-CMSIWAEEC-RRNREPLICPLCR-SKWRSHDFYSHELSSPVDSPSSL   515
                     |. ..|  |... ||:   .| .| | |.  |    |: .|| ..|||::
BCK1_YEAST       287 HQKTKS--ASSLYRRS---FI-SL-RGSS--S----SN-ASS-AKSPSNI   321

M3K1_HUMAN       516 R-A--AQ-----Q--QTV-QQ-QPLAG----S--RR-----NQESN-FN-   540
                     : :  |:      :  .|: :. .|.|.     |  ||      :.||: .|
BCK1_YEAST       322 KLSIPARPHSIIESNSTLTKSASPPASPSYPSIFRRHHKSSSSESSLLNS   371

M3K1_HUMAN       541 LTHYGT---QQIPPAYKDLAEPWIQVFG--M--E-LV-GCLFSRNW--NV   579
                     |  :|:   :: .|. |  ..|  |  |  :  | |. |  .|:::  ||
BCK1_YEAST       372 L--FGSGIGEE-APT-K--PNP--Q--GHSLSSENLAKG--KSKHYETNV   409

M3K1_HUMAN       580 REMALRR--L--SHDVSGALLLAN---GES-TG----NSGG--SSGSSPS   615
                      ...|::  |  |.| .|.|.  |   .:| .|     |:..  :|..:||
BCK1_YEAST       410 -SSPLKQSSLPTSDD-KGNLW--NKFKRKSQIGVPSPNTVAYVTSQETPS   455

M3K1_HUMAN       616 GGATSGSS-QT-SI-SGDV-VEA-CCS---V-------LSMVCA-D-PVY   648
                       ..|.|| .| :: :.|| :.: ..|    :        |.::.. | |
BCK1_YEAST       456 --LKSNSSTATLTVQTADVNIPSPSSSPPPIPKTANRSLEVISTEDTP--   501

M3K1_HUMAN       649 KV--YVAALK-TLRAMLVYTP-CHSL-AER---IKL--QR------LL-Q   681
                     |:  ..|:.| |       | | |  : .::   :.:  |:       || |
BCK1_YEAST       502 KISSTTASFKET------Y-PDC--INPDKTVPVPVNNQKYSVKNFLLDQ   542

M3K1_HUMAN       682 ---PV----V-DT----ILV-KCADANSRTSQLSISTLLELCKGQAGELA   718
                        |:    : |:    ||| |  | |  .| . :.  |.| |..| :|:
BCK1_YEAST       543 KFYPLKKTGLNDSENKYILVTK--D-N--VS-F-VP--LNL-KSVA-KLS   581

M3K1_HUMAN       719 VG-RE-IL-KAGSIGIG--GV-----DYVLNC-ILG----NQT-ESNNW-   751
                      . :| .| |   :||.  .|      |:  :| | |     :.| |   :
BCK1_YEAST       582 -SFKESALTK---LGINHKNVTFHMTDF--DCDI-GAAIPDDTLE---FL   621

M3K1_HUMAN       752 -QEL-L---GRLCLI-DRLLLE---FPAEFYPHIVS-TDVSQAEPVE-IR   790
                      :.| |   |:: .| |::.|:   .||   | :.| .:|    |:: ::
BCK1_YEAST       622 KKSLFLNTSGKI-YIKDQMKLQQKPKPA---P-LTSENNV----PLKSVK   662

M3K1_HUMAN       791 YK---K-----LL------SLLTFA--LQSIDNSH-SMVGKLS-RRIY--   820
                     .|   :      |:       |::|.:  :.|.| .| |  |  | || |
BCK1_YEAST       663 SKSSMRSGTSSLIASTDDVSIVTSSSDITSFD-EHAS--G--SGRR-YPQ   706

M3K1_HUMAN       821 LSS---ARMVT-TVP--HV-F-S-KLLEMLSVSSSTHFTRMRRRLMAIAD   861
                     ..|   .| |: |.|  .: : : |  |:||      |     ...     |.
BCK1_YEAST       707 TPSYYYDR-VSNTNPTEELNYWNIK--EVLS-----H----EEN----AP   740
```

```
M3K1_HUMAN       862 E-V-EIAEAIQLGVEDTLDGQQDSFLQASVPNNYLET--TEN-S-SP-EC   904
                     : | :.:..::|.:.|  .|   |.|  ::|      |  ||| | |. :
BCK1_YEAST       741 KMVFKTSPKLELNLPD--KG---SKL--NIP-----TPITENESKSSFQ-   777

M3K1_HUMAN       905 TVHLEKTGKGLCATKL--S---AS--SE-DIS-ERLASISVGPSSSTT--   943
                      | |.| .:|   |::  :   .|  :: ::: :|.|. .  |.::|:
BCK1_YEAST       778 -V-LRK-DEG---TEIDFNHRRESPYTKPELAPKREAP-K--PPANTSPQ   818

M3K1_HUMAN       944 TTTTTTEQPKP--MVQ--TK-GRPHSQCLNSSPLSHHSQLMFPALSTP--   986
                     .|.:|::|.||  :|:  || .|  |:  .|.||.  .||    ||:|
BCK1_YEAST       819 RTLSTSKQNKPIRLVRASTKISR--SK--RSKPLP--PQL----LSSPIE   858

M3K1_HUMAN       987 -SSSTP-SVPAGTA--TDVSKHRLQGFIPCRIP--SASPQTQRKFSLQFH  1030
                      |||:| |:   |:  |..|.|.|   || : |  .|: ...|:  |
BCK1_YEAST       859 ASSSSPDSL---TSSYTPASTHVL---IP-Q-PYKGAN-DVMRR--L---   894

M3K1_HUMAN      1031 RNCPENKDSDKLSP-V-FTQ--SR---PLPSSN-I-HRPKP--SRPTPGN  1069
                     :.   ::||...|| : ..|  :|   .:.:|| | :.|.|  .|   ||
BCK1_YEAST       895 KT---DQDSTSTSPSLKMKQKVNRSNSTVSTSNSIFYSPSPLLKR---GN   938

M3K1_HUMAN      1070 TSKQ-------GD--PSKNSMTL-D---L-NS-SSKCDDSFGCSSNSSNA  1104
                      ||:          .|   .:|.:|. |   : :| .|  ||.   || ||:.
BCK1_YEAST       939 -SKRVVSSTSAADIF-EENDITFADAPPMFDSDDS--DDD---SS-SSDD   980

M3K1_HUMAN      1105 VIPS-DETVFTPVE---E--K--CRLDVN--T---EL--NSSIEDLLEAS  1139
                     :|.| .:|  .| |   |  |   :.| |  |   |:  :|..:|.:|..
BCK1_YEAST       981 IIWSKKKT--AP-ETNNENKKDE-KSD-NSSTHSDEIFYDSQTQDKMERK  1025

M3K1_HUMAN      1140 M---PSSDTTV--T----F------K--SE-VA----------VLSPEK-  1160
                     |   ||.: .|  .     |       |  :| :|           :||| |
BCK1_YEAST      1026 MTFRPSPE-VVYQNLEKFFPRANLDKPITEGIASPTSPKSLDSLLSP-KN  1073

M3K1_HUMAN      1161 -A----E----N-----DDTY---KDDVN--H---NQ-KC-KE-K-ME--  1182
                      |    |    :      |.:|   :|.:|  :   || |. |. | :.
BCK1_YEAST      1074 VASSRTEPSTPSRPVPPDSSYEFIQDGLNGKNKPLNQAKTPKRTKTIRTI  1123

M3K1_HUMAN      1183 AEEEEALAIAMAMSASQDALPIVPQLQVEN----GEDIIIIQQDTPETLP  1228
                     |.|       | :: |.:::   | :|:.:|     |..::.:   | |
BCK1_YEAST      1124 AHE------A-SL-ARKNS---V-KLKRQNTKMWGTRMVEV---T-E---  1154

M3K1_HUMAN      1229 GH----TKAK--Q-PYREDTEWLKGQQIGLGAFSSCYQAQDVGTGTLMAV  1271
                     .|     .|||  : .|:| ..|:|||:.||.|:.|.:.|...:|.||.:|||
BCK1_YEAST      1155 NHMVSINKAKNSKGEYKE-FAWMKGEMIGKGSFGAVYLCLNVTTGEMMAV  1203

M3K1_HUMAN      1272 KQVTYVRNTSSEQEEV---VEALREEIRMMSHLNHPNIIRMLGATCE-KS  1317
                     |||. |...||:|.:   |||||.|:..:.:|.||:::.||  .| |:
BCK1_YEAST      1204 KQVE-VPKYSSQNEAILSTVEALRSEVSTLKDLDHLNIVQYLG--FENKN  1250

M3K1_HUMAN      1318 N-YNLFIEWMAGGSVAHLLSKYGAFKESVVINY-TEQLLRGLSYLHENQI  1365
                     | |:||:|::|||||..|:..|||.|.|.: :|.: |.|:|:||:|||...|
BCK1_YEAST      1251 NIYSLFLEYVAGGSVGSLIRMYGRFDEP-LIKHLTTQVLKGLAYLHSKGI  1299

M3K1_HUMAN      1366 IHRDVKGA-NLLIDSTGQRL-RIADFGAAARLASKGTGAGE-F-QGQL-L  1410
                     :|||:| | |||:| | ||| .:| .||      : : ...: :
BCK1_YEAST      1300 LHRDMK-ADNLLLDQDG--ICKISDFG-ISR-KSK-----DIYSNSDMTM  1339

M3K1_HUMAN      1411 -GTIAF-MAPE-V-LRGQQYGRSCDVWSVGCAIIEM-ACAKPPW-NAEKH  1454
                      ||: | |||| | .: |.|....|:||:||.::|:|| .|.|| |.|.
BCK1_YEAST      1340 RGTV-FWMAPEMVDTK-QGYSAKVDIWSLGCIVLEMFA-GKRPWSNLEV-  1385

M3K1_HUMAN      1455 SNHLALIFKIASATTAPSIPSH-LSP-----GLR---DVALRCLELQPQD  1495
                        :|.:|||..:.:|||.||.. | |      | |   | |  |.|:.|:.
BCK1_YEAST      1386 ---VAAMFKIGKSKSAPPIPEDTL-PLISQIG-RNFLD-A--CFEINPEK  1427

M3K1_HUMAN      1496 RPPSRELLKHP---V-----FRTT   1511
                     ||.:.|||.||   |      |::|
BCK1_YEAST      1428 RPTANELLSHPFSEVNETFNFKST   1451

-------------------------------------------
Name 1: DCTN1_HUMAN
Name 2: NIP80_YEAST

Dynactin subunit 1 OS=Homo sapiens GN=DCTN1 PE=1 SV=3
```

```
Protein NIP100 OS=Saccharomyces cerevisiae (strain ATCC 204508 / S288c) GN=NIP10
0 PE=1 SV=2

Needed for following interaction(s):
yeast   genetic PTTG1_HUMAN     DCTN1_HUMAN     PTTG1   DCTN1   SECU_YEAST
NIP80_YEAST

Length sequence 1:      1278
Length sequence 2:      868
Sequence #1: DCTN1_HUMAN
Sequence #2: NIP80_YEAST
Length #1: 1343
Length #2: 1343
Matrix: BLOSUM62
Gap open: 2.0
Gap extend: 1.0
Length: 1343
Identity: 352/1343 (26.21%)
Similarity: 544/1343 (40.51%)
Gaps: 608/1343 (45.27%)
Score: 1042.00

DCTN1_HUMAN       11 RTPSG----SRMSAEASARPLR--VGSRV-EVIGKGHRGTVAYVGATLFA     53
                     |. :|    :.|. :.|   |:  |  .| |:  || |  |.::|.|.||
NIP80_YEAST        2 RN-AGVQVDTNMQ-KIS---LQDTV--LVNEM--KG-R--VKFIGETQFA     39

DCTN1_HUMAN       54 TGKWVGVILDEAK--GKNDGTVQG-RKYFTCD--E-----G-HGIFVRQS     92
                     .|.|.|:.||  |  |||||:..| | ||..|  :     | :|:|.::.
NIP80_YEAST       40 KGIWYGIELD--KPLGKNDGSANGIR-YFDIDLKKANSNGGYYGLFCKKD     86

DCTN1_HUMAN       93 QIQVFEDGADTTSPETPDSSASKVLKREGTDTTAKTSKLRGLKPKKAPTA    142
                     .:| |.         : ||          :  |   :.|.|.|    .|  |
NIP80_YEAST       87 TLQ-FY--------K-PD---------D--D---EHSLLNG----NA--A    106

DCTN1_HUMAN      143 RKTTTRRPKPTRPASTGVAGASSSLGPSGSASAGELSSSEPSTPAQTPLA    192
                     ::|.    |              :|          :: .:| |      ||
NIP80_YEAST      107 QETI----K--------------NL---------QV-KCE-S------LA    121

DCTN1_HUMAN      193 APIIPTPVLTSPGAVPPLPSPSK-E-EEGLRAQVRDLEEKL---ET-LRL    236
                     :.      |..   :         | | .| |:..|    |||   || | |
NIP80_YEAST      122 SK------LNK---I-------KIENHE-LKTSV----EKLSTNETVL-L    149

DCTN1_HUMAN      237 -K--RAEDKAKL-KELEKHKIQLEQVQEWKSKMQEQQADLQRRLKEARKE    282
                      |  |. |  || |||   |      | | ...|             | ..
NIP80_YEAST      150 SKISRL-D--KLVKEL---K-----V-E-NGNM-----------K-TH--    172

DCTN1_HUMAN      283 AKEALEAKERYMEEMADTADAIEMA-TLDK-EMAEERAESLQQEVEALKE    330
                         |   :.: ..:.|.:|:: || .||| .:. ||:..||    .|
NIP80_YEAST      173 ----L---DNF-NHLLDASDSV-MAPDLDKGTLL-ERSHLLQ----GL--    206

DCTN1_HUMAN      331 RVDELTTDL--EILKA-EIEEKGSDGAASSYQLKQLEEQNARLKDALVRM    377
                      :|:  |.|  :  || :::|   |        |  ||| |.:|
NIP80_YEAST      207 -LDQ--TKLSYD--KAMKVQE---D-------L--LEE-NTQL-------    231

DCTN1_HUMAN      378 RDLSSSEKQEH-VKLQKLMEKKNQELEVVRQQRERLQEELSQAESTI-DE    425
                       |   |  |: |     | .||..:|.:   |   ||    |..:|| |
NIP80_YEAST      232 --L---E--ENAV----L-SKKISDLGL---Q---LQ----QTNNTIGD-    258

DCTN1_HUMAN      426 LKEQVDAALGAEEMVEMLT-DRNLNLEEKVRELRETVGDLEAMNEMNDEL    474
                     |..|::|...:..:|:.|| | |: |      |  | .:::|:|  | ||
NIP80_YEAST      259 LALQIEAQSKSSNIVDKLTND-NI-L------L--T-SNIKALN--N-EL    294

DCTN1_HUMAN      475 QENARETELELREQLDMAGARVREAQKRVEAAQETVADYQQTI-KKYR-Q    522
                          | ||:.:|:||       | ..|:     |   |:| : ::.| |
NIP80_YEAST      295 -----E-ELQAKEKLD-------E-NLRI-----T---YEQ-LEQELRLQ    321

DCTN1_HUMAN      523 LTAHLQDVNRELTNQQE-A-S-VERQQQPPPETFDFKIKFAETKAHAK--    567
                     | ::||..   |.|::| | : :|...: ...|.: .|: |:| :| |
NIP80_YEAST      322 L-SNLQSA---LENEKEIAGTYIEENSR-LKATLE-SIE-AKT-SH-KFQ    362

DCTN1_HUMAN      568 AIEME---LRQMEVAQANRHMSLLTAF--M--PDSFLRPGGDHDCVLVLL    610
                     ::|::   | |.|:.| |:   ||..|  :  |  |.:|   |   |. .
NIP80_YEAST      363 SLELKVNTL-QEELYQ-NK---LLKKFYQIYEP--FAQP---H---LA-A    398
```

```
DCTN1_HUMAN      611 LMPRL--ICKAELIRKQAQEKF-ELSENCSE-R--PGLRGAAGEQLSFAA    654
                     |..:|  :  ||:|  :: |.| :| ||. | .    |: .. ..:|:|
NIP80_YEAST      399 LSSQLQYL--AEVI--ES-ENFGKL-ENI-EIHII--LK-VL-SSISYA-    436

DCTN1_HUMAN      655 GL-VYSLSLLQAT--LHRYEHALSQC-SVDVYKKVGSLY-PE-M----SA    694
                      | :|:   ::.|   | .|..| || .|:: ..: |:: .| :    |:
NIP80_YEAST      437 -LHIYT---IKNTPD-H-LETTL-QCFKVNI-API-SMWLSEFLQRKFSS    477

DCTN1_HUMAN      695 -HER--SL-DFLIE-LLHK--DQLDETVNVEPLTKAIKYYQHLYSIHLAE    737
                      .|.  |: .|| |   :|  |: |.|: :  | | |      |:.| | |
NIP80_YEAST      478 KQETAFSICQFL-ED--NKFLDK-DVTL-I--L-K-I-----LHPI-L-E    511

DCTN1_HUMAN      738 Q--PEDCTMQLADHIKFTQSAL---D--CMSVEVGRLRAFLQGGQEATDI    780
                     .  |:   : || .:: |.|..    |  |:   :|.|  :     |.: :
NIP80_YEAST      512 TTVPK---L-LA-FLR-TNSNFNDNDTLCL---IGSL--Y-----ERS-L    544

DCTN1_HUMAN      781 ALLLR-D-L----ETSCSDIRQFCKKIRRRMPGTDAPGIPAALA--FGPQ    822
                     :|:.| | |     |.|..|.|.|   :   .|..|.. :.:.|.  |
NIP80_YEAST      545 SLIARIDKLIGKEEISKQDNRLF---L---YPSCDIT-LSSILTILF---    584

DCTN1_HUMAN      823 VSDTL-L--DCRKHLTWVVAV--LQEVAAAAAQLIAPLAENEGLLVAALE    867
                      ||.| |  | .|.::   ::  | ||   ..|.|..|.||  :.:  ..
NIP80_YEAST      585 -SDALFLRQD-YKRIS---SLKKL-EV---FFQGIESLLEN--ITI--FP    621

DCTN1_HUMAN      868 ELAFKASEQIYGTPSSSPYECLRQSCNILISTMNKLATAMQEGEY-D---    913
                     |   :.|:|   | |.|  |  .| |||      |       ||.: :
NIP80_YEAST      622 E---QPSQQ---T-SDS--E--SQ-CNI------K-------EGNFSNSLL    647

DCTN1_HUMAN      914 AERPPSKPPPVE-LRAAALRAEI-TDAEGLGL-KLEDR-ETVI-KEL-KK    957
                     ::| .::     | :|    |: |: ...|.: | :||.: :.:| ::| :|
NIP80_YEAST      648 SDR-LNE----ENIR---LK-EVLVQKENM-LTELETKIKIIIGRDLERK    687

DCTN1_HUMAN      958 SLKIKGEELSEANVRLSLLEK-KLDSAAKDADERIEKVQTRLEETQALLR   1006
                     :|     ||    |:: :|  | :|::  |: :|...|  |    |   :|.
NIP80_YEAST      688 TL----EE----NIK-TL--KVELNN--KN-EENCGK--T---E---ILN    715

DCTN1_HUMAN     1007 K-KEKEFE-----ETMD-AL-Q-AD---IDQ--LEAEKAELKQRLNSQSK   1042
                     | ||:.|.      :.|: .| | .|   :::  |:.||.:   |:|..|:
NIP80_YEAST      716 KLKEENFNLVNRLKNMELKLYQIKDNNTLNKIYLDREKVD---RVNLVSE    762

DCTN1_HUMAN     1043 RTI-EGLRGPPPSGIATLVSGIAGEEQQRGAIPGQAPGSVPGPGLVKD-S   1090
                       | | ||        .|:...|  :||:|  :       |:        | |
NIP80_YEAST      763 --IME-LR-------ETIRRQI--KEQKR--V------SI-------DFS    785

DCTN1_HUMAN     1091 PLLLQQISAMRLHISQLQHENSILKGAQMKASLASLPPL--HVAKLSHEG   1138
                       .|.::.|     :      ||    |  |            |.  |:   :|
NIP80_YEAST      786 --WLDELPA----V-----EN---K--Q---------PFKEHI---NH--    805

DCTN1_HUMAN     1139 PGSELPAGALYRKTSQL-LETLNQLSTHTHVVDITR-TSPAASKSPSAQLM   1186
                       |       |  .|  | :|..|.:|| :.::|: : ..|.|:.   :|.
NIP80_YEAST      806 --S------L--DT--LGIEMFNFVST-SRILDL-KLDQPLAED---ELW    838

DCTN1_HUMAN     1187 -EQ----VAQLKSLSDTVEKLKDEV-LKETVSQRPGATVPTDF   1223
                      |:    ::.||       .|.|: : ||   ||.   .| | :
NIP80_YEAST      839 HERDHSYISYLK------RKRKN-IRLK---SQN---VV-T-Y    866

--------------------------------------------
Name 1: AQR_HUMAN
Name 2: SEN1_YEAST

Intron-binding protein aquarius OS=Homo sapiens GN=AQR PE=1 SV=4
Helicase SEN1 OS=Saccharomyces cerevisiae (strain ATCC 204508 / S288c) GN=SEN1 P
E=1 SV=2

Needed for following interaction(s):
yeast   genetic AQR_HUMAN       BLM_HUMAN       AQR     BLM     SEN1_YEAST
SGS1_YEAST

Length sequence 1:      1485
Length sequence 2:      2231
Sequence #1: AQR_HUMAN
Sequence #2: SEN1_YEAST
Length #1: 2164
Length #2: 2164
```

```
Matrix: BLOSUM62
Gap open: 2.0
Gap extend: 1.0
Length: 2164
Identity: 570/2164 (26.34%)
Similarity: 849/2164 (39.23%)
Gaps: 952/2164 (43.99%)
Score: 1759.00

AQR_HUMAN          5 AQPK-KIV---A-----PTVSQINAEFVTQLACKYW---A--PHIKKK-S     39
                     |:.| |::   |     |. ..: |:| ..:.|: |   |  | |.:. |
SEN1_YEAST       125 ARGKCKMLQHFAIQRHVPH-EHV-AKF-NDIVCQ-WRVEAVFP-ILRNIS    169

AQR_HUMAN         40 PFD---IKV---IED-IYE-----------KEIVKSRF-AIRKIMLLEFS     70
                     ..|   |.:   ||. :||           |:: |:.| ||.|.    |
SEN1_YEAST       170 VNDNTGINITNEIETAMYECLCNPHMLRLNKQL-KATFEAIFKF----F-    213

AQR_HUMAN         71 QYLE-NY-LWMNY-SPEVSSKAYLMS--I-C-CMVNE--KFRENVPAW-E    110
                      | : .: | ::. :| :|.|.:: |  | | |   |  | .||  .| .
SEN1_YEAST       214 -Y-DTKHRL-LDVTNP-LSIKTFI-SGVIFCWC---EGSK-EEN--EWSR    252

AQR_HUMAN        111 IFKKKPDHFPFF---FKHI-LKAALAET-D--GE-FSLHEQTVLLLFLDH    152
                     .|.|  |   .:   | || | :.|  | |  .| : :|     :|||.:
SEN1_YEAST       253 AFLK--D---LYSRNF-HINL-SNL--TPDIIEEVY-IH-----ILFLQN    287

AQR_HUMAN        153 -CFN-SLEVDLIRSQV-QQLISLPMWMGL----------QLAR-LELELK    188
                      . | : |: :: ||. .:|  ||:: .|          |:.: :| .||
SEN1_YEAST       288 PA-NWT-EI-VV-SQFWSRL--LPVF-NLFDKDVFIEYFQVPKNVE-SLK    329

AQR_HUMAN        189 KT---PKLR---KFW-N-LIKK-NDEKMDP-E--AR------EQAY-QER    219
                     ||   | |.   |.| | |.|. :| |  | :  .|      .: : .|
SEN1_YEAST       330 KTFKFP-LEPIFKMWYNHLSKSYHD-K--PLDFLLRGLTMFLNK-FGSE-    373

AQR_HUMAN        220 RFLSQLIQKF-I-SVL-----K-SVP--L---SE-PVTMDKVHYCERFIE    255
                      |.|: |:.| . |:|     : |.|  |   .: |: ::  |..|.:.:
SEN1_YEAST       374 -FWSK-IEPFTFHSILDIIFNRDSFPIKLIKIQDNPI-VE--HQTEVYFQ    418

AQR_HUMAN        256 L--MI-DL--------EALLPT-R----R---W-F-NTILD-DSHL-LV-    281
                      |  .: ||        .||.|: |    |   . | ..|.: .| | .:
SEN1_YEAST       419 LTGSVTDLLSWTLPFYHALSPSKRIQMVRKVSMAFLRIIANYPS-LKSIP    467

AQR_HUMAN        282 -HCYLSN----LVR---------R-------E-E-------DGH-LFSQ-    300
                      .| |.|    |:|         |       | |       |.. |.:.
SEN1_YEAST       468 KAC-LMNSATALLRAVLTIKENERAMLYKNDEFETVLLTKTDSRALLNNP    516

AQR_HUMAN        301 LL-D-MLK-------FYTGFEINDQTG--NA-L-TENEMT---TIHYDRI    334
                     |: | :::       ||.|  :    |  :| : |...|.   .|.:| |
SEN1_YEAST       517 LIQDIIIRSASNPNDFYPG--L----GAASASVATSTMMVLAECIDFD-I    559

AQR_HUMAN        335 TSLQRAAFAH--FPELY------DFALS-NVAE-VDTRE-SLVK-FF-GP    371
                       |   ...|  | :||      :..:| ||.| | |.: .| : |. ||
SEN1_YEAST       560 --L---LLCHRTF-KLYSGKPISEIPISTNVLENV-TNKIDL-RSFHDGP    601

AQR_HUMAN        372 -LSSNTLHQVASYL--C--LLPTLPKN----E-DTTFDKEFLLELLVS-R    410
                      |:...|  | | |  .  || .:|.|     | ....:::||  || | |
SEN1_YEAST       602 LLAKQLL--V-S-LKNINGLL-IVPSNTAVAEAHNALNQKFL--LL-STR    643

AQR_HUMAN        411 -HERRISQI--QQLNQMPLYPTEKI---IW------DENI--VPTE--Y-    443
                      .| :.:.|  .||::: | ..|..   .|      |:::  ..|.   |
SEN1_YEAST       644 LME-KFADILPGQLSKI-L-ADEDASQGFWSCIFSSDKHLYQAATNILYN    690

AQR_HUMAN        444 -Y--SG--EGCLALPKLNLQFLTLHDYLLRNFN--LFRL---ESTYE---    480
                      :  .|  ||.||:  ||.. ||::   |:|.|  |.||   | .||
SEN1_YEAST       691 TFDVEGRLEGILAI--LNSN-LTVN---LKNINVMLQRLINCE-FYEPCP    733

AQR_HUMAN        481 --IR--QD-IE---D--S-V--S-R-MKPWQS---EYGGVVFGGWAR--M    510
                       :|  .| :.   |  | |  : : :|. |:   |:  :.|  |..  :
SEN1_YEAST       734 RAVRVLMDVVSAFVDPISGVFANFQTLKS-QNTEKEF--LKF--WESCWL    778

AQR_HUMAN        511 -AQPIVAFTVVE-VAK---PNIGENW--PT----R--V---R--ADV---    539
                      ...|..|| :: .:|   ..: ||:  .|    |  |   |  :|:
SEN1_YEAST       779 FLDTIYKFT-LKWASKYDYSEL-ENFTKDTLDLSRSLVDSFREFSDILHD    826

AQR_HUMAN        540 -TIN--LNVRDHIKD--EWEGLRKHD-V----CF-LI--T--------VR    568
                       |.|  |||.:..|:  .|  ||..| |     |. ||  |         |:
SEN1_YEAST       827 QTKNLLLNVLETFKNMLYW--LRLSDEVLLESCVRLIISTSDLAHEKHVK    874

AQR_HUMAN        569 --PT------KPYGTKFDRRRPF---I-EQ----VGL----VYVRGC--E    596
                       .:      | |.:|  .:| |   : ||    : |    ::.:..  |
SEN1_YEAST       875 VDDSLVEMMAK-YASK--AKR-FSNKLTEQQASEI-LQKAKIFNKALTEE    919

AQR_HUMAN        597 I----QGMLDDK-----GRVIE--DG-P-EP--RPNL-------RGESRT    624
                     :    :....:|     |:||:  |. | .|  .|:|       ..|||.
SEN1_YEAST       920 VATEAENYRKEKELSRLGKVIDLTDSVPASPSLSPSLSSTIASSSAESRA    969

AQR_HUMAN        625 -F--RVFLD------PNQYQQDMTN--TIQ---NGAEDVYETFN-I--I-    656
                      :  |..|.      |...|..:|:  |.|   | |: ::.| . :  :
SEN1_YEAST       970 DYLQRKALSSSITGRPRVAQPKITSFGTFQSSAN-AK-LHRT-KPVKPLS   1016

AQR_HUMAN        657 -M---R------R------KP----K------EN---------N------    665
                      |   |      |      .|    |      :|         |
SEN1_YEAST      1017 KMELARMQLLNNRVVHPPSAPAFHTKSRGLSNKNDDSSSEESDNDIESAR   1066

AQR_HUMAN        666 --F---KAV---LET--I------R---------NL--M----NTDCVVP    684
                       |   ||.   ::|  |      |         .|  |    |.| :.|
SEN1_YEAST      1067 ELFAIAKAKGKGIQTVDINGKVVKRQTAAELAKQELEHMRKRLNVD-MNP   1115

AQR_HUMAN        685 DWLHDIIL-------G-Y-GD-P----S-------S-AHYSK-M-P----    706
                       |::|||       . | .| |    |       | |.|.| | |
SEN1_YEAST      1116 --LYEIILQWDYTRNSEYPDDEPIGNYSDVKDFFNSPADYQKVMKPLLLL   1163

AQR_HUMAN        707 -N-Q-I-ATLDFND--TFLSIEHL---K-A-S-FPGHNV-----K-VTVE    738
                      : | : ::.|..|  .| ||  :   : | | |  ::|      | | ::
SEN1_YEAST      1164 ESWQGLCSSRDREDYKPF-SI--IVGNRTAVSDF--YDVYASVAKQV-IQ   1207

AQR_HUMAN        739 D----PA-L----QIPPFRITFP-VR-SG---KGK-------K-R--KD-    763
                     |    .: |    .:|.||   | .| |.   | |       | |  |:
SEN1_YEAST      1208 DCGISESDLIVMAYLPDFR---PDKRLSSDDFK-KAQHTCLAKVRTLKNT   1253

AQR_HUMAN        764 --ADVEDEDT------EE-AK--TL---I--VEPHV-----IPNR-----    787
                       .:| |. |      .. :|  ||   |  |:  |     | .|
SEN1_YEAST      1254 KGGNV-DV-TLRIHRNHSFSKFLTLRSEIYCVK--VMQMTTI-EREYSTL   1298

AQR_HUMAN        788 -G-PYPYN------Q--PKR--N-------TIQ--F-THT-QIEAI-RAG    813
                      | .| |:      |  |..  |       |::  : .:| |.||| .:
SEN1_YEAST      1299 EGLEY-YDLVGQILQAKPSPPVNVDAAEIETVKKSYKLNTSQAEAIVNS-   1346

AQR_HUMAN        814 M-QPGLTMVVGPPGTGKTDVAVQIISNIY----HNFPEQRTLI-V-THSN    856
                     : :.|.:::.||||||||...:.||.  |    .| .....:| | ...|
SEN1_YEAST      1347 VSKEGFSLIQGPPGTGKTKTILGIIG--YFLSTKN-ASSSNVIKVPLEKN   1393

AQR_HUMAN        857 QALN--QLF--EKIM--A-LD--IDERHL-LRL--G-H---GEEELETEK    890
                     .: |  ||.  :||:  | .:  :||  : |||  | :   | .:.   |
SEN1_YEAST      1394 SS-NTEQLLKKQKILICAPSNAAVDE--ICLRLKSGVYDKQG-HQF---K   1436

AQR_HUMAN        891 -DFSRYGR---VNYVLARRI-EL-LEE-V-KRLQKSLGVPGDASYTCE--    930
                      ...|.||   || | |  | :| ||| | ||       : |:.:|  |
SEN1_YEAST      1437 PQLVRVGRSDVVN-V-A--IKDLTLEELVDKR------I-GERNY--EIR   1473

AQR_HUMAN        931 TAGYFFLYQVMSR-WEEYISKVKN-KGSTLPDVTEVSTFFPFHEYFANAP    978
                     |..      : :.| :...::|.:. :|. | | :| |          .| |
SEN1_YEAST      1474 TDP-----E-LERKFNNAVTKRRELRGK-L-D-SE-S---------GN-P   1503

AQR_HUMAN        979 Q-PIFKGRSYEED---MEIAEGCFRHIKKIFTQL----EEFR-ASEL-LR   1018
                     : |:     | .||   ::: :  .|.:.||..:|    :|.| .:.: .|
SEN1_YEAST      1504 ESPM----S-TEDISKLQL-K--IRELSKIINELGRDRDEMREKNSVNYR   1545

AQR_HUMAN       1019 S-GLDR--SK-YLL-VKEAKIIAMTC-THAAL--KRHD-LVKLGFKYDNI   1059
                     : .|||  :: ::| |.:  ||   | |   |  ..|| |..:|.|:|.:
SEN1_YEAST      1546 NRDLDRRNAQAHILAVSD--II---CST---LSGSAHDVLATMGIKFDTV   1587

AQR_HUMAN       1060 LMEEAAQILEIETFIPLLLQNPQDGFSRLKRWIMIGDHHQLPP-VIKNMA   1108
                     :::||.|..|:.:.||  |:  ..|    ||.||:||.:|||| |:...|
SEN1_YEAST      1588 IIDEACQCTELSSIIP--LR--YGG----KRCIMVGDPNQLPPTVLSGAA   1629

AQR_HUMAN       1109 --FQKYSNMEQSLFTRFVRVGVPTVDLDAQGRARASL-----CNLYNWRY   1151
                       | || |  ||||.|..:...|.: ||.|.|...|:     ...|..|.
SEN1_YEAST      1630 SNF-KY-N--QSLFVRMEKNSSPYL-LDVQYRMHPSISKFPSSEFYQGRL   1674
```

```
AQR_HUMAN       1152 KNLG------N-LP-HVQL--L-P-EFSTANAGLLYDFQLINVEDFQGVG   1189
                     |: |       | .| | ||  | | :|         :|  :|:        |
SEN1_YEAST      1675 KD-GPGMDILNKRPWH-QLEPLAPYKF--------FD--IIS-------G   1705

AQR_HUMAN       1190 ESEPN--PYFYQNLGEAEYV-VAL-FM-YMCLL-GY--PAD---KISILT   1228
                     ..|.|  ...|.|: | | : ||: .: |  |. .:  ..|   ||.|::
SEN1_YEAST      1706 RQEQNAKTMSYTNM-E-E-IRVAIELVDY--LFRKFDNKIDFTGKIGIIS   1750

AQR_HUMAN       1229 TYNG--QKHLIR-DIINRRCGNNPLIGRPNK-V--TTVDRFQGQQNDYIL   1272
                     .|..  ||  :| :.. |..|.  :|    || :  .|:|.||||:.:.||
SEN1_YEAST      1751 PYREQMQK--MRKEFA-RYFGG--MI---NKSIDFNTIDGFQGQEKEIIL   1792

AQR_HUMAN       1273 LSLVR---TR-AVGHLRDVRRLVVAMSRARLGLYIFA--RVSLFQNCFEL   1316
                     :|.||   |: :||.|:|.||:.||::||:.:::..  | ||.::  :|
SEN1_YEAST      1793 ISCVRADDTKSSVGFLKDFRRMNVALTRAKTSIWVLGHQR-SLAKS--KL   1839

AQR_HUMAN       1317 TPAFSQL--TA--RP-LHLHIIPTEPF--PTTRKNGERPSHEVQ-II-K-   1356
                        :..|  .|  |. | .:  ....|  |  |.|  |     .| |: |
SEN1_YEAST      1840 ---WRDLIEDAKDRSCL-AY--ACSGFLDP--RNN--R----AQSILRKF   1875

AQR_HUMAN       1357 NMP----QMANFVYNMYM-HLIQTTHHY--HQ-TLLQLPPAMVEEGEE--   1396
                     |:|     |..:  |.:.| ::.|.....  :: |  : ...:|:||||
SEN1_YEAST      1876 NVPVPSEQEDD--YKLPMEYITQGPDEVKSNKDT--K-KRRVVDEGEEAD   1920

AQR_HUMAN       1397 --VQNQETELETEEEAMTVQADIIPSPTD---TSCRQ-ETPAFQTDT-T-   1438
                       |:.::.| :.:|:..: :||       |    .: :: |:|:  |.: |
SEN1_YEAST      1921 KAVKKKKKE-KKKEKKKS-KAD------DKKKNN-KKAESPS--TSSGTK   1959

AQR_HUMAN       1439 -PSET-GATSTPEA-IPALSETTPTV-----VGAVSAPAEANTP----QD   1476
                      .|.. |..|.|.| :|   :|.|.|      ..|| ...:.|..     .|
SEN1_YEAST      1960 KKSSIFGGMSVPSAVVP---KTFPDVDSNKKAAAV-VGKKKNNKHVCFSD   2005

AQR_HUMAN       1477 ATS-AP---E-ETK   1485
                     ..| .|   | |.|
SEN1_YEAST      2006 DVSFIPRNDEPEIK   2019

--------------------------------------------
Name 1: M3K6_HUMAN
Name 2: GCN2_YEAST

Mitogen-activated protein kinase kinase kinase 6 OS=Homo sapiens GN=MAP3K6 PE=1
SV=3
Serine/threonine-protein kinase GCN2 OS=Saccharomyces cerevisiae (strain ATCC 20
4508 / S288c) GN=GCN2 PE=1 SV=3

Needed for following interaction(s):
yeast   genetic M3K6_HUMAN      RASK_HUMAN      MAP3K6  KRAS    GCN2_YEAST
IRA2_YEAST

Length sequence 1:      1288
Length sequence 2:      1659
Sequence #1: M3K6_HUMAN
Sequence #2: GCN2_YEAST
Length #1: 1728
Length #2: 1728
Matrix: BLOSUM62
Gap open: 2.0
Gap extend: 1.0
Length: 1728
Identity: 444/1728 (25.69%)
Similarity: 671/1728 (38.83%)
Gaps: 734/1728 (42.48%)
Score: 1307.00

M3K6_HUMAN        21 LA-VALSRGRQLAAPPGRGC----A-RSRPLSVVYV-----LT-R----E     54
                     |: :.|.:..::      : |    | ||      :|:      || |     :
GCN2_YEAST         3 LSHLTLDQYYEI-----Q-CNELEAIRS-----IYMDDFTDLTKRKSSWD     41

M3K6_HUMAN        55 PQPGL--E-P-REGTEAEPL--PLRCLREACAQVPR-PRPPPQLRSLPFG     97
                     .||.:  | . | ..:.||:  .: .|.  .|..|. |...|::.   |.
GCN2_YEAST        42 KQPQIIFEITLR-SVDKEPVESSI-TLH--FAMTPMYPYTAPEIE---FK     84

M3K6_HUMAN        98 TLE--LGDT--AALDA-F---YN-A---DVVVLEVSSSLVQPSLFYHLGV    135
                     .::  : |:  ..|.: |   :| :   : ::.|: :|.|..|..|
GCN2_YEAST        85 NVQNVM-DSQLQMLKSEFKKIHNTSRGQE-IIFEI-TSFTQEKL------    125

M3K6_HUMAN       136 RESFSMTNNVLLCSQA--DLPD-LQ-------AL----RE---DVFQKNS    168
                      :.|   .|| :.:|:  |  | ||        .|     ||    :..:|.|
GCN2_YEAST       126 -DEF---QNV-VNTQSLED--DRLQRIKETKEQLEKEEREKQQETIKKRS    168

M3K6_HUMAN       169 DCVGSYTLIPYVVTATGRVL----CGDAGLL--R--GLADGLVQ------    204
                     |    ....|..:|.   |.|    ..|..||  |   .| | | |
GCN2_YEAST       169 D---EQRRIDEIVQ---RELEKRQDDDDDLLFNRTTQL-D-L-QPPSEWV    209

M3K6_HUMAN       205 AGVGTEALL---TPLVGRLARLLEATPTDSCGY-FRETIR-RDIRQARER    249
                     |. | |.|::    |   : : |:|     ..|.|| : |:..:. :.|:..:.
GCN2_YEAST       210 AS-G-EAIVFSKT--I-K-AKL----PNNSM-FKFKAVVNPKPIKLTSDI    248

M3K6_HUMAN       250 FS-GPQ-L-R----QE--LARLQRRLDSVELLSPDIIM-NL--LLSYRDV    287
                     || ..| | :     .|  ||       | . |:|.: :| |.  |||  ::
GCN2_YEAST       249 FSFSKQFLVKPYIPPESPLA------D-F-LMSSE-MMENFYYLLS--EI    287

M3K6_HUMAN       288 Q-D--Y---S----AI--I--ELVETLQALPTCDVAEQH-NV--CFHYTF    320
                     : |  |    |     .|  :  || ||:  |.     | :| ||  .|.||
GCN2_YEAST       288 ELDNSYFNTSNGKKEIANLEKEL-ETV--LK----A-KHDNVNRLFGYT-    328

M3K6_HUMAN       321 ALNR--RNRPGDRAKAL-SVLLPLVQLEGSVAPDLYCMCGRIYK--DMFF    365
                      :.|  ||     .|..: .:.| |.  |         ||   ..|.  |: .
GCN2_YEAST       329 -VERMGRN----NATFVWKIRL-LT--E-------YC---NYYPLGDL-I    359

M3K6_HUMAN       366 SS-GFQD-AGHREQAYHW-YRKAFD-VEPSLHS-G-----IN-AAVLLIA    404
                     .| ||.: |..|    . | .| ..: :| ::|. |      || ..|:|:.
GCN2_YEAST       360 QSVGFVNLATAR---I-WMIR-LLEGLE-AIHKLGIVHKCINLETVILVK    403

M3K6_HUMAN       405 -AGQHFEDSKEL-RLI----G--MKLGCLLAR---K-G-CVEKMQ-YYWD    440
                      |  .| .| .: :|:     |   : |. :|:|    | | .|| :. ..|
GCN2_YEAST       404 DA--DF-GS-TIPKLVHSTYGYTV-LN-MLSRYPNKNGSSVE-LSPSTW-    445

M3K6_HUMAN       441 VGFYLGA-QILA-ND--P---T---QV-VLAAEQLYK-----LN--APIW    472
                     :      | ::|. |:  |    |    |: ||.. |:..      :|  .|..
GCN2_YEAST       446 I-----APELLKFNNAKPQRLTDIWQLGVLFI-QIISGSDIVMNFETPQE    489

M3K6_HUMAN       473 YL--VSVM-ET-F-LLYQHFRPTPEPPGGPPRRAHFWLHFLLQSCQP--F    515
                     :|  .| | || : ||.:... . :     |.:|.. .|. ||     |  |
GCN2_YEAST       490 FLDSTS-MDETLYDLLSKMLN-N-D----PKKRLG-TLE-LL----PMKF    526

M3K6_HUMAN       516 -KTACAQGDQCL--V-LVLE-MN-KVL-L-PA-KLEVRG----T---DPV    549
                      :|..    |..:  . ||.| :| ..| | |. .:.|||     |    ..:
GCN2_YEAST       527 LRTNI---DSTINRFNLVSESVNSNSLELTPGDTITVRGNGGRTLSQSSI    573

M3K6_HUMAN       550 ---S-TV-T-LSLLEPETQDIPSSW-T-F-PVASICGVSA--S--K-R--    583
                        | .| : .|.:.|.|:   |.: : | .:| :.|..|   .  | |
GCN2_YEAST       574 RRRSFNVGSRFSSINPATR---SRYASDFEEIA-VLGQGAFGQVVKARNA    619

M3K6_HUMAN       584 -DERCCFLYALPP---AQ--------DVQLCFPSVGHCQWFCGLIQ---A    618
                      |.|  : ||:..    .:          :|.| ..|:.| | :  :::   |
GCN2_YEAST       620 LDSR--Y-YAIKKIRHTEEKLSTILSEVML-LASLNH-Q-Y--VVRYYAA    661

M3K6_HUMAN       619 WV---T---NP-DSTAPAEE---AEGAG---E--MLE----F----DYEY    645
                     |:    :    |. :||  .||    :|.:.    |  :|:     |     :::.
GCN2_YEAST       662 WLEEDSMDENVFEST--DEESDLSESSSDFEENDLLDQSSIFKNRTNHDL    709

M3K6_HUMAN       646 TETG-ERLVLGKGTY-GVVY--AGRD---R---H-TRVRIAIKEIPERDS    684
                     ..:. : .:.|.| | .:|:  :.||    .    | |. ..:..| .:.|:
GCN2_YEAST       710 DNSNWD-FISGSG-YPDIVFENSSRDDENEDLDHDTS-STSSE-SQDDT    755

M3K6_HUMAN       685 -RFSQPLHEEIALHRRLRHKNIVRYLGSA-SQGGYLK--IF--ME--EVP    726
                      :.|:.: :.:.  || |  |.|:.: :| .:     |  :|  ||  |
GCN2_YEAST       756 DKESKSI-QNVP--RR-R--NFVKPM-TAVKK----KSTLFIQMEYCE--    792

M3K6_HUMAN       727 GGSLSSLLRSVWGPL---KDNES-TISFYTRQILQGLGYLHDNHIVHRDI    772
                     ..:|..|:.|. . |    :| |. .: |  ||||:.|.|:|...|:|||:
GCN2_YEAST       793 NRTLYDLIHSE-N-LNQQRD-EYWRL-F--RQILEALSYIHSQGIIHRDL    836

M3K6_HUMAN       773 KGDNVLINTFS-GLLKISDFGTSK---R-LAGI----T---P-CTETFT-    808
                     |..|:.|:. | . :||.|||.:|   | | .|     :   | .::..|
GCN2_YEAST       837 KPMNIFIDE-SRN-VKIGDFGLAKNVHRSL-DILKLDSQNLPGSSDNLTS    883
```

```
M3K6_HUMAN       809 --GTLQYMAPEIIDQGPRG-YG-KAADIWSLGCTVI--EMATGRPPFHEL    852
                       ||..|:|.|::| | .| |. | .|::|||  :|  ||..   || ..
GCN2_YEAST       884 AIGTAMYVATEVLD-G-TGHYNEK-IDMYSLG--IIFFEMIY---PF-ST    924

M3K6_HUMAN       853 GSPQAAMFQVGMYK----V---HPP-M-PSSLSAEAQAFLLR-TFEPDPR    892
                     |     |.:|.:.|    |   .|| . .:.:..|.:  ::| ..:.||.
GCN2_YEAST       925 G-----MERVNILKKLRSVSIEFPPDFDDNKMKVEKK--IIRLLIDHDPN    967

M3K6_HUMAN       893 LRASAQTLL--G--DPFL--QPG--K---RSRS-PSSP-----RHA--PR    923
                     .|..|:|||  |   | :  |..  |   :|.| ||||     |.:  .:
GCN2_YEAST       968 KRPGARTLLNSGWL-P-VKHQDEVIKEALKSLSNPSSPWQQQVRESLFNQ   1015

M3K6_HUMAN       924 P-S-------D--APSASPTPSANSTTQSQ-T------FPCPQAPSQH--    954
                     . |       |  .|: | ||.|| ..:|| |      |   :   :|
GCN2_YEAST      1016 SYSLTNDILFDNSVPT-S-TPFAN-ILRSQMTEEVVKIF---R---KHGG   1056

M3K6_HUMAN       955 -----PPS--P--P----KRCLSY-----GGT-SQLR--V--P------E    975
                          ||.  |  |    :. : |     ||| .||:  :  |      :
GCN2_YEAST      1057 IENNAPPRIFPKAPIYGTQN-V-YEVLDKGGTVLQLQYDLTYPMARYLSK   1104

M3K6_HUMAN       976 EPA--AEE--------PASPEES-SGLSLLHQESKRR-AML-AAVLEQ--   1010
                     .|:  :::        |  |:.| |  ||   | .|: ..: ..::.:
GCN2_YEAST      1105 NPSLISKQYRMQHVYRP--PDHSRS--SL---E-PRKFGEIDFDIISKSS   1146

M3K6_HUMAN      1011 -ELPAL--AENLH--QE--------QKQEQGARLGRNHVEELLRCLGAHI   1047
                      | ...  ||:|.  .|        :|......|  ||. ::|..: .:.
GCN2_YEAST      1147 SE-SGFYDAESLKIIDEILTVFPVFEKTNTFFIL--NHA-DILESV-FNF   1191

M3K6_HUMAN      1048 HTPN-RRQLAQE-L--RAL-Q-G--R--------LRAQ-GL-GPAL--LH   1077
                      | | .:  ||. |  |.| | |  |        |:|| .: ..||  |.
GCN2_YEAST      1192 -T-NIDK--AQRPLVSRMLSQVGFARSFKEVKNELKAQLNISSTALNDLE   1237

M3K6_HUMAN      1078 RPLFAFP-D--AVKQILRKRQI-RPHWM-FVLDSL--LSRAVRAALGVLG   1120
                       ||.|. |  |.|:.|.|..| .|| : .:.|||  :|: |   |..|.
GCN2_YEAST      1238 --LFDFRLDFEAAKKRLYKLMIDSPH-LKKIEDSLSHISK-V---LSYLK   1280

M3K6_HUMAN      1121 P-EVEKEAV-SPRSEELSN------EG---------D--SQQ---SPGQQ   1148
                     | ||.:..| ||    |||      :|         |  |:.   :.|:.
GCN2_YEAST      1281 PLEVARNVVISP----LSNYNSAFYKGGIMFHAVYDDGSSRNMIAAGGRY   1326

M3K6_HUMAN      1149 SPLPV----EPEQG-------PAPLMVQLSLLRA-ETDRLREILAGKERE   1186
                     ..| :    .| .|       .|   |..:|  | ||     |. |..:.
GCN2_YEAST      1327 DTL-ISFFARP-SGKKSSNTRKA---VGFNL--AWET-----IF-GIAQN   1363

M3K6_HUMAN      1187 Y-QALV--QRAL-QRLNEEARTYVLAPEPPTAL----S-TDQGLV-QWLQ   1226
                     | : |.  .| : :| |   | : | .:  ||:    | .|. |: .:..
GCN2_YEAST      1364 YFK-LASGNR-IKKR-N---R-F-L-KD--TAVDWKPSRCDV-LISSFSN   1401

M3K6_HUMAN      1227 ELNVDS-G-TIQMLLNHSFTL--HTLLTYA--TR-----DDLIYTRIRG-   1264
                     .| :|: | ||   ||   ||  ..:  .|  .|     ||:: |   |
GCN2_YEAST      1402 SL-LDTIGVTI---LN---TLWKQNI--KADMLRDCSSVDDVV-T---GA   1438

M3K6_HUMAN      1265 ---GMVCRI-WRAIL--AQRAGSTPVTS   1286
                        |    | |  ||  .|:|  .|:|:
GCN2_YEAST      1439 QQDG----IDW--ILLIKQQA--YPLTN   1458

--------------------------------------------
Name 1: SGPP1_HUMAN
Name 2: DS1P1_YEAST

Sphingosine-1-phosphate phosphatase 1 OS=Homo sapiens GN=SGPP1 PE=1 SV=2
Dihydrosphingosine 1-phosphate phosphatase LCB3 OS=Saccharomyces cerevisiae (str
ain ATCC 204508 / S288c) GN=LCB3 PE=1 SV=1

Needed for following interaction(s):
yeast   genetic RASK_HUMAN      SGPP1_HUMAN     KRAS    SGPP1   IRA2_YEAST
DS1P1_YEAST

Length sequence 1:      441
Length sequence 2:      409
Sequence #1: SGPP1_HUMAN
Sequence #2: DS1P1_YEAST
Length #1: 517
Length #2: 517
Matrix: BLOSUM62
Gap open: 2.0
Gap extend: 1.0
Length: 517
Identity: 149/517 (28.82%)
Similarity: 220/517 (42.55%)
Gaps: 193/517 (37.33%)
Score: 558.00

SGPP1_HUMAN       10 LV-GRLQDPQKVARFQRLCGVEAPPRRSADRRED-EKAEAP--LAGDPRL     55
                     :| | | :...: | :|     |   |:.....| :  | |  |. ||
DS1P1_YEAST        1 MVDG-L-NTSNI-R-KR-----A---RTLSNPNDFQ--E-PNYLL-DP--     32

SGPP1_HUMAN       56 RGRQPGAPGGPQPPGSD--RNQCPAK---PDGGGAPNGVRNGLAAELGPA    100
                      |..|          ||  |.:. :|          | :|..|   |
DS1P1_YEAST       33 -GNHP----------SDHFRTRM-SKFRF-------N-IREKL---L---     56

SGPP1_HUMAN      101 SPRRAGALRRN-SLT-GE-EGQLARVS--NWPLYCLFCFGTEL-GNELFY    144
                           ....| |.| .. :.:. | |  | .||  |.: |.| |:..||
DS1P1_YEAST       57 ------VFTNNQSFTLSRWQKKY-R-SAFN-DLY--FTY-TSLMGSHTFY     94

SGPP1_HUMAN      145 IL-F-FPFWI-WNLDPLVGRRLVVIWVL---VMYL-GQCTKDIIRW--PR    185
                     :| . .|.|. : .:  ..:.:|  ::|   : || | ..||.  |  ||
DS1P1_YEAST       95 VLCLPMPVWFGY-FE--TTKDMV--YILGYSI-YLSG-FFKDY--WCLPR    135

SGPP1_HUMAN      186 PASPPV--VKL-EVFYNS-EYSMPSTH-AMSGTAIPISMVLLTYGRW--Q    228
                     |.:||:  :.| |  |.: ||..||:| | :.|.  :|::.| |..|  |
DS1P1_YEAST      136 PRAPPLHRITLSE--YTTKEYGAPSSHTA-NATG--VSLLFL-YNIWRMQ    179

SGPP1_HUMAN      229 YP--LIYGLILIPC-----WCSLVCLSRIYMGMHSILDIIAGFLYTIL--    269
                     ..  ::. | |:.|     :.:|| ..|||.|||.|||:::|.|..|:
DS1P1_YEAST      180 ESSVMVQ-L-LLSCVVLFYYMTLV-FGRIYCGMHGILDLVSGGLIGIVCF    226

SGPP1_HUMAN      270 ILAV-F-YPFVDL-IDNFNQTHKYAP-FII-IGLHLALGIFSFT--LDTW    312
                     |:.: | |.|..| |:   : |.:.| |.: .|| | | :|...  :|..
DS1P1_YEAST      227 IVRMYFKYRFPGLRIE---E-HWWFPLFSVGWGL-L-L-LFKHVKPVDEC    269

SGPP1_HUMAN      313 STSRGDT-AEILG--SGAGI-AC---GS--HVT--YNMGLVLDPSLDTLP    351
                     ...: |: | .:|  |  || .|   |.  .||  ||    |:|:
DS1P1_YEAST      270 PCFQ-DSVA-FMGVVS--GIECCDWLGKVFGVTLVYN----LEPN-----    306

SGPP1_HUMAN      352 LAGPPITVTLFGKAILRILIGMVFVLII-RDVMKK--I-TIPLACKIFNI    397
                      .|  ..:||   |  |:|:|:..| :| :.|:.|  | |: | .|:|::
DS1P1_YEAST      307 -CG--WRLTL---A--RLLVGLPCV-VIWKYVISKPMIYTL-L-IKVFHL    345

SGPP1_HUMAN      398 PCDD--I--RKAR-Q--HME----VELP-Y----------RYITY-GMVG    424
                     . ||  :  || | :  |.|    .|.| |          |:|.| | |.
DS1P1_YEAST      346 K-DDRNVAARK-RLEATHKEGASKYECPLYIGEPKIDILGRFIIYAG-VP    392

SGPP1_HUMAN      425 FSITFFVPYIFFFIGIS    441
                     |::....|.:|..:.|:
DS1P1_YEAST      393 FTVVMCSPVLFSLLNIA    409

--------------------------------------------
Name 1: IF4A1_HUMAN
Name 2: IF4A_DROME

Eukaryotic initiation factor 4A-I OS=Homo sapiens GN=EIF4A1 PE=1 SV=1
Eukaryotic initiation factor 4A OS=Drosophila melanogaster GN=eIF-4a PE=2 SV=3

Needed for following interaction(s):
fly     genetic IF4A1_HUMAN     PTEN_HUMAN      EIF4A1  PTEN    IF4A_DROME
Q7KMQ6_DROME

Length sequence 1:      406
Length sequence 2:      403
Sequence #1: IF4A1_HUMAN
Sequence #2: IF4A_DROME
Length #1: 409
Length #2: 409
Matrix: BLOSUM62
Gap open: 2.0
Gap extend: 1.0
```

```
Length: 409
Identity: 299/409 (73.11%)
Similarity: 357/409 (87.29%)
Gaps: 15/409 (3.67%)
Score: 1580.00

IF4A1_HUMAN        6 DSRS---RDNGPDGMEPEGVIESNWNEIVDSFDDMNLSESLLRGIYAYGF     52
                     |.|:   :| ||..|||||||||.|:|:.|:||||||.|.||||||.|||
IF4A_DROME         2 DDRNEIPQD-GPASMEPEGVIESTWHEVYDNFDDMNLREELLRGIYGYGF     50

IF4A1_HUMAN       53 EKPSAIQQRAILPCIKGYDVIAQAQSGTGKTATFAISILQQIELDLKATQ    102
                     |||||||||||:||::|.||||||||||||||||:|:|||||:..::..|
IF4A_DROME        51 EKPSAIQQRAIIPCVRGRDVIAQAQSGTGKTATFSIAILQQIDTSIRECQ    100

IF4A1_HUMAN      103 ALVLAPTRELAQQIQKVVMALGDYMGA-SCHACIGGTNVRAEVQKLQMEA    151
                     ||:||||||||.|||:||||||:||.. | |||||||||| |..:: :|:
IF4A_DROME       101 ALILAPTRELATQIQRVVMALGEYMKVHS-HACIGGTNVR-EDARI-LES    147

IF4A1_HUMAN      152 P-HIIVGTPGRVFDMLNRRYLSPKYIKMFVLDEADEMLSRGFKDQIYDIF    200
                     . |::|||||||:||:||:.|..:|||:||||||||||||||||||.|:|
IF4A_DROME       148 GCHVVVGTPGRVYDMINRKVLRTQYIKLFVLDEADEMLSRGFKDQIQDVF    197

IF4A1_HUMAN      201 QK-LNSNTQVVLLSATMPSDVLEVTKKFMRDPIRILVKKEELTLEGIRQF    249
                      | |..:.||:|||||||.|||||::.|||||:.||||||||||||||:||
IF4A_DROME       198 -KMLPPDVQVILLSATMPPDVLEVSRCFMRDPVSILVKKEELTLEGIKQF    246

IF4A1_HUMAN      250 YINVEREEWKLDTLCDLYETLTITQAVIFINTRRKVDWLTEKM--HARDF    297
                     |:||::|.|||.||||||:||:|||:|||.|||||||.||::|  |  :|
IF4A_DROME       247 YVNVKQENWKLGTLCDLYDTLSITQSVIFCNTRRKVDQLTQEMSIH--NF    294

IF4A1_HUMAN      298 TVSAMHGDMDQKERDVIMREFRSGSSRVLITTDLLARGIDVQQVSLVINY    347
                     |||||||||:|::|:|||::||||||||||||||||||||||||||||||
IF4A_DROME       295 TVSAMHGDMEQRDREVIMKQFRSGSSRVLITTDLLARGIDVQQVSLVINY    344

IF4A1_HUMAN      348 DLPTNRENYIHRIGRGGRFGRKGVAINMVTEEDKRTLRDIETFYNTSIEE    397
                     |||:|||||||||||||||||||||||.:|::|:|.|:|||.||:|:|||
IF4A_DROME       345 DLPSNRENYIHRIGRGGRFGRKGVAINFITDDDRRILKDIEQFYHTTIEE    394

IF4A1_HUMAN      398 MPLNVADLI    406
                     ||.|:||||
IF4A_DROME       395 MPANIADLI    403

--------------------------------------------
Name 1: TERA_HUMAN
Name 2: CDC48_YEAST

Transitional endoplasmic reticulum ATPase OS=Homo sapiens GN=VCP PE=1 SV=4
Cell division control protein 48 OS=Saccharomyces cerevisiae (strain ATCC 204508
 / S288c) GN=CDC48 PE=1 SV=3

Needed for following interaction(s):
yeast   genetic TERA_HUMAN      BLM_HUMAN       VCP     BLM     CDC48_YEAST
SGS1_YEAST

Length sequence 1:      806
Length sequence 2:      835
Sequence #1: TERA_HUMAN
Sequence #2: CDC48_YEAST
Length #1: 834
Length #2: 834
Matrix: BLOSUM62
Gap open: 2.0
Gap extend: 1.0
Length: 834
Identity: 577/834 (69.18%)
Similarity: 699/834 (83.81%)
Gaps: 40/834 (4.80%)
Score: 3058.00

TERA_HUMAN         2 ASGADSKGDD-LSTAILKQKNRPNRLIVDEAINEDNSVVSL-SQPKMDEL     49
                     |||.|.:.:| .:||||::|.:.|.|:||:|||:||||::: |. .||:|
CDC48_YEAST       11 ASGVDPREEDKTATAILRKKKDNMLLVDDAINDDNSVIAINSN-TMDKL     59

TERA_HUMAN        50 QLFRGDTVLLKGKKRREAVCIVLSDDTCSDEKIRMNRVVRNNLRVRLGDV     99
                     :||||||||:|||||::.|.|||.||...|...|:|||||||||:||||:
CDC48_YEAST       60 ELFRGDTVLVKGKKRKDTVLIVLIDDELEDGACRINRVVRNNLRIRLGDL    109

TERA_HUMAN       100 ISIQPCPDVKYGKRIHVLPIDDTVEGITGNLFEVYLKPYFLEAYRPIRKG    149
                     ::|.||||:||..||.||||.||:||||||||:|:|||||:|||||:|||
CDC48_YEAST      110 VTIHPCPDIKYATRISVLPIADTIEGITGNLFDVFLKPYFVEAYRPVRKG    159

TERA_HUMAN       150 DIFLVRGGMRAVEFKVVETDPSPYCIVAPDTVIHCEGEPIKREDEEESLN    199
                     |.|:||||||.||||||:.:|..|.:||.||:||.|||||.|||||.::|
CDC48_YEAST      160 DHFVVRGGMRQVEFKVVDVEPEEYAVVAQDTIIHWEGEPINREDEENNMN    209

TERA_HUMAN       200 EVGYDDIGGCRKQLAQIKEMVELPLRHPALFKAIGVKPPRGILLYGPPGT    249
                     |||||||||||||:|||:||||||||||.||||||:|||||:|:||||||
CDC48_YEAST      210 EVGYDDIGGCRKQMAQIREMVELPLRHPQLFKAIGIKPPRGVLMYGPPGT    259

TERA_HUMAN       250 GKTLIARAVANETGAFFFLINGPEIMSKLAGESESNLRKAFEEAEKNAPA    299
                     ||||:|||||||||||||||||||:|||:|||||||||||||||||||||
CDC48_YEAST      260 GKTLMARAVANETGAFFFLINGPEVMSKMAGESESNLRKAFEEAEKNAPA    309

TERA_HUMAN       300 IIFIDELDAIAPKREKTHGEVERRIVSQLLTLMDGLKQRAHVIVMAATNR    349
                     ||||||:|:|||||:||:||||||:||||||||||:|.|::|:|:|||||
CDC48_YEAST      310 IIFIDEIDSIAPKRDKTNGEVERRVVSQLLTLMDGMKARSNVVVIAATNR    359

TERA_HUMAN       350 PNSIDPALRRFGRFDREVDIGIPDATGRLEILQIHTKNMKLADDVDLEQV    399
                     ||||||||||||||||||||||||||||||:|:|||||||||||||||.:
CDC48_YEAST      360 PNSIDPALRRFGRFDREVDIGIPDATGRLEVLRIHTKNMKLADDVDLEAL    409

TERA_HUMAN       400 ANETHGHVGADLAALCSEAALQAIRKKMDLIDL-EDETIDAEVMNSLAVT    448
                     |.||||:||||:|:||||||:|.||:||||||| ||| |||||::||.||
CDC48_YEAST      410 AAETHGYVGADIASLCSEAAMQQIREKMDLIDLDEDE-IDAEVLDSLGVT    458

TERA_HUMAN       449 MDDFRWALSQSNPSALRETVVE-VPQVTWEDIGGLEDVKRELQELVQYPV    497
                     ||:||:||..|||||||||||| | .|||:|:|||:::|.||:|.|:|||
CDC48_YEAST      459 MDNFRFALGNSNPSALRETVVESV-NVTWDDVGGLDEIKEELKETVEYPV    507

TERA_HUMAN       498 EHPDKFLKFGMTPSKGVLFYGPPGCGKTLLAKAIANECQANFISIKGPEL    547
                     .|||::.|||::||||||||||||.||||||||:|.|..||||:|||||
CDC48_YEAST      508 LHPDQYTKFGLSPSKGVLFYGPPGTGKTLLAKAVATEVSANFISVKGPEL    557

TERA_HUMAN       548 LTMWFGESEANVREIFDKARQAAPCVLFFDELDSIAKARGGNIGDGGGAA    597
                     |:||:||||:|:|:||||||.|||.|:|.||||||||||||::||.|||:
CDC48_YEAST      558 LSMWYGESESNIRDIFDKARAAAPTVVFLDELDSIAKARGGSLGDAGGAS    607

TERA_HUMAN       598 DRVINQILTEMDGMSTKKNVFIIGATNRPDIIDPAILRPGRLDQLIYIPL    647
                     |||:||:|||||||:.|||||:||||||||.||||||||||||||||:||
CDC48_YEAST      608 DRVVNQLLTEMDGMNAKKNVFVIGATNRPDQIDPAILRPGRLDQLIYVPL    657

TERA_HUMAN       648 PDEKSRVAILKANLRKSPVAKDVDLEF--LAKMTNGFSGADLTEICQRAC    695
                     |||.:|::||.|.|||:|:  :..||.  :||.|.|||||||..|.|||.
CDC48_YEAST      658 PDENARLSILNAQLRKTPL--EPGLELTAIAKATQGFSGADLLYIVQRAA    705

TERA_HUMAN       696 KLAIRESIESEIRR---ERE-R------Q-TNPSA-MEVE-EDDPVPEIR    732
                     |.||::|||:. |:   |:| :      : |:..| .|.| |.||||.|.
CDC48_YEAST      706 KYAIKDSIEAH-RQHEAEKEVKVEGEDVEMTDEGAKAEQEPEVDPVPYIT    754

TERA_HUMAN       733 RDHFEEAMRFARRSVSDNDIRKYEMFAQTLQQSRG-FGSFRF---PSG--    776
                     ::||.|||:.|:|||||.::|:||.::|.::.||| |.:|.|   |.|
CDC48_YEAST      755 KEHFAEAMKTAKRSVSDAELRRYEAYSQQMKASRGQFSNFNFNDAPLGTT    804

TERA_HUMAN       777 -----NQGGAGPSQGSGGGTGGSVYTEDNDDDLY    805
                          |...:.|| |:|... || ..|: |||||
CDC48_YEAST      805 ATDNANSNNSAPS-GAGAAF-GS-NAEE-DDDLY    834

--------------------------------------------
Name 1: CDK2_HUMAN
Name 2: CDK1_YEAST

Cyclin-dependent kinase 2 OS=Homo sapiens GN=CDK2 PE=1 SV=2
Cyclin-dependent kinase 1 OS=Saccharomyces cerevisiae (strain ATCC 204508 / S288
c) GN=CDC28 PE=1 SV=1

Needed for following interaction(s):
yeast   physical        CDK2_HUMAN      PTTG1_HUMAN     CDK2    PTTG1   CDK1_YEA
ST      SECU_YEAST
```

```
yeast   genetic CDK2_HUMAN      PTTG1_HUMAN     CDK2    PTTG1   CDK1_YEAST
SECU_YEAST

Length sequence 1:      298
Length sequence 2:      298
Sequence #1: CDK2_HUMAN
Sequence #2: CDK1_YEAST
Length #1: 306
Length #2: 306
Matrix: BLOSUM62
Gap open: 2.0
Gap extend: 1.0
Length: 306
Identity: 192/306 (62.75%)
Similarity: 241/306 (78.76%)
Gaps: 27/306 (8.82%)
Score: 1050.00

CDK2_HUMAN          1 M--E--NFQKVEKIGEGTYGVVYKA---R-NKLTGE-VVALKKIRLDTET     41
                      |  |  |::::||:|||||||||||   | .:  |: |||||||||::|.
CDK1_YEAST          1 MSGELANYKRLEKVGEGTYGVVYKALDLRPGQ--GQRVVALKKIRLESED     48

CDK2_HUMAN         42 EGVPSTAIREISLLKELNHPNIVKLLDVIHTE-NKLYLVFEFLHQDLKKF     90
                      |||||||||||||||||...|||:|.|::|:: :|||||||||..|||::
CDK1_YEAST         49 EGVPSTAIREISLLKELKDDNIVRLYDIVHSDAHKLYLVFEFLDLDLKRY     98

CDK2_HUMAN         91 MDASALTGIP--LPL----IKSYLFQLLQGLAFCHSHRVLHRDLKPQNLL    134
                      |:     |||  .||    :|.::.||.:|:|:|||||:|||||||||||
CDK1_YEAST         99 ME-----GIPKDQPLGADIVKKFMMQLCKGIAYCHSHRILHRDLKPQNLL    143

CDK2_HUMAN        135 INTEGAIKLADFGLARAFGVPVRTYTHEVVTLWYRAPEILLGCKYYSTAV    184
                      ||.:|.:||.|||||||||||:|.||||:|||||||||:|||.|.|||.|
CDK1_YEAST        144 INKDGNLKLGDFGLARAFGVPLRAYTHEIVTLWYRAPEVLLGGKQYSTGV    193

CDK2_HUMAN        185 DIWSLGCIFAEMVTRRALFPGDSEIDQLFRIFRTLGTPDEVVWPGVTSMP    234
                      |.||:|||||||..|:.:|.|||||||:|:|||.||||:|.:||.:..:|
CDK1_YEAST        194 DTWSIGCIFAEMCNRKPIFSGDSEIDQIFKIFRVLGTPNEAIWPDIVYLP    243

CDK2_HUMAN        235 DYKPSFPKWARQDFSKVVPPLDEDGRSLLSQMLHYDP-NKRISA-KAALA    282
                      |:|||||:|.|:|.|:|||.||..|..||.::|.||| | |||| :||:
CDK1_YEAST        244 DFKPSFPQWRRKDLSQVVPSLDPRGIDLLDKLLAYDPIN-RISARRAAI-    291

CDK2_HUMAN        283 HPFFQD    288
                      ||:||:
CDK1_YEAST        292 HPYFQE    297

--------------------------------------------
Name 1: FOXF1_HUMAN
Name 2: HCM1_YEAST

Forkhead box protein F1 OS=Homo sapiens GN=FOXF1 PE=2 SV=2
Forkhead transcription factor HCM1 OS=Saccharomyces cerevisiae (strain ATCC 2045
08 / S288c) GN=HCM1 PE=1 SV=3

Needed for following interaction(s):
yeast   genetic PTTG1_HUMAN     FOXF1_HUMAN     PTTG1   FOXF1   SECU_YEAST
HCM1_YEAST

Length sequence 1:      379
Length sequence 2:      564
Sequence #1: FOXF1_HUMAN
Sequence #2: HCM1_YEAST
Length #1: 576
Length #2: 576
Matrix: BLOSUM62
Gap open: 2.0
Gap extend: 1.0
Length: 576
Identity: 168/576 (29.17%)
Similarity: 217/576 (37.67%)
Gaps: 269/576 (46.70%)
Score: 560.00

FOXF1_HUMAN         1 MSSAPEKQQPPHGGGGGGGGGGAAMDP-ASS--GPSKAKK----T----     39
```

```
                      |... .| :|.|            .:.| .||  .|||||:     |
HCM1_YEAST         49 MKEV-NK-RPSH------------PLSPDHSSPIAPSKAKRQRSDTCARS     84

FOXF1_HUMAN        40 NAG------I------RR--PE---KPPYSYIA-LIVMAI-QSSPTKRLT     70
                      | |      |      ||  .|   |||||| | ||.:|| ||...| ||
HCM1_YEAST         85 N-GNLTLEEILQSLERRRINGELAKKPPYSY-ATLICLAILQSQEGK-LT    131

FOXF1_HUMAN        71 LSEIYQFLQSRFPFFRGSYQ---GWKNSVRHNLSLNECFIKLPKGLGRPG    117
                      ||:||.::...||:::   |   .|:||:|||||||:.|||..|..  .|
HCM1_YEAST        132 LSQIYHWIHVHFPYYK---QKDASWQNSIRHNLSLNDAFIKTEKSC--DG    176

FOXF1_HUMAN       118 KGHYWTIDPASE--FMFEEGSFRRRPRG--F-RRKCQAL-KPMY----S-    156
                      |||:|.:.|.:|  | | :|   .. ||  | :...|.: |  |    |
HCM1_YEAST        177 KGHFWEVRPGAETKF-F-KG---EN-RGYEFVKDSLQDIGK--YFEIDST    218

FOXF1_HUMAN       157 M--M----NGLGFN-HLPD-----TYG-F-----Q-GSAGGL--S----C    181
                      :  :    :|.| | .|||     ..| |     | .|:..|  |    .
HCM1_YEAST        219 LDELEQVESGEG-NDDLPDEEEREEAGKFPSIEIQLNSSPILRVSQLHHI    267

FOXF1_HUMAN       182 P----PNSLAL---EGGL-GM--M--NGHLPGNVDGMAL-PSHSV--P-H    215
                      |    .||: |   | .| .|  |  | .: .|:|  :| |.: |  . |
HCM1_YEAST        268 PQLKTDNSV-LNPHE-NLESMRNMIEN-DV-NNID--SLEPPY-VMKKYH    310

FOXF1_HUMAN       216 ----LPS--NG-GHSYMGGC--GG-AAAGEYPHHDSSVP---A-SP----    247
                          |||  |. .| :..|.  .. ..|..:    :::|   | ||
HCM1_YEAST        311 TSLGLPSLVNAKDH-FQAGVKNNNITQANRF----NTLPITSAKSPQNFR    355

FOXF1_HUMAN       248 ------------LLP----TGAGGVMEPHAV-YSGSAAAWP----PSASA    276
                                  |.|    .|||.:::|  : ||      |    ...:.
HCM1_YEAST        356 KYFTSFNSNFEDLSPLRSNVGAGSLLDP--LPYS------PLKLYDQKNL    397

FOXF1_HUMAN       277 AL----NSGASYIKQQ-PLSPCNPAANPLS-GS--LST----HS--LEQ-    311
                      ||    .|..||...| |  |  |   |.| ||  |.|    ||  ||:
HCM1_YEAST        398 ALMSKPQSQQSYSNSQLP--P--P---PSSHGSDLLKTPKMRHSDGLEKT    440

FOXF1_HUMAN       312 -------P-----YLH--QN-SH---N---APAEL-QGI--P-RY-----    331
                             |     .|.  |. ||   :   :|  | :.|  | ||
HCM1_YEAST        441 PSRLISTPKDGNSILRKWQTPSHLFEDLYCSP--LFRAIETPIRYITTPG    488

FOXF1_HUMAN       332 ---HSQ-SPSMCDRK----EFVF-S-FNA-MASSSM-----HS----A--    359
                         .:| ||    ||    : |. | .|: .|||.:     :|    |
HCM1_YEAST        489 GTLETQISP----RKSSAPD-VLTSATNSKFASSGLFGVDVYSVWKRATE    533

FOXF1_HUMAN       360 ----GGG---S----Y-YHQQVTYQD    373
                          |..   |    : ||...: .|
HCM1_YEAST        534 KISDGNNTTDSNQKHHPYHNHPS-ND    558

--------------------------------------------
Name 1: CLIP4_HUMAN
Name 2: NIP80_YEAST

CAP-Gly domain-containing linker protein 4 OS=Homo sapiens GN=CLIP4 PE=1 SV=1
Protein NIP100 OS=Saccharomyces cerevisiae (strain ATCC 204508 / S288c) GN=NIP10
0 PE=1 SV=2

Needed for following interaction(s):
yeast   genetic PTTG1_HUMAN     CLIP4_HUMAN     PTTG1   CLIP4   SECU_YEAST
NIP80_YEAST

Length sequence 1:      705
Length sequence 2:      868
Sequence #1: CLIP4_HUMAN
Sequence #2: NIP80_YEAST
Length #1: 957
Length #2: 957
Matrix: BLOSUM62
Gap open: 2.0
Gap extend: 1.0
Length: 957
Identity: 250/957 (26.12%)
Similarity: 356/957 (37.20%)
Gaps: 403/957 (42.11%)
Score: 663.00
```

```
CLIP4_HUMAN          3 IEDLPDFPL---EG--NPL--F----------GRYPF-IFSASDTPVI-F     33
                       || | |.||   :|  |.:  |          |.| : :|...||  : |
NIP80_YEAST         46 IE-L-DKPLGKNDGSANGIRYFDIDLKKANSNGGY-YGLFCKKDT--LQF     90

CLIP4_HUMAN         34 SISAAPMPSDCEFSFFDPNDA---------SCQEI---L----FD--P-K     64
                        ..    |.|.|.|..:.|.|         .|:.:   |    .:  . |
NIP80_YEAST         91 -YK----PDDDEHSLLNGNAAQETIKNLQVKCESLASKLNKIKIENHELK    135

CLIP4_HUMAN         65 TSV---S--E--LFA-ILR--QWVPQVQ-QNIDIIGNEILKRGC-NVN--    100
                       |||   |  |  |.: |.|  :.|.::: :|    ||  :|... |.|
NIP80_YEAST        136 TSVEKLSTNETVLLSKISRLDKLVKELKVEN----GN--MKTHLDNFNHL    179

CLIP4_HUMAN        101 -D-RDG-LT-DM---TLLHYTCKSGAH---GIGDVET------AVK----    130
                        | .|. :. |:   |||.   :|  |   |:.| :|      |:|
NIP80_YEAST        180 LDASDSVMAPDLDKGTLLE---RS--HLLQGLLD-QTKLSYDKAMKVQED    223

CLIP4_HUMAN        131 -FA--TQLID----LG---ADISLRSRWTNMNALHYAAYFDVPEL-IRVI    169
                        ..  |||::    |.   :|:.|:.:.|| |.:   .  |: .| |..
NIP80_YEAST        224 LLEENTQLLEENAVLSKKISDLGLQLQQTN-NTI---G--DL-ALQIEA-    265

CLIP4_HUMAN        170 LKTSKPKD-VD-ATCSDFN--FGTALHIAAYN-----LCAGAVKC---L-    206
                        : ||..: || .| :| |  . |: :|.|.|     |.| ..|.   |
NIP80_YEAST        266 -Q-SKSSNIVDKLT-ND-NILL-TS-NIKALNNELEELQA-KEKLDENLR    308

CLIP4_HUMAN        207 -----LEQG-----AN--PAFRNDKGQIPADV-VPD--PVDMPLEMADAA    241
                            |||.     :|  .|..|:| :| |.. :.:  .:...||..: |
NIP80_YEAST        309 ITYEQLEQELRLQLSNLQSALENEK-EI-AGTYIEENSRLKATLESIE-A    355

CLIP4_HUMAN        242 ATA-K----EIK----Q--M----LL-------D--AVP----LS-----    258
                       .|: |    |:|    |  :    ||       :  |.|    ||
NIP80_YEAST        356 KTSHKFQSLELKVNTLQEELYQNKLLKKFYQIYEPFAQPHLAALSSQLQY    405

CLIP4_HUMAN        259 -CN-I-SK--AMLPNYD-HVTGKAMLTSLGLKLGDRV-VIAGQKVGT---    298
                        .. | |:  ..|.|.: |:..| :|:|:...|  .: .|   | .|
NIP80_YEAST        406 LAEVIESENFGKLENIEIHIILK-VLSSISYAL--HIYTI---K-NTPDH    448

CLIP4_HUMAN        299 LRFCGTT-E-F----A--SGQWAGIE-LDEPEGKNNGSVGKVQ--YFK-C    336
                       |.   || : |    |  | .|.. | |   :.|.: |  | |  .|. |
NIP80_YEAST        449 LE---TTLQCFKVNIAPIS-MWLS-EFL---QRKFS-S--K-QETAFSIC    486

CLIP4_HUMAN        337 APKYGI----FAPLSK-ISKAKGRRKNITHTP--STKAAVPLIRSQKIDV    379
                         :: :    |  |.| ::..   .| |.| |  .|  .||     |: :
NIP80_YEAST        487 --QF-LEDNKF--LDKDVTLI---LK-ILH-PILET--TVP-----KL-L    518

CLIP4_HUMAN        380 AHV-T-SKVN---TGL-MTSKKDSASESTLSL----PP--G-EELKTVTE    416
                       |.: | |..|   | | :..   |..|.:|||    ..  | ||   :::
NIP80_YEAST        519 AFLRTNSNFNDNDT-LCLIG---SLYERSLSLIARIDKLIGKEE---ISK    561

CLIP4_HUMAN        417 KDVAL-LGSVSSC----SS--T---S-SLEH-RQSYP-----KK-----Q    444
                       :|..| |  ..||    ||  |   | :| . ||.|.     ||     |
NIP80_YEAST        562 QDNRLFL--YPSCDITLSSILTILFSDAL-FLRQDYKRISSLKKLEVFFQ    608

CLIP4_HUMAN        445 NAISS---NKKTM-SKSPS--LS---SRAS--AGLNSSATSTANNSRC-E    482
                        .|.|   | .|: .:.||  .|   |:.:  .| |.| .|..:: |. |
NIP80_YEAST        609 -GIESLLEN-ITIFPEQPSQQTSDSESQCNIKEG-NFS-NSLLSD-RLNE    653

CLIP4_HUMAN        483 GE-LRLGERVLVVGQR------LGT-IR-FFG------T--TNFAPGYWY    515
                        | :||.| |||  |:      |.| |: ..|      |  .|...   .
NIP80_YEAST        654 -ENIRLKE-VLV--QKENMLTELETKIKIIIGRDLERKTLEENIKT---L    696

CLIP4_HUMAN        516 GIELEKPHGKNDGSVGGVQYFS-CSPRYGIFAPPSRVQRVTD-SLDTLSE    563
                       .:||   :.||:.:.|..:..: .... . |   :.|.|:.: .| .|.:
NIP80_YEAST        697 KVEL---NNKNEENCGKTEILNKLKEE-N-F---NLVNRLKNMEL-KLYQ    737

CLIP4_HUMAN        564 ISSNKQ-NHSYPGF-RRSFSTTS-ASS--Q-KE-INRRNAFSKSK-AALR    605
                       |..|.. |..|  . |......: .|.  : :| | ||. ..:.| .::.
NIP80_YEAST        738 IKDNNTLNKIY--LDREKVDRVNLVSEIMELRETI-RRQ-IKEQKRVSID    783

CLIP4_HUMAN        606 RSW-SSTPTAGGIEGS--VK--L-HEGSQVLL-TSSNEMGTVRYVGPTDF    648
                       .|| ...| |  :|..  .|  : |   |   | |...||  ..:|. |
NIP80_YEAST        784 FSWLDELP-A--VENKQPFKEHINH--S---LDTLGIEM--FNFVS-T--    820

CLIP4_HUMAN        649 ASGIWLGLEL-RS-AKG----KNDGS-VG--DKRYFTCK-PNHGVLVR-P    687
                        |.| |.|:| :. |:.    :.|.| :.   ||    | .|    :| .
NIP80_YEAST        821 -SRI-LDLKLDQPLAEDELWHERDHSYISYL-KR----KRKN----IRLK    859

CLIP4_HUMAN        688 SR--VTY    692
                       |:  |||
NIP80_YEAST        860 SQNVVTY    866

--------------------------------------------
Name 1: ESPL1_HUMAN
Name 2: ESP1_YEAST

Separin OS=Homo sapiens GN=ESPL1 PE=1 SV=3
Separin OS=Saccharomyces cerevisiae (strain ATCC 204508 / S288c) GN=ESP1 PE=1 SV
=2

Needed for following interaction(s):
yeast   genetic PTTG1_HUMAN     ESPL1_HUMAN     PTTG1   ESPL1   SECU_YEAST
ESP1_YEAST
yeast   physical        ESPL1_HUMAN     PTTG1_HUMAN     ESPL1   PTTG1   ESP1_YEA
ST      SECU_YEAST

Length sequence 1:      2120
Length sequence 2:      1630
Sequence #1: ESPL1_HUMAN
Sequence #2: ESP1_YEAST
Length #1: 2381
Length #2: 2381
Matrix: BLOSUM62
Gap open: 2.0
Gap extend: 1.0
Length: 2381
Identity: 613/2381 (25.75%)
Similarity: 962/2381 (40.40%)
Gaps: 1033/2381 (43.39%)
Score: 1895.00

ESPL1_HUMAN         17 KEAEELLPALKEFLS-NPPAGFPSSRSDAERRQACDAILRACNQQLTAKL     65
                       |: ||  | |.| :| |.|  . :|:|         .:|   |..| :|:
ESP1_YEAST           4 KQ-EE--P-LNE-ISPNTP--M-TSKS---------YLL---NDTL-SKV     32

ESPL1_HUMAN         66 ACPRHLGSLLELAELACDGYLVSTPQRPPLYLERILFVLLRNAAAQ--GS    113
                          .|  |          |   .|  ||   |..   ||..:|::.   |
ESP1_YEAST          33 ---HH--S----------G---QT--RP---LTS---VLSGDASSNSIG-     55

ESPL1_HUMAN        114 PEATLRLAQPLHACLVQ-----CSR--EAAPQDYEAV--ARGS-FSLL--    151
                           : ||  :|..:::     .|.  :.|.:|...|  :..| :|||
ESP1_YEAST          56 ----I-LA--MHNNIIRDFTKIASNNIDLAIEDITTVDHSLNSIYSLLKS     98

ESPL1_HUMAN        152 ---WKG-AEALLERRAAFAARL---KALSFLVLLEDESTPCEVPH-FASP    193
                          | | ..:.::......:|   .||. |.     |:  |:.. | :.
ESP1_YEAST          99 HHMW-GHINSTVKQHLMIIVKLINNNALG-LA-----SS--EIIFLF-NE    138

ESPL1_HUMAN        194 TACRAVAAHQLFDASGHGL-N--EAD-A---D-FLDDL--LSRHVI--RA    231
                       |        .||.|  |.| |  .|| :   | :|.:|  |:..:|  |.
ESP1_YEAST         139 T--------NLFQA--HSLKNILLADFSTWNDYYLSNLKILALQIILKRK    178

ESPL1_HUMAN        232 LVGERGSSSGLLSPQRALCLLELTLEHCRRFCWSRHHDK--AISAVE---    276
                       ||.|      .| |.    :|||       |  |  |||  .:.  :
ESP1_YEAST         179 LVDE------YL-PH----ILEL-------F--S--HDKRYLLK--DPNL    204

ESPL1_HUMAN        277 KAHSYLRNTN--LA--PSLQL-CQ-L-GVKLLQ-VGE-EGP--QAVAKLL    315
                       |||: |  |.  |:   |:.. |: | |:|.|| :.: :.|  :.:.:.
ESP1_YEAST         205 KAHA-L--TKIVLSFF-SVTTSCKVLFGLKFLQYIKQFKLPFKKFISNIT    250

ESPL1_HUMAN        316 IKA-SA-VLSKS-ME-APSPPLRALY-ESCQFFLS-G-L-ERGTKRRYRL    357
                       ::. |. :|.|: :| .|:   : :| .|  |:|| . | : |      |
ESP1_YEAST         251 VECFSKNLLHKNYLEMGPN---K-IYLNS--FYLSYSMLYD-G------L    287

ESPL1_HUMAN        358 DAILSLFAFLGGYCSLLQ-QLRDDGVYGGSSKQQQSFLQMY--F-QG-LH    402
                       |.|: |...| .|....: | |  .:   .||::  |.: |  . :. |
ESP1_YEAST         288 DKIM-LLDIL-SYEETTEVQ-R--AI---KSKKE--FNE-YCNMSENRL-    325

ESPL1_HUMAN        403 LY---TV----VVYD----FAQ--GCQIVDLADLTQ-LVDSCKSTVVW-M    437
                       |:   :|    |:.:    |.|  | :.:. |.| : ||  |    :| .
ESP1_YEAST         326 LWSCISVDDLNVILENATNFLQNKG-KHIS-ATL-KCLV--C----LWST    366
```

```
ESPL1_HUMAN      438 LEALEGL-SGQE-LT--DHMGMTASY-TSNLAYSFYSHKLYAE-ACA-IS    480
                     :. |||| ..:: |.  |   .|..| .||:. |.  :.   | |.| :|
ESP1_YEAST       367 IR-LEGLPKNKDILRQFD---CTVIYINSNIK-SI--ND---ESAAALLS    406

ESPL1_HUMAN      481 EPLCQHLGLVKPGTYPEVPPEKLHRC--FRLQVESLKKLGKQAQGCKMV-    527
                     | |   ||::   :  |:       |  ::   |. |:|   :   .::
ESP1_YEAST       407 E-L---LGVL---S--EI-------CIDYK---EP-KRL---S---NIIS    430

ESPL1_HUMAN      528 ILWLAA-L-QPCSPEHMAEPVTFWVRVKMDAARAGDKELQLKTLRDSLSG    575
                     :|:.|: | :  |  |     :| : :|  .|   :.|:. ..|   :|.
ESP1_YEAST       431 VLFNASVLFK--S--H-----SF-L-LK--TA---NLEIS-NVL---ISN    460

ESPL1_HUMAN      576 WDPETLA--LLLREELQAYKAV-RADTGQER-------FNIICDLL--E-    612
                      |.:| :  .:|:.|    |.: .|.:.|::       ||:.| :|  :
ESP1_YEAST       461 -DSKT-SHRTILKFE----KFISSAQSAQKKIEIFSCLFNVYC-MLRNDT    503

ESPL1_HUMAN      613 LS---PE--ETPAGAWARA-THLVELAQVLCYHDFTQQTNCSALDAIREA    656
                     ||    :  :   .|:... |.| ::.:.:   :|   :| |: : |  .
ESP1_YEAST       504 LSFVF-DFCQ---NAFIHCFTRL-KITKFI---EF---SN-SS-E-I--M    537

ESPL1_HUMAN      657 LQLL---DSVR--PEAQAR-DQLLDDKAQALLWLYICTLEAKMQEGI-ER    699
                     |.:|   .|:.  | :: . .||    ::  : :: |:|  :   || :.
ESP1_YEAST       538 LSVLYGNSSIENIP-SE-NWSQL----SR--M-IF-CSL--R---GIFDL    572

ESPL1_HUMAN      700 DRRAQAPGNLEEFEVN--D----LN-YEDKLQEDRFLYSNIAFNLAADAA    742
                     |     |  | |.. |  |    || ||  |.  .|.:| : .||  |
ESP1_YEAST       573 D-----P--L-ELN-NTFDKLHLLNKYE--LL-IRIVY--L-LNL--D--    603

ESPL1_HUMAN      743 QSKCLDQALALWKELLTKGQAPAV-RCLQQT--AASLQILAALYQL--VA    787
                     .||.|...|:  |  :||  . .: :.||::  .|.  :| :: ::: |
ESP1_YEAST       604 MSKHLTTNLS--K--ITK--L-YINKWLQKSDEKAE-RI-SS-FEMDFV-    642

ESPL1_HUMAN      788 KPMQALEVLLLLRIVSERLKDHSKAAGSSCHITQLLLTLGC-PSYAQLHL    836
                     | |  |  |..|..  ... |  |.   |  | :  |:| | .|  :
ESP1_YEAST       643 K-M--L--LCYLNF--NNF-D--KL---S--I-E--LSL-CIKS--K---    668

ESPL1_HUMAN      837 EEAASSLKHLDQTTDTYLL---LSL-TCD--L-LRSQLYWTHQKVTKGVS    879
                     |:..||:  :.. .|.|||   ||| ..|  | :::||    || |  ::
ESP1_YEAST       669 EKYYSSI--VPY-ADNYLLEAYLSLYMIDDALMMKNQL----QK-T--MN    708

ESPL1_HUMAN      880 LLLSVLRDPALQKSSKAWYLLRVQVLQLVAAYLSLPSNNLSHSLWE-QLC    928
                       ||.    |     |    :. |.| |.|:  ||  .|: | ||: .|.
ESP1_YEAST       709 --LST----A-----K----IE-QAL-LHAS--SL--INV-H-LWDSDLT    735

ESPL1_HUMAN      929 A-Q---GWQT-----PEIALID---SHKL-LRSIILLLMGSDILSTQKAA    965
                     | |   | :|     ||  |.|   .|.| : |    |.   |    | .
ESP1_YEAST       736 AFQIYFG-KTLPAMKPE--LFDINNDHNLPM-S----LY---I----K-V    769

ESPL1_HUMAN      966 VETSFLDYGENLVQKWQVLSEVLSCSEKLVCHLGRLGSV-SEAKAF-CLE   1013
                     :   .|    | : |  :.:|    |.||  :: :.|:| | | .. |
ESP1_YEAST       770 I---LL----N-I-K--IFNE----SAKL--NI-KAGNVIS-A-VIDC--    797

ESPL1_HUMAN     1014 ALKLTTKLQIPRQCALFLVLKGELELARNDIDLCQ-SDLQQVLFLLESCT   1062
                       :   |.|   ..||.| ||   :  :|  .|.| |   : |.||:|.:
ESP1_YEAST       798 --R---KAQ---NLALSL-LK---K--KN--KLSQGS---R-LALLKSLS    827

ESPL1_HUMAN     1063 EFGGVTQHLDSVKKVHLQKGKQQAQVPCPPQLPEEELFLRGPALELVATV   1112
                      | ...| |  : |:|::.|  .|: .|  :...:|| .|     :::.:
ESP1_YEAST       828 -F-SFFQ-L--I-KIHIRIG--SAR-DC--EFYSKEL-SR-----IISDL    860

ESPL1_HUMAN     1113 AKEPGPIAPSTNSSPVLKTKPQPIPNFLSHSPTCDCSLCASPVLT-AVCL   1161
                      :|  ||.       |.:.    : :|| |      ...   ::| ..||
ESP1_YEAST       861 -EE--PII-------VYRC----L-HFL-H------RYY---MITEQTCL    885

ESPL1_HUMAN     1162 RWVLVTAGVRLAMGHQAQGLDLLQVVLKGCPEAAERLTQALQASLNHKTP   1211
                     :  .:|.| : |  ::|  .|.|..      | |: :|     ||   |
ESP1_YEAST       886 Q--NITLG-K-A--NKA--FDYLDA------E-AD-IT-----SL---T-    910

ESPL1_HUMAN     1212 PSLVPSLLDEILAQAYTLLALEGLNQPSNESLQKVLQSG--L-K-FVAAR   1257
                       :.  |.|.   :.:  :.||   |    ||  ||..|  | | |
ESP1_YEAST       911 --MF--LYDN---KEF--VKLE---Q----SL--VLYFGDQLEKTF----    938

ESPL1_HUMAN     1258 IPHLEPWRASLLLIWALTKLG-GL--SCCTTQLFASSWGWQPPLIKSVPG   1304
```

```
                     :|:|  |:        | .|| .:  |.|   |  |.  :.|   |:|
ESP1_YEAST       939 LPNL--WK--------L-HLGKDIDDSIC---L--SE--YMP---KNV--    965

ESPL1_HUMAN     1305 SEPSKTQG--QK-RSGRGRQKLASAPLRLNNTSQKGL-EGR-GLPCTPKP   1349
                       .::...  || .|    | |...|. .     ||: |.. |:| :..|
ESP1_YEAST       966 --INRVHNMWQKVMS----Q-LEEDPF-F-----KGMFESTLGIP-SSLP   1001

ESPL1_HUMAN     1350 PDRIRQAGPHVPFTVFEEVCPTESKPEVPQAPRVQQRVQTRLKVNFSDDS   1399
                        :      :|.|:     |  :  .:.:.|  .:. .|.||:  . ||
ESP1_YEAST      1002 ---V------IPSTM-----P--N--NILKTP--SKH-STGLKL--C-DS   1027

ESPL1_HUMAN     1400 DLEDPVSAEAWLAEEPKRRGTASRGRGRARKGLSLKTDAVVAPGSAPGNP   1449
                         |.|: : :.  |       ||: ..|:    |.|. :|   |
ESP1_YEAST      1028 ----PRSS-S-MT--P-------RGK-NIRQ----KFDR-IA---A----   1049

ESPL1_HUMAN     1450 GLNGRSRRAKKVASRHCEERRPQRASDQARPGPEIMRTIPEEEL-T-DNW   1497
                      :   |    |:           :   |.:   |::.::   :| | ||
ESP1_YEAST      1050 -I---S----KL-----------K---QMK---ELLESL---KLDTLDN-   1070

ESPL1_HUMAN     1498 RKMSFEILRGSDGEDSASGGKTPAPGPEAASGEWEL-LRLDSSKKKLPSP   1546
                     .::| :|   |    |.| ..|             | : | |:   :.|
ESP1_YEAST      1071 HELS-KI---S----SLS-SLT-------------LTI-L-SN---ITS-   1092

ESPL1_HUMAN     1547 CPDKES----D---KDLGPRLRLPSAPVATGL--STLDSICDSLSV-AFR   1586
                     ..:.||    :   .|| || .:   |:   |  ..|::| |:.:. .||
ESP1_YEAST      1093 IHNAESSLITNFSLTDL-PR-HM---PL---LFDKVLNNI-DNKNYREFR   1133

ESPL1_HUMAN     1587 GISHCPPSGLYAHLCRFLALCLGHRDP--YATAFLVTESVSITCRHQLLT   1634
                      :|    |     |   :|       |  .:|   :||  ||
ESP1_YEAST      1134 -VS----S-----L---IA-------PNNIST---ITE--SI--------   1150

ESPL1_HUMAN     1635 HLHRQLSKAQKHRGSL-EIADQLQGLSLQEMPGD-VPL-ARIQRLFSFRA   1681
                        | :|.|||   .| | :: | .:::..:  | .|: ..:  |.| :
ESP1_YEAST      1151 ---R-VSAAQK---DLME-SN-L-NINVITI--DFCPITGNL--LLS-K-   1184

ESPL1_HUMAN     1682 LESGHFPQPEKESFQERLALIPSGVTVCVLALATLQPGTVGNTLLLTR-L   1730
                     ||    |: .|.  :..|.| |         |  ::    .|    :| |
ESP1_YEAST      1185 LE----PR-RKR--RTHLRL-P---------L--IR----SN----SRDL   1207

ESPL1_HUMAN     1731 EKDSPPVSVQIP--TGQNKLHLRSVLNEFDAIQKAQKE--NS-SCT-DKR   1774
                       |.  |.:..|  |  .|| | |::||.:  |....|  |. . | ::|
ESP1_YEAST      1208 --DE--VHLSFPEAT--KKL-L-SIINESN--QTTSVEVTNKIK-TREER   1246

ESPL1_HUMAN     1775 E-WWTGRLALDHRMEVLIASLEKSVLGCW----KGLLLP----SS--EEP   1813
                     : |||.|..||.||:.|:.::|.|    |    :|...|    :|  |:
ESP1_YEAST      1247 KSWWTTRYDLDKRMQQLLNNIENS----WFNGVQGFFSPEVVDNSLFEK-   1291

ESPL1_HUMAN     1814 GPAQEASRLQELL-QDC-GWK-Y--P-------DRTLLKIMLSGAGALTP   1851
                       .::  :..|:| |:. ..| |  |       | .::::.|.    |.|
ESP1_YEAST      1292 --FKD--KFYEILHQNLPSRKLYGNPAMFIKVED-WVIELFLK----LNP   1332

ESPL1_HUMAN     1852 Q--D----IQALAY---------GLCPTQPERA-QE-----L---LNEAV   1877
                     |  |    ::.|.|         |    : |.| .|     |   |.|.:
ESP1_YEAST      1333 QEIDFLSKMEDLIYFVLDILLFHG----E-ENAYDEIDFSMLHVQLEEQI   1377

ESPL1_HUMAN     1878 GRLQGLTVPSNS--HLVLVLDKDLQKL-PWESMPS-LQALPVTRLPSF--   1921
                     .:.:. |:.:||  |..||:..... | |||.: | |:.|.:||:||:
ESP1_YEAST      1378 KKYRA-TMTTNSIFHTFLVVSSSCH-LFPWECL-SFLKDLSITRVPSYVC   1424

ESPL1_HUMAN     1922 --RFLLS-YSIIKEYGASPVLSQGV---DPR-STFYVLNPHN-NLSSTEE   1963
                       : ||| :    .| ..| | | |   | . |  .:||| | :||.||.
ESP1_YEAST      1425 LNK-LLSRF----HY-QLP-L-Q-VTIED-NIS--MILNP-NGDLSRTES   1461

ESPL1_HUMAN     1964 QFRANFSS--EAGWR--G--VVGEVPRPEQVQEALTK---H-DLYIYAGH   2003
                     :|:..|..  :|  :  .  |:.|  :||  :|.|.|   : :|::|.||
ESP1_YEAST      1462 KFKGMFQKIIDA--KPSSQLVMNE--KPE--EETLLKMLQNSNLFVYIGH   1505

ESPL1_HUMAN     2004 GAGARFLDGQAVLRLSCRAVA---LLFGCSSAALAVRGNLEGAGIVLKYI   2050
                     |.|.:::..:.: : .|..:|   || ||||||:...|.||..|.:..|:
ESP1_YEAST      1506 GGGEQYVRSKEI-K-KCTKIAPSFLL-GCSSAAMKYYGKLEPTGTIYTYL   1552

ESPL1_HUMAN     2051 MAGCPLFLGNLWDVTDRDIDRYTEALLQ--GW------LGAGPGAPL-L-   2090
                     :.|||:.|||||||||:|||:::|.|.:  |:      |. | .: | :
ESP1_YEAST      1553 LGGCPMVLGNLWDVTDKDIDKFSEELFEKMGFRCNTDDLN-G-NS-LSVS   1599
```

```
ESPL1_HUMAN     2091 YYVNQARQAPRLKYLIGAAPIAYGLP---VS   2118
                     |.|:::|....|:||.||||:.||||   ||
ESP1_YEAST      1600 YAVSKSRGVCHLRYLNGAAPVIYGLPIKFVS   1630

--------------------------------------------
Name 1: 1433E_HUMAN
Name 2: 1433E_DROME

14-3-3 protein epsilon OS=Homo sapiens GN=YWHAE PE=1 SV=1
14-3-3 protein epsilon OS=Drosophila melanogaster GN=14-3-3epsilon PE=1 SV=2

Needed for following interaction(s):
fly     genetic RASK_HUMAN      1433E_HUMAN     KRAS    YWHAE   RAS1_DROME
1433E_DROME

Length sequence 1:      255
Length sequence 2:      262
Sequence #1: 1433E_HUMAN
Sequence #2: 1433E_DROME
Length #1: 263
Length #2: 263
Matrix: BLOSUM62
Gap open: 2.0
Gap extend: 1.0
Length: 263
Identity: 219/263 (83.27%)
Similarity: 238/263 (90.49%)
Gaps: 12/263 (4.56%)
Score: 1111.00

1433E_HUMAN        1 MDDREDLVYQAKLAEQAERYDEMVESMKKVAGMDVELTVEERNLLSVAYK     50
                     |.:||:.||:|||||||||||||||:|||||.|||||||||||||||||||
1433E_DROME        1 MTERENNVYKAKLAEQAERYDEMVEAMKKVASMDVELTVEERNLLSVAYK     50

1433E_HUMAN       51 NVIGARRASWRIISSIEQKEENKGGEDKLKMIREYR-QMVETELKLICCD     99
                     ||||||||||||:||||||||||.|:||:||:.|| | ||.||:.||.|
1433E_DROME       51 NVIGARRASWRIITSIEQKEENKGAEEKLEMIKTYRGQ-VEKELRDICSD     99

1433E_HUMAN      100 ILDVLDKHLIPAANTGESKVFYYKMKGDYHRYLAEFATGNDRKEAAENSL    149
                     ||:||:|||||.|.:|||||||||||||||||||||||:|||:||||||
1433E_DROME      100 ILNVLEKHLIPCATSGESKVFYYKMKGDYHRYLAEFATGSDRKDAAENSL    149

1433E_HUMAN      150 VAYKAASDIAMTELPPTHPIRLGLALNFSVFYYEILNSPDRACRLAKAAF    199
                     :||||||||||.:||||||||||||||||||||||||||||||||||||||
1433E_DROME      150 IAYKAASDIAMNDLPPTHPIRLGLALNFSVFYYEILNSPDRACRLAKAAF    199

1433E_HUMAN      200 DDAIAELDTLSEESYKDSTLIMQLLRDNLTLWTSDMQ--------GDGEE    241
                     |||||||||||||||||||||||||||||||||||||        ||||.
1433E_DROME      200 DDAIAELDTLSEESYKDSTLIMQLLRDNLTLWTSDMQAEEVDPNAGDGEP    249

1433E_HUMAN      242 QNKEALQDVEDEN    254
                       ||.:||||||::
1433E_DROME      250 --KEQIQDVEDQD    260

--------------------------------------------
Name 1: RS16_HUMAN
Name 2: RS16_YEAST

40S ribosomal protein S16 OS=Homo sapiens GN=RPS16 PE=1 SV=2
40S ribosomal protein S16 OS=Saccharomyces cerevisiae (strain ATCC 204508 / S288
c) GN=RPS16A PE=1 SV=2

Needed for following interaction(s):
yeast   genetic RASK_HUMAN      RS16_HUMAN      KRAS    RPS16   IRA2_YEAST
RS16_YEAST

Length sequence 1:      146
Length sequence 2:      143
Sequence #1: RS16_HUMAN
Sequence #2: RS16_YEAST
Length #1: 146
Length #2: 146
Matrix: BLOSUM62
```

```
Gap open: 2.0
Gap extend: 1.0
Length: 146
Identity: 94/146 (64.38%)
Similarity: 117/146 (80.14%)
Gaps: 3/146 (2.05%)
Score: 497.00

RS16_HUMAN         1 MPSKGPLQSVQVFGRKKTATAVAHCKRGNGLIKVNGRPLEMIEPRTLQYK     50
                     | |..|  |||.||:||:||||||.|.|.|||||||.|:.::||..|::|
RS16_YEAST         1 M-SAVP--SVQTFGKKKSATAVAHVKAGKGLIKVNGSPITLVEPEILRFK     47

RS16_HUMAN        51 LLEPVLLLGKERFAGVDIRVRVKGGGHVAQIYAIRQSISKALVAYYQKYV    100
                     :.||:||:|.::|:.:||||||.|||||:|:||||:|:|.||||:||||
RS16_YEAST        48 VYEPLLLVGLDKFSNIDIRVRVTGGGHVSQVYAIRQAIAKGLVAYHQKYV     97

RS16_HUMAN       101 DEASKKEIKDILIQYDRTLLVADPRRCESKKFGGPGARARYQKSYR    146
                     ||.||.|:|.....||||||:||.||.|.|||||.|||:|:|||||
RS16_YEAST        98 DEQSKNELKKAFTSYDRTLLIADSRRPEPKKFGGKGARSRFQKSYR    143

--------------------------------------------
Name 1: GSK3A_HUMAN
Name 2: RIM11_YEAST

Glycogen synthase kinase-3 alpha OS=Homo sapiens GN=GSK3A PE=1 SV=2
Serine/threonine-protein kinase RIM11/MSD1 OS=Saccharomyces cerevisiae (strain A
TCC 204508 / S288c) GN=RIM11 PE=1 SV=1

Needed for following interaction(s):
yeast   physical        GSK3A_HUMAN     RASK_HUMAN      GSK3A   KRAS    RIM11_YE
AST     IRA2_YEAST

Length sequence 1:      483
Length sequence 2:      370
Sequence #1: GSK3A_HUMAN
Sequence #2: RIM11_YEAST
Length #1: 401
Length #2: 401
Matrix: BLOSUM62
Gap open: 2.0
Gap extend: 1.0
Length: 401
Identity: 210/401 (52.37%)
Similarity: 274/401 (68.33%)
Gaps: 71/401 (17.71%)
Score: 1066.00

GSK3A_HUMAN       82 AGTSFPPPGVKLGRD--SGK-VTTVVA----TLGQGP-ERSQEVAY-TDI    122
                     :..|   |  .|..:  | | |  ..|    |:  .| :..| ::: | .
RIM11_YEAST        5 SNNS---P--NLSNNIVS-KQV--YYAHPPPTI--DPNDPVQ-ISFPT-T     42

GSK3A_HUMAN      123 KVIGNGSFGVVYQAR-LAETRELVAIKKVLQDKRFKNRELQIMRKLDHCN    171
                     :|:|:||||||: |. :.||.|.||||||||||||||||:||:.|.|.|
RIM11_YEAST       43 EVVGHGSFGVVF-ATVIQETNEKVAIKKVLQDKRFKNRELEIMKMLSHIN     91

GSK3A_HUMAN      172 IVRLRYFFYSSGEK--KDELYLNLVLEYVPETVY-RVARHFTKAKLTIPI    218
                     |:.|:||||   |:  :||:||||:|||:|:::| |: |||...: | |:
RIM11_YEAST       92 IIDLKYFFY---ERDSQDEIYLNLILEYMPQSLYQRL-RHFVHQR-T-PM    135

GSK3A_HUMAN      219 --LYVKVYMYQLFRSLAYIHS-QGVCHRDIKPQNLLVDPDT-AVLKLCDF    264
                       |.:|.||:|||:||.|:|. ..|||||||||||||||:| : |||||
RIM11_YEAST      136 SRLEIKYYMFQLFKSLNYLHHFANVCHRDIKPQNLLVDPETWS-LKLCDF    184

GSK3A_HUMAN      265 GSAKQLVR-GEPNVSYICSRYYRAPELIFGATDYTSSIDVWSAGCVLAEL    313
                     |||||| : .||||||||||||||||||||:||:.||:||:|||:|||
RIM11_YEAST      185 GSAKQL-KPTEPNVSYICSRYYRAPELIFGATNYTNQIDIWSSGCVMAEL    233

GSK3A_HUMAN      314 LLGQPIFPGDSGVDQLVEIIKVLGTPTREQIREMNPNYTEFKFPQIKAHP    363
                     |||||:|||:||:|||||||:||||:::|..|||||.|.||||||..|
RIM11_YEAST      234 LLGQPMFPGESGIDQLVEIIKILGTPSKQEICSMNPNYMEHKFPQIKPIP    283

GSK3A_HUMAN      364 WTKVFK---SRTPPEAIALCSSLLEYTPSSRLSPLEA-CAHS-FFDELRC    408
                     .::|||   .:| .|.:|   .:|:|.|..|.:.|:. |  | :||||:
RIM11_YEAST      284 LSRVFKKEDDQT-VEFLA---DVLKYDPLERFNALQCLC--SPYFDELK-    326
```

```
GSK3A_HUMAN      409 L--G--TQLPNN-RPLPPL-F--NFSAGELSIQP-SLNAI---LIPPHLR    446
                     |  |  .|:..: : |  | |  |...|.||  | .|:::   |.|   :
RIM11_YEAST      327 LDDGKINQITTDLK-L--LEFDENVELGHLS--PDELSSVKKKLYP---K    368

GSK3A_HUMAN      447 S    447
                     |
RIM11_YEAST      369 S    369

--------------------------------------------
Name 1: PEX13_HUMAN
Name 2: PEX13_YEAST

Peroxisomal membrane protein PEX13 OS=Homo sapiens GN=PEX13 PE=1 SV=2
Peroxisomal membrane protein PAS20 OS=Saccharomyces cerevisiae (strain ATCC 2045
08 / S288c) GN=PEX13 PE=1 SV=1

Needed for following interaction(s):
yeast   genetic RASK_HUMAN      PEX13_HUMAN     KRAS    PEX13   IRA2_YEAST
PEX13_YEAST

Length sequence 1:      403
Length sequence 2:      386
Sequence #1: PEX13_HUMAN
Sequence #2: PEX13_YEAST
Length #1: 445
Length #2: 445
Matrix: BLOSUM62
Gap open: 2.0
Gap extend: 1.0
Length: 445
Identity: 149/445 (33.48%)
Similarity: 213/445 (47.87%)
Gaps: 147/445 (33.03%)
Score: 615.00

PEX13_HUMAN        1 MASQPPPPPKPWETRRIPGAG-PGPGPGPTFQ-SA-DLGPTLMT------     41
                     |:|...|.||||||    .|. ..|      | :| .|. .:||
PEX13_YEAST        1 MSSTAVPRPKPWET----SASLEEP------QRNAQSLS-AMMTSNQQDS     39

PEX13_HUMAN       42 RP------GQPALTRVPPPILPRP----SQQT-G-SSSV-----NT----     70
                     ||      ...| :...|.:||||    |..| | |:::      |:
PEX13_YEAST       40 RPTEESNNSNSA-SESAPEVLPRPAALNSSGTYGESNTIPGIYGNSNYGI     88

PEX13_HUMAN       71 -F--RPAYS--S-F-SS-G-YGAYGNSFYG-GY-SPYSY--GYN-GLGYN    106
                      :  .| ||  | : :| | || ||.|:|| .| | : |  ||. |.||.
PEX13_YEAST       89 PYDNNP-YSMNSIYGNSIGRYG-YGGSYYGNNYGS-F-YGGGYGAGAGYG    134

PEX13_HUMAN      107 RLRVDDLPPSRFVQQAEESSRGAFQSIESIVHA---FASVSMMMDATFSA    153
                        :::  .|. :  . ||::..||.|||::.|   ||   .|:::|:.|
PEX13_YEAST      135 ---MNN--GSG-L--G-ESTKATFQLIESLIGAVTGFA---QMLESTYMA    172

PEX13_HUMAN      154 VYNSFRAVLDVANHFSRLK--I-HFTKVFSAFALVRTI-RYLYRRLQ-RM    198
                     .:|||..::.||..|..||  : .|   |..||:::.: :.|||..: |
PEX13_YEAST      173 THNSFFTMISVAEQFGNLKEMLGSF---FGIFAIMKFLKKILYRATKGR-    218

PEX13_HUMAN      199 LGL--R-----RGSEN---EDL--WAESEGTVACLGAEDRAATSAK-SW-    234
                     ||:  :     .||:|   ||.  :.:| ||:   .:.:: ||..| ||
PEX13_YEAST      219 LGIPPKNFAESEGSKNKLIEDFQKFNDS-GTI---NSNEK-ATRRKISWK    263

PEX13_HUMAN      235 PIFLFF--AVILGGPYL----IWKLLSTHSDEVTDSINWAS---G-E--D    272
                     |: |||  || .|.|||    |.| |.| |.  |  |. ||   | |  |
PEX13_YEAST      264 PL-LFFLMAV-FGFPYLLNKFITK-LQT-SG--T--IR-ASQGNGSEPID    304

PEX13_HUMAN      273 -DHV-VARAEYDFAAVSE--E-EISFRAGDMLNLA-LKEQQPKV-R-G--    312
                      ..: .|||.|||  |.|  | |::.:.|| | :| |.::.| : | .
PEX13_YEAST      305 PSKLEFARALYDF--VPENPEMEVALKKGD-L-MAILSKKDP-LGRDSDW    349

PEX13_HUMAN      313 WLLASLDGQTTGLIPANYVKILGKRKGRKTVESSKVSKQQQSFTN    357
                     |.:.:.:| ..|.||.||::|: ||  ||.:|  .|..:.:  |:
PEX13_YEAST      350 WKVRTKNG-NIGYIPYNYIEII-KR--RKKIE--HVDDETR--TH    386

--------------------------------------------
Name 1: DPOD1_HUMAN
Name 2: DPOD_YEAST

DNA polymerase delta catalytic subunit OS=Homo sapiens GN=POLD1 PE=1 SV=2
DNA polymerase delta catalytic subunit OS=Saccharomyces cerevisiae (strain ATCC
204508 / S288c) GN=POL3 PE=1 SV=3

Needed for following interaction(s):
yeast   genetic DPOD1_HUMAN     MUS81_HUMAN     POLD1   MUS81   DPOD_YEAST
MUS81_YEAST

Length sequence 1:      1107
Length sequence 2:      1097
Sequence #1: DPOD1_HUMAN
Sequence #2: DPOD_YEAST
Length #1: 1165
Length #2: 1165
Matrix: BLOSUM62
Gap open: 2.0
Gap extend: 1.0
Length: 1165
Identity: 566/1165 (48.58%)
Similarity: 759/1165 (65.15%)
Gaps: 155/1165 (13.30%)
Score: 2856.00

DPOD1_HUMAN        4 KRRP---GPG--P-G----VPP----K-RARGGLW--DDDD--APR-PSQ     33
                     ||:.   |.|  | .    :|.    | .::|  :  .|.|  ..: .|.
DPOD_YEAST        27 KRQSIDHGVGSEPVSTIEIIPSDSFRKYNSQG--FKAKDTDLMGTQLEST     74

DPOD1_HUMAN       34 FEEDLALMEEMEAEHRLQEQEEEELQSVLEGVADGQVPPSAIDPRWLRPT     83
                     ||:|::   :|  ||.:.:|||.:|.| .|      :       .: | ||
DPOD_YEAST        75 FEQDVS---QM--EHDMADQEEHDLSS-FE-----R-------KK-L-PT    104

DPOD1_HUMAN       84 PPALDPQTEPL--I-FQQLEIDHYVGPA-QPVPGGPPPSRGSVPVLRAFG    129
                     .  .|| :  |  | |||  ||     | |.|..|......|. |:|.||
DPOD_YEAST       105 D--FDP-S--LYDISFQQ--ID-----AEQSVLNGIKDENTST-VVRFFG    141

DPOD1_HUMAN      130 VTDEGFSVCCHIHGFAPYFYTPAPPGFGPEHMGDLQRELN-LA--ISRDS    176
                     ||.||.||.|::.||..|.|.|| |. ..: ..| |.::| ..  ::
DPOD_YEAST       142 VTSEGHSVLCNVTGFKNYLYVPA-PN-SSD-AND-QEQINKFVHYLN---    184

DPOD1_HUMAN      177 RGGRELT-GPAVLAVELCSRESMFGYHGHGPSPFLRITVALPRLVAPARR    225
                         | | ..|:.::|:.|::|::||.|....||.:|.|..|.:|...|.
DPOD_YEAST       185 ----E-TFDHAIDSIEVVSKQSIWGYSGDTKLPFWKIYVTYPHMVNKLRT    229

DPOD1_HUMAN      226 LLEQG-I--R--VAGLGTPSFAPYEANVDFEIRFMVDTDIVGCNWLELPA    270
                     ..|:| :  .  .:. ||.:    |: |:.:.:|.|||..|||.:|:.||.
DPOD_YEAST       230 AFERGHLSFNSWFSN-GTTT---YD-NIAYTLRLMVDCGIVGMSWITLPK    274

DPOD1_HUMAN      271 GKYALRLKE---KATQCQLEADVLWSDVVSHPPEGPWQRIAPLRVLSFDI    317
                     |||:: : |   :.:.||||..:.:.:::||.||.|...||||::||||
DPOD_YEAST       275 GKYSM-I-EPNNRVSSCQLEVSINYRNLIAHPAEGDWSHTAPLRIMSFDI    322

DPOD1_HUMAN      318 ECAGRKGIFPEPERDPVIQICS-LGLRWGEPEPFLRLALTLRPCAPILGA    366
                     |||||.|:|||||.||||||.: :.:. |..:||:|...||..|:||.|:
DPOD_YEAST       323 ECAGRIGVFPEPEYDPVIQIANVVSIA-GAKKPFIRNVFTLNTCSPITGS    371

DPOD1_HUMAN      367 KVQSYEKEEDLLQAWSTFIRI-MDPDVITGYNIQNFDLPYLISRAQTLKV    415
                     .:.|:..||::|..|..|| | :|||||.|||..|||:|||::|||:.|||
DPOD_YEAST       372 MIFSHATEEEMLSNWRNFI-IKVDPDVIIGYNTTNFDIPYLLNRAKALKV    420

DPOD1_HUMAN      416 QTFPFLGRVAGLCSNIRDSSFQSKQTGRRDTKVVSMV-GRVQMDMLQVLL    464
                     ..||:.||:..:...|::|.|.||..|.|:||.|: : ||:|:|:||.:.
DPOD_YEAST       421 NDFPYFGRLKTVKQEIKESVFSSKAYGTRETKNVN-IDGRLQLDLLQFIQ    469

DPOD1_HUMAN      465 REYKLRSYTLNAVSFHFLGEQKEDVQH-SIITDLQNGND-QTRRRLAVYC    512
                     |||||||||||||.|||||||||| | |||:||||| | :|||||||||
DPOD_YEAST       470 REYKLRSYTLNAVSAHFLGEQKEDV-HYSIISDLQNG-DSETRRRLAVYC    517

DPOD1_HUMAN      513 LKDAYLPLRLLERLMVLVNAVEMARVTGVPLSYLLSRGQQVKVVSQLLRQ    562
                     ||||||||||:|:||.|||..|||||||||.||||:||||:||||||.|:
DPOD_YEAST       518 LKDAYLPLRLMEKLMALVNYTEMARVTGVPFSYLLARGQQIKVVSQLFRK    567

DPOD1_HUMAN      563 AMHEGL--LMPVVKSEGGED-YTGATVIEPLKGYYDVPIATLDFSSLYPS    609
```

```
                      .: | :  ::|.::|:..:| |.|||||||::||||||||||||:|||||
DPOD_YEAST        568 CL-E-IDTVIPNMQSQASDDQYEGATVIEPIRGYYDVPIATLDFNSLYPS    615

DPOD1_HUMAN       610 IMMAHNLCYTTLLRPGTAQKLGL--TEDQFIRTPTGDEFVKTSVRKGLLP    657
                      |||||||||||....|.::|.|  .|| ::.||.||.||.|..|:|:||
DPOD_YEAST        616 IMMAHNLCYTTLCNKATVERLNLKIDED-YVITPNGDYFVTTKRRRGILP    664

DPOD1_HUMAN       658 QILENLLSARKRAKAELAKETDPLRRQVLDGRQLALKVSANSVYGFTGAQ    707
                      .||:.|:|||||||.:|..|.||.:|.||:|||||||:||||||||||.
DPOD_YEAST        665 IILDELISARKRAKKDLRDEKDPFKRDVLNGRQLALKISANSVYGFTGAT    714

DPOD1_HUMAN       708 VGKLPCLEISQSVTGFGRQMIEKTKQLVESKYTVENGYSTSAKVVYGDTD    757
                      |||||||.||.|||.:||.||.|||..|:.||.::|||...|.|||||||
DPOD_YEAST        715 VGKLPCLAISSSVTAYGRTMILKTKTAVQEKYCIKNGYKHDAVVVYGDTD    764

DPOD1_HUMAN       758 SVMCRFGVSSVAEAMALGREAADWVSGHFPSPIRLEFEKVYFPYLLISKK    807
                      |||.:||.:.:.|||.||.|||.:||..|..||.|||||.||||||||:||
DPOD_YEAST        765 SVMVKFGTTDLKEAMDLGTEAAKYVSTLFKHPINLEFEKAYFPYLLINKK    814

DPOD1_HUMAN       808 RYAGLLFSSRPDAHDRMDCKGLEAVRRDNCPLVA---NLVTASLRRLLID    854
                      |||| ||.:.||..|::|.|||.:||||:|.||:   |.|   |:::||:
DPOD_YEAST        815 RYAG-LFWTNPDKFDKLDQKGLASVRRDSCSLVSIVMNKV---LKKILIE    860

DPOD1_HUMAN       855 RDPEGAVAHAQDVISDLLCNRIDISQLVITKELTRAASDYAGKQAH-VEL    903
                      |:.:||:|..::.|:|:|.||:|||:|:|:|  | .|.:|...|.| | |
DPOD_YEAST        861 RNVDGALAFVRETINDILHNRVDISKLIISK--T-LAPNYTNPQPHAV-L    906

DPOD1_HUMAN       904 AERMRKRDPGSAPSLGDRVPYVIISA-AKGVAAYMKSEDPLFVLEHSLPI    952
                      ||||::|: |..|::||||.||||.. .| :  |.::|||||||||::::.:
DPOD_YEAST        907 AERMKRRE-GVGPNVGDRVDYVIIGGNDK-L--YNRAEDPLFVLENNIQV    952

DPOD1_HUMAN       953 DTQYYLEQQLAKPLLRIFEPILGEGRAEAVLLRGDHTRCKTV-L-TG--K    998
                      |::|||..||..|::.|..||:|: : :|   .|... .|:: : ||  |
DPOD_YEAST        953 DSRYYLTNQLQNPIISIVAPIIGD-K-QA---NGMFV-VKSIKINTGSQK    996

DPOD1_HUMAN       999 VGGLLAFAKR-RNC--CIG-CRTVLSHQGAVCEFCQPRESELYQKEVSH-   1043
                       |||::|.|: ..|  |.| .|   ..:|.:|..|..|..|||.|.: :
DPOD_YEAST        997 -GGLMSFIKKVEACKSCKGPLR---KGEGPLCSNCLARSGELYIKAL-YD   1041

DPOD1_HUMAN      1044 LNALEERFSRLWTQCQRCQGSLHEDVICTSRDCPIFYMRKKVRKDLEDQ-   1092
                      :..|||::||||||||||.|:||.:|:|::::|.|||||.||:|:|:::
DPOD_YEAST       1042 VRDLEEKYSRLWTQCQRCAGNLHSEVLCSNKNCDIFYMRVKVKKELQEKV   1091

DPOD1_HUMAN      1093 EQLLRRFGPPGPEAW   1107
                      || |.:        |
DPOD_YEAST       1092 EQ-LSK--------W   1097

----------------------------------------------
Name 1: BOP1_HUMAN
Name 2: ERB1_YEAST

Ribosome biogenesis protein BOP1 OS=Homo sapiens GN=BOP1 PE=1 SV=2
Ribosome biogenesis protein ERB1 OS=Saccharomyces cerevisiae (strain ATCC 204508
 / S288c) GN=ERB1 PE=1 SV=1

Needed for following interaction(s):
yeast   physical        BOP1_HUMAN      MUS81_HUMAN     BOP1    MUS81   ERB1_YEA
ST      MUS81_YEAST

Length sequence 1:      746
Length sequence 2:      807
Sequence #1: BOP1_HUMAN
Sequence #2: ERB1_YEAST
Length #1: 860
Length #2: 860
Matrix: BLOSUM62
Gap open: 2.0
Gap extend: 1.0
Length: 860
Identity: 353/860 (41.05%)
Similarity: 496/860 (57.67%)
Gaps: 169/860 (19.65%)
Score: 1744.00
```

```
BOP1_HUMAN          1 MAGSRGAGRTA-AP-SVRPEKR-RSEPELEPEPEPEPPLLCTSPL--SHS     45
                      ||  :. .:|. |. |   :|| .|| |.:.| |.|..||....|  :.:
ERB1_YEAST          2 MA--KN-NKTTEAKMS---KKRAASE-ESDVE-EDEDKLLSVDGLIDAEA     43

BOP1_HUMAN         46 TGSDSGVSDSE-ES-VFSGLE-DSGS-----DSSEDD-D-E-----GDEE     80
                      :.||.  .|.| || | .  | :|.|     |.|:|| | |     .:||
ERB1_YEAST         44 SESDE--DDDEYESAV-E--EKESSSDKEAQDDSDDDSDAELNKLLAEEE     88

BOP1_HUMAN         81 GEDGALD-D--EGHSGIKKTTE--EQ---VQAST--PCPR--TEMA--SA    116
                      | ||..| |  | .|  ..||.  ::   |:..|  . |.  ::.|  |.
ERB1_YEAST         89 G-DGEEDYDSSE-FS--DDTTSLTDRLSGVKLQTIVD-PNIYSKYADGSD    133

BOP1_HUMAN        117 R-IGDE----Y-AEDSSDEEDIRNTVGNVPLEWYDDFPHVGYDLDGRRIY    160
                      | |..|    | ::| ||.| .:||:||:||..||:.||:|||::|:||.
ERB1_YEAST        134 RIIKPEINPVYDSDD-SDAE-TQNTIGNIPLSAYDEMPHIGYDINGKRIM    181

BOP1_HUMAN        161 KPLRTRDELDQFLDKMDDPDYWRT-VQDPMTGRDLRLTDEQVALVRRLQ-    208
                      :|.: ...|||.||.::.|:.| | :.|..:|..|.||.|::.|:.::|
ERB1_YEAST        182 RPAK-GSALDQLLDSIELPEGW-TGLLDKNSGSSLNLTKEELELISKIQR    229

BOP1_HUMAN        209 SGQFGDVGFNPYEPAVDFFS--GDVMIHPVTNRPADKRSFIPSLVEK-E-    254
                      :.|..| ..|||||.:|:|:  .:||  |:|..|..||.|:||   | |
ERB1_YEAST        230 NEQTDD-SINPYEPLIDWFTRHEEVM--PLTAVPEPKRRFVPS---KNEA    273

BOP1_HUMAN        255 K-VSRMVHAIKMG-WIQPR-----RPRDPTPSF-YDLWAQE-DPNAVLGR    295
                      | |.::|.||:.| .|.|:      :.::...:: ||||... :.|    .
ERB1_YEAST        274 KRVMKIVRAIREGRIIPPKKLKEMKEKEKIENYQYDLWGDSTETN----D    319

BOP1_HUMAN        296 HKMHVPAPKLALPGHAESYNPPPEYLLSEEERLAWEQQEPGERKLSFLPR    345
                      |.||:.||||..|.:.||||||.|||||.||:.|||..|..||:.:|:|:
ERB1_YEAST        320 HVMHLRAPKLPPPTNEESYNPPEEYLLSPEEKEAWENTEYSERERNFIPQ    369

BOP1_HUMAN        346 KFPSLRAVPAYGRFIQERFERCLDLYLCPRQRKMRVNVDPEDLIPKLPRP    395
                      |:.:||.||.||..|:|||||.|||||.||.||.::|:||..|||:||.|
ERB1_YEAST        370 KYSALRKVPGYGESIRERFERSLDLYLAPRVRKNKLNIDPNSLIPELPSP    419

BOP1_HUMAN        396 RDLQPFP-TCQALVYRGHSDLVRCLSVSPGGQWLVSGSDDGSLRLWEVAT    444
                      :||:||| .| :.:|.||...||.||:.|.|.||.:|||||::|:||:.|
ERB1_YEAST        420 KDLRPFPIRC-STIYAGHKGKVRTLSIDPSGLWLATGSDDGTVRVWEILT    468

BOP1_HUMAN        445 ARCV-RT--V-----PVGGV-VKSVAWNPSPA---VCLVAAAV-EDSV-L    480
                      .|.| ||  :     |  .. ::.:.|||. |   : | |.|| | :: |
ERB1_YEAST        469 GREVYRTTLIDDEENP--DYHIECIEWNPD-ANNGI-L-AVAVGE-NIHL    512

BOP1_HUMAN        481 LLNPALG-DRLVA--GST---DQL-LSAF--VPP--------------E    506
                      ::.|..| |  :.  |.|   |.. ...|  |..              |
ERB1_YEAST        513 IVPPIFGYD--IENNGKTKIEDGFGYDTFGTVKKSNLEVNENGDGDEDGE    560

BOP1_HUMAN        507 -EP---PL--QPARWLEASE-E-ERQVGLRLRI-CHGKPVTQVTWHGR-G    546
                       |.   .:  |.|:|.:.|: : |:.: . :.| |. |.|.::|| | |
ERB1_YEAST        561 NESAKNAVKKQVAQWNKPSQKQLEKDI-C-ITISCK-KTVKKLSWH-RKG    606

BOP1_HUMAN        547 DYLAVVLAT-Q---GHTQVLIHQLSRRRSQSPFRRSHGQVQRV--A-FHP    589
                      ||.  |  | |   |:|.|||||:|:..:||||::|.| :  :  | |||
ERB1_YEAST        607 DYF--V--TVQPDSGNTSVLIHQVSKHLTQSPFKKSKG-I--IMDAKFHP    649

BOP1_HUMAN        590 ARPFLLVASQRSVRLYHLLRQE-LTKKLMPNCKWVSSLAVHPAGDNVICG    638
                      .:|.|.|.|||.||:|. |.|: |.|||:|..:|:|.:.:||.|||:|..
ERB1_YEAST        650 FKPQLFVCSQRYVRIYD-LSQQILVKKLLPGARWLSKIDIHPRGDNLIAS    698

BOP1_HUMAN        639 SYDSKLVWFDLDL-STKPYRMLRHHKKALRAVAFHPRYPLFASGSDDGSV    687
                      |:|.:::|.|||| || ||:.||:|:||:|.||.:.|||:|.:|||::
ERB1_YEAST        699 SFDKRVLWHDLDLAST-PYKTLRYHEKAVRSVNFHKKLPLFSSAADDGTI    747

BOP1_HUMAN        688 IVCHGMVYNDLLQNPLLVPVKVLKGH-VLTRDLGVLDVIFHPTQPWVFSS    736
                      .|.|..||:|:::||::||:|.|.|| |: ..|||||.|:||.:.|:||:
ERB1_YEAST        748 HVFHATVYDDMMKNPMIVPLKKLTGHKVI-NSLGVLDAIWHPREAWLFSA    796

BOP1_HUMAN        737 GADGTVRLFT    746
                      |||.|.||:|
ERB1_YEAST        797 GADNTARLWT    806

----------------------------------------------
Name 1: PP2AB_HUMAN
```

```
Name 2: PP2A2_YEAST

Serine/threonine-protein phosphatase 2A catalytic subunit beta isoform OS=Homo s
apiens GN=PPP2CB PE=1 SV=1
Serine/threonine-protein phosphatase PP2A-2 catalytic subunit OS=Saccharomyces c
erevisiae (strain ATCC 204508 / S288c) GN=PPH22 PE=1 SV=1

Needed for following interaction(s):
yeast   genetic BLM_HUMAN       PP2AB_HUMAN     BLM     PPP2CB  SGS1_YEAST
PP2A2_YEAST

Length sequence 1:      309
Length sequence 2:      377
Sequence #1: PP2AB_HUMAN
Sequence #2: PP2A2_YEAST
Length #1: 322
Length #2: 322
Matrix: BLOSUM62
Gap open: 2.0
Gap extend: 1.0
Length: 322
Identity: 234/322 (72.67%)
Similarity: 273/322 (84.78%)
Gaps: 19/322 (5.90%)
Score: 1319.00

PP2AB_HUMAN        2 DDKA-----FT----KELDQWVEQLNECKQLNENQV-RTLCEK-AKEILT     40
                     |.|:     .|    .:||||:|.|::|:.|:|:.| | || | |.::|
PP2A2_YEAST       61 DHKSSKPLKLTNTNINQLDQWIEHLSKCEPLSEDDVAR-LC-KMAVDVL-    107

PP2AB_HUMAN       41 K-ESNVQEVRCPVTVCGDVHGQFHDLMELFRIGGKSPDTNYLFMGDYVDR     89
                     : |.||:.:..|||:|||||||||||:|||:|||..||||||||||||||
PP2A2_YEAST      108 QFEENVKPINVPVTICGDVHGQFHDLLELFKIGGPCPDTNYLFMGDYVDR    157

PP2AB_HUMAN       90 GYYSVETVTLLVALKVRYPERITILRGNHESRQITQVYGFYDECLRKYGN    139
                     ||||||||:.|||:|||||.|||||||||||||||||||||||||||||:
PP2A2_YEAST      158 GYYSVETVSYLVAMKVRYPHRITILRGNHESRQITQVYGFYDECLRKYGS    207

PP2AB_HUMAN      140 ANVWKYFTDLFDYLPLTALVDGQIFCLHGGLSPSIDTLDHIRALDRLQEV    189
                     |||||.|||||||.|:|||||.:||||||||||.|:|:|.:|.|:|:|||
PP2A2_YEAST      208 ANVWKMFTDLFDYFPVTALVDNKIFCLHGGLSPMIETIDQVRDLNRIQEV    257

PP2AB_HUMAN      190 PHEGPMCDLLWSDPDDRGGWGISPRGAGYTFGQDISETFNHANGLTLVSR    239
                     ||||||||||||||||||||||||||||:||||||||.|||.|.|:|::|
PP2A2_YEAST      258 PHEGPMCDLLWSDPDDRGGWGISPRGAGFTFGQDISEQFNHTNDLSLIAR    307

PP2AB_HUMAN      240 AHQLVMEGYNWCHDRNVVTIFSAPNYCYRCGNQAAIMELDDTLKYS--FL    287
                     |||||||||:|.|.:||||||||||||||||||||||||:|:  .::  ||
PP2A2_YEAST      308 AHQLVMEGYSWSHQQNVVTIFSAPNYCYRCGNQAAIMEVDE--NHNRQFL    355

PP2AB_HUMAN      288 QFDPAPRRGEPHVTRRTPDYFL    309
                     |:||:.|.|||.|||:||||||
PP2A2_YEAST      356 QYDPSVRPGEPTVTRKTPDYFL    377

--------------------------------------------
Name 1: MED31_HUMAN
Name 2: MED31_YEAST

Mediator of RNA polymerase II transcription subunit 31 OS=Homo sapiens GN=MED31
PE=1 SV=1
Mediator of RNA polymerase II transcription subunit 31 OS=Saccharomyces cerevisi
ae (strain ATCC 204508 / S288c) GN=SOH1 PE=1 SV=1

Needed for following interaction(s):
yeast   genetic MED31_HUMAN     RASK_HUMAN      MED31   KRAS    MED31_YEAST
IRA2_YEAST

Length sequence 1:      131
Length sequence 2:      127
Sequence #1: MED31_HUMAN
Sequence #2: MED31_YEAST
Length #1: 135
Length #2: 135
Matrix: BLOSUM62
Gap open: 2.0
Gap extend: 1.0
Length: 135
Identity: 49/135 (36.30%)
Similarity: 73/135 (54.07%)
Gaps: 26/135 (19.26%)
Score: 222.00

MED31_HUMAN        1 MAA----AVAMETDDAGNRL--RFQLELEFVQCLANPNYLNF-LAQRGYF     43
                     |::    |.|..:.|. |.|  ||::||||:|.|||..|:.: |.|:..:
MED31_YEAST        1 MSSTNGNAPATPSSDQ-NPLPTRFEVELEFIQSLANIQYVTYLLTQQQIW     49

MED31_HUMAN       44 KDKAFVNYLKYLLYWKDPEYAKYLKYPQCLHMLELLQYEHFRKE--LVNA     91
                     |...|.||||||.||.:|.|::.:.||.||.:|:||  ..| .|  :||.
MED31_YEAST       50 KSPNFKNYLKYLEYWCNPPYSQCIVYPNCLFILKLL--NGF-MESAIVNE     96

MED31_HUMAN       92 Q-CAKFIDE-QQI--L---HWQHYSRKRMRLQQ-A    118
                     . ..:.:|| .:|  |   .|.:    .| ::: |
MED31_YEAST       97 DGLLEGLDELPKIIQLQGPQWMN----EM-VERWA    126

--------------------------------------------
Name 1: MARE1_HUMAN
Name 2: BIM1_YEAST

Microtubule-associated protein RP/EB family member 1 OS=Homo sapiens GN=MAPRE1 P
E=1 SV=3
Protein BIM1 OS=Saccharomyces cerevisiae (strain ATCC 204508 / S288c) GN=BIM1 PE
=1 SV=1

Needed for following interaction(s):
yeast   genetic MARE1_HUMAN     RASK_HUMAN      MAPRE1  KRAS    BIM1_YEAST
IRA2_YEAST

Length sequence 1:      268
Length sequence 2:      344
Sequence #1: MARE1_HUMAN
Sequence #2: BIM1_YEAST
Length #1: 330
Length #2: 330
Matrix: BLOSUM62
Gap open: 2.0
Gap extend: 1.0
Length: 330
Identity: 125/330 (37.88%)
Similarity: 174/330 (52.73%)
Gaps: 94/330 (28.48%)
Score: 554.00

MARE1_HUMAN        7 STSVTSDNLSRHDMLAWINESLQLNLT--KIEQLC-SGAAYCQFMDMLFP     53
                     |..: .:  ||.::|.|:| .| |||.  |||: | :||||||.||.::
BIM1_YEAST         2 SAGI-GE--SRTELLTWLN-GL-LNLNYKKIEE-CGTGAAYCQIMDSIY-     44

MARE1_HUMAN       54 GSIALKKVKFQAKLEHEYIQ-NFKILQAGFKRMGVDKIIPVDKLVKGKFQ    102
                     |.:.:.:|||.|..|:|: | |:||||:.|.|.|::|.:.||||::.|||
BIM1_YEAST        45 GDLPMNRVKFNATAEYEF-QTNYKILQSCFSRHGIEKTVYVDKLIRCKFQ     93

MARE1_HUMAN      103 DNFEFVQWFKKFFDANYDGKD---YDPVAARQGQETAVAPSLVAPALNKP    149
                     ||.||:||.||.: ..:  ||   ||| .||  ::  ..| ::..  |..
BIM1_YEAST        94 DNLEFLQWLKKHW-IRH--KDESVYDP-DAR--RK--YRP-IITN--NSA    132

MARE1_HUMAN      150 KKPLT-SS-SAAPQRPISTQRTAAAPKAGPGV-VRKNP-GVGNG------    189
                     .||.| |: :.| :|..|| .|.:| .:| |: .|.:. |: ||
BIM1_YEAST       133 TKPRTVSNPTTA-KRSSST-GTGSA-MSG-GLATRHSSLGI-NGSRKTSV    177

MARE1_HUMAN      190 -DDE--A--AEL--MQQ-V---N--V--LKLTVEDLEKERDFYFGKLRNI    224
                      ..:  |  |||  .|: :   |  :  .|.||..||.||:|||.|||:|
BIM1_YEAST       178 TQGQLVAIQAELTKSQETIGSLNEEIEQYKGTVSTLEIEREFYFNKLRDI    227

MARE1_HUMAN      225 E-LI--CQE--NEG--E-ND-PV----------LQR-I--VD-ILYATDE    251
                     | |:  .|:  |||  : || .:          |.| :  |: |||||.|
BIM1_YEAST       228 EILVHTTQDLINEGVYKFNDETITGHGNGNGGALLRFVKKVESILYATAE    277

MARE1_HUMAN      252 GFVI---PDE------G--G--PQEEQEEY    268
                     ||.:   .||      |  |  |  .|..|
```

```
BIM1_YEAST       278 GFEMNDGEDELNDKNLGEHGTVP--NQGGY    305

--------------------------------------------

Name 1: MRCKA_HUMAN
Name 2: HSL1_YEAST

Serine/threonine-protein kinase MRCK alpha OS=Homo sapiens GN=CDC42BPA PE=1 SV=1
Probable serine/threonine-protein kinase HSL1 OS=Saccharomyces cerevisiae (strai
n ATCC 204508 / S288c) GN=HSL1 PE=1 SV=1

Needed for following interaction(s):
yeast   genetic PTTG1_HUMAN     MRCKA_HUMAN     PTTG1   CDC42BPA        SECU_YEA
ST      HSL1_YEAST

Length sequence 1:      1732
Length sequence 2:      1518
Sequence #1: MRCKA_HUMAN
Sequence #2: HSL1_YEAST
Length #1: 2051
Length #2: 2051
Matrix: BLOSUM62
Gap open: 2.0
Gap extend: 1.0
Length: 2051
Identity: 526/2051 (25.65%)
Similarity: 836/2051 (40.76%)
Gaps: 855/2051 (41.69%)
Score: 1694.00

MRCKA_HUMAN        1 MSGEV-RLRQLEQFILDG-PAQ-TNGQCFSVETLLDILICLYDECNNSPL     47
                     |:|.| :...:.:    | |:. .. :. :...:..:        |::|
HSL1_YEAST         1 MTGHVSKTSHVPK----GRPSSLAK-KA-AKRAMAKV--------NSNP-     35

MRCKA_HUMAN       48 RR-----EKNILEYL-EWA-K----P-FT-SKV--K-QMRLHRED----F     77
                     :|     |: :::.: : | |     | .| | |  | ..|..| |    :
HSL1_YEAST        36 KRASGHLER-VVQSVND-ATKRLSQPDSTVS-VATKSSKRKSR-DTVGPW     81

MRCKA_HUMAN       78 EIL-KVIGRGAFGEVAVVKL-KNAD--KVFAMKIL---------------    108
                     : | |.:|:|:|:.|.   |:| ||.:  ::.|:||:
HSL1_YEAST        82 K-LGKTLGKGSSGR---VRLAKNMETGQLAAIKIVPKKKAFVHCSNNGTV    127

MRCKA_HUMAN      109 -NKW--EML-KRAET---ACFRE------------ERDVLVNGDNKWITT    139
                      |.:  .|: ....:   |. ||             ||::::   .|.|:
HSL1_YEAST       128 PNSYSSSMVTSNVSSPSIAS-REHSNHSQTNPYGIEREIVI---MKLIS-    172

MRCKA_HUMAN      140 LH---YA-FQ--DDN--NLYLVMDYYV-GGDLLT-LLSKFEDRLPE-DMA    178
                      |    .| |:   |  .||||:: || ||:|.. |:|| .:||| : |
HSL1_YEAST       173 -HTNVMALFEVW-ENKSELYLVLE-YVDGGELFDYLVSK--GKLPERE-A    216

MRCKA_HUMAN      179 RFYLAEMVIAIDSVHQLH-Y-V-HRDIKPDNILMD-MNGHIRLADFG-SC    223
                     ..|..:  | ::.|...| : : |||:|:||:|:|| .|..|::|||| :.
HSL1_YEAST       217 IHYFKQ--I-VEGVSYCHSFNICHRDLKPENLLLDKKNRRIKIADFGMAA    263

MRCKA_HUMAN      224 L----KLMEDGTVQSSVAVGTPDYISPEILQAMEDGKGR-Y--GPECDWW    266
                     |     ||::  |    |   .|:|.|.||||:  |     || |  ||. |.|
HSL1_YEAST       264 LELPNKLLK--T---S--CGSPHYASPEIV--M----GRPYHGGPS-DVW    299

MRCKA_HUMAN      267 SLGVCMYEMLYGETPFYAESLVETYGKIMNHK-E--RFQFPAQVTDVSEN    313
                     |.|:.::.:|.|..||.:: ::   |:: .| :  ::|.|:.::  ||
HSL1_YEAST       300 SCGIVLFALLTGHLPFNDDN-IK---KLL-LKVQSGKYQMPSNLS--SE-    341

MRCKA_HUMAN      314 AKDLIRR-LICSREHRL-GQNGIEDFKKHPFFSGI-DWDNIRNCEAPYIP    360
                     |:|||.: |:...|.|: .|   | ..|||.   | .:|::      | :.
HSL1_YEAST       342 ARDLISKILVIDPEKRITTQ---E-ILKHPL---IKKYDDL-----P-VN    378

MRCKA_HUMAN      361 EV--SSPTDT-----SNFDVDDDCLKN-SETMPP-PT-HTAFSGHHLPFV    400
                     :|  ....|.       ||.|:  ..|.| |   |. .| |:  .|.    :
HSL1_YEAST       379 KVLRKMRKDNMARGKSNSDL--HLLNNVS---PSIVTLHS--KGE----I    417

MRCKA_HUMAN      401 GFTYTSSCVLSDRSCLRVT-AGPTSLDLDVN---VQRTL--DN---NLAT    441
                         ..| :|  || |::. .| .|.:| :.   :|:.:  :.   :|..
HSL1_YEAST       418 ----DES-IL--RS-LQILWHG-VSREL-ITAKLLQKPMSEEKLFYSLLL    457

MRCKA_HUMAN      442 EAYERR--IKRLEQEKLELSRKLQESTQ-TV-Q-ALQY-S-TVD--GPLT    482
```

```
                      : |::|  |. |.... | ::|  .:|: :| : .::| | |.:  | |
HSL1_YEAST       458 Q-YKQRHSIS-LSSSS-E-NKK--SATESSVNEPRIEYASKTANNTG-L-    499

MRCKA_HUMAN      483 ASKDLEIKNLKEEIEKLRKQVTESSHLEQQLEEANA---VRQELDDAFRQ    529
                     .|::.::|.| ..:| :..:| |: :.|.    ||    |..|: :|  .
HSL1_YEAST       500 RSENNDVKTL-HSLE-IHSEDT-ST-VNQN----NAITGVNTEI-NA--P    538

MRCKA_HUMAN      530 IKAYEK-Q--IKTLQQ-EREDLNK---E---L-----V-QA-SERL-KNQ    561
                     :.| :| |  |.||.| | .|  |    |    |      : .| |.|: :|
HSL1_YEAST       539 VLA-QKSQFSINTLSQPE-SD--KAEAEAVTLPPAIPIFNASSSRIFRN-    583

MRCKA_HUMAN      562 S-KELKDAHCQRKLAMQEFMEINERLTEL--HTQKQKLARHVRDKEEEVD    608
                     | ..: .:. .|: :::  :. |.||: |  .|.::.:  |  |  .|:.
HSL1_YEAST       584 SYTSI-SSR-SRR-SLR--LS-NSRLS-LSASTSRETV--H--D--NEMP    620

MRCKA_HUMAN      609 LVMQ--KVESLRQELRRTERAKKELEVHTEALAAEASKD-RK-L-REQ-S    652
                     | .|  |..| |..|.|  ||      :|     |:.::|. .| | |:. :
HSL1_YEAST       621 L-PQLPKSPS-RYSLSR--RA-----IH----ASPSTKSIHKSLSRKNIA    657

MRCKA_HUMAN      653 EHY-SKQ-LENELEGLKQKQISYSPGVCSIEHQQEITK--LK-TDLEKKS    697
                     ... ::: |:|...  | :.: |     |:   |.|:|  |. .||    .
HSL1_YEAST       658 ATVAARRTLQNSAS--K-RSL-Y-----SL---QSISKRSLNLNDL---L    692

MRCKA_HUMAN      698 IFYEEEL-SKREGIHANE-IKNLKKELH--DSEGQ-QLAL-NKEIMI---    738
                     :| ::.| ||:.   |:| : | |.|.|  :|:.. :: | : :|:.
HSL1_YEAST       693 VF-DDPLPSKKP---ASENV-N-KSEPHSLESDSDFEI-LCD-QILFGNA    734

MRCKA_HUMAN      739 LKDK-LEKTRRESQSEREEFESEFKQQYEREKVLLTE---EN-KKLTSEL    783
                     | |: ||         | || ::| |   ||:     |:    :| .|  |..
HSL1_YEAST       735 L-DRILE--------E-EE-DNE-K---ERD----TQRQRQNDTK--SSA    763

MRCKA_HUMAN      784 DKLT-TLYENLSIHNQQLEEEVKDLADKKESVAHWEAQITEIIQWVSDEK    832
                     |  | |: ..:|. |:  |.|     .  .|     :.   |:|       ||
HSL1_YEAST       764 D--TFTI-SGVST-NK--ENE----G--PE----YP---TKI------EK    788

MRCKA_HUMAN      833 DARGYLQALAS-KMTEELEALRNSSLGTRATDMPWKMRRFAKLDMSARLE    881
                     : : : . : | |.:|       |.| |  .:..|  :  |.| : :.
HSL1_YEAST       789 N-Q-F-N-M-SYKPSE------NMS-G--LSSFP--I--FEK-E-NT---    815

MRCKA_HUMAN      882 LQSA-LDAEIRAKQ-AIQEEL-NKVKAS-NIITECKL-K-DS---EKKNL    922
                     |.|: |: |.:.|: |: .:: |     | |      |: | :.   ||| :
HSL1_YEAST       816 LSSSYLE-EQKPKRAAL-SDITN----SFN-----KMNKQEGMRIEKK-I    853

MRCKA_HUMAN      923 ELLSEIEQLIKDTEELR-SE-KGIEHQDSQHSFLAFLNT-PTDALDQFER    969
                     :    . |||.|..:  | |. |.|:||:      |. :|: | :  || .:
HSL1_YEAST       854 Q---R-EQLQKKND--RPSPLKPIQHQE-----LR-VNSLP-N--DQ-GK    887

MRCKA_HUMAN      970 SPSCT--PASKGRRTVDSTPL-S-VHT--PTLR-KKGCPGS--T---G--   1005
                      ||.:  |     ||.: |.|: | |.:  ..|: ||. |.|  |    |
HSL1_YEAST       888 -PSLSLDP----RRNI-SQPVNSKVESLLQGLKFKKE-PASHWTHERGSL    930

MRCKA_HUMAN     1006 FPPKRKTHQFFVKSFTTPTKCHQCTSLMVGLIRQGCSCEVCGFSCHI--T   1053
                     |    ...|    |:. ..|.|     .|    . :     |.|     |.:: |
HSL1_YEAST       931 F---MSEH---VED-EKPVK----AS---D-V----SIE----SSYVPLT    957

MRCKA_HUMAN     1054 CVNKAPTTC--P-VPPE-QT--KGP-LGIDPQKGIGTA--Y---------   1085
                     .|  | |:.  | |..| .|  | | |.: |...:.|:  :
HSL1_YEAST       958 TV--A-TSSRDPSVLAESSTIQK-PMLSL-PSSFLNTSMTFKNLSQILAD   1002

MRCKA_HUMAN     1086 EG---HVRIP----K------PAGVKKGWQRA-LAIV--CDFKLFLYDIA   1119
                     :|   |:.:|     :        |  ::|  |.| :::.  .:....| .:.
HSL1_YEAST      1003 DGDDKHLSVPQNQSRSVAMSHP--LRK--QSAKISLTPRSNLNANL-SVK   1047

MRCKA_HUMAN     1120 --EGKASQP-SVVISQVIDMRDEEFSVSSVLASDVIHASRKDIPC-IFRV   1165
                       :|  | | | .:|.  |: |       .:  ||:..|  .:||. .|
HSL1_YEAST      1048 RNQG--S-PGS-YLSN--DL-D------GI--SDMTFA--MEIPTNTF--   1078

MRCKA_HUMAN     1166 TA-S-QLSASNNKCSILMLADTENEK-N---KWVGVLS-ELHKILK----   1204
                     || : ||  .||         ||:|.| |    | ....: |  |::|
HSL1_YEAST      1079 TAQAIQL--MNN--------DTDNNKINTSPK-ASSFTKE--KVIKSAAY   1115

MRCKA_HUMAN     1205 --KNKFR-DRS-V-YVPKEAYDSTLPLIKTTQAAAI-IDHERIALGNEEG   1248
                       |.| . |.| . |:| : |  |:|  .|....|| | .|. | .::||
HSL1_YEAST      1116 ISKEK-EPDNSDTNYIP-D-Y--TIP--NTYDEKAINI-FED-A-PSDEG   1155
```

```
MRCKA_HUMAN     1249 -LFVVHVTKDEIIRVGDNK-KIHQ--IELIPNDQLVA---VIS-GRN-RH   1289
                      |   :.:..|    .|:: .:|:  :. |  |.: |   |:: ..| |
HSL1_YEAST      1156 SL---NTSSSE----SDSRASVHRKAVS-I--DTM-ATTNVLTPATNVR-   1193

MRCKA_HUMAN     1290 VRLF--PMSALDG--RET--DFY-K--LS-ETKGCQTVTSGKVRHGALTC   1329
                     |.|:  ..|:  |  |||  :.. |  || |... .|       |
HSL1_YEAST      1194 VSLYWNNNSS--GIPRETTEEILSKLRLSPENPS-NT-------H-----   1228

MRCKA_HUMAN     1330 LCVAM-KRQVLCYELFQSKTR-HRKFKEIQVPYNVQWMAIFSEQLCVGFQ   1377
                         | ||       | |.|| .|   : .   |.  :.| |:.|    |
HSL1_YEAST      1229 ----MQKR-------F-SSTRGSR---D-S---NA--LGI-SQSL----Q   1252

MRCKA_HUMAN     1378 SGFLRYPLNGEGNPYSMLHSND-HTLSFIAHQPMDAICAVEISSKEYLLC   1426
                     |.| : .|  |       ...| || |    | .| |  :| ||    :
HSL1_YEAST      1253 SMF-K-DL--E-------EDQDGHT-S----Q-AD-I--LE-SS----M-   1276

MRCKA_HUMAN     1427 FNSIGIYTDCQGRRSRQQELMWPANPSSCCYNAPYLSVYSENAVD-IFDV   1475
                       |   |:    :| |      |:. .|  .| |     .:. |. :||
HSL1_YEAST      1277 --S---YS----KR-R------PSE-ES--VN-P-----KQR-VTMLFD-   1299

MRCKA_HUMAN     1476 NSMEWIQTLPLKKVRPLNNEGSLNLLG-L--E-TIRLIYFKNKMA-EGDE   1520
                     ...|  ::   |||      |.    | :  | | :|   .||:: |..:
HSL1_YEAST      1300 EEEE--ES---KKV------GG----GKIKEEHT-KL---DNKISEESSQ   1330

MRCKA_HUMAN     1521 LVVP--ETSDNSRKQMVRNINN-KRRYSFRVPEEERMQQRREMLRDPEMR   1567
                     ||:|  |     :|:   |.|| :..|| ::|:...:    ::.:|..|
HSL1_YEAST      1331 LVLPVVE-----KKE---NANNTENNYS-KIPKPSTI----KVTKDTAM-   1366

MRCKA_HUMAN     1568 NKLISNPTNFNHIAHMGPGDGIQILKDLPMN----PRP-QESRT--V--F   1608
                      :  || |. .| ... |     |||.: .|    ..| .:.:.  |  |
HSL1_YEAST      1367 -E--SN-TQ-TH-TKK-P-----ILKSV-QNVEVEEAPSSDKKNWFVKLF   1403

MRCKA_HUMAN     1609 SG-SVSIPSITK-SRPEPGRSMS---AS--SGLSA--RSSA--Q--NGSA   1645
                     .. | |..:.|| |:... .::|   |.  : |:.  ::|.  |  |
HSL1_YEAST      1404 QNFS-SHNNATKASKNHV-TNISFDDAHMLT-LNEFNKNSIDYQLKN---   1447

MRCKA_HUMAN     1646 L--K--R---E----FSGGSYSAK-RQPMPS-PSEGSLSSGGMDQGSDAP   1682
                     |  |  |   |    |..|::..| :  :.| |:..|:.:  :.:
HSL1_YEAST      1448 LDHKFGRKVVEYDCKFVKGNFKFKIK--ITSTPNASSVIT--VKK-----   1488

MRCKA_HUMAN     1683 ARDFDGEDSDSPRHSTASN-SSNLSSPPSPASPRKTKSLSLESTDR--G-   1728
                      |        | :|   || |||      .|. .|... .:|...|  |
HSL1_YEAST      1489 -R--------S-KH---SNTSSN------KAF-EKFND-DVERVIRNAGR   1517

MRCKA_HUMAN     1729 S   1729
                     |
HSL1_YEAST      1518 S   1518

--------------------------------------------
Name 1: SNW1_HUMAN
Name 2: PRP45_YEAST

SNW domain-containing protein 1 OS=Homo sapiens GN=SNW1 PE=1 SV=1
Pre-mRNA-processing protein 45 OS=Saccharomyces cerevisiae (strain ATCC 204508 /
 S288c) GN=PRP45 PE=1 SV=2

Needed for following interaction(s):
yeast   physical        SNW1_HUMAN      BLM_HUMAN       SNW1    BLM     PRP45_YE
AST     SGS1_YEAST

Length sequence 1:      536
Length sequence 2:      379
Sequence #1: SNW1_HUMAN
Sequence #2: PRP45_YEAST
Length #1: 517
Length #2: 517
Matrix: BLOSUM62
Gap open: 2.0
Gap extend: 1.0
Length: 517
Identity: 165/517 (31.91%)
Similarity: 244/517 (47.20%)
Gaps: 177/517 (34.24%)
```

```
Score: 665.00

SNW1_HUMAN        34 LVSSRREPPPYGYRKGWIPRLLEDFGDGGAFPEIHVAQYPLDMGRKKKMS     83
                     :.|:|. |||    |                   | :|     ||   :|
PRP45_YEAST        1 MFSNRL-PPP----K-------------------H-SQ-----GR---VS     17

SNW1_HUMAN        84 NALAIQVDSEGKIKYDAIARQGQ-SKDKVIYSKYTDLVPKEVMNAD-D-P    130
                     .||:    |: ::: .||... | :|: |   |..|.:||...|.: . |
PRP45_YEAST       18 TALS----SD-RVE-PAILTD-QIAKN-V---KLDDFIPKRQSNFELSVP     56

SNW1_HUMAN       131 DLQRPDE-EAIKEITEKTRVALEKS-VSQKVAAAMPVRAADKLA----PA    174
                      |  |.: | |:|.|.:|     || : |:    : |.|  |||    .|
PRP45_YEAST       57 -L--PTKAE-IQECTART-----KSYI-QR----L-VNA--KLANSNNRA     89

SNW1_HUMAN       175 QYIRY-TPSQQGVA---FNSGAKQRVIRMVEMQKDPMEPPRFKINKKIPR    220
                     . .|| |.:.|..|   .|:  ... |.:|..|.||: .||| :.|| .|
PRP45_YEAST       90 S-SRYVTETHQAPANLLLNN--SHH-IEVVSKQMDPL-LPRF-VGKK-AR    132

SNW1_HUMAN       221 ---GP-PSPP-APVMH---SPSR-KMTVKEQQEWKIPPCISNWKNAKGYT    261
                        .| .:.. .||:|   |..| :   .:..|||||..:|||||..|||
PRP45_YEAST      133 KVVAPTENDEVVPVLHMDGSNDRGE---ADPNEWKIPAAVSNWKNPNGYT    179

SNW1_HUMAN       262 IPLDKRL--AADGRGLQTVHINENFAKLAEALYIADRKAREAVEMRAQVE    309
                     :.|::|:  |.|... .|  ||:.|.||:|||..||:|||:  |:|:::|
PRP45_YEAST      180 VALERRVGKALDNEN-NT--INDGFMKLSEALENADKKARQ--EIRSKME    224

SNW1_HUMAN       310 -RKMAQKEKE---KHEEKLREMAQKARERRAGIKTHVEKEDGEARERDEI    355
                      :::|. |:|   | |.||:|::|:||... |  |   .:.| |     |
PRP45_YEAST      225 LKRLAM-EQEMLAK-ESKLKELSQRARYHN-G--T---PQTG-A-----I    260

SNW1_HUMAN       356 RHDRRKERQHDRNLSRAAPDKRSK-LQRNENRDISE-VIALGVPNPRTSN    403
                       .:.| :|    .|..|   |.| |..::.||:|| :| ||... |:..
PRP45_YEAST      261 --VKPK-KQ----TSTVA---RLKELAYSQGRDVSEKII-LGAAK-RSEQ    298

SNW1_HUMAN       404 -EVQYDQRLFNQSKGMDSGFA-GGEDEIY-N-VYDQAWRGGKDMAQSIYR    449
                      ::|||.|.|  ::|.::. | ..||::| | ::.|     :|: :|||:
PRP45_YEAST      299 PDLQYDSRFF--TRGANAS-AKRHEDQVYDNPLFVQ-----QDI-ESIYK    339

SNW1_HUMAN       450 PSKNLDKDMYGDDLEARIKTNRFVPDK-EFSGSDRRQRGREGPVQF---E    495
                      : |.:| :   | || :  |  |  | |  |:.    |..||:||   |
PRP45_YEAST      340 -T-NYEK-L---D-EA-V--N--V--KSE--GAS----GSHGPIQFTKAE    369

SNW1_HUMAN       496 EDPFGLDKFLEEAKQHG    512
                     .|    ||  .:  .:|
PRP45_YEAST      370 SD----DK--SD--NYG    378

--------------------------------------------
Name 1: PTN13_HUMAN
Name 2: Q8I120_CAEEL

Tyrosine-protein phosphatase non-receptor type 13 OS=Homo sapiens GN=PTPN13 PE=1
 SV=2
Protein C09D8.1c, confirmed by transcript evidence OS=Caenorhabditis elegans GN=
ptp-3 PE=1 SV=1

Needed for following interaction(s):
worm    genetic RASK_HUMAN      PTN13_HUMAN     KRAS    PTPN13  LET60_CAEEL
Q8I120_CAEEL

Length sequence 1:      2485
Length sequence 2:      1216
Sequence #1: PTN13_HUMAN
Sequence #2: Q8I120_CAEEL
Length #1: 1899
Length #2: 1899
Matrix: BLOSUM62
Gap open: 2.0
Gap extend: 1.0
Length: 1899
Identity: 475/1899 (25.01%)
Similarity: 716/1899 (37.70%)
Gaps: 855/1899 (45.02%)
Score: 1502.00
```

```
PTN13_HUMAN      735 MEKLDLSYIKEE-LPKLHNTYVGASE-KETELEFLKVCQRLT-E-YGVHF    780
                     ||: |      || |  |...:..:|| :||    ..| :. | : :|
Q8I120_CAEEL       1 MEE-D-----EEAL--LLKEFESSSEVEET----TTV-EP-TSDPFG---     33

PTN13_HUMAN      781 HRVHPEKKSQTGILLGVCSK-GVLVFEVHNGVRTLVLRFPWRETKKISFS    829
                     ..:       | | |  . | | |:    ::| :..| |       .|:: |
Q8I120_CAEEL      34 DSI-----S-T-I--S-C-KYGI----INN-LEAL-L-------NKVA-S     58

PTN13_HUMAN      830 --KKKITLQNTSDGIKHG-FQTDN--SKICQYLLH---LCSYQHKFQLQM    871
                        |..::  || |.:|.. |:.||  |:||.   |   |..  ....:::
Q8I120_CAEEL      59 GWKYHVS--NT-DNVKDAWFRLDNATSRICG---HIGALVG--PPTNVRV    100

PTN13_HUMAN      872 RARQSNQDA--Q-DIERASFRSLNLQ-AES--VRGFNMGRAISTGSLASS    915
                     .| .||..|  | |      |.|    | |:|  |: : | .  ..|
Q8I120_CAEEL     101 EA-TSNSTAVVQWD-----FES---QKADSFVVK-Y-M-H--EPG-----    131

PTN13_HUMAN      916 TLNKLAVRPLSVQAEILK-RLSCSELSLYQPLQNSSKEKNDKASWEEKPR    964
                       |    |   :..|  | :    :|    |:. |.    ||    |.|:
Q8I120_CAEEL     132 --N----R---MDTE--KWK----QL----PVV-SI----DK----ENPK    153

PTN13_HUMAN      965 EMS-KSYHDLSQASLY-PHR------KNVIVNME-P---PPQTVAELVGK   1002
                     ..: .|  ||: |  : |:.       ||    |.: |    || ||.|.| .
Q8I120_CAEEL     154 RFAVVS--DLN-A--HKPYAFCVLAVKN---NRQGPCSDPP-TVLESV-T    193

PTN13_HUMAN     1003 PSHQMSRSDAESLAGVT-KLNNSKSVASL----NRSPERRKHESDSSSIE   1047
                     |:: |     .::|. |. |.:|  || .|     | .|  |        ::
Q8I120_CAEEL     194 PTY-M----VQNLR-VLWKTSN--SV-QLTWEYN-GP--R-------NV-    223

PTN13_HUMAN     1048 DPGQAYVLGMTMHSSGNSSSQVPLKEN-DVLHKRWSIVSSP-------E-   1088
                       | .||   . | :|. ...|     | : |.:: : :|:|        |
Q8I120_CAEEL     224 --G-FYV---N-H-TGR-KDYV----NHE-LQEK-T-MSTPGFGQDVDEK    257

PTN13_HUMAN     1089 -RE-I-TLVNLKKDAKYGL--GFQII--GGEK-MGRLDLGIFISSVAPGG   1130
                       || : |  ||:....|.:  |.:.:  |..| ..: :: :.|:.  |.|
Q8I120_CAEEL     258 HREYLWT--NLRPHMMYTIHVGVRTLPPGARKYWPQ-EV-VTITD--PTG    301

PTN13_HUMAN     1131 P--AD---L-D--GCLKPGDRLISVNSVSLE-G-VSHHAAIEIL--QN-A   1167
                     |  .|   | |  | .:||.::|.:...:.| | :||: .| ||  .| :
Q8I120_CAEEL     302 PPFVDVPKLVDSSG-TQPGQQMIRLTPATEEYGPISHY-WI-ILVPANYS    348

PTN13_HUMAN     1168 PEDVTLVISQPKEKISKVPSTPVHLTNEMKNYMKKSSYMQDSAIDSSSKD   1217
                     .|||  |       .:.      |:.|  |       |:       :| :   |
Q8I120_CAEEL     349 TEDV--V------NLD-----PIEL--E------KA-----TA-E---K-    367

PTN13_HUMAN     1218 HHWSRGTL-RHISENSFGPSGGLREGSLSSQDSRTESASLSQSQVNGFFA   1266
                         |..| |.:|. |  ||    ::  |     .|  .|     |:|
Q8I120_CAEEL     368 ----RAQLARSLSV-S--PS---KK--L----KR--KA----SEV-----    390

PTN13_HUMAN     1267 SHLGDQTWQE-SQHGSPSPSVISKATEKETFTDSNQSKTKKPGISDVTDY   1315
                        ||.: |. |.|     |     |  ||.     :.:: | .|| :.||
Q8I120_CAEEL     391 ---GDDS-QSASYH----P----K--EKR----ARRA-T-VPG-AYVT--    417

PTN13_HUMAN     1316 SDRGDSDMDEATYSSSQDHQTPKQESSSSVNTSNKMNFKTFSSSPPKPGD   1365
                     : |     :         |: | :. ||:     ..  |  |         :|
Q8I120_CAEEL     418 A-R----L------SA-D-RV-KQQ----YR--N--N---------QP--    434

PTN13_HUMAN     1366 IFEVELAKNDNSL--GISVTGGVNTSVRHGGIYVKAVIPQGAAESDGRIH   1413
                      | : :  .|:.|  |.  |    |         |     |    .|     |
Q8I120_CAEEL     435 -F-I-V--GDSQLYDGF--T---N--------Y-----P---LE-----H    453

PTN13_HUMAN     1414 KGDRVLAVNGVSLEGATHKQAVETLRNTGQVVHLLLEKGQSPTSKEHVPV   1463
                             |   |     |      .|        |::    ::  .:|..|.
Q8I120_CAEEL     454 --------N---L----H------YR-------LMM---RA-FAKNDVR-    470

PTN13_HUMAN     1464 TPQCTLS-DQNAQGQGP--EKVKKTTQVKDYSFVTEENTFEVKLFKNSSG   1510
                     |..    | :|.|     |  ||:.:  ...| |.:||  .|.:|       |.
Q8I120_CAEEL     471 TKD---SFEQRA----PMSEKLSR--MYSD-SVLTE--PFTIK-----SA    503

PTN13_HUMAN     1511 L-GFSFSREDNLIPEQINASIV-RVKKLFPGQPAAESGKIDVGDVI---L   1555
                     |  | : |:.:  | .:.|.|. .| ||          |  | ||.:|    |
Q8I120_CAEEL     504 LRG-A-SQKSS--P-WVGACIAFLV--LF-------S--I-VGMLICWWL    536

PTN13_HUMAN     1556 KVNGASLKGLSQQEVISALRGTAPEVFLLLCRPPPGVLPEIDTALLTPLQ   1605
                     :.|    |      :  ||  |..|        |  .|.:.::  | ||
```

```
Q8I120_CAEEL     537 RCN----K-----K--SA--GRHP-------R--HGSITKV--A-LT---    558

PTN13_HUMAN     1606 SPAQVLPNSSKDSSQPSCVEQSTS---SDENEMSDKSKKQCKSPSRRDSY   1652
                       ..:: |..  ...|.  |  ||    |..||..     :|..:|      |
Q8I120_CAEEL     559 --GNIM-NGG--GGIPG--E--TSKLLSTSNEYG----RQIMNP-----Y    590

PTN13_HUMAN     1653 SDSSGSGEDDLVTAPANISNSTWSSALHQTLSNMVSQAQSHHEAPKSQED   1702
                             | .:      | .|        |                |.|:  |. |
Q8I120_CAEEL     591 -------E-QM-----N-GN-------H-----------HMES--SM-D    604

PTN13_HUMAN     1703 TICTMFYYPQKIPNKPEFEDSNP-SPLPPDMA-PGQSYQPQSESASSSSM   1750
                           :  ||  :|.  ....||. :|:|  :| |  |. |    : :.::|
Q8I120_CAEEL     605 ----L--YP--LPT--SHSRSNGYAPVP--VAIP--SL-P---N-NGNNM    635

PTN13_HUMAN     1751 DKYHIHHISEPTRQENWTPLKNDLENHLEDFELEVELLITLIKSEKG--S   1798
                     ..  :.|   |.      .|:. :|.||:|  .|.         :.:..|  |
Q8I120_CAEEL     636 TT--VSH---PA-----VPIA-ELANHIE--RLR-------MNNNAGFQS    665

PTN13_HUMAN     1799 LGF-TV-TKGNQRIGCYVHDVIQDPAKSDGRL-KPGDRLIKVNDTDVTNM   1845
                      .| :: | | |.. .:.|       :.:|  : |    .  |        |
Q8I120_CAEEL     666 -EFESIET-G-QHF-TWEH------SSAD--MNK---H--K-------N-    690

PTN13_HUMAN     1846 THTDAVNLLRAA-SKTVRLVIGRVLE-LPRIPMLPHLLPDITLTCNKEEL   1893
                     .:   | |:  || ..: |:|:..| | .|  .|       |. :..|
Q8I120_CAEEL     691 RY--A-NV--AAYDHS-RVVLSNV-EGYP--GM------DY-INAN----    720

PTN13_HUMAN     1894 GFSLCGGHDSLYQVVYISDINPRS-VAAIEGNL-QLL-DVIHYVNGVSTQ   1940
                      : :     |.      |  | .||| :|. :|.| :.. |     :    .:
Q8I120_CAEEL     721 -Y-V----DG-----Y--D-KPRSYIAT-QGPLPETFSD---F-----WR    747

PTN13_HUMAN     1941 GMTLEEVNRALDMSLPSLVLKATRNDLPVVPSSKRSAVSA----PKSTKG   1986
                      |..||       .|: ::|: .| | |      .:||.|..     | | :|
Q8I120_CAEEL     748 -MVWEE------QSV-TIVM-LT-N-L-----EERSRVKCDQYWP-S-RG    779

PTN13_HUMAN     1987 NGSYSVGSCSQPALTPNDSFS-TVAGEEINEIS-YPKGKCSTYQIK--GS   2032
                     ..:|  |           | .. |:. |.:: :: |.. :  |.::|  |.
Q8I120_CAEEL     780 TATY--G----------D-IEVTLL-ESVH-LAHYTM-R--TMRLKMVGE    811

PTN13_HUMAN     2033 PNLTLPKE-SYIQEDDIY----D----DSQEAE-VIQSLLDVVDEEAQNL   2072
                     |.:   :| .::|     |      |    | .... :| .|     :.:.:.
Q8I120_CAEEL     812 PEV---REIKHLQ----YTAWPDHGVPD-HPTPFLI--FL----KRVKT-    846

PTN13_HUMAN     2073 LNENNAAG--YS-CGPGTLKMNGKLSEERTEDT----DCDGSPLPE---Y   2112
                     ||.|: ||  .| |..| :   |      || ..     ||    .| |   |
Q8I120_CAEEL     847 LNPND-AGPIISHCSAG-I---G-----RT-GAFIVIDC---ML-ERLRY    881

PTN13_HUMAN     2113 FTEATKMNGCEEY-CEEKVKSE-S-LIQKPQEKK--TDD---DEITWGND   2154
                        :     |..:.| |...:::: | ::| .:|:.  ..|    |.:..|:.
Q8I120_CAEEL     882 --D----NTVDIYGCVTALRAQRSYMVQ-TEEQYIFIHDAVLDAVNSGST    924

PTN13_HUMAN     2155 ELPIERTNHEDSDKDHSFLTNDELAVLPVVKVLPSGKYTGAN-LKSVIRV   2203
                     |:|..|. |:       |   | :     :|.  :  ||      |: | |
Q8I120_CAEEL     925 EVPASRL-HQ-----H--L-H----ILS--Q--PS-----ADQL-S----    947

PTN13_HUMAN     2204 LRGLLDQGIPSKELENLQELK-PLDQCLIGQTKENRRKNRYKNILPYDAT   2252
                        | :|  :   |..:|..|| ..::|.:.....||.|||..:.:.|||:.
Q8I120_CAEEL     948 --G-ID--M---EFRHLTTLKWTSNRCTVANLPVNRPKNRMLSAVPYDSN    989

PTN13_HUMAN     2253 RV-----PLG-DEGG-YINASFIKIPVG-KEEFVYIACQGPLPT--TVGD   2292
                     ||      | | | |. |||||:|.    | ||...|||.|.  ||  |..|
Q8I120_CAEEL     990 RVIMRLLP-GAD-GSDYINASWID---GYKERGAYIATQA--PTNETAAD   1032

PTN13_HUMAN     2293 FWQMIWEQKSTVIAMM--TQEVEGEKIKCQRYWPNILGKT-TMVSNRLRL   2339
                     ||:.|||..|.:|||:  |.| .|::: :|..|||  | :| ..|.  : |
Q8I120_CAEEL    1033 FWRAIWEHNSPIIAMLVRTNE-RGQE-QCSDYWP--L-ETGVQVG--M-L   1074

PTN13_HUMAN     2340 ALVR-M-Q-QLKGFVVRAMTLEDIQTREVRHI-S-HLNFTAWPD----HD   2380
                      :|. | : .:|.:.:|...:.||.|||||.: . |  |..|||     |
Q8I120_CAEEL    1075 -VVEPMAEYDMKHYHLREFRISDINTREVRTVRQFH--FMEWPDVGKPH-   1120

PTN13_HUMAN     2381 TPSQPDDLLTFISYMRH-IH-R---SGPIIT-HCSAGIGRSGTLICIDVV   2424
                     | :  |..|.|::.: | .: :    :|| || ||.:|.||:...|.:.::
Q8I120_CAEEL    1121 T-A--DHFLDFVTQV-HNTYAQFGCTGP-ITVHCCSGAGRTAVFIALSII   1165
```

```
PTN13_HUMAN     2425 LG-LISQD-LD-FDISDLVRCMRLQRHGMVQTE-DQYIFCYQVILY-VL   2468
                     |. :.::. :| |  :  .|::|.:|..|:| | :||.|.| :..| .|
Q8I120_CAEEL    1166 LDRMRAEHVVDVF--T-TVKLLRTERQNMIQ-EPEQYHFLY-LAAYEYL   1209

--------------------------------------------
Name 1: GSTCD_HUMAN
Name 2: OMS1_YEAST

Glutathione S-transferase C-terminal domain-containing protein OS=Homo sapiens G
N=GSTCD PE=1 SV=2
Methyltransferase OMS1, mitochondrial OS=Saccharomyces cerevisiae (strain ATCC 2
04508 / S288c) GN=OMS1 PE=1 SV=1

Needed for following interaction(s):
yeast   genetic GSTCD_HUMAN     RASK_HUMAN      GSTCD   KRAS    OMS1_YEAST
IRA2_YEAST

Length sequence 1:      633
Length sequence 2:      471
Sequence #1: GSTCD_HUMAN
Sequence #2: OMS1_YEAST
Length #1: 700
Length #2: 700
Matrix: BLOSUM62
Gap open: 2.0
Gap extend: 1.0
Length: 700
Identity: 171/700 (24.43%)
Similarity: 275/700 (39.29%)
Gaps: 327/700 (46.71%)
Score: 473.00

GSTCD_HUMAN       18 FSHQTEGCIFPLH-----TSVTLFLL-SYCDCKIFKICLVVTKEVS-R-D     59
                     |  .|  |:  ||     .|.:| || |  . :  : .|..|   | : :
OMS1_YEAST         7 F--PT--CL--LHHIRQPASRSL-LLES--Q-R--R-SLSFT---SYKYN     40

GSTCD_HUMAN       60 SSLLRDDLIQDVEIQIISRQELPPIVQNCCLPAVVERSDNFCRAGLAVVL    109
                     ||.: ||   |   :  |:::|    :|     |.:.:.|  |     |:
OMS1_YEAST        41 SSHI-DD---D---K--SKKKL----KN-----VFQMNSN--R-----VI     65

GSTCD_HUMAN      110 RHIIQKSYEADPLKKELLELLGFKKTCLKACAE--VSQW---TRLCE---    151
                     |.  ||:.| : |.||  .   |::. |:: ..  | :|   .| .|
OMS1_YEAST        66 RK--QKTKE-E-LAKE--R---FEEQ-LRS-PNRFV-RWGAIAR-SEKFS    102

GSTCD_HUMAN      152 --LT---IPLA--IENFL-------RE--SSDQPPTIPVEILQ-LEKKLS    184
                       :|   |. |  |  ||       ::  :.|:      | |: |.|| .
OMS1_YEAST       103 KGMTKYMIG-AYVI--FLIYGLFFTKKLFAKDK------E-LERLLKK-Q    141

GSTCD_HUMAN      185 EPVRVHND-DKLR-RQKLK-Q-QKADGVGPPLTK-GKAKSKVHTQETSEG    229
                     |.... |: :.|| :: || : ::.|    .| | .:.| |:  ||  ||
OMS1_YEAST       142 EEGNA-NEYEALRIKE-LKGKLRRRD----EL-KLEEYK-KM--QE--EG    179

GSTCD_HUMAN      230 LDSSSKSLELKVA-F--SKLTVQEE--PA--TTN--REPSHIRKA----K    266
                     ::::..   :::|. |  :||  .|:  ||  |||  :|      ||    |
OMS1_YEAST       180 IENFD---DIRVQNFDQNKL--NEQILPARDTTNFYQE-----KANEYDK    219

GSTCD_HUMAN      267 ASDLPPLEHV-FAEG-----L--YFTLADIVL-LPC-----IHHFLVIIS    302
                     |.::.  |.| |. |      |  : ...| || :.|      | .:| .:|
OMS1_YEAST       220 AINME--ERVIFL-GKRRKWLMKH-CQGD-VLEVSCGTGRNI-KYL-DMS    262

GSTCD_HUMAN      303 R----KF---SEKLVEFPLLASWYQRIQE-VPGVKTAASKCGIQFLHLPK    344
                     |    .|   ||.::|   : : :::.:| .|  |  ..|  :.|: :.|
OMS1_YEAST       263 RINSITFLDSSENMME---I-T-HKKFREKFP--K--YKK--VAFV-VGK    300

GSTCD_HUMAN      345 LLTTSTEQHPNLCEVPGVEEQSDPLFIGGPRPTMAKLMEKGIE--VMFSP    392
                          .|   ||  | .:.|:        | :|::..  ||  |  |.:.
OMS1_YEAST       301 -----AE---NL--V-DLAEK-------G-KPSLEN--EK--ENQVKYD-    326

GSTCD_HUMAN      393 HPCPTWTLDWNVLPAAVSPKEGKM-S-SDRALRKQQQLNNLVYVVTNQ-A    439
                           |:   |  .|.    | : | .| .: |  .|||  :   .: .
OMS1_YEAST       327 ------TI---V--EAF----G-LCSHED-PV-K--ALNN--F---GKLL    351

GSTCD_HUMAN      440 KPGDRIVDFCSGGGHVGIVLAH-MLPSCQVTLIENKELSL-IRAKKRSDE    487
                     ||      |      |.: |:|.| .  . |...| || : | .||::|
```

```
OMS1_YEAST       352 KP-----D-----GRI-ILLEHGR--G-QYDFI-NK-I-LDNRAERR---    381

GSTCD_HUMAN      488 LGLSNIWFIQANMEYFTG-MFNIGVALHACG-VA--TDM-VIEHCIKTRA    532
                       | |.|          | .:|:.  |   | |.  :|: ::|.  | |
OMS1_YEAST       382 --L-NTW----------GCRWNLD--L---GEVLDDSDLELVEE--K-R-    409

GSTCD_HUMAN      533 SFV-TCPCCYGFIQNTSKFNFPKSE-QFKK-TLSYKEHMILCRFADQTAV    579
                     :.: | ..|   |  .:| .  |.: : || .|.:.|     ::. |:::
OMS1_YEAST       410 THLGT-TWC---I--VAK-R--KGDVK-KKDELGFVE-----KYL-QSSI    443

GSTCD_HUMAN      580 QLPPQRRL--IGKQCMCLVDLDRARAAEECGYSVQVISMEPESCSP--KN    625
                         ::|:  ..|:     | |.| :.:|         :||  ..|  |:
OMS1_YEAST       444 ----RKRMESFEKK-----D-DMA-SKKE---------LEP--VPPVSKS    471

--------------------------------------------
Name 1: HXD4_HUMAN
Name 2: LIN39_CAEEL

Homeobox protein Hox-D4 OS=Homo sapiens GN=HOXD4 PE=2 SV=3
Homeobox protein lin-39 OS=Caenorhabditis elegans GN=lin-39 PE=2 SV=1

Needed for following interaction(s):
worm    genetic RASK_HUMAN      HXD4_HUMAN      KRAS    HOXD4   LET60_CAEEL
LIN39_CAEEL

Length sequence 1:      255
Length sequence 2:      253
Sequence #1: HXD4_HUMAN
Sequence #2: LIN39_CAEEL
Length #1: 254
Length #2: 254
Matrix: BLOSUM62
Gap open: 2.0
Gap extend: 1.0
Length: 254
Identity: 98/254 (38.58%)
Similarity: 119/254 (46.85%)
Gaps: 60/254 (23.62%)
Score: 404.00

HXD4_HUMAN         1 MVMS-SYMVNSKYVD-PK-FPPCEEYLQGGYLGEQGADYYGG-GAQG---     43
                     |..| |  .:|  .| |: ..| |. ........:..... ||.|
LIN39_CAEEL        1 MTTSTS--PSS--TDAPRATAP-ES-SSSSSSSSSSSSTSSVGASGIPS     44

HXD4_HUMAN        44 -ADFQPP-GLY-P-RPD--FGEQPFGGS--GPGPGS--A--LPARGHGQE     81
                      ::.... | | | ...  .... | ||  .|...|  |  . |...|.
LIN39_CAEEL       45 SSELSSTIG-YDPMTASAALSAH-F-GSYYDPTSSSQIASYF-ASSQGL-     89

HXD4_HUMAN        82 PGGPGGHYAAP--GEP--C--PAPPAP-PP-APLPGARAYSQSDPKQPPS    123
                      |||  .|  |  |:.  |  |:..:. .. ..|.|.....:.|.|:..|
LIN39_CAEEL       90 -GGP--QY--PILGDQSLCYNPSVTSTHHDWKHLEGDDDDDKDDDKKGIS    134

HXD4_HUMAN       124 G--TAL-KQP--AVVYPWMKKVHVNSVNPNYTG---GEPKRSRTAYTRQQ    165
                     |  ..: |..  | |||||.:||  |.    ||    || ||.||||||.|
LIN39_CAEEL      135 GDDDDMDKNSGGA-VYPWMTRVH--ST----TGGSRGE-KRQRTAYTRNQ    176

HXD4_HUMAN       166 VLELEKEFHFNRYLTRRRRIEIAHTLCLSERQIKIWFQNRRMKWKKDHK-    214
                     ||||||||.::||||:||||:||:|.|:|||:||||||||||.||::|
LIN39_CAEEL      177 VLELEKEFHTHKYLTRKRRIEVAHSLMLTERQVKIWFQNRRMKHKKENKD    226

HXD4_HUMAN       215 LPNT    218
                     .|.|
LIN39_CAEEL      227 KPMT    230

--------------------------------------------
Name 1: RAD52_HUMAN
Name 2: RAD52_YEAST

DNA repair protein RAD52 homolog OS=Homo sapiens GN=RAD52 PE=1 SV=1
DNA repair and recombination protein RAD52 OS=Saccharomyces cerevisiae (strain A
TCC 204508 / S288c) GN=RAD52 PE=1 SV=2

Needed for following interaction(s):
yeast   genetic BLM_HUMAN       RAD52_HUMAN     BLM     RAD52   SGS1_YEAST
```

```
RAD52_YEAST

Length sequence 1:      418
Length sequence 2:      471
Sequence #1: RAD52_HUMAN
Sequence #2: RAD52_YEAST
Length #1: 540
Length #2: 540
Matrix: BLOSUM62
Gap open: 2.0
Gap extend: 1.0
Length: 540
Identity: 179/540 (33.15%)
Similarity: 256/540 (47.41%)
Gaps: 191/540 (35.37%)
Score: 704.00

RAD52_HUMAN        1 MSGTEEAILGGRD-SHPAAGGGSVLCFGQCQYTAEEYQAIQ-KALRQRLG     48
                     |:   | |: ..| ..|      |  ||  .: :|:   || | |.::||
RAD52_YEAST        1 MN---E-IM-DMDEKKP------V--FG--NH-SED---IQTK-LDKKLG     30

RAD52_HUMAN       49 PEYISSRMAG-GGQKVCYIEGHRVINLANEMFGYNGWAHSITQ-QNV--D     94
                     |||||.|: | |..::.||||.|||||||::||||||  | |: ::|  |
RAD52_YEAST       31 PEYISKRV-GFGTSRIAYIEGWRVINLANQIFGYNGW--S-TEVKSVVID     76

RAD52_HUMAN       95 FVDLNNGKFYVGVC-AFVRVQLKDGSYHEDVGYG-V-SEGLKSK-ALSLE    140
                     |:|...|||.:| | |.|||.|..|:|.||:||| | :|  :.| | :.|
RAD52_YEAST       77 FLDERQGKFSIG-CTAIVRVTLTSGTYREDIGYGTVENE--RRKPA-AFE    122

RAD52_HUMAN      141 KARKEAVTDGLKRALRSFGNALGNCILDKDYLRSLNKL----P----RQL    182
                     :|:|.||||.|||:||.||||||||:.|||:|..::|:    |    ..|
RAD52_YEAST      123 RAKKSAVTDALKRSLRGFGNALGNCLYDKDFLAKIDKVKFDPPDFDENNL    172

RAD52_HUMAN      183 --PLEVDLTKAKRQD-L-EPSVEEARYNSCRPNMALGHPQLQQV--TSP-    225
                       |.: :::::.|.: | | :.|:.:|    ||.   ..||.:|  |:|
RAD52_YEAST      173 FRPTD-EISESSRTNTLHE-NQEQQQY----PNK---RRQLTKVTNTNPD    213

RAD52_HUMAN      226 ------------SR--PSHAVIPADQDCSSRSLSSS--A-VES-EATHQ-    256
                                 ||  |..|. ||  :.:|:: ||:  . ::| :|:.|
RAD52_YEAST      214 STKNLVKIENTVSRGTPMMAA-PA--EANSKN-SSNKDTDLKSLDASKQD    259

RAD52_HUMAN      257 R-KLRQKQL--QQQFRE----RM----------EKQQVRV---S-T----    281
                     : .|....|  ...|::    .|          ||..| |   | |
RAD52_YEAST      260 QDDLLDDSLMFSDDFQDDDLINMGNTNSNVLTTEKDPV-VAKQSPTASSN    308

RAD52_HUMAN      282 PSAE-------K---S---------EAA--P--PAPPVTHSTPV--TVSE    306
                     |.||       |   |         |:.  |  .|..:.| | |  |.|:
RAD52_YEAST      309 PEAEQITFVTAKAATSVQNERYIGEESIFDPKYQAQSIRH-T-VDQTTSK    356

RAD52_HUMAN      307 --P--LL-EK------D-----FLA--GVTQELIKTLEDNSEKWAVTP--    336
                       |  :| :|      |     | |  |  ::|  ::: |::| .:.|
RAD52_YEAST      357 HIPASVLKDKTMTTARDSVYEKF-APKG--KQL--SMK-NNDK-ELGPHM    399

RAD52_HUMAN      337 -D-AGDGVVKPSSR-ADPAQTSDTLALNNQMVTQNR-TPHS-VCHQKPQA    381
                      : ||:.|  |  | ..|.:|:.| |.  ......| .|.| |.|  |..
RAD52_YEAST      400 LEGAGNQV--P--RETTPIKTNAT-AF--PPAAAPRFAPPSKVVH--PNG    440

RAD52_HUMAN      382 KSGSWDLQTYSA--DQRTTGNWESHR-KSQDMKKRKYDPS    418
                      :|:  :   .|  .||:|.. |..| |...:..||  |:
RAD52_YEAST      441 -NGA--V---PAVPQQRSTRR-EVGRPKINPLHARK--PT    471

--------------------------------------------
Name 1: IF4A1_HUMAN
Name 2: IF4A_YEAST

Eukaryotic initiation factor 4A-I OS=Homo sapiens GN=EIF4A1 PE=1 SV=1
ATP-dependent RNA helicase eIF4A OS=Saccharomyces cerevisiae (strain ATCC 204508
 / S288c) GN=TIF1 PE=1 SV=3

Needed for following interaction(s):
yeast   genetic PTEN_HUMAN      IF4A1_HUMAN     PTEN    EIF4A1  TEP1_YEAST
IF4A_YEAST

Length sequence 1:      406
Length sequence 2:      395
Sequence #1: IF4A1_HUMAN
Sequence #2: IF4A_YEAST
Length #1: 408
Length #2: 408
Matrix: BLOSUM62
Gap open: 2.0
Gap extend: 1.0
Length: 408
Identity: 269/408 (65.93%)
Similarity: 331/408 (81.13%)
Gaps: 25/408 (6.13%)
Score: 1369.00

IF4A1_HUMAN        9 SRDNG-PDGMEPEGVIESNWNEIVDSFDDMNLSESLLRGIYAYGFEKPSA     57
                     | : | .| :| |..|::|::::|..||||.|.|:||||::.||||:|||
IF4A_YEAST         2 S-E-GITD-IE-ESQIQTNYDKVVYKFDDMELDENLLRGVFGYGFEEPSA     47

IF4A1_HUMAN       58 IQQRAILPCIKGYDVIAQAQSGTGKTATFAISILQQIELDLKATQALVLA    107
                     ||||||:|.|:|:||:||||||||||.||:|:.||:|:..:||.|||:||
IF4A_YEAST        48 IQQRAIMPIIEGHDVLAQAQSGTGKTGTFSIAALQRIDTSVKAPQALMLA     97

IF4A1_HUMAN      108 PTRELAQQIQKVVMALGDYMGASCHACIGGTN-VR-AEVQKLQMEAPHII    155
                     ||||||.|||||||||..:|....|||||||: |. ||  .|: :| .|:
IF4A_YEAST        98 PTRELALQIQKVVMALAFHMDIKVHACIGGTSFVEDAE--GLR-DA-QIV    143

IF4A1_HUMAN      156 VGTPGRVFDMLN---RRYLSPKYIKMFVLDEADEMLSRGFKDQIYDIFQK    202
                     |||||||||  |   ||:.:.| ||||:|||||||||.|||:|||.||..
IF4A_YEAST       144 VGTPGRVFD--NIQRRRFRTDK-IKMFILDEADEMLSSGFKEQIYQIFTL    190

IF4A1_HUMAN      203 LNSNTQVVLLSATMPSDVLEVTKKFMRDPIRILVKKEELTLEGIRQFYIN    252
                     |...|||||||||||:||||||.||||:|:||||||:|||||||:|||:|
IF4A_YEAST       191 LPPTTQVVLLSATMPNDVLEVTTKFMRNPVRILVKKDELTLEGIKQFYVN    240

IF4A1_HUMAN      253 VEREEWKLDTLC--DLYETLTITQAVIFINTRRKVDWLTEKMHAR-D-FT    298
                     ||.||:|.:  |  |||:::::||||||.||||||:.||.|:  | | ||
IF4A_YEAST       241 VEEEEYKYE--CLTDLYDSISVTQAVIFCNTRRKVEELTTKL--RNDKFT    286

IF4A1_HUMAN      299 VSAMHGDMDQKERDVIMREFRSGSSRVLITTDLLARGIDVQQVSLVINYD    348
                     |||::.|:.|:|||.||:||||||||:||:||||||||||||||||||||
IF4A_YEAST       287 VSAIYSDLPQQERDTIMKEFRSGSSRILISTDLLARGIDVQQVSLVINYD    336

IF4A1_HUMAN      349 LPTNRENYIHRIGRGGRFGRKGVAINMVTEEDKRTLRDIETFYNTSIEEM    398
                     ||.|:|||||||||||||||||||||.||.||...:|::|.||:|.|||:
IF4A_YEAST       337 LPANKENYIHRIGRGGRFGRKGVAINFVTNEDVGAMRELEKFYSTQIEEL    386

IF4A1_HUMAN      399 PLNVADLI    406
                     |.::|.|:
IF4A_YEAST       387 PSDIATLL    394

--------------------------------------------
Name 1: FBX18_HUMAN
Name 2: SRS2_YEAST

F-box only protein 18 OS=Homo sapiens GN=FBXO18 PE=1 SV=2
ATP-dependent DNA helicase SRS2 OS=Saccharomyces cerevisiae (strain ATCC 204508
/ S288c) GN=SRS2 PE=1 SV=2

Needed for following interaction(s):
yeast   genetic BLM_HUMAN       FBX18_HUMAN     BLM     FBXO18  SGS1_YEAST
SRS2_YEAST
yeast   physical        BLM_HUMAN       FBX18_HUMAN     BLM     FBXO18  SGS1_YEA
ST      SRS2_YEAST

Length sequence 1:      1043
Length sequence 2:      1174
Sequence #1: FBX18_HUMAN
Sequence #2: SRS2_YEAST
Length #1: 1356
Length #2: 1356
Matrix: BLOSUM62
Gap open: 2.0
Gap extend: 1.0
Length: 1356
```

```
Identity: 348/1356 (25.66%)
Similarity: 510/1356 (37.61%)
Gaps: 619/1356 (45.65%)
Score: 967.00

FBX18_HUMAN       75 MAKSNS------VGQ-DSCQDSEGDMIFPAESSCALPQEGSAGPGSPGSA    117
                     |: ||:      |.| :: | .....:|  :.:..| |. .||||: |
SRS2_YEAST         1 MS-SNNDLWLHLVSQLNT-Q-QRAAALF--DYTRGL-QV-IAGPGT-G--     40

FBX18_HUMAN      118 PPSRKRSWSSEEESNQATGTSRWDGVSKKAPRHHLSVP----CTR-PREA    162
                         |         .:.. |||   |:.....||:. |    .|. ..:|
SRS2_YEAST        41 ----K---------TKVL-TSR---VAYLILHHHIH-PRDIIVTTFTNKA     72

FBX18_HUMAN      163 RQEAEDSTSRLSAE--SGETDQDAGDVGPDPIPDSYYGLLGTLPCQEALS    210
                     ..|.::   ||. |  .|     || |  : |  |.. |:||.
SRS2_YEAST        73 ANEMKE---RLQ-EMLRG-----AG-V--N-I--SEL-LIGTF-------     99

FBX18_HUMAN      211 H-ICSLPSEVL-RHVFAFLPVEDLYWNLSLVCHLWREIISDPLFIPWKKL    258
                     | || |  ::| |  |..| | ||  ...     || || |      :|
SRS2_YEAST       100 HSIC-L--KILYR--FGHL-V-DL--QKD-----WR-II-D------EK-    126

FBX18_HUMAN      259 YHRYLMNEEQAVSKVDGILSNCGIEKESDLCVLNLIR-YTAT-T-K---C    302
                             |     :|.||.:. |||..|    . || |.:: | |   |
SRS2_YEAST       127 --------E-----IDVILDDM-IEKVPD----Q-IRDYASSITRKVNLC    157

FBX18_HUMAN      303 SPSV--DPERVLWSLRDHP---------L-----LPEAEACV-RQHLPDL    335
                     .||.  | |   |::  ||         |     ||| |..: ..|  |
SRS2_YEAST       158 MPSKNGD-E---WTI--HPKLIKKQISKLKSNAILPE-EYILDSNH--D-    197

FBX18_HUMAN      336 YAAAGGVNIWALVAAVVLLSSSVN--DIQRLL---F-CL-R-RPSSTV--    375
                      ||.|  ..:.:..:.  ||.. |  |...||   | .| | |..|.:
SRS2_YEAST       198 -AALG--YFYQIYQSE--LSKK-NTLDFDDLLMYTFRLLTRVRVLSNIKH    241

FBX18_HUMAN      376 TMPD-VTET--LYCIAVL-LYAMREKGIN--ISNR---I----HYNIF-Y    411
                     .:.| ..:|  :. :.:: |:|   || |  :| |   |    ..:|: :
SRS2_YEAST       242 VLVDEFQDTNGIQ-LDLMFLFA---KG-NHHLS-RGMTIVGDPDQSIYAF    285

FBX18_HUMAN      412 --CL---YLQE--NSC-TQ-AT--KVKEE-PSVWPGKKTIQL-THE----    444
                       .|   :| |  ..| .: :|  .| |. .|   .:| | | |.|
SRS2_YEAST       286 RNALAHNFL-EMGRKCPIEYSTIILV-ENYRS---SQK-I-LNTSEILIT    328

FBX18_HUMAN      445 QQLILNHK-ME---PL--Q--V----VKIM---A-F--AGTGKTSTLVK-    475
                     ||   | | .:   ||  |  :    | .|   | |  |    .| ||:
SRS2_YEAST       329 QQ---N-KGRQNRAPLRAQFDLDFPPV-YMNFPAYFLEA----PS-LVRE    368

FBX18_HUMAN      476 --YAEKWSQSRFLYVTFNK-SI----AKQAERVFPSNVICKTFHSMAY--    516
                       |. | :... |: |||. :|    .:|.:|: .|.:| :  |.:.|
SRS2_YEAST       369 LLYL-K-ALPN-LF-TFNDFAILVRQRRQIKRI-ESALI-E--HRIPYKI    410

FBX18_HUMAN      517 --GHIGRKY-QSKK-K--LNLFKL--TP-----FMVNSVL--AEGKG---    548
                       ||   .: .||: :  |||.||  :|     .:. |:|  |.|.|
SRS2_YEAST       411 IRGH---SFWDSKETRAMLNLLKLIFSPNDKHAILA-SLLYPARGLGPAT    456

FBX18_HUMAN      549 G-FIR-A--KL---V-C-KTLENFFASADEELTIDHVPIWCKNSQGQR-V    588
                     | .|: |  .|   | | :.|::.  |: :::.:| :|  .|   | | |
SRS2_YEAST       457 GEKIKNALDTLATDVSCFQILKDI--SS-KKIMLD-IP--TK---G-RSV    496

FBX18_HUMAN      589 ------MVE------QSEKLNGV------L-EAS--R---LW-DNMRK--    611
                           |:|      ||..|.|:      | |.|  :   |: |..:|
SRS2_YEAST       497 IADFISMIENCQLLLQSTLLGGLSDLFDKLYELSGLKYEYLYKDGKKKND    546

FBX18_HUMAN      612 -LGECTE----EA-H---QMTHDGY-LKLWQLSKPSLASFD-----AI--    644
                      | |.:|    .| |   ::..: | |.|  ||| |.:| |     ||
SRS2_YEAST       547 QL-EKSEPNLLNARHKNIELLKN-YFLAL--LSK-SESS-DKEKNEAIKA    590

FBX18_HUMAN      645 FVDEAQDCTPAIMNIVLSQPCGK--I---F----V-GD--PHQQIYTFRG    682
                     ..|||:   | |.|.|:: |  |  :   |    : .|  ..::..:.:.
SRS2_YEAST       591 ATDEAE---P-IENKVIT-P--KEYLRNFFNSLSLHSDAAEEEESESNKD    633

FBX18_HUMAN      683 A-V----NALF-TVPHT-HVFYLTQSFRFGVE--IAYV-GAT--ILDVCK    720
                     | :    |. | |: .| |      ..: |:|  :.:: |..  |:. |
SRS2_YEAST       634 AKIKREKNG-FVTI-STIH------GAK-GLEWPVVFIPGCEEGIIP-C-    672

FBX18_HUMAN      721 RV-R--KK--TLVGGNH-QSGIRGDA--KGQVALLSRTNANVFDE-----    757
                      | .  ||  :...... |...:.||  | :..:|| ...:: ||
SRS2_YEAST       673 -VFNDDKKDESEEDEEEDQENSKKDASPK-KTRVLS-VEDSI-DEERRMF    718

FBX18_HUMAN      758 --A-VR----------VT-EG-EFP---SRIHL---IGGIKSFGLD--RI    784
                       | .|          || |. :.|   ||. |   :  ||:.. |  ::
SRS2_YEAST       719 FVAQTRAKYLLYLSNTVTVEDVDRPRIASRF-LTTDL--IKAMS-DSQKL    764

FBX18_HUMAN      785 ID----I---W-IL-LQP--EEERRKQ-NL-VI-KD--KFI--RR----W    812
                     .:    |   : || .:|  |::  |. :| .: ||  :||  ||    |
SRS2_YEAST       765 FESTNSIKKLYRILNKKPPAEDD--KLFSLDQLRKDYNQFIENRRERMIW    812

FBX18_HUMAN      813 --VH-KE--G--FS-----G-FKRYVTAAEDK-ELEAK--I----AVVEK    842
                       :. .:  |  .|     | ...: |:|.|: .||.:  |    .::||
SRS2_YEAST       813 QGIQMNDVYGIQLSRNKLLGSVSDF-TSAADQLRLETQNSIFPQKKLIEK    861

FBX18_HUMAN      843 ---------Y--NIRI--PELVQRI--E--KCH--I-EDLDFA-EYILGT    871
                              |  ..|:  ||  :|.  |  ..|  . :.: :| :|: .|
SRS2_YEAST       862 SRPSKINGNYAPKSRVKSPE--KRYAPETTSFHSPTKKKV-YAPQYV-ST    907

FBX18_HUMAN      872 --V------HKA--KGLEF----D-TVHVLDDFVKVP--CARHNLP-QLP    903
                       |      |.:  |.:.|    | ::   .| :. |  ..| :|. ..|
SRS2_YEAST       908 TNVPSRQEFHSSTGKNIPFLRREDRSI---TD-IS-PRSSTR-SLKGASP    951

FBX18_HUMAN      904 HFRVES-FSEDEWNLLYVAVTRAK-KRLIMTKSLENI--LTLAGEYFLQA    949
                     : :. | .|:|   |:..:.|| | |  : |:   ||  .| ||. | :.
SRS2_YEAST       952 N-KT-SHMSDD---LMRPSPTR-KDK--V-TR---NIHFAT-AGT-F-RI    986

FBX18_HUMAN      950 ELT-SNV--L-------K-----T-----GVVRCCVG--Q-------CNN    970
                     | | |||  |       |     |     |....|..  |       .||
SRS2_YEAST       987 E-TQSNVDELHPPEYSNKSGQSLTSSEFSGFSSACSNSDQPTNLIEDINN   1035

FBX18_HUMAN      971 AIPV-------D-TV------L----TMKKL-PITYSNRKENKGGYLCHS   1001
                     .:.:       | ::      |    | ||: |.| .|||..:|..:  .
SRS2_YEAST      1036 ELDLSDEELLNDISIERRRELLGSKKT-KKIKPKT-RNRKSKRGDKV--K   1081

FBX18_HUMAN     1002 CAEQRIGPL-A-F-------LTA-----SPEQVRAMERTVEN--IVLPRH   1035
                      .|:.| .| : |       .||     :|:     :.||:|  |:   .
SRS2_YEAST      1082 -VEEVI-DLKSEFEEDDSRNTTAAELLHNPD-----DTTVDNRPII---S   1121

FBX18_HUMAN     1036 EALLFL   1041
                     .| .||
SRS2_YEAST      1122 NA-KFL   1126

--------------------------------------------
Name 1: IF4E3_HUMAN
Name 2: IF4E_YEAST

Eukaryotic translation initiation factor 4E type 3 OS=Homo sapiens GN=EIF4E3 PE=
2 SV=4
Eukaryotic translation initiation factor 4E OS=Saccharomyces cerevisiae (strain
ATCC 204508 / S288c) GN=CDC33 PE=1 SV=1

Needed for following interaction(s):
yeast   physical        MUS81_HUMAN     IF4E3_HUMAN     MUS81   EIF4E3  MUS81_YE
AST     IF4E_YEAST

Length sequence 1:      224
Length sequence 2:      213
Sequence #1: IF4E3_HUMAN
Sequence #2: IF4E_YEAST
Length #1: 226
Length #2: 226
Matrix: BLOSUM62
Gap open: 2.0
Gap extend: 1.0
Length: 226
Identity: 78/226 (34.51%)
Similarity: 114/226 (50.44%)
Gaps: 67/226 (29.65%)
Score: 354.00

IF4E3_HUMAN       26 AAPEPPLGLQQLSA-LQPEPGGV--PLHSSWTFWLDRSLPGAT-AAECA-     70
                     |.|: .: |.. || .  :   |  ||::.||.|..:  | |. .:| :
IF4E_YEAST        21 ATPK-TV-LSD-SAHF--D---VKHPLNTKWTLWYTK--P-AVDKSE-SW     58
```

```
IF4E3_HUMAN          71 SN-LKKIYT-VQTVQIFWSVYNNIP-PVTSLPLRCS-YHLMRGERRPLWE    116
                        |: |:.: | .|||:.||::..||| | ..|||: | ||:.|.:.||.||
IF4E_YEAST           59 SDLLRPV-TSFQTVEEFWAIIQNIPEP-HELPLK-SDYHVFRNDVRPEWE    105

IF4E3_HUMAN         117 EESNAKGGVW--KMKVPK--DSTSTVWKEL--LLATIGEQFT-DCAAADD    159
                        :|:|||||.|  ::: .|  | ...:|  |  |||.|||  | |   .||
IF4E_YEAST          106 DEANAKGGKWSFQLR-GKGAD-IDELW--LRTLLAVIGE--TID---EDD    146

IF4E3_HUMAN         160 -EVIGVSV-SVRDR-EDVVQVW--NVNAS--L-V-GE-ATVLEKIYELLP    199
                         ::.|| | |:| : .:...:|  :.:..  | : |: ..|| |:.:  .
IF4E_YEAST          147 SQINGV-VLSIR-KGGNKFALWTKSEDKEPLLRIGGKFKQVL-KLTD--D    191

IF4E3_HUMAN         200 -HITFKAVFYKPHEEHHAFEGGRGKH    224
                         |:.|   |  ||..  |  .||  |
IF4E_YEAST          192 GHLEF---F--PHSS--A--NGR--H    206

--------------------------------------------
Name 1: CLK2_HUMAN
Name 2: KNS1_YEAST

Dual specificity protein kinase CLK2 OS=Homo sapiens GN=CLK2 PE=1 SV=1
Dual specificity protein kinase KNS1 OS=Saccharomyces cerevisiae (strain ATCC 20
4508 / S288c) GN=KNS1 PE=1 SV=2

Needed for following interaction(s):
yeast   genetic RASK_HUMAN      CLK2_HUMAN      KRAS    CLK2    IRA2_YEAST
KNS1_YEAST

Length sequence 1:      499
Length sequence 2:      737
Sequence #1: CLK2_HUMAN
Sequence #2: KNS1_YEAST
Length #1: 695
Length #2: 695
Matrix: BLOSUM62
Gap open: 2.0
Gap extend: 1.0
Length: 695
Identity: 208/695 (29.93%)
Similarity: 322/695 (46.33%)
Gaps: 237/695 (34.10%)
Score: 874.00

CLK2_HUMAN            2 P-HPRRY-H-------SS-----E-R--GS--RGSYREHYRSR--KH---     27
                        | .||:: |       ::     | |  .:  ..| ..|..||  :.
KNS1_YEAST           75 PLRPRQHQHQLFLDNENAIELDEEPRIINTTINNS-NNHNSSRVDEDADD    123

CLK2_HUMAN           28 -----KRR--R-SRS-----WSSSS----DR--TRRR------RREDS-Y     51
                             |.:  : | |      ||||    :.  |...      ...:| |
KNS1_YEAST          124 DIIFIKEQPIQFS-SPLILP-SSSSINNNNNIVTSNNPGCGTAATSNSTY    171

CLK2_HUMAN           52 HVRSRSSY-DDRS-S-DRRV-YDRRYCGSYRRNDY-S-RDRGDAYY----     91
                         :.:...: ..|: | . :: ..:  . ||:.| | : .|:.:|..
KNS1_YEAST          172 -ITTPKKFKKQRTISLP-QLPLSK--L-SYQSN-YFNVPDQTNAIVPRVT    215

CLK2_HUMAN           92 DTDYR--H---S----YEYQRE-N-----S-S-YRS----Q-RS---SRR    116
                        .|:..  |   |    .|..:. |     | | : |    | .|   |..
KNS1_YEAST          216 QTENELLHLTGSCAKTLEGNKAVNLTIAHSTSPF-SNPPAQIASLPQSNL    264

CLK2_HUMAN          117 KHRRRRRRS-RTF-SRSSSQH-SSRRAKS-VEDDAEGHLIYHVGDWL--Q    160
                        |  ::...| |.| |..||:. ||.  || .:.|.:||.:|...| :  .
KNS1_YEAST          265 K--KQIGSSLRKFTSNGSSESASSN--KSNFKTDKDGHYVYQEND-IFGS    309

CLK2_HUMAN          161 -ERYEIV-STLGEGTFGRVVQCVDHR-RGGARVALKIIKNVEKYKEAARL    207
                         .|: :| ..||:||||:|::|:|:: ... .||:|:|:.|::|:|||:.
KNS1_YEAST          310 GGRF-VVKDLLGQGTFGKVLKCIDNKYEPN-YVAVKVIRAVDRYREAAKT    357

CLK2_HUMAN          208 EINVLEKI-NEKDPDNK-NLCVQMF-DWFDYHGHMC-ISFELLGLSTFDF    253
                        |:.:|:.| | .||..: . |: :. :.|||..|:| :: :|.|.|.:||
KNS1_YEAST          358 ELRILQTILN-NDPQGQFQ-CL-LLRECFDYKNHICLVT-DLYGRSIYDF    403

CLK2_HUMAN          254 LKDNNYLP-YP-IHQVRHMAFQLCQAVKFLHD-NKLTHTDLKPENILFVN    300
                        :..|. :. :| .| ::.:|.||.::|.|||| . :.|||||||||| :
KNS1_YEAST          404 MCSNG-IARFPGSH-IQAIARQLIRSVCFLHDLG-IIHTDLKPENIL-I-    448

CLK2_HUMAN          301 SD--Y--E---L-T-YNLEKKRDE-----RSV-KSTAVRVVDFGSATFDH    335
                        .|  :  :   | | .:|.|:|.|     |.: |:..::::|||||.| |
KNS1_YEAST          449 CDETHIAQKLPLKTVQSLSKRRREASKGKRKILKNPEIKIIDFGSAIF-H    497

CLK2_HUMAN          336 -EHHSTIVSTRHYRAPEVILELGWSQPCDVWSIGCIIFEYYVGFTLFQTH    384
                         |:|..::|||||||||::|.||||.|||:|||.|::.|..:|.:|:..|
KNS1_YEAST          498 YEYHPPVISTRHYRAPEIVLGLGWSFPCDIWSIACVLVELVIGESLYPIH    547

CLK2_HUMAN          385 DNREHLAMMERILG-PIPS----RMIRKTR-K---------Q---KYFYR    416
                        :|.||:|||:||.| |.|:    :|..|:: |         .   |:|.|
KNS1_YEAST          548 ENLEHMAMMQRINGTPFPTDIIDKMFYKSKHKLGNSPSDLNSTVIKHFDR    597

CLK2_HUMAN          417 GR---LDWDE-N-------T--SAGRYVRENC--------KPLRR-Y---    441
                         :   |.|.| |       |  |..| |.::|        |.|:: |
KNS1_YEAST          598 -KTLSLQWPEKNKRGDTITTEKSMKR-VLQSCDRLDIYISKVLKQDYGDS    645

CLK2_HUMAN          442 LT---------------SE-A---EEH-------HQL----------F-D    454
                        |:               |: |   :.|       .:|          | |
KNS1_YEAST          646 LSINWNLPPEKNWSLINSKLAWKRQTHSSSSSTTDELDKETFLFWYWFID    695

CLK2_HUMAN          455 LIESMLEYEPAKRLTLGEALQHPFFARLRAEPPNKLWDSSRD-IS    498
                        |:..|.|::|.||:|..:||.|.:| .|     ..| |   | |:
KNS1_YEAST          696 LLRKMFEFDPTKRITAKDALDHEWF-NL-----GIL-D---DGIA    730

--------------------------------------------
Name 1: PTPA_HUMAN
Name 2: PTPA1_YEAST

Serine/threonine-protein phosphatase 2A activator OS=Homo sapiens GN=PPP2R4 PE=1
 SV=3
Serine/threonine-protein phosphatase 2A activator 1 OS=Saccharomyces cerevisiae
(strain ATCC 204508 / S288c) GN=RRD1 PE=1 SV=1

Needed for following interaction(s):
yeast   genetic PTPA_HUMAN      BLM_HUMAN       PPP2R4  BLM     PTPA1_YEAST
SGS1_YEAST

Length sequence 1:      358
Length sequence 2:      393
Sequence #1: PTPA_HUMAN
Sequence #2: PTPA1_YEAST
Length #1: 373
Length #2: 373
Matrix: BLOSUM62
Gap open: 2.0
Gap extend: 1.0
Length: 373
Identity: 150/373 (40.21%)
Similarity: 204/373 (54.69%)
Gaps: 80/373 (21.45%)
Score: 707.00

PTPA_HUMAN           12 SSEEA--PPATQNFIIPKKEIHTVPDMGKWKRSQAYADYIGFILTLNEGV     59
                        |.:..  |.||  |..|.|.|.   |      :|...|   |     :
PTPA1_YEAST           2 SLDRVDWPHAT--FSTPVKRIF---D------TQTTLD---F-----Q--     30

PTPA_HUMAN           60 KGKKLTFEYRVSEMWNEVHEEKEQAAKQSVS-C-DECIPLPRAGHCAPSE    107
                          ..|.. :|: : :: :|  | ...  .:| | |   |.|   | |.:.
PTPA1_YEAST          31 --SSLAI-HRI-K-YH-LH--K-YTT--LISHCSD---PDP---H-ATAS     62

PTPA_HUMAN          108 AIEKLV-ALLNTLDR---WIDETPPVDQPSRFGNKAYRTWYAKLDEEAEN    153
                        :| .:| .|:..||:   .||||||:..|.|:||.|.|.|:.||||....
PTPA1_YEAST          63 SI-AMVNGLMGVLDKLAHLIDETPPLPGPRRYGNLACREWHHKLDERLPQ    111

PTPA_HUMAN          154 -LVATVVPT--HLAAAVPEVAVYLKESVGNSTRIDYGTGHEAAFAAFLCC    200
                         | ..::|:  |  ..|||:..||..|.|:|||:|||||||.:|.|.:..
PTPA1_YEAST         112 WL-QEMLPSEYH--EVVPELQYYLGNSFGSSTRLDYGTGHELSFMATVAA    158

PTPA_HUMAN          201 LCKIGV---LR-VDDQIAIVFKVFNRYLEVMRKLQKTYRMEPAGSQGVWG    246
                        |..:|:   :| .|     ||.:||:|..:||:|..||.:|||||.||||
PTPA1_YEAST         159 LDMLGMFPHMRGAD-----VFLLFNKYYTIMRRLILTYTLEPAGSHGVWG    203
```

```
PTPA_HUMAN       247 LDD-FQFLPFIWGSS--QLID-H-PYLEPRHFVDEKA-VNENHKDYMFLE    290
                     ||| |. |.:|.|||  ||:| . | |:||..:| |: |.| :||..|.
PTPA1_YEAST      204 LDDHFH-LVYILGSSQWQLLDAQAP-LQPREILD-KSLVRE-YKDTNFY-    248

PTPA_HUMAN       291 C--ILFITEMKTGPFAEHSNQLWNIS-AVPSWSKVNQGLIRMYKAECLEK    337
                     |  |.||.|:|.|||.|||..|::|: .||.||||.:||::||..|.|:|
PTPA1_YEAST      249 CQGINFINEVKMGPFEEHSPILYDIAVTVPRWSKVCKGLLKMYSVEVLKK    298

PTPA_HUMAN       338 FPVIQHFKFGS-LLP-IHPVTSG    358
                     |||:|||.||: ..| :: :.:|
PTPA1_YEAST      299 FPVVQHFWFGTGFFPWVN-IQNG    320
```

Name 1: GEN_HUMAN
Name 2: RAD2_YEAST

Flap endonuclease GEN homolog 1 OS=Homo sapiens GN=GEN1 PE=1 SV=2
DNA repair protein RAD2 OS=Saccharomyces cerevisiae (strain ATCC 204508 / S288c) GN=RAD2 PE=1 SV=2

Needed for following interaction(s):
yeast genetic BLM_HUMAN GEN_HUMAN BLM GEN1 SGS1_YEAST RAD2_YEAST

Length sequence 1: 908
Length sequence 2: 1031
Sequence #1: GEN_HUMAN
Sequence #2: RAD2_YEAST
Length #1: 1204
Length #2: 1204
Matrix: BLOSUM62
Gap open: 2.0
Gap extend: 1.0
Length: 1204
Identity: 306/1204 (25.42%)
Similarity: 487/1204 (40.45%)
Gaps: 474/1204 (39.37%)
Score: 995.00

```
GEN_HUMAN          1 MGVNDLWQILEPVKQHIPLR--NLGGKTIAVDLSLWVCE-AQTVKKMMGS     47
                     |||:..|.|..|..:  |:|  :|..|.:|||.|:|:.: .:.|:...|:
RAD2_YEAST         1 MGVHSFWDIAGPTAR--PVRLESLEDKRMAVDASIWIYQFLKAVRDQEGN     48

GEN_HUMAN         48 VMK-PHLRNLFF-RISYLTQMDVKLVFVMEGEPPKLKADVISKRNQSRYG     95
                     .:| .|:.. || ||..|....::.|||.:|..|.||.:.|.:|.:.|.|
RAD2_YEAST        49 AVKNSHITG-FFRRICKLLYFGIRPVFVFDGGVPVLKRETIRQRKERRQG     97

GEN_HUMAN         96 --SSGKSWSQK-------TGRSHFKSVLRECLHMLECLG----I--P---    127
                       .|.||.::|       .| |: .:| :   :.....|    |  |
RAD2_YEAST        98 KRESAKSTARKLLALQLQNG-SN-DNV-K---NSTPSSGSSVQIFKPQDE    141

GEN_HUMAN        128 W-V-QAAG---EAE-A-M-C--AY--L-NA-GGHVDGCLTN-DGDT----    158
                     | : ...|   :.| | :  .  .:  | |: .|  || |.: |.||
RAD2_YEAST       142 WDLPDIPGFKYDKEDARVNSNKTFEKLMNSING--DG-LEDIDLDTINPA    188

GEN_HUMAN        159 ---F--LYGAQTVY------R---NFTMN-TKD------PH-VDCYT---    183
                        |  |..| |.|       |   ...|. :|:       |: :| ::
RAD2_YEAST       189 SAEFEELPKA-TQYLILSSLRLKSRLRMGYSKEQLETIFPNSMD-FSRFQ    236

GEN_HUMAN        184 MSSIK------SKL----GLDRDALVG---L--AIL--L-G--C-DY-LP    211
                     :..:|      .||    |. :|.  |   |  .::  : |  . :| |.
RAD2_YEAST       237 IDMVKRRNFFTQKLINTTGF-QDG--GASKLNEEVINRISGQKSKEYKLT    283

GEN_HUMAN        212 K---G-VPGVG-KE--QALKLIQIL--K--GQSLLQRF--N-------RW    241
                     |   | :.|:| .:  .|.|.| ::  |  | :|:::.  |       ||
RAD2_YEAST       284 KTNNGWILGLGANDGSDAQKAI-VIDDKDAG-ALVKQLDSNAEDGDVLRW    331

GEN_HUMAN        242 NETSCNSSPQL-LVTKKLAHCSVCSH-PGSPKDHER-NGCRLCKS-DKYC    287
                     ::...||   | :|  :  |.|  |: ..:|:  :| |  |    | |:.|
RAD2_YEAST       332 DDLEDNS---LKIV--R--HES--SNATTAPQ--KRSN--R---SEDEGC    365

GEN_HUMAN        288 EPHDYEYCCPCEWHRTEHDRQLSEVE---NNIK--K----KACCCEGFPF    328
                     : .| |    |||   |       |||   .|:|  :     ||  .. .|:
RAD2_YEAST       366 D-SD-E----CEW---E------EVELKPKNVKFVEDFSLKA--AR-LPY    397

GEN_HUMAN        329 -HEVI-----QEFLLNK--D-----K----L-V-KV-IR-----Y--QRP    351
                      .:.:      :.| |:|  |      |    : : :: :.      |  |..
RAD2_YEAST       398 MGQSLNNAGSKSF-LDKRHDQASPSKTTPTMRISRISVEDDDEDYLKQIE    446

GEN_HUMAN        352 DL-LLFQRFTLEKME-WPNHYACEKLLVL--LTHYDMIE-R----K---L    389
                     :: :: :...|.||| .|.  |.:|..:.  :|... .| |     :   |
RAD2_YEAST       447 EIEMM-EAVQLSKMEKKPE--ADDKSKIAKPVTSKG-TEARPPIVQYGLL    492

GEN_HUMAN        390 GSR-NSNQLQPIRI--V--KTR--I-RNG--VHCFEIEWEKPEHYAMEDK    429
                     |:: :| : ||..:  :  |:.  | |..  |  .. |:..|..  .|||
RAD2_YEAST       493 GAQPDS-K-QPYHVTNLNSKSESVIKRTSKTV--LS-EFRPPSQ--QEDK    535

GEN_HUMAN        430 QHGEFALLTIE-EESL-FEAAY--P------E--IVAVYQKQKLE--I--    463
                       |  |:|| | |::| | .::  |       |  :  ::|| ..|  :
RAD2_YEAST       536 --G--AILT-EGEQNLNF-ISHKIPQFDFNNENSL--LFQK-NTESNVSQ    576

GEN_HUMAN        464 KGKKQKRIKPKENNLPE-PDEVMS-FQSHMTLKPTCEIFHKQNSKLNSGI    511
                     :..|:|  .|    :|| |     | |.|  |...  ::::..|: .|. :
RAD2_YEAST       577 EATKEK--SP----IPEMP----SWFSS--TASQ--QLYNPYNT-TNF-V    610

GEN_HUMAN        512 SPDPTL--PQESISA-SLN---S--LL--LPKN-TPCLNAQ-EQFMSS--    547
                     . |..:  .||| .| :.|    |  ||  |  | |..|..: |: .||
RAD2_YEAST       611 E-DKNVRNEQES-GAETTNKGSSYELLTGL--NATEILERESEK-ESSND    655

GEN_HUMAN        548 ------LRPLAIQQI-KAV-SKSLISESSQPNTSSHNI-SVIADLH---L    585
                           |..|: ::: :.| :||.||:.::.| .|..: |:..: |   |
RAD2_YEAST       656 ENKDDDLEVLS-EELFEDVPTKSQISKEAEDN-DSRKVESINKE-HRKPL    702

GEN_HUMAN        586 STI-DWEGTSFS-----NSPAIQRNTFSHDLKSEVESELSAIPDGFENIP    629
                       | |::   ||      |  .:: | .   :| |.| |.    |.|:|
RAD2_YEAST       703 --IFDYD---FSEDEEDN--IVE-N-M---IK-EQE-EF----DTFKN--    732

GEN_HUMAN        630 EQLSCESERYTA-N--IK-KVLDE---DS-DGISPEEHLLSGIT-DLC--    668
                     ..||..:||..| |  :: ::.::   |. |  | :|     :| |:.
RAD2_YEAST       733 TTLSTSAERNVAENAFVEDELFEQQMKDKRD--S-DE-----VTMDMIKE    774

GEN_HUMAN        669 LQDLPLKERIFTKLSY---PQD-NLQ-PDVNLKTLSILSVKESCIANSGS    713
                     :|:| | .| | .:.|   |.: ..| .:: |: |: | | :..|.:. |
RAD2_YEAST       775 VQEL-L-SR-F-GIPYITAPMEAEAQCAEL-LQ-LN-L-V-DGIITDD-S    814

GEN_HUMAN        714 DC-----TSHLSKDLPGIPLQN--ESRDSK-ILK--G-D--------QLL    744
                     |.      |. :.|::  ...:|  |..|:: |||  | |          |||
RAD2_YEAST       815 DVFLFGGTK-IYKNM--FHEKNYVEFYDAESILKLLGLDRKNMIELAQLL    861

GEN_HUMAN        745 QEDYKVN--TSV-PYSVSN-TVV------KTCNVRP-PNTA-LDHSRKVD    782
                     ..|| .|  ..: |  ||: .|:       |  |.:. .|.. .| .||
RAD2_YEAST       862 GSDY-TNGLKGMGP--VSSIEVIAEFGNLK--NFKDWYNNGQFD-KRK--    903

GEN_HUMAN        783 MQTTRKI---LMKK----SVCLDRHSSDE-QSAPVFGKAKYTTQR--MKH    822
                     .:|..|.    |.||     .:.||     |: .|..|: .| |  .|  :|
RAD2_YEAST       904 QETENKFEKDLRKKLVNNEIILD----DDFPSVMVY-DA-Y--MRPEVDH    945

GEN_HUMAN        823 SSQKH-------N--SSHF-K-ESG--HNKLSS----PKIHIKETEQCVR    855
                     .:...        :  .| | | :.|  |.| |.    | : |::.:  |
RAD2_YEAST       946 DTTPFVWGVPDLDMLRS-FMKTQLGWPHEK-SDEILIP-L-IRDVNK--R    989

GEN_HUMAN        856 SYETAE---NEESCFPDSTKSSLSSLQCHKKENNS---GTCLDSPLPLRQ    899
                     ..:..:   ||  .||   :..:|.   .||.|.|   .|....   |::
RAD2_YEAST       990 KKKGKQKRINE--FFP---REYISG---DKKLNTSKRISTATGK---LKK   1028

GEN_HUMAN        900 RLKL    903
                     | |:
RAD2_YEAST      1029 R-KM   1031
```

Name 1: PFD6_HUMAN
Name 2: PFD6_YEAST

Prefoldin subunit 6 OS=Homo sapiens GN=PFDN6 PE=1 SV=1
Prefoldin subunit 6 OS=Saccharomyces cerevisiae (strain ATCC 204508 / S288c) GN=YKE2 PE=1 SV=1

Needed for following interaction(s):

```
yeast   genetic RASK_HUMAN      PFD6_HUMAN      KRAS    PFDN6   IRA2_YEAST
PFD6_YEAST

Length sequence 1:      129
Length sequence 2:      114
Sequence #1: PFD6_HUMAN
Sequence #2: PFD6_YEAST
Length #1: 136
Length #2: 136
Matrix: BLOSUM62
Gap open: 2.0
Gap extend: 1.0
Length: 136
Identity: 60/136 (44.12%)
Similarity: 76/136 (55.88%)
Gaps: 30/136 (22.06%)
Score: 249.00

PFD6_HUMAN         1 MAELIQKKLQGEVEKYQQLQKDLSKSMSGRQKLEAQLTENNIVKEELALL     50
                     |:||      |  .||||||.:|.:.:..|||||.||.||.||.||...|
PFD6_YEAST         1 MSEL------G--AKYQQLQNELEEFIVARQKLETQLQENKIVNEEFDQL     42

PFD6_HUMAN        51 DGSNV-VFKLLGPVL--VKQELGEARAT-VGKRLDYI-TAEIKRYESQLR     95
                     : .:. |:||.|.||  |:|  .||| | |.|||::| | ||.|.|..:|
PFD6_YEAST        43 E-EDTPVYKLTGNVLLPVEQ--SEAR-TNVDKRLEFIET-EITRCEKNIR     87

PFD6_HUMAN        96 DLERQSE-QQ-R-ETLAQLQQEFQRAQAAKAGAPGK    128
                     |  :|.| :: | | |.:|..     .||..| ||:
PFD6_YEAST        88 D--KQEELEKMRSE-LIKLNN-----TAASTG-PGR    114

--------------------------------------------
Name 1: AP1G1_HUMAN
Name 2: AP1G1_YEAST

AP−1 complex subunit gamma−1 OS=Homo sapiens GN=AP1G1 PE=1 SV=5
AP−1 complex subunit gamma−1 OS=Saccharomyces cerevisiae (strain ATCC 204508 / S
288c) GN=APL4 PE=1 SV=1

Needed for following interaction(s):
yeast   genetic AP1G1_HUMAN     BLM_HUMAN       AP1G1   BLM     AP1G1_YEAST
SGS1_YEAST

Length sequence 1:      822
Length sequence 2:      832
Sequence #1: AP1G1_HUMAN
Sequence #2: AP1G1_YEAST
Length #1: 939
Length #2: 939
Matrix: BLOSUM62
Gap open: 2.0
Gap extend: 1.0
Length: 939
Identity: 332/939 (35.36%)
Similarity: 500/939 (53.25%)
Gaps: 248/939 (26.41%)
Score: 1428.00

AP1G1_HUMAN        7 LRELIRTIRTARTQAEEREMIQKECAAIRSSFREEDN-TY--RCR-NVAK     52
                     ||..|:.:|.|:|.|:||.:|.|:.|.||:..| :|: .:  | | |:.|
AP1G1_YEAST        5 LRSFIKDVRGAKTLADERAIITKQSAKIRTKLR-DDHLPHEKR-RVNIQK     52

AP1G1_HUMAN       53 LLYMHMLGYPAHFGQLECLKLIASQKFTDKRIGYLGAMLLLDERQDVHL-    101
                     |||:::||...||||:|.:.||||..|.|||:|||.|.|||||.:|: |
AP1G1_YEAST       53 LLYLYILGEKTHFGQVESINLIASDDFVDKRLGYLAATLLLDESEDL-LT    101

AP1G1_HUMAN      102 LMTNCIKNDLNHSTQF-VQGLALCT-LGCMGSS-EMCRDLAGEVEKLLKT    148
                     |:||.:.|||:|..:: | .||| | ||.: || |:.|||..:||.::|
AP1G1_YEAST      102 LLTNMLNNDLHHPNKYAV-SLAL-TSLGFL-SSPELARDLYPDVENIIK-    147

AP1G1_HUMAN      149 SNS---YLRKKAAL-CAVHVIRK-VPELMEMF-L-PATKNLLNEKN---H    188
                      ||   :|.|| || ||..:|.| | .|:|:| : ..|| :|:..:   |
AP1G1_YEAST      148 -NSRDPFLLKK-ALQCAAKLIFKDV-SLLEIFNIEDITK-ILSSHSICTH    193

AP1G1_HUMAN      189 GVL--HT----SVVL--LT----EMCERSPDMLAHFRK--LVP-Q-LVR-    221
                     |||  .|    |::|  |.    |  :...|.: .:..  |.| . |:|
AP1G1_YEAST      194 GVLLGVTKIIQSILLIGLNRKKDE--DEDEDGI-DYSNDILSPLSLLLRD    240

AP1G1_HUMAN      222 --I-LKNLIM-S-GYSPEHDVSGISDPFLQ--VRI--LRL-LRILGR--N    259
                       | |:|  | | ...|.:||.||.|||||  : |  |:| .:: |.  |
AP1G1_YEAST      241 FFIRLEN--MNSKNIEPGYDVQGICDPFLQCEI-IYTLKLYFQV-GELLN    286

AP1G1_HUMAN      260 -DD--DSSEAMN--DILAQVATNTETSKNVGNAILYETVLTI--MDIKSE    302
                      ::  |..:  |  |:|.::||||:::||.|.|||||||.||  :|: ::
AP1G1_YEAST      287 SNNVLDYKD--NFCDLLTRIATNTDSTKNSGQAILYETVKTIFSLDL-NQ    333

AP1G1_HUMAN      303 SGLRVLAINILGRFLLNNDKNIRYVALTSLLKTV-QTDHNAVQRHRSTIV    351
                     . ||||.||||.:||...|.|.:||:|.:|||.| | :..||||||..|.
AP1G1_YEAST      334 P-LRVLGINILAKFLAGKDNNTKYVSLNTLLKVVPQ-EPTAVQRHRKFIS    381

AP1G1_HUMAN      352 DCLKDLDVSIKRRAMELSFALVNGNN-IRGMMKELLYFL---DSCEPEFK    397
                     .||:|.||||:.||:|||||:::.:| :. ::.||:.||   |  | :.|
AP1G1_YEAST      382 HCLQDTDVSIRMRALELSFAILDDSNLVE-LVNELMKFLAKQD--E-DSK    427

AP1G1_HUMAN      398 ADCASGIF----LAAEKYAPS---K--RWHIDTIMRVLTTAGSYVRDDAV    438
                      |..  |:    | .:.: .:   |  .|.:|....:|...||::..:.:
AP1G1_YEAST      428 -DLI--IYTIDHL-IDTF-DTRVVKDESWKLDVFFNILKLVGSFINYEKI    472

AP1G1_HUMAN      439 PN--LIQLITN-SVEM--HA-YTVQRLYKAI-L-G---DYSQQPLV--Q-    474
                      |  || :|.| | ::  .: : ::::. .| | |   :.|::. :  |
AP1G1_YEAST      473 -NDILI-IINNTS-QLSDKSEF-LRKML-TISLNGTSAEISEEN-IGWQL    516

AP1G1_HUMAN      475 VAAWCIGEYGDLLVS-GQCEE-EEPIQVTEDEVLD-IL---ESVL--ISN    516
                     |..|||||||||::: |. :. .: | :.|..:.| :|   |  |  .:|
AP1G1_YEAST      517 VLIWCIGEYGDLVLNEGN-KNGAD-I-INESSITDYLLTLQE--LYTATN    561

AP1G1_HUMAN      517 MSTSVTRGYALTAIMKLSTRF-TC-TVNRIKK-VVSIYGSSIDVELQQRA    563
                     :  .:. .|.|||.:|||.|| .. .:.:::: ::| |..|.|:.||.::
AP1G1_YEAST      562 L--KII-NYILTAALKLSVRFHDAKNIEKLRQLILS-YTDSTDLSLQMKS    607

AP1G1_HUMAN      564 VEYNALFKKYDH---MRSALLERMPVMEKVTTNGPTEIVQTNGETEPAPL    610
                     .:|. :|  ::.   ::..:||.||..||:|    .|  |.||:   | |
AP1G1_YEAST      608 NQYE-IF--FNQSISVKKIILETMPKFEKIT----EE--QDNGK---A-L    644

AP1G1_HUMAN      611 ETKPPPSGPQPTSQANDLL-DLLGGNDITPVIPTAPTSKPSSAGGEL--L    657
                      :|...|. :|.    ||| ||| |.| :    .| .|| :|.|..:  :
AP1G1_YEAST      645 -SKNLISN-EPV----DLLSDLL-GED-S----KA-ESK-ASTGDNVKPI    680

AP1G1_HUMAN      658 DLL----GDIN-LTGAPAAAPAPASVPQISQPPFLLDGLSS--QPLFNDI    700
                     |:|    |:.| :    |..|..|:..: |     ::. ||  :.  |
AP1G1_YEAST      681 DILEEIFGEKNDI----AQVPKNANKEE-S-----INH-SSAVEA--N--    715

AP1G1_HUMAN      701 AAGI--PSITAYSKNGLKIEFTFERSNTNPSV--TVITIQASNS--TELD    744
                      :|:  | :.|   |  ||   ::.|:.|  |  ::::  | ||  ..||
AP1G1_YEAST      716 -SGVTLP-LDA---N--KI---YDSSSLN--VYASLLS--A-NSGLAHLD    750

AP1G1_HUMAN      745 MTDFVFQA-----------AVPKTFQ-LQL--LSPSSSIVPAFNTGTI-T    779
                     :  : |||           ||||. | |.|  |.|||:|    |...| .
AP1G1_YEAST      751 L--Y-FQAKSLISDLKTFCAVPKA-QKLTLGQLYPSSTI----NASQICK    792

AP1G1_HUMAN      780 QVIKVLNPQKQQLRMRIKLTYNH-KGSAMQDLAEVN-NF    816
                     |.:|:....|  |::|:||.: | .||:  .:  .| .|
AP1G1_YEAST      793 QSLKISGSGK--LKLRVKLDF-HLNGSS--SI--TNEQF    824

--------------------------------------------
Name 1: RL35_HUMAN
Name 2: RL35_YEAST

60S ribosomal protein L35 OS=Homo sapiens GN=RPL35 PE=1 SV=2
60S ribosomal protein L35 OS=Saccharomyces cerevisiae (strain ATCC 204508 / S288
c) GN=RPL35A PE=1 SV=1

Needed for following interaction(s):
yeast   genetic RASK_HUMAN      RL35_HUMAN      KRAS    RPL35   IRA2_YEAST
RL35_YEAST

Length sequence 1:      123
Length sequence 2:      120
Sequence #1: RL35_HUMAN
```

```
Sequence #2: RL35_YEAST
Length #1: 125
Length #2: 125
Matrix: BLOSUM62
Gap open: 2.0
Gap extend: 1.0
Length: 125
Identity: 71/125 (56.80%)
Similarity: 93/125 (74.40%)
Gaps: 7/125 (5.60%)
Score: 339.00

RL35_HUMAN         1 MAKIKARDLRGKKKEELLKQLDDLKVELSQLRVAKVTGGAASKLSKIRVV     50
                     ||.:||.:||.|.||:|..||.|||.||::|:|.|::  ..| |.||:.|
RL35_YEAST         1 MAGVKAYELRTKSKEQLASQLVDLKKELAELKVQKLS--RPS-LPKIKTV     47

RL35_HUMAN        51 RKSIARVLTVINQTQKENLRKFYKGKKYKPLDLRPKKTRAMRRRLNKHE-     99
                     |||||.||||||:.|:|.:|:.||||||:|.|||.|||||:||.|.|.|
RL35_YEAST        48 RKSIACVLTVINEQQREAVRQLYKGKKYQPKDLRAKKTRALRRALTKFEA     97

RL35_HUMAN       100 ENLKTKKQQRKERL-YPLRKYAVKA    123
                     ..: |:| |||::: :|.||||:||
RL35_YEAST        98 SQV-TEK-QRKKQIAFPQRKYAIKA    120

--------------------------------------------
Name 1: DDR1_HUMAN
Name 2: Q95ZV7_CAEEL

Epithelial discoidin domain-containing receptor 1 OS=Homo sapiens GN=DDR1 PE=1 S
V=1
Discoidin domain receptor protein 2, isoform a, partially confirmed by transcrip
t evidence OS=Caenorhabditis elegans GN=ddr-2 PE=2 SV=1

Needed for following interaction(s):
worm    genetic RASK_HUMAN      DDR1_HUMAN      KRAS    DDR1    LET60_CAEEL
Q95ZV7_CAEEL

Length sequence 1:      913
Length sequence 2:      797
Sequence #1: DDR1_HUMAN
Sequence #2: Q95ZV7_CAEEL
Length #1: 982
Length #2: 982
Matrix: BLOSUM62
Gap open: 2.0
Gap extend: 1.0
Length: 982
Identity: 330/982 (33.60%)
Similarity: 478/982 (48.68%)
Gaps: 271/982 (27.60%)
Score: 1482.00

DDR1_HUMAN         9 LLLLLL-VA--SGD--A-DMKGHFDPAKCRYALGMQDRTIPDSDISASSS     52
                     |||.|. |.  |..  | :::      :|.:.|||.:|.|.|..||||||
Q95ZV7_CAEEL       3 LLLYLFGVTFHSNTVVALELR------ECSHQLGMSNRKIRDEQISASSS     46

DDR1_HUMAN        53 WSD--STAARHSRL--ESSDGDGAWCPAGSV--FPKEEEYLQ----VDLQ     92
                     : |  ||..:|:|.  ||  |.|||||...:  ..|  |:||    ||.
Q95ZV7_CAEEL      47 F-DLQSTGPQHARAHQES--GSGAWCPKNQINSLSK--EWLQISFSVDT-     90

DDR1_HUMAN        93 RLHLVALVGTQGRHAGGLGKEFSRSYRLRYSRDG-RRWMGWKDRWGQ-EV    140
                        ::..|.||||...|.|.|::.::::|.|.. ..|..:||.: : |.
Q95ZV7_CAEEL      91 ---VITSVETQGRFDDGRGMEYATAFKIQYWRPSLNAWASYKDDF-ELET    136

DDR1_HUMAN       141 ISGNEDPEGVVLKDLGPPMVARLVRFYP--RADRVMSVCLRVELYGCLWR    188
                     |..|.|.|..:.:.|...::||.:|..|  .:.|  :||:|||::||.:.
Q95ZV7_CAEEL     137 IPANNDTEHAIRRHLDRAIIARRIRIVPVSNSTR--TVCMRVEVFGCPFD    184

DDR1_HUMAN       189 DGLLSYTAPV--GQTMYLSEAV-YLND-STYDGHTVGG--LQYGGLGQLA    232
                     |.|:.|.  |  |.   |...: | :| | |||:....  |. ||:|:|.
Q95ZV7_CAEEL     185 DSLVFYN--VDQGD---LQSGISY-HDFS-YDGNLANSPHLT-GGIGKLY    226

DDR1_HUMAN       233 DGVVGLDD-FRKSQELRVWPGYDYVGWSNHSFSSGYVEMEFEFDRLRAFQ    281
                     ||.||.:: | .:.. : |     ||| ... .:|.|::.|||..||...
Q95ZV7_CAEEL     227 DGEVGKNNVF-VNHH-K-W-----VGW-RRK-RNGNVKLAFEFSELRNIS    266

DDR1_HUMAN       282 AMQVHCNNMHTLGAR-LPGG-VECRFR-RGPAMAWE-GEPMRH-NLGGNL    326
                      .:.:|.:|.....|: .... |  .|. .|.    : .:.:.| |   |.
Q95ZV7_CAEEL     267 GILIHTSNEFKKSAKAFSSATV--LFSINGK----DFSDTIVHFN---NP    307

DDR1_HUMAN       327 GD-----PR-ARAVSVPLGGRVARFLQCRFL-FAG---PWLLF-SEISFI    365
                     .|     || .|   :|:..|:|:..:.| | | |   .| || ||::|.
Q95ZV7_CAEEL     308 EDTESEVPRWIR---IPVNNRIAKVAKIR-LNF-GTDSDW-LFISEVNFE    351

DDR1_HUMAN       366 SDVVNNSS-PALGG--TFPPA-PWWPPGPPPTNFSSLELEPRGQQPVAKA    411
                     |   |::: ..|..  ..|.: .:          | |: .|   ..
Q95ZV7_CAEEL     352 S---NHTNIELLNDDVVIPDSVSY---------F-SV-TE---HD-----    379

DDR1_HUMAN       412 EGSPTAILIGCLVAIILLLLLIIA---L-MLWR-LHWR-RLLS---KAER    452
                     :|  |: :. ..: |...:.||:|   | :|:| ..:| :..|   .|:|
Q95ZV7_CAEEL     380 DG--TS-MF-AFI-IFFFMFLIVAVIILTVLYRKREYRVKASSPSPNAKR    424

DDR1_HUMAN       453 RVLEEELTVHLSVPGDTILINNRPGPREPPPYQEPRPRGNPPHSAPCVPN    502
                     .:|   ||    :.|:|  |.:..   .|..||  ..|.|       :.|
Q95ZV7_CAEEL     425 EIL---LT----IDGNT--IKHHV---SPSTYQ--MARDN-------LQN    453

DDR1_HUMAN       503 GSALL----LSNPAYRLLLATYARPPRGP-GPPTPAWAKPTNTQ-AYSG-    545
                       ||:    :| |    :::.||.|.... ...|   |   ||. .| |
Q95ZV7_CAEEL     454 --ALIEKMPMS-P----IISDYAEPDISVCSDVT---A---NTPLLY-GI    489

DDR1_HUMAN       546 D--YMEPEKPGAPLLPPPPQNS-VPHYAEADIVTLQGVTGGNTYAVPALP    592
                     |  | :.:|...||      :| | .|  :|.        |..|.. .||
Q95ZV7_CAEEL     490 DGPY-DTQKRSNPL------SSMV-KY--SDY--------GEVYCT-TLP    520

DDR1_HUMAN       593 PGAVGDGPPRVDFPRSRLRF-KEKLGEGQFGEVHLCEVDSPQDLVSLDFP    641
                       .:.    | |    :| . ..::|:|:||||.||      |    |:
Q95ZV7_CAEEL     521 --EIA----R-D----KL-ICVSRIGQGEFGEVDLC-----Q----LE--    547

DDR1_HUMAN       642 LNVRKGHPLLVAVKILRPDATKNARNDF-L-KEVKIM-SRLKDPNIIRLL    688
                      | ||     ||||.|.  ....| ::| . :|:::: | ||.||::.::
Q95ZV7_CAEEL     548 -N-RK-----VAVKKLH--GISQA-DEFSFHREIRVLGS-LKHPNVVEVV    586

DDR1_HUMAN       689 GVC-VQDDP-LCMITDYMENGDLNQFLSAHQLEDKAAEGAPGDGQAAQGP    736
                     ||| :| .| || |.:|||||||..::     |  |    . | ..|.:|
Q95ZV7_CAEEL     587 GVCTIQ-KPILC-IMEYMENGDLKSYI----L--K---N-P-TIQTSQ--    621

DDR1_HUMAN       737 TISYPMLLHVAAQIASGMRYLATLNFVHRDLATRNCLV-GE-NFTIKIAD    784
                     .||      :..|:|:|:.||.:.||||||:|.||||| || |  :||||
Q95ZV7_CAEEL     622 CIS------ICTQLAAGLAYLESCNFVHRDIAARNCLVDGEGN--VKIAD    663

DDR1_HUMAN       785 FGMSRNLYAGDYYRVQGRAVLPIRWMAWECILMGKFTTASDVWAFGVTLW    834
                     |||:|:||:.:||:|:|:.|||||||||.:|:|||:||||||.||||:|
Q95ZV7_CAEEL     664 FGMARSLYSQEYYKVEGKFVLPIRWMAWEALLLGKFSTASDVWGFGVTMW    713

DDR1_HUMAN       835 EVLMLCRAQPFGQLTDEQVIENAGEFFRDQG--RQVYLSRPPACPQGLY-    881
                     |:..||..:|:..:||:.|:||. :.....|  :|| ||||..||..||
Q95ZV7_CAEEL     714 EIFSLCSEKPYSDMTDDDVVENL-QSMSSTGSLKQV-LSRPRMCPSKLYN    761

DDR1_HUMAN       882 ELMLRCWSRESEQRPPFSQLHRFLAEDAL-NT    912
                     |.:|.||:.||.:||.|..:|..| : :| :|
Q95ZV7_CAEEL     762 EQILPCWNYESSRRPSFENVHLHL-Q-SLVHT    791

--------------------------------------------
Name 1: RAB9A_HUMAN
Name 2: YPT7_YEAST

Ras-related protein Rab-9A OS=Homo sapiens GN=RAB9A PE=1 SV=1
GTP-binding protein YPT7 OS=Saccharomyces cerevisiae (strain ATCC 204508 / S288c
) GN=YPT7 PE=1 SV=1

Needed for following interaction(s):
yeast   genetic PTTG1_HUMAN     RAB9A_HUMAN     PTTG1   RAB9A   SECU_YEAST
YPT7_YEAST

Length sequence 1:      201
Length sequence 2:      208
Sequence #1: RAB9A_HUMAN
Sequence #2: YPT7_YEAST
```

```
Length #1: 220
Length #2: 220
Matrix: BLOSUM62
Gap open: 2.0
Gap extend: 1.0
Length: 220
Identity: 111/220 (50.45%)
Similarity: 148/220 (67.27%)
Gaps: 31/220 (14.09%)
Score: 564.00

RAB9A_HUMAN         1 MAG-KSSLFKVILLGDGGVGKSSLMNRYVTN-KFDTQLFH-TIGVEFLNK     47
                      |:. |.::.|||:|||.||||:|||:||| | |: :|.:. |||.:||.|
YPT7_YEAST          1 MSSRKKNILKVIILGDSGVGKTSLMHRYV-NDKY-SQQYKATIGADFLTK     48

RAB9A_HUMAN        48 DLEVDGHFV-TMQIWDTAGQERFRSLRTPFYRGSDCCLLTFSVDDSQSFQ     96
                      ::.|||..| |||:|||||||||:||...||||:|||:|.:.|.::.||:
YPT7_YEAST         49 EVTVDGDKVATMQVWDTAGQERFQSLGVAFYRGADCCVLVYDVTNASSFE     98

RAB9A_HUMAN        97 NLSNWKKEFIYYADVKEPESFPFVILGNKIDISE-RQ-VSTEE-AQ--AW    141
                      |:.:|:.||:.:|:|..||:|||||||||||..| :: || |: ||  |
YPT7_YEAST         99 NIKSWRDEFLVHANVNSPETFPFVILGNKIDAEESKKIVS-EKSAQELA-    146

RAB9A_HUMAN       142 CRDNGDYPYFETSAKDATNVAAAFEE-AVRRVL----A-T---EDRSDHL    182
                       :..||.|.|.||||:|.||..|||| | |..|    | |   ||  |:
YPT7_YEAST        147 -KSLGDIPLFLTSAKNAINVDTAFEEIA-RSALQQNQADTEAFED--DY-    191

RAB9A_HUMAN       183 IQTDTVNLHRKPKPSSSC-C    201
                        .|.:|: |....::|| |
YPT7_YEAST        192 --NDAINI-RLDGENNSCSC    208

------------------------------------------
Name 1: IRS1_HUMAN
Name 2: IRS1_DROME

Insulin receptor substrate 1 OS=Homo sapiens GN=IRS1 PE=1 SV=1
Insulin receptor substrate 1 OS=Drosophila melanogaster GN=chico PE=1 SV=1

Needed for following interaction(s):
fly     genetic IRS1_HUMAN      PTEN_HUMAN      IRS1    PTEN    IRS1_DROME
Q7KMQ6_DROME

Length sequence 1:      1242
Length sequence 2:      968
Sequence #1: IRS1_HUMAN
Sequence #2: IRS1_DROME
Length #1: 1306
Length #2: 1306
Matrix: BLOSUM62
Gap open: 2.0
Gap extend: 1.0
Length: 1306
Identity: 371/1306 (28.41%)
Similarity: 554/1306 (42.42%)
Gaps: 497/1306 (38.06%)
Score: 1300.00

IRS1_HUMAN          1 MASPPESDGFSDVRKVGYLRKPKSMHKRFFVL--RAASEAGGPARLEYYE     48
                      |||..: ||.: : . |||:|.|:|.|:||||  ..::.|   ||||||:
IRS1_DROME          1 MASISD-DGMA-L-S-GYLKKLKTMKKKFFVLYEETSTSA---ARLEYYD     43

IRS1_HUMAN         49 NEKKWRHKSSAPKRSIPLESCFNINKRADSKNKHLVALYTRDEHFAIAAD     98
                      .|||:..::. |||.|.|::|||||:|.|:|::.::.|.:||..|.|..
IRS1_DROME         44 TEKKFLQRAE-PKRVIYLKNCFNINRRLDTKHRFVIVLSSRDGGFGIVL-     91

IRS1_HUMAN         99 SEAEQD--SWYQALLQLHNRAKGHHDGAAALGAGGGGGSCSGSSGLGEAG    146
                       |.|.|  .|...||.| .|     :    : |    .| :|:     |
IRS1_DROME         92 -ENENDLRKWLDKLLVL-QR-----N----I-A----NS-NGT-----A-    118

IRS1_HUMAN        147 EDLS-YGDVPPGPAFKEVWQVIL-KPKGLGQTKNLIGI---YRLCLTSKT    191
                      .  | | |        .||||:: | ||:.: |  :||   |..|||||:
IRS1_DROME        119 H--SPY-D--------HVWQVVIQK-KGISE-K--VGITGTYHCCLTSKS    153

IRS1_HUMAN        192 ISFV-----KL-NSE--AAAV-VLQLMNIRRCGH-S-ENFFFIEVGRSAV    230
                      ::||     |. |.|  .|:: :| |..|||||| | :..|::|:||.:|
IRS1_DROME        154 LTFVCIGPEKTPNGEDRVASIEIL-LTTIRRCGHASPQCIFYVELGRQSV    202

IRS1_HUMAN        231 TGPGEFWMQVDDSVVAQNMHETILEAMRAM--SD----E-F--RP-----    266
                      .|.|:.||:.|::.:|.|||.|||.||.|.  |:    . :  ||
IRS1_DROME        203 LGSGDLWMETDNAAIATNMHNTILSAMSAKTESNTNLINVYQNRPDLSHE    252

IRS1_HUMAN        267 --RSKSQSSSN-CSNPISVPLRRHHLNNPPPSQVGLTRRSRTESITATSP    313
                        |.:| ||:| .|.||:|.:    :.|   ||..|..||      .:||
IRS1_DROME        253 PMRKRS-SSANEASKPINVNV----IQN---SQNSLELRS------CSSP    288

IRS1_HUMAN        314 ASMVG-GKPGSFRVRASSDGEGTMSRPASVDGSPVSPSTNRTH-AHRH--    359
                      .: .| |     |.|.  |     |.|.. :|: :|.|:|:|: ...|
IRS1_DROME        289 HN-YGFG-----RERC--D-----SLPTR-NGT-LSESSNQTYFGSNHGL    323

IRS1_HUMAN        360 RG---SA-RLHPPLN-HSRSIP---MPASRCSPSATSPVSLSSSSTSGHG    401
                      |.   |. |.| ..| ||.| |   ||. |||.|..|.:|:..|  ..:|
IRS1_DROME        324 RSNTISGIRPH-STNKHSNS-PTFTMPL-RCSESEESSISVDES--DDNG    368

IRS1_HUMAN        402 STSDCLFPR---RSS-ASVSGSPSDGGFISSDEYGSSPCD-FRSSFRSVT    446
                      |.|.  : |   ||| .::   |.:.  |  |::.|:  : |  | : ||
IRS1_DROME        369 SFSH--Y-RLNTRSSETAI---PEEN--I--DDFASA--ELF--S-K-VT    402

IRS1_HUMAN        447 PDSLGHTPPARGEEELSNYICMGGKGPSTLTAP-NG-HYILSR-GGNGHR    493
                       :.  :.     .:|  |||.|   .|  :. | :. |   .: ..:..|
IRS1_DROME        403 -EQ--NV-----SDE--NYIPM---NP--VN-PTDAIH---EKEKADMQR    433

IRS1_HUMAN        494 CTPGTGLGTS-PALAGDEAASAADL--DNR-FRK-RTHSAGTSPTITHQK    538
                      ....: |..: |..|.::.|...||  ||: .|. |.:|.|..  :.|.|
IRS1_DROME        434 LEDAS-LHFNFPEHASEKLAKDFDLDSDNQCCRPIRAYSIGNK--VEHLK    480

IRS1_HUMAN        539 TPSQSSVASIEEYTEMMP----AYPPGGGSGGRLPGHRHSAFVPTRSYPE    584
                      . :: .:..:.: |...|    ||     |    : |.: |. :| |.
IRS1_DROME        481 F-NK-RLGHLND-TGQNPNRVRAY-----S---V-GSK-SK-IP-RC---    512

IRS1_HUMAN        585 EGLE-MHPL-E-RRGGH----HRPDSSTLHTDDG--Y------MPMSPGV    619
                      : |: : .| | .:  |    :|..|| : |.:|  |      ...|..
IRS1_DROME        513 D-LQRV-VLVEDNK--HEFTANRSQSS-I-TKEGTSYGSSANRQKKSTS-    555

IRS1_HUMAN        620 AP-------VPSGRKGSGDYMPM--SPKSVS-APQQII--NPIRRHPQRV    657
                      ||       :.|.|. | |.|.:  | ::.: ..|:.|  |.|   |:.:
IRS1_DROME        556 APLLSLKNQINSDRM-S-DLMEIDFS-QATNLEKQKFIKNNEI---PKYI    599

IRS1_HUMAN        658 DPNGYMMMSPSGGCSP--DIGGGPSSSSSSSNAVPSGTSYGK-LWTNGVG    704
                      : |   :. |.   :|  |      |||.:.:|    ||. | :: ||
IRS1_DROME        600 E-N---VF-PK---APRTD------SSSLTLHA----TSQ-KDIF-NG--    627

IRS1_HUMAN        705 GHHSHVLPHPKPPVESSGGKLLPCTG-D-YMNMSPVGDSN--TSSP-SDC    749
                         :      |  :.::.   :  |. | |:.|.|||  |  |  | |:|
IRS1_DROME        628 ---T------K--LNNTA---I--TSEDGYLEMKPVG--NGYT--PSSNC    657

IRS1_HUMAN        750 YYGP---EDPQHKPVLS-YYSLPRSFKHTQRPGEPEEGARHQHL-RLSTS    794
                      .  |   |  :.|  || |.:.| ..  | ....|   . |. | ::||
IRS1_DROME        658 L--PMKVE--KLK--LSDYQTAP-PL--T-ATAAP---V-HD-LNKIST-    691

IRS1_HUMAN        795 SGRLLYAATAD---D--S-SSSTSSDS-LGGG-YCGAR---LE-PSLPHP    832
                           |..:|:   :  | |....|:| |... : .::   :| .|..|.
IRS1_DROME        692 -----YNISAEKWREQPSRSEEKKSNSPLNDNTF-SSKPTNVESTSKSHD    735

IRS1_HUMAN        833 -H--HQV-LQPHLPRKVDTAAQTNSRLARPTRLSLGDPK-A--STLP-RA    874
                       |  :|: .:      ||  .||::.:| . ..|:  | | .  :.|. ..
IRS1_DROME        736 VHSANQIDCE-----KV--CAQSSDKL-N-NHLA--D-KIVENNNLDIGG    773

IRS1_HUMAN        875 REQQQQQQPLLHPPEPKS-PGE-Y--V----NIEFGS-DQSGY--LSGPV    913
                      .|:::    |:|     | ..| |  :    | :|.. :::||  |.  :
IRS1_DROME        774 HEEKK----LVH-----SISSEDYTQIKDKSN-DFTKFNEAGYKILQ--I    811

IRS1_HUMAN        914 AFHS-SPSVRCPSQL-QPAPREEETGTEEYMKMD-LGPGRRAAWQESTGV    960
                        .| | |: ..|:| |   :    |.  :  | | |   .|:  |..|
IRS1_DROME        812 --KSDS-SL-ISSKLYQ---K----GI--H-K-DNL---ERS--QRLT--    839

IRS1_HUMAN        961 E-MGRLGPAPPGAASICRPTRAVPSSRGDYMTMQMSCPRQSYVDTS-PAA   1008
                      | :..:   |..|.:    | || ||  .. :| :.:      :::: |||
IRS1_DROME        840 ESVNTI---PDNATA----T-AV-SS-SS-LT-KFN------INSAKPAA    871
```

```
IRS1_HUMAN      1009 PVSYADMR-TGI--AA-E---EV-SL--P-RATMAAASSSSAASASPTGP   1047
                       : ||.| ||.  :. :   :: .|  | |       |||..| .|
IRS1_DROME       872 --A-ADSRSTGTDPSTPQNILQIKDLNFPSR-------SSSRIS-QP---    907

IRS1_HUMAN      1048 QGAAELAAH-SSL-LGGPQGPGGMSAFTRVNLSPNRNQSAKVIRADPQGC   1095
                         ||  | :|| |  |.    .|.  : |  |      ||.::   :|.
IRS1_DROME       908 ----EL--HYASLDL--PH----CSG--Q-N--P-----AKYLK---RGS    932

IRS1_HUMAN      1096 RRRHSSETFSSTP-SA-TRVGNTVPFGAGAAVG-GGGGSSSSSEDVKRHS   1142
                     |     |   |.| || ...|||  :   |.:. ....|||||.::
IRS1_DROME       933 R-----E---SPPVSACPEDGNT--Y---AKIDFDQSDSSSSSSNI----    965

IRS1_HUMAN      1143 SASFEN   1148
                        | |
IRS1_DROME       966 ---F-N    967

--------------------------------------------
Name 1: PK3CB_HUMAN
Name 2: P91634_DROME

Phosphatidylinositol-4,5-bisphosphate 3-kinase catalytic subunit beta isoform OS
=Homo sapiens GN=PIK3CB PE=1 SV=1
Phosphoinositide 3-kinase, Dp110 OS=Drosophila melanogaster GN=Pi3K92E PE=1 SV=1

Needed for following interaction(s):
fly     physical        RASK_HUMAN      PK3CB_HUMAN     KRAS    PIK3CB  RAS1_DRO
ME      P91634_DROME

Length sequence 1:      1070
Length sequence 2:      1088
Sequence #1: PK3CB_HUMAN
Sequence #2: P91634_DROME
Length #1: 1187
Length #2: 1187
Matrix: BLOSUM62
Gap open: 2.0
Gap extend: 1.0
Length: 1187
Identity: 484/1187 (40.78%)
Similarity: 695/1187 (58.55%)
Gaps: 255/1187 (21.48%)
Score: 2354.00

PK3CB_HUMAN        8 PPAMA-DILDIWAVDSQIAS--DGSIPVDFLLPTGIYIQLEV-PREA-TI     52
                     ||.|. .: ::|  .:::.:  |  : : .|||.|..::|.| |  | ||
P91634_DROME      27 PPCMRFSV-NLW--KNEMLNWVD--L-I-CLLPNGFLLELRVNP--ANTI     67

PK3CB_HUMAN       53 SYIK-QMLWKQVHNYPMFNLLMDIDSYMF--ACVNQTAVY--E--ELE--     93
                     ..|| :|: .|....|     |     .|:.  ||..|  ||  .  .:|
P91634_DROME      68 QVIKVEMV-NQAKQMP----L----GYVIKEACEYQ--VYGISTFNIEPY    106

PK3CB_HUMAN       94 -DETRRLCDVRPFLPVLKL-----VTR-SCDPGEKLDSK-I-GVLIGKGL    134
                      |||:||.:|:|:..:|.|      .|. |.| .| | :| : | :||.
P91634_DROME     107 TDETKRLSEVQPYFGILSLGERTDTTSFSSD-YE-L-TKMVNG-MIGT--    150

PK3CB_HUMAN      135 HEFD-SLK---DPEVNEFRRKMRKF--S-E-EKILSLVGLSWMDWLKQ--    174
                      .|| : :   .||:::||..|.:.  : | |:  |    :: .| :|
P91634_DROME     151 -TFDHN-RTHGSPEIDDFRLYMTQTCDNIELER--S----AY-TW-QQRL    190

PK3CB_HUMAN      175 TYPPEHEP-----S--IPENL--E---DKLYGGKLIVAV-HFENCQDVFS    211
                     .|  || |     |  :|| |  |   .:.:   ||| | : ||.|..|:
P91634_DROME     191 LY--EH-PLRLANSTKMPE-LIRERHPTRTF---LIV-VKN-ENDQSTFT    231

PK3CB_HUMAN      212 FQVSPN-MN-PIKVNELAIQK--RLTIHGK-EDEVSPYDYVLQVSGRVEY    256
                     ..|  | .: |..:.|..:||  |..:  | .|..|  ||:|:||||.||
P91634_DROME     232 LSV--NEQDTPFSLTESTLQKMNRSQM--KMNDRTS--DYILKVSGRDEY    275

PK3CB_HUMAN      257 VFGDHPLIQFQYIRNCVMNRALPHFILVECCKIKKMYEQEMIAIEAAIN-    305
                     :.||:|||||.||:..:.:.|:|: : |    ::.:| :    :|:.||
P91634_DROME     276 LLGDYPLIQFLYIQEMLSDSAVPN-V-V----LQSVY-R----LESYINH    314

PK3CB_HUMAN      306 RNSSNLPL---PLPPKKT-RI---ISHVWENNNPFQIVL--VKGNKLNTE    346
                     .|...: :   |||.|:| .:   ||.:|:..|.||:.|  : .| :|.:
P91634_DROME     315 HNEQAM-VTKRPLPKKRTVHLHKSISSLWDMGNYFQLTLHSI-SN-VNFD    361

PK3CB_HUMAN      347 ET--VK--VHVRAGLFHGTELLC--K-TIVSSEVSGKN-D-HIWNEPL--    385
                     :|  :|  ||| . |:||.:.||  : |  .|. :| | | .::|: |
P91634_DROME     362 KTRALKVGVHV-C-LYHGDKKLCAQRST--DSP-NG-NFDTFLFND-LVM    404

PK3CB_HUMAN      386 EFDINICDLPRMARLCFAVYAVLDKV-KTKKSTKTINPSKYQTIRKAGK-    433
                     :|||.:.:||||.|||..::.| .|: ::|||:   | :|  .|  |.|
P91634_DROME     405 DFDIQMRNLPRMTRLCIVIFEV-TKMSRSKKSS---N-NK--DI--ALKD    445

PK3CB_HUMAN      434 VHY---PVAWVNTMVFDFKGQLRTGDIILHSWSSFPDELE--EMLNPMGT    478
                     |.|   |:|||||.:||.|..||||...|::| ::.|:::  |:.:|:||
P91634_DROME     446 VPYNKNPLAWVNTTIFDHKDILRTGRHTLYTW-TYADDIQSVEVFHPLGT    494

PK3CB_HUMAN      479 VQTNPYTENATALHV--KFPENKKQPYYYPPFDKII-EKAAEIASSDSAN    525
                     ::.||..|.. || |  .|..:......||. :::: :.||:   .:..|
P91634_DROME     495 IEPNPRKEEC-AL-VDLTFLSSGTGTVRYPS-EEVVLQYAAD---REQVN    538

PK3CB_HUMAN      526 -VSSR--GG-KKFLPV--LKEI------LDRDPLSQLCENEMDLIWTLRQ    563
                      : .|  .| :|  |:  |||:      ||:  :.:.:.:.:.||..|.
P91634_DROME     539 RL-QRQLAGPEK--PIKELKELMANYTGLDK--IYEMVDQDRNAIWERRN    583

PK3CB_HUMAN      564 D-CREIFPQSLPKLLL-SIKWNKLE--DVAQLQALL-QIWP--KLPPREA    606
                     | .||: |:.| .:|| .:.| | |  |||.:..|| | ||  .: .| :
P91634_DROME     584 DILREL-PEEL-SILLHCVYW-K-ERDDVADMWYLLKQ-WPLISI-ER-S    626

PK3CB_HUMAN      607 LELLDFNYPDQYVREYAVGCLRQMSDEELSQYLLQLVQVLKYEPFL--DC    654
                     |||||:.|||..||.:|:.||..:.||:|..|||||||.:|:|.:|  |.
P91634_DROME     627 LELLDYAYPDPAVRRFAIRCLHFLKDEDLLLYLLQLVQAIKHESYLESDL    676

PK3CB_HUMAN      655 ALSRFLLERALGNRRIGQFLFWHLRSEVHIPAVSVQ--FGVILEAYCRGS    702
                     .:  |||||||.|:|||.:.|||||||:..|  |:|  ||::||.|.:|.
P91634_DROME     677 VV--FLLERALRNQRIGHYFFWHLRSEMQTP--SMQTRFGLLLEVYLKGC    722

PK3CB_HUMAN      703 VGHMKVLSKQVEALNKLKTLNSLIKLNAVKLNRAKG-KEAMHTCLKQSAY    751
                     ..|:..|.||:..|.|||. .|||   | |    || ||.:.|.| |. :
P91634_DROME     723 KHHVAPLRKQLHVLEKLKQ-GSLI---A-K----KGSKEKVKTML-QD-F    761

PK3CB_HUMAN      752 -RE----AL-SDLQSPLNP---CVILSELYV--EKCKYMDSKMKPLWLVY    790
                      |:    |: .::|:||||   |   |.  |  ::||.|||||:|||:|:
P91634_DROME     762 LRDQRNSAVFQNIQNPLNPSFRC---SG--VTPDRCKVMDSKMRPLWVVF    806

PK3CB_HUMAN      791 NN-KVFGEDSVGVIFKNGDDLRQDMLTLQMLRLMDLLWKEAGLDLRM-LP    838
                     .| .|...| |.:||||||||||||||||||||:||.|||..|:|.|| :
P91634_DROME     807 ENADVNASD-VHIIFKNGDDLRQDMLTLQMLRVMDQLWKRDGMDFRMNI-    854

PK3CB_HUMAN      839 YGCLATGDRS-GLIEVVSTSETIADIQLNSSNV-AAAAAFNKDALLNWLK    886
                     |.|::. ::| |:||||..:||||:|| ....: :|.:.|.|.:||:|||
P91634_DROME     855 YNCISM-EKSLGMIEVVRHAETIANIQ-KEKGMFSATSPFKKGSLLSWLK    902

PK3CB_HUMAN      887 EYNS-GDDLDRAIEEFTLSCAGYCVASYVLGIGDRHSDNIMVKKTGQLFH    935
                     |:|. .|.|::||.||||||||||||:||||:.||||||||||:.|||||
P91634_DROME     903 EHNKPADKLNKAINEFTLSCAGYCVATYVLGVADRHSDNIMVKRNGQLFH    952

PK3CB_HUMAN      936 IDFGHILGNFKSKFGIKRERVPFILTYDFIHVIQQGKTG-N-TE-K-FGR    981
                     ||||||||:||.|.|::||||||:||:||::||  .| | | .| | |..
P91634_DROME     953 IDFGHILGHFKEKLGVRRERVPFVLTHDFVYVI--NK-GFNDRESKEFCH    999

PK3CB_HUMAN      982 FRQCCEDAYLILRRHGNLFITLFALMLTAGLPELTSVKDIQYLKDSLALG   1031
                     |::.||.|:|:||:||.|.::|::|:::.|||||:|.||:.|||:::|.|.
P91634_DROME    1000 FQELCERAFLVLRKHGCLILSLFSMMISTGLPELSSEKDLDYLRETLVLD   1049

PK3CB_HUMAN     1032 KSEEEALKQ-FKQKFDEALRESWTTKVNWMAHTVRKD   1067
                     .:||:| :: |:.||.|||..||.|.:||.:|...|:
P91634_DROME    1050 YTEEKA-REHFRAKFSEALANSWKTSLNWASHNFSKN   1085

--------------------------------------------
Name 1: KTHY_HUMAN
Name 2: KTHY_YEAST

Thymidylate kinase OS=Homo sapiens GN=DTYMK PE=1 SV=4
Thymidylate kinase OS=Saccharomyces cerevisiae (strain ATCC 204508 / S288c) GN=C
DC8 PE=1 SV=2
```

```
Needed for following interaction(s):
yeast   physical        KTHY_HUMAN      BLM_HUMAN       DTYMK   BLM     KTHY_YEA
ST      SGS1_YEAST
yeast   genetic KTHY_HUMAN      BLM_HUMAN       DTYMK   BLM     KTHY_YEAST
SGS1_YEAST

Length sequence 1:      212
Length sequence 2:      216
Sequence #1: KTHY_HUMAN
Sequence #2: KTHY_YEAST
Length #1: 230
Length #2: 230
Matrix: BLOSUM62
Gap open: 2.0
Gap extend: 1.0
Length: 230
Identity: 104/230 (45.22%)
Similarity: 135/230 (58.70%)
Gaps: 50/230 (21.74%)
Score: 491.00

KTHY_HUMAN          1 MAARRGALIVLEGVDRAGKSTQ-S---RKLVEALCAAGHRAELLRFPERS     46
                      |.. ||.||::||:||.||:|| :   :|| :..|      :||:|||||
KTHY_YEAST          1 MMG-RGKLILIEGLDRTGKTTQCNILYKKL-QPNC------KLLKFPERS     42

KTHY_HUMAN         47 TEIGKLLSSYLQKKS-DVEDHSVHLLFSANRWEQVPLIKEK-LSQGVTLV     94
                      |.||.|::.||...| .:.|.::||||||||||.|..|| | |.:|..:|
KTHY_YEAST         43 TRIGGLINEYLTDDSFQLSDQAIHLLFSANRWEIVDKIK-KDLLEGKNIV     91

KTHY_HUMAN         95 VDRYAFSGVAFTGAK-EN-FSLDWCKQPDVGLPKPDLVLFLQLQ-LAD--    139
                      :|||.:||||::.|| .| ..||||.||||||.||||.|||..| : |
KTHY_YEAST         92 MDRYVYSGVAYSAAKGTNGMDLDWCLQPDVGLLKPDLTLFLSTQDV-DNN    140

KTHY_HUMAN        140 AAKRGAFGHERYENGAFQERALRCFHQLMKDTTLNWKMVDASKSI-EAVH    188
                      |.|.| ||.||||...|||:        :|.|.:  |::|  |.| :. .
KTHY_YEAST        141 AEKSG-FGDERYETVKFQEK--------VKQTFM--KLLD--KEIRKG-D    176

KTHY_HUMAN        189 EDIRVLSEDAIRTATEKPLG----E-L-WK    212
                      |.|.::  | :   |.|  |    | | |:
KTHY_YEAST        177 ESITIV--D-V---TNK--GIQEVEALIWQ    198

--------------------------------------------
Name 1: DOCK3_HUMAN
Name 2: YL422_YEAST

Dedicator of cytokinesis protein 3 OS=Homo sapiens GN=DOCK3 PE=1 SV=1
DOCK-like protein YLR422W OS=Saccharomyces cerevisiae (strain ATCC 204508 / S288
c) GN=YLR422W PE=1 SV=1

Needed for following interaction(s):
yeast   genetic RASK_HUMAN      DOCK3_HUMAN     KRAS    DOCK3   IRA2_YEAST
YL422_YEAST

Length sequence 1:      2030
Length sequence 2:      1932
Sequence #1: DOCK3_HUMAN
Sequence #2: YL422_YEAST
Length #1: 2312
Length #2: 2312
Matrix: BLOSUM62
Gap open: 2.0
Gap extend: 1.0
Length: 2312
Identity: 610/2312 (26.38%)
Similarity: 970/2312 (41.96%)
Gaps: 926/2312 (40.05%)
Score: 2116.00

DOCK3_HUMAN         2 WTPTEEEK--YGVVICSF----RGSVPQGLVLE--------IG-ETVQIL     36
                      |.||  ::  |||::.||    |  .|: ||.|        :| | |.:.
YL422_YEAST         9 WLPT--DRLIYGVLVKSFLPLQR--YPE-LVYENSNYANVYVGAE-VYVF     52

DOCK3_HUMAN        37 E-----K-CEGWY---RG-----VS---TKK---PNVKG---IFPANYIH     63
                      |     | |.. |   |.     :|   :..   |:||.   |||..|:|
YL422_YEAST        53 EESVDKKWCRA-YQCLRPFPEEFISNMNSANDVLPDVKPKVVIFPRKYVH    101

DOCK3_HUMAN        64 L--KKAIVSN----RG-QYETVVPL-----E-----DSI-V--T-EV-TA     91
                      .  :|| ||.    :. ..|...||     |     ||: |  | :: |.
YL422_YEAST       102 FEAEKA-VSTMPFFKAPSAEDFKPLISKECESRSFCDSLYVSSTDDISTG    150

DOCK3_HUMAN        92 ----T---------LQEWASLWK-Q----L-YV-KHKV----DL--F--Y    113
                          |         .|: .| :| :    | .: .| |    .:  |  |
YL422_YEAST       151 KPRKTPRPPFPFFRYQK-RS-FKDEMGPILSLISSH-VYSMYSIGEFSIY    197

DOCK3_HUMAN       114 -KLRHVMNEL---ID-LR-RQLLSGHL-TQ-DQ---VR---EVKRHIT-V    148
                       |:  :   :|  :| :| |  ||.:| |: ::   :|   .:  |  | :
YL422_YEAST       198 RKM--I--KLYYDLDTIRFR--LSMNLTTEAEKINLIRAATSL-R--TKI    238

DOCK3_HUMAN       149 -R-LD--W-GNEHLGL-DLVPRK-D---FE-V----VDSDQ-IS--VSDL    180
                       : |.  : .|: | : :..||. |   || :    :|:.: :|  : |
YL422_YEAST       239 AKFLSSTYRKNK-L-IANSTPRNPDPYGFEGIFARDIDTGELLSYEI-D-    284

DOCK3_HUMAN       181 YKMH-L-SSRQSVQQS-TSQVDTMRP---------RHG--ETCRMPVPHH    216
                       |:. | ||  |:... |:...|: |         .:|  .|.|..:
YL422_YEAST       285 -KLRTLVSS--SMLCGLTNNFPTV-PVVESDDESSSNGLFGTVRSSI---    327

DOCK3_HUMAN       217 FFLSLKSFTYN-TIGED---TDVFFSL--YDMREGK-QI-SERFLVRLNK    258
                       .::||...:: :| .|   .|:   |:  | :|. | :: :|.|  .:.|
YL422_YEAST       328 -LVNLKDLAWDPSI-SDPKYQDL--SICVY-LRT-KDEVLTESF--TMTK    369

DOCK3_HUMAN       259 NGGPRNPEK-IERMCA-LFTD-LSS---KDMKRDLYIVAHVIR--IG---    297
                      :.   |.|. ::.:.| ||.: |.:   |: |  :|:|. |::   |.
YL422_YEAST       370 SS---NMESALDEIPAMLFKNILETIVHKN-K--VYLVV-VLKETIAITT    412

DOCK3_HUMAN       298 RM---L--LN----DSKK-GP--P------H-L-HYRRPYG-CA-VLSIL    325
                      ..   :  .|    :|.. .|  |      : : |.::  | .|  |::|.
YL422_YEAST       413 ETAPEISSYNISTEESSSHSPFSPFNSSTENKIDHVKK--GLAAGVINIS    460

DOCK3_HUMAN       326 DVLQ---SLTEV--KEEKDFVLKVYTC------N-NES---E--W-SQIH    357
                      .|.:   .|: |  |.:: |.|.:|:.      | |.|   :  |  ..|
YL422_YEAST       461 PVFKFYNGLS-VANKAQR-FNLYLYSSDSSDSQNFNSSKDADLGWGGLI-    507

DOCK3_HUMAN       358 EN-IIRKSSAK-YSA-PSA-SHGLIISL-QLLRGDMEQIRRENPMIFNRG    402
                       | || |.|:: .|. |.| |  |.::: ::: |  :| ..|.  :.:..
YL422_YEAST       508 -NKII-KDSSEGVSVNPRAVS--LSVTVKEII-G--KQ-EAEK--VLSTS    547

DOCK3_HUMAN       403 LA-ITRKLGFP----DVIM--PGDIRNDLYLTLEKGDFER-G--G-KSVQ    441
                      |. | |.:  |    |. |  ..: |  :||.|  |   | .  | .:..
YL422_YEAST       548 LVPI-RSI--PTYFYDT-MFSQAE-R--IYLNL--G---RVSLYGLPAAD    585

DOCK3_HUMAN       442 KNIE-VTMYV-LYADGEILKDCIS-LGSGEPNRS-SYHSFVLYHSN-SPR    486
                      .||| ||  | :....:.:|.|.: |   | .|| .: .||....| |
YL422_YEAST       586 TNIENVT--VQISCRNKAVKFCKNKL---E-ERSGDW-KFVSVRPNES--    626

DOCK3_HUMAN       487 WGEIIKLP-IP-I--D---R---F-RGSHL--RFEFRHCSTKDKGE----    519
                      .||.|::. :. :  |   |   : .| .|  :... |  .|.|.|
YL422_YEAST       627 IGESIRIEGVENMNEDETLRVLVYLNG-FLMAKSNI-H--IKKKNEIIEY    672

DOCK3_HUMAN       520 KKLFGFAFSTLMRDDGTTLSDDIH-ELYVYKCDENSTFNNHALYLGLPCC    568
                      :|  |..|. :|......|   || ||      |       |.|.|
YL422_YEAST       673 RK--GTVFQ-IMSSKSVPL---IHLEL------E-------ASYFG----    699

DOCK3_HUMAN       569 KEDYNGCPNIPSSLIFQRSTKESFFISTQLSSTKLTQNVDLLALLKWKAF    618
                      :. || . | |:  |    |  :|.:   | .||   ||:         |
YL422_YEAST       700 RR-YN-I-N-PA--I----T--NFLV---L-QTK---NVE---------F    721

DOCK3_HUMAN       619 PDRIMDVLGRLR-H--VSGEEI--VKFLQDIL---DT-L--F-VILDD-N    655
                       |:      :|: |  |:.:::  |.| :|:|   || |  : ::|:. |
YL422_YEAST       722 -DQ------QLKEHYSVTLKQLNNVSF-KDLLKHFDTILAHYLLLLESVN    763

DOCK3_HUMAN       656 --TEKYG----L--LVFQSLV-FIINL-L--RD-IKY-FH--FR------    683
                        |:|.|    |  :||...| | :|| |  :: .:| |:  ::
YL422_YEAST       764 EATDKKGPSSSLPNIVFSEFVKF-LNLMLTHQENSRYWFNRLYKKVMSKE    812

DOCK3_HUMAN       684 -------PV----MDTYI--QKH--F--AG-A----------LA----YK    701
                             |:    |.| |  :.|  :  .| |          ||    :.
YL422_YEAST       813 LECPNVAPILIKHMTT-IFDRSHSSWTRTGTAICRTILYIIVLAIGSSHS    861
```

```
DOCK3_HUMAN       702 -EL------IRCL-KWYM--DCSAELIRQDHIQ--E---AMRALEYL---    733
                       |:      .|.| |:.|  |   |.|..|.|.  |   :|  ||.:
YL422_YEAST       862 DEMPNFSHFFRSLHKFLMLAD---EPIMADQILLIESIPSM--LETMTNH    906

DOCK3_HUMAN       734 FKFIVQS--R--I-LYSRATCGMEEE--Q--F-RS-SI-QELFQSIRF--    769
                      .|  |:.  |  | |:.  .| .|:|  |   : |. |: :|.:.:.:|
YL422_YEAST       907 CK--VEDLVRFAIGLFE--CC-QEKEMNQKMYSRPLSVREEEYLNTKFNC    951

DOCK3_HUMAN       770 VLSLDSRNSETLLFTQAALLNSFPTIFDEL-LQMFTVQEVAEFVRGTLGS    818
                      :|.|  .|.: :|  |..|.|: .:: |:| || | :.:..|::   | :
YL422_YEAST       952 LLKL--INKK-VL--QNYLTNT-ESV-DKLRLQ-F-LSKTLEWL---L-T    988

DOCK3_HUMAN       819 MPST------VHIGQSMDVVKLQSIARTV--DSRLFSFSE--SRR-I-LL    856
                       |.|      .|: :|:.:|  .|:..|:  |   :.| :  .|. | ||
YL422_YEAST       989 -PYTPGDDKCFHV-ESLRLV--NSVFITIIED---YKF-DMLQRNLIRLL   1030

DOCK3_HUMAN       857 PVVL--HHIHLHLRQ-QK-ELL----ICSGILGSIF--SIVKTSSLEAD-    895
                      | .|  ..:||. |. :| .|:    :.: :|   |  .| ..:.:..|
YL422_YEAST      1031 P-YLCKSFVHLR-RYCKKARLMRPRRVFT-ML---FPREI-PCNYIPVDS   1073

DOCK3_HUMAN       896 VMEEVEMMVESLLDVLLQTLLTIMSKSHAQEAVRGQRCPQCTAEI-TG--    942
                      ::.: |::||    |||:  |.|:         :    | :.| :| :.
YL422_YEAST      1074 IVND-EVVVE----VLLE--LAII--------I----C-EIT-KIASSRF   1102

DOCK3_HUMAN       943 -EYVSCLLS-LLRQMC--DTHFQHLLDNFQSKDEL-KE--F-LLK-IFCV    983
                       .|.|  .| :: .:|  ||.||   .||.|: :: .|  : :.| :| :
YL422_YEAST      1103 PSYQS--FSEII-NLCDKDTLFQ---SNFYSR-QITNENVYTITKTVF-L   1144

DOCK3_HUMAN       984 FRNLMKMSVFP--RDWM-VMRLL-TSNIIVTTV--QYL----S-SALHKN   1022
                      |   .|...||  : |: |..|| .|::|:.::  .|:    | |. .|.
YL422_YEAST      1145 F---FKQDWFPGMK-WLGVSALLGRSSLILLSLCKDYIIENNSPSP-SKE   1189

DOCK3_HUMAN      1023 FTETDFDFKVWNSYFSLAVLFI-NQPSLQL-EI-ITSAKRKKILDKY---   1066
                       :|...|.::|..|..: :|.: |..|..| :: ||  .||.:   |
YL422_YEAST      1190 -SEKRVDMRLWAEYVKV-ILLVSNHKSASLTKLAIT--PRKAV---YLIS   1232

DOCK3_HUMAN      1067 GDMRVMMAYELFS-MWQNL--GEH-KIHFIPGMIGPFLGVTLVP-QPE--   1109
                      ||::.:.|| :.: .|..|  | | .|.:.. ..|  || .|.. |.|
YL422_YEAST      1233 GDLKKISAY-ILNECWDALATG-HYNITYAK-KYG--LG-ALSDCQFELF   1276

DOCK3_HUMAN      1110 VRN-IMI-PIF------H-D-------MMDWEQRKN-G-NF-K-----Q-   1134
                      |.| .:| .||      | |       :: |    . | || :     |
YL422_YEAST      1277 VHNQFLIREIFIFAFHRHIDATRICCKIL-W----GLGLNFWRIFGSLQP   1321

DOCK3_HUMAN      1135 -VEAELIDKLDS--MVSEGK---GDESYREL--F-SLLTQLFGPYPSLLE   1175
                       |.| .|.:|.|  .:  ||   .|  | ||  | |.   ||  :  ::.
YL422_YEAST      1322 AVNA-CIPELFSAYQI--GKLRLND--Y-ELERFVSC---LF--F--MMH   1358

DOCK3_HUMAN      1176 KV-EQETW--------RE-TGISFVTSVTRLMERLLDYRDCMKG-EETEN   1214
                       | :.:|:         |: .|  |: .:..  |  : |:  :.. ||.::
YL422_YEAST      1359 -VPDSDTFFPACMDFLRDLLG--FL-HIVN--E--I-YK--IPNQEEFDD   1397

DOCK3_HUMAN      1215 KKIGCTV-NL-MNF-YKSEINKEEMYIRYIHKLC-DM--H-LQAENYTEA   1257
                      .:   |. :: | | |..|.|:.|::    ||:. |:  | :|.:::.:|
YL422_YEAST      1398 DR---TARHIEM-FEYLLEANRPELF----HKMIYDLFIHFIQKKDFVQA   1439

DOCK3_HUMAN      1258 AFTL-LL---YCELLQWE--DRPLRE---F--LHYPSQTEWQRKEGLCRK   1296
                      |.:| ||   |.    |:  | .| |   |  |  |.|:.::|||.|.::
YL422_YEAST      1440 ALSLELLAGTYA----WDSND-TL-EAISFPPL--PEQSSFERKEYLLKE   1481

DOCK3_HUMAN      1297 IIHYFNKG-KSWEFGIPLCREL--ACQYESL-YDYQSLSWIR-KMEASYY   1341
                      ....|::| |. |..:.:.::|  |  |:.: ||...|:::. :: |..|
YL422_YEAST      1482 SARNFSRGQKP-EKALAVYKDLIKA--YDEINYDLNGLAFVHDQI-AGIY   1527

DOCK3_HUMAN      1342 DNIMEQ--QRLEPEFFRVGF--YGRKFPFFLRNKEYVCRGHDYERLEAFQ   1387
                      ..:  |  .||.|.:|:|.|  :|  ||..||||.:|..|..:|.:.:..
YL422_YEAST      1528 TRL--QSIDRLVPTYFKVSFMGFG--FPKSLRNKSFVFEGLPFEHITSMH   1573

DOCK3_HUMAN      1388 QRML-SEFPQAVAMQH-PN--H--PD-DAILQCD---AQYLQIYA-VTP-   1425
                      .|:| | :       | .|  |  .: |.:|. :   .:|:.: | |.|
YL422_YEAST      1574 DRLLRS-Y-------HGSNIVHSQEEVDMLLM-NPPMGKYIHV-ASVEPC   1613

DOCK3_HUMAN      1426 --IPD-YVDVLQMDR---VPDRVKSFYRV-N-NVRKF---RYDR-PFHKG   1463
                        |.| | :  ..|:   :.::|: .| : | ::|.|   |  | |..||
YL422_YEAST      1614 LSISDNY-N--SSDKKSSINNKVR-MY-IENRDLRTFSNSR--RLPGAKG   1656

DOCK3_HUMAN      1464 PKDKENEFKSLWIERTTL-TLTHSLPGISRWFEVERRELVEV-----SPL   1507
                      ..|      ||:|..|. |: ::.|.:     :.|.|:|:|     |||
YL422_YEAST      1657 VTD-------LWVEEYTYHTM-NTFPTL-----MNRSEIVKVTKSKLSPL   1693

DOCK3_HUMAN      1508 ENAI---QVVENKNQELRSLISQYQ-HKQV--HGNIN-L---LSMCLN--   1545
                      ||||   ||   |.|||..|  :.. :|.:  ||::| |   ||  .|
YL422_YEAST      1694 ENAIRSLQV---KIQELYGL--ENMCNKTLKDHGDVNDLFTELS--TNIT   1736

DOCK3_HUMAN      1546 GVIDAAVNGGIARYQEAFFD----KDYINKHPGDAEKITQL--KELMQEQ   1589
                      |.|.|.|||||::| :||.:    |.: :  ..|..::| |  .||
YL422_YEAST      1737 GTISAPVNGGISQY-KAFLEPSTSKQF-S--TDDLGRLT-LAFDEL----   1777

DOCK3_HUMAN      1590 VHVLG-VGLAVH-EKFVHP--EMRPLHKKLIDQFQMMRASLYHE-F-PGL   1633
                      |.||| . |.:| | .: |  :::|.|    | . ::|  |:.| | ..:
YL422_YEAST      1778 VAVLGRC-LTLHAE-LL-PSKDLKPSH----D-L-LVR--LFEENFAEEI   1816

DOCK3_HUMAN      1634 DK----LSPACSGTSTPRGNVLASHSPMSPESIKMTHRHSPMNLMG--TG   1677
                      ::    ||.|  ..|  |.|::.:.       | ::|: :| |...  :|
YL422_YEAST      1817 ERYSRTLSEA--NRS--RNNMITAR-------I-ISHK-NP-NKKASFSG   1852

DOCK3_HUMAN      1678 R-HSSSSLSSHASSEAGNMVML--GDGSMGDAPEDLYHHMQLAYPNPRYQ   1724
                      | |.:|. |:|  |:   .| |  .| |.|  |..|      | : . :|
YL422_YEAST      1853 RDHHTSG-SNH--SQ---FV-LEHSD-SFG--PNSL-----L-F-G-KY-   1883

DOCK3_HUMAN      1725 GSVTNVSVLSSSQASPSSSSLSSTHSAPSQMITSAPSS---ARGS-PSL-   1769
                        :|.  .||.|  | ::|||..  |.    |.|..||   | || |:.
YL422_YEAST      1884 --LTR--TLSHS--S-TTSSLDK--SG----IVSGTSSTFLA-GSQPNTN   1919

DOCK3_HUMAN      1770 PD---K--YRHA   1776
                      .|   |  |.|:
YL422_YEAST      1920 TDSQHKHDYSHS   1931

--------------------------------------------
Name 1: TOP1_HUMAN
Name 2: TOP1_YEAST

DNA topoisomerase 1 OS=Homo sapiens GN=TOP1 PE=1 SV=2
DNA topoisomerase 1 OS=Saccharomyces cerevisiae (strain ATCC 204508 / S288c) GN=
TOP1 PE=1 SV=2

Needed for following interaction(s):
yeast   genetic TOP1_HUMAN      MUS81_HUMAN     TOP1    MUS81   TOP1_YEAST
MUS81_YEAST

Length sequence 1:      765
Length sequence 2:      769
Sequence #1: TOP1_HUMAN
Sequence #2: TOP1_YEAST
Length #1: 837
Length #2: 837
Matrix: BLOSUM62
Gap open: 2.0
Gap extend: 1.0
Length: 837
Identity: 381/837 (45.52%)
Similarity: 493/837 (58.90%)
Gaps: 186/837 (22.22%)
Score: 1840.00

TOP1_HUMAN         43 DREKSKH--SNSEHKD---SE--KKHK--EKEKTKHKDGSSEKHKDKHKD     83
                      |..|..|  |:.:..|    |:  ||.|  .......:| .:|.:     |
TOP1_YEAST          5 DASKVNHELSSDDDDDVPLSQTLKKRKVASMNSASLQD-EAEPY-----D     48

TOP1_HUMAN         84 RDKEKRKEEKVRASGDAKIKKEKENGFSSPPQIKDEP-EDDGYFVP-PKE    131
                      .|      |.:  |    ||.|:|    :.  :||.|| :...  :| |
TOP1_YEAST         49 SD------EAI--S---KISKKK----TK--KIKTEPVQSSS--LPSP--     77

TOP1_HUMAN        132 DIKPLKRPRDEDDADYKPKKIKTE--DTK-KEKKRKLEEEEDGKLKKPKN    178
                         |.|:     .|..||||||.|  |.| |..| | ||:|: : || |
TOP1_YEAST         78 ---PAKK-----SATSKPKKIKKEDGDVKVKTTK-K-EEQEN-E-KK-K-    113

TOP1_HUMAN        179 KDKDKKVPEPDNKKKKPKKEEEQKWKWWEEERYPEG--IKWKFLEHKGPV    226
```

```
                       :::::: | |   || .||||::::||||:|.  |.  |||..|:|.|.:
TOP1_YEAST       114 REEEEE--E-D---KK-AKEEEEEYKWWEKEN--EDDTIKWVTLKHNGVI    154

TOP1_HUMAN       227 FAPPYEPLPENVKFYYDGK-VMKLSPKAEEVATFFAKML--DHEYTTKE-    272
                     |.|||:|||.::|.|||||  | .|.|:||||.|||.:|  ||   .|.
TOP1_YEAST       155 FPPPYQPLPSHIKLYYDGKPV-DLPPQAEEVAGFFAALLESDH---AKNP    200

TOP1_HUMAN       273 IFRKNFFKDW-R--KEMTNEEKN-I-ITNLSKCDFTQMSQYFKAQTEARK    317
                     :|:||||.|: :  || :....| | |...|:||||:|..||:.|.|.:|
TOP1_YEAST       201 VFQKNFFNDFLQVLKE-SGGPLNGIEIKEFSRCDFTKMFDYFQLQKEQKK    249

TOP1_HUMAN       318 QMSKEEKLK-IKEENEKLLKE-YGFCIMDNHKERIANFKIEPPGLFRGRG    365
                     |:::||| | |:.|.|| .:| |.||.:|..:|::.|||:|||.||||||
TOP1_YEAST       250 QLTSQEK-KQIRLEREK-FEEDYKFCELDGRREQVGNFKVEPPDLFRGRG    297

TOP1_HUMAN       366 NHPKMGMLKRRIMPEDIIINCSKDAKVPSP-PPGHKWKEVRHDNKVTWLV    414
                     .|||.|.||||:.||||::|.||||.|| | |.||||.|:||||.|.||.
TOP1_YEAST       298 AHPKTGKLKRRVNPEDIVLNLSKDAPVP-PAPEGHKWGEIRHDNTVQWLA    346

TOP1_HUMAN       415 SWTENIQGSIKYIML--NPSSRIKGEKDWQKYETARRLKKCVDKIRNQY-    461
                     .|.|||..|.||:.|  | || :||:.|::|:|.||:||..:|.||..|
TOP1_YEAST       347 MWRENIFNSFKYVRLAAN-SS-LKGQSDYKKFEKARQLKSYIDAIRRDYT    394

TOP1_HUMAN       462 REDWKSKEM-KVRQRAVALYFIDKLALRAGNEKEEGETADTVGCCSLRVE    510
                     | :.|||.| : ||:|||:|.||..|||||.||.|.| ||||||||||.|
TOP1_YEAST       395 R-NLKSKVMLE-RQKAVAIYLIDVFALRAGGEKSEDE-ADTVGCCSLRYE    441

TOP1_HUMAN       511 HINLHPELDGQEYVVEFDFLGKDSIRYYNKVPVEKRVFKNLQLFMEN--K    558
                     |:.|.|    ...|: |||||||||:|.:|.|:|:|||||.:| :.  |
TOP1_YEAST       442 HVTLKP----PNTVI-FDFLGKDSIRFYQEVEVDKQVFKNLTIF-KRPPK    485

TOP1_HUMAN       559 QPEDDLFDRLNTGILNKHLQDLMEGLTAKVFRTYNASITLQQQLKELTAP    608
                     ||...|||||:..||||:||:.|.||||||||||||.|:|.|| :| .|
TOP1_YEAST       486 QPGHQLFDRLDPSILNKYLQNYMPGLTAKVFRTYNASKTMQDQL-DL-IP    533

TOP1_HUMAN       609 DE-NIPA-KILSYNRANRAVAILCNHQRAPPK----TFEKS---MMNL--    647
                     :: :: | |||.||.|||.|||||||||...|    |.||:   :..|
TOP1_YEAST       534 NKGSV-AEKILKYNAANRTVAILCNHQRTVTKGHAQTVEKANNRIQELEW    582

TOP1_HUMAN       648 Q---TK--I-----D-AKK-----EQ---LA--D-A----R---RDL-K-    666
                     |   .|  |      | .||      |:   |.  | |     |   |:: |
TOP1_YEAST       583 QKIRCKRAILQLDKDLLKKEPKYFEEIDDLTKEDEATIHKRIIDREIEKY    632

TOP1_HUMAN       667 SAK-A---D----AK--VM----------K-DAK----TK--KV--VE--    685
                     ..| .    |     .|  ::           | |.|     .|  |.  ||
TOP1_YEAST       633 QRKFVRENDKRKFEKEELLPESQLKEWLEKVDEKKQEFEKELKTGEVELK    682

TOP1_HUMAN       686 ----S-KK-KA-VQRLEEQLMKLEVQATDREENKQIALGTSKLNYLDPRI    728
                         | :| || |::||:::....:|..|:|||.|::|||||:||:|||:
TOP1_YEAST       683 SSWNSVEKIKAQVEKLEQRIQTSSIQLKDKEENSQVSLGTSKINYIDPRL    732

TOP1_HUMAN       729 TVAWCKKWGVPIEKIYNKTQREKFAWAIDMADEDYEF    765
                     :|.:|||:.||||||:.||.||||.|||:..||::.|
TOP1_YEAST       733 SVVFCKKYDVPIEKIFTKTLREKFKWAIESVDENWRF    769

--------------------------------------------
Name 1: H2B1M_HUMAN
Name 2: H2B1_YEAST

Histone H2B type 1-M OS=Homo sapiens GN=HIST1H2BM PE=1 SV=3
Histone H2B.1 OS=Saccharomyces cerevisiae (strain ATCC 204508 / S288c) GN=HTB1 P
E=1 SV=2

Needed for following interaction(s):
yeast   genetic H2B1M_HUMAN     RASK_HUMAN      HIST1H2BM       KRAS    H2B1_YEA
ST      IRA2_YEAST

Length sequence 1:      126
Length sequence 2:      131
Sequence #1: H2B1M_HUMAN
Sequence #2: H2B1_YEAST
Length #1: 127
Length #2: 127
Matrix: BLOSUM62
```

```
Gap open: 2.0
Gap extend: 1.0
Length: 127
Identity: 89/127 (70.08%)
Similarity: 104/127 (81.89%)
Gaps: 8/127 (6.30%)
Score: 450.00

H2B1M_HUMAN        3 E--PV-KSAPVPKK-GSKKAINKAQKKDGKKRKRSRKESYSVYVYKVLKQ     48
                     |  |. | ||..|| .:||.   :...|||||.::|||:||.|:||||||
H2B1_YEAST         6 EKKPASK-APAEKKPAAKKT---STSTDGKKRSKARKETYSSYIYKVLKQ     51

H2B1M_HUMAN       49 VHPDTGISSKAMGIMNSFVNDIFERIAGEASRLAHYNKRSTITSREIQTA     98
                     .|||||||.|:|.|:||||||||||||.|||:||.|||:|||::||||||
H2B1_YEAST        52 THPDTGISQKSMSILNSFVNDIFERIATEASKLAAYNKKSTISAREIQTA    101

H2B1M_HUMAN       99 VRLLLPGELAKHAVSEGTKAVTKYTSS    125
                     |||:||||||||||||||:||||||:||
H2B1_YEAST       102 VRLILPGELAKHAVSEGTRAVTKYSSS    128

--------------------------------------------
Name 1: EF1A2_HUMAN
Name 2: EF1A_YEAST

Elongation factor 1-alpha 2 OS=Homo sapiens GN=EEF1A2 PE=1 SV=1
Elongation factor 1-alpha OS=Saccharomyces cerevisiae (strain ATCC 204508 / S288
c) GN=TEF1 PE=1 SV=1

Needed for following interaction(s):
yeast   physical        MUS81_HUMAN     EF1A2_HUMAN     MUS81   EEF1A2  MUS81_YE
AST     EF1A_YEAST

Length sequence 1:      463
Length sequence 2:      458
Sequence #1: EF1A2_HUMAN
Sequence #2: EF1A_YEAST
Length #1: 466
Length #2: 466
Matrix: BLOSUM62
Gap open: 2.0
Gap extend: 1.0
Length: 466
Identity: 369/466 (79.18%)
Similarity: 414/466 (88.84%)
Gaps: 11/466 (2.36%)
Score: 1984.00

EF1A2_HUMAN        1 MGKEKTHINIVVIGHVDSGKSTTTGHLIYKCGGIDKRTIEKFEKEAAEMG     50
                     |||||:|||:||||||||||||||||||||||||||||||||||||||:|
EF1A_YEAST         1 MGKEKSHINVVVIGHVDSGKSTTTGHLIYKCGGIDKRTIEKFEKEAAELG     50

EF1A2_HUMAN       51 KGSFKYAWVLDKLKAERERGITIDISLWKFETTKYYITIIDAPGHRDFIK    100
                     ||||||||||||||||||||||||:||||||.||.:|:||||||||||||
EF1A_YEAST        51 KGSFKYAWVLDKLKAERERGITIDIALWKFETPKYQVTVIDAPGHRDFIK    100

EF1A2_HUMAN      101 NMITGTSQADCAVLIVAAGVGEFEAGISKNGQTREHALLAYTLGVKQLIV    150
                     |||||||||||:||:|.||||||||||||:||||||||||:||||:||||
EF1A_YEAST       101 NMITGTSQADCAILIIAGGVGEFEAGISKDGQTREHALLAFTLGVRQLIV    150

EF1A2_HUMAN      151 GVNKMDSTEPAYSEKRYDEIVKEVSAYIKKIGYNPATVPFVPISGWHGDN    200
                     .||||||.:  :.|.|:.|||||.|.:|||:||||.|||||||||||:|||
EF1A_YEAST       151 AVNKMDSVK--WDESRFQEIVKETSNFIKKVGYNPKTVPFVPISGWNGDN    198

EF1A2_HUMAN      201 MLEPSPNMPWFKGW-KVERKEGNASGVSLLEALDTILPPTRPTDKPLRLP    249
                     |:|.:.|.||:|:|| | |.|.|...|.:||||:|.|..|:||||||||||
EF1A_YEAST       199 MIEATTNAPWYKGWEK-ETKAGVVKGKTLLEAIDAIEQPSRPTDKPLRLP    247

EF1A2_HUMAN      250 LQDVYKIGGIGTVPVGRVETGILRPGMVVTFAPVNITTEVKSVEMHHEAL    299
                     ||||||||||||||||||||:::|:||||||||..:||||||||||||.|
EF1A_YEAST       248 LQDVYKIGGIGTVPVGRVETGVIKPGMVVTFAPAGVTTEVKSVEMHHEQL    297

EF1A2_HUMAN      300 SEALPGDNVGFNVKNVSVKDIRRGNVCGDSKSDPPQEAAQFTSQVIILNH    349
                     .:.:||||||||||||||||:|||||||||:|:|||:..|.|.:.:||:|||
EF1A_YEAST       298 EQGVPGDNVGFNVKNVSVKEIRRGNVCGDAKNDPPKGCASFNATVIVLNH    347
```

```
EF1A2_HUMAN      350 PGQISAGYSPVIDCHTAHIACKFAELKEKIDRRSGKKLEDNPKSLKSGDA    399
                     |||||||||||:|||||||||:|.||.||.||||||||||:||.||||||
EF1A_YEAST       348 PGQISAGYSPVLDCHTAHIACRFDELLEKNDRRSGKKLEDHPKFLKSGDA    397

EF1A2_HUMAN      400 AIVEMVPGKPMCVESFSQYPPLGRFAVRDMRQTVAVGVIKNVEK--KSGG    447
                     |:|:.||.||||||:||:||||||||||||||||||||||:|:|  |
EF1A_YEAST       398 ALVKFVPSKPMCVEAFSEYPPLGRFAVRDMRQTVAVGVIKSVDKTEK---    444

EF1A2_HUMAN      448 AGKVTKSAQKAQKAGK    463
                     |.||||:||||.|  |
EF1A_YEAST       445 AAKVTKAAQKAAK--K    458

--------------------------------------------
Name 1: GRP78_HUMAN
Name 2: GRP78_YEAST

78 kDa glucose-regulated protein OS=Homo sapiens GN=HSPA5 PE=1 SV=2
78 kDa glucose-regulated protein homolog OS=Saccharomyces cerevisiae (strain ATC
C 204508 / S288c) GN=KAR2 PE=1 SV=1

Needed for following interaction(s):
yeast   physical        RASK_HUMAN      GRP78_HUMAN     KRAS    HSPA5   IRA2_YEA
ST      GRP78_YEAST

Length sequence 1:      654
Length sequence 2:      682
Sequence #1: GRP78_HUMAN
Sequence #2: GRP78_YEAST
Length #1: 687
Length #2: 687
Matrix: BLOSUM62
Gap open: 2.0
Gap extend: 1.0
Length: 687
Identity: 449/687 (65.36%)
Similarity: 542/687 (78.89%)
Gaps: 54/687 (7.86%)
Score: 2279.00

GRP78_HUMAN        3 LSLVA-AM--LLL-L-----SA---AR-AEEEDKKEDVGTVVGIDLGTTY     39
                     ||:|. |:  ::| |     |:   .| |  :| .|:.|||:||||||||
GRP78_YEAST       15 LSVVLYALFVVILPLQNSFHSSNVLVRGA--DD-VENYGTVIGIDLGTTY     61

GRP78_HUMAN       40 SCVGVFKNGRVEIIANDQGNRITPSYVAFTPEGERLIGDAAKNQLTSNPE     89
                     |||.|.|||:.||:||:||||||||||||| :.|||||||||||:.:||:
GRP78_YEAST       62 SCVAVMKNGKTEILANEQGNRITPSYVAFT-DDERLIGDAAKNQVAANPQ    110

GRP78_HUMAN       90 NTVFDAKRLIG-RTWNDPSVQQDIKFLPFKVVEKKTKPYIQVDIGGGQTK    138
                     ||:||.||||| : :||.|||:|||.|||.||.|..||.::|.: .|:.|
GRP78_YEAST      111 NTIFDIKRLIGLK-YNDRSVQKDIKHLPFNVVNKDGKPAVEVSV-KGEKK    158

GRP78_HUMAN      139 TFAPEEISAMVLTKMKETAEAYLGKKVTHAVVTVPAYFNDAQRQATKDAG    188
                     .|.|||||.|:|.|||:.||.|||.|||||||||||||||||||||||||
GRP78_YEAST      159 VFTPEEISGMILGKMKQIAEDYLGTKVTHAVVTVPAYFNDAQRQATKDAG    208

GRP78_HUMAN      189 TIAGLNVMRIINEPTAAAIAYGLDKREGEKNILVFDLGGGTFDVSLLTID    238
                     |||||||:||:||||||||||||||.:.|..|:|:||||||||||||:|:
GRP78_YEAST      209 TIAGLNVLRIVNEPTAAAIAYGLDKSDKEHQIIVYDLGGGTFDVSLLSIE    258

GRP78_HUMAN      239 NGVFEVVATNGDTHLGGEDFDQRVMEHFIKLYKKKTGKDVRKD-NRAVQK    287
                     ||||||.||:|||||||||||.:::...||.:|||.|.|| .| |:|:.|
GRP78_YEAST      259 NGVFEVQATSGDTHLGGEDFDYKIVRQLIKAFKKKHGIDV-SDNNKALAK    307

GRP78_HUMAN      288 LRREVEKAKRALSSQHQARIEIESFYEGEDFSETLTRAKFEELNMDLFRS    337
                     |:||.||||||||||...||||:||.:|.|.|||||||||||||:|||:.
GRP78_YEAST      308 LKREAEKAKRALSSQMSTRIEIDSFVDGIDLSETLTRAKFEELNLDLFKK    357

GRP78_HUMAN      338 TMKPVQKVLEDSDLKKSDIDEIVLVGGSTRIPKIQQLVKEFFNGKEPSRG    387
                     |:|||:|||:||.|:|.|:|:||||||||||||:|||::.:|:||:.|:|
GRP78_YEAST      358 TLKPVEKVLQDSGLEKKDVDDIVLVGGSTRIPKVQQLLESYFDGKKASKG    407

GRP78_HUMAN      388 INPDEAVAYGAAVQAGVLSGDQDTG--DLVLLDVCPLTLGIETVGGVMTK    435
                     ||||||||||||||||||||::  |  |:|||||..|||||||.|||||.
GRP78_YEAST      408 INPDEAVAYGAAVQAGVLSGEE--GVEDIVLLDVNALTLGIETTGGVMTP    455

GRP78_HUMAN      436 LIPRNTVVPTKKSQIFSTASDNQPTVTIKVYEGERPLTKDNHLLGTFDLT    485
                     ||.|||.:|||||||||||.||||||.||||||||.::|||:|||.|:||
GRP78_YEAST      456 LIKRNTAIPTKKSQIFSTAVDNQPTVMIKVYEGERAMSKDNNLLGKFELT    505

GRP78_HUMAN      486 GIPPAPRGVPQIEVTFEIDVNGILRVTAEDKGTGNKNK-ITITNDQNRLT    534
                     ||||||||||||||||.:|.||||:|:|.||||| |:: ||||||:.|||
GRP78_YEAST      506 GIPPAPRGVPQIEVTFALDANGILKVSATDKGTG-KSESITITNDKGRLT    554

GRP78_HUMAN      535 PEEIERMVNDAEKFAEEDKKLKERIDTRNELESYAYSLKNQI-GDKEKLG    583
                     .|||:|||.:|||||.||..:|.::::||:||:||:|||||: ||   ||
GRP78_YEAST      555 QEEIDRMVEEAEKFASEDASIKAKVESRNKLENYAHSLKNQVNGD---LG    601

GRP78_HUMAN      584 GKLSSEDKET-MEKAVEEKIEWLESH-QDADI-EDFKAKKKE-LEEIVQP    629
                     .||..||||| :: |..:.:|||:.: :.| | |||. :|.| |.::..|
GRP78_YEAST      602 EKLEEEDKETLLD-AANDVLEWLDDNFETA-IAEDFD-EKFESLSKVAYP    648

GRP78_HUMAN      630 IISKLYGSA-GPPPTG------E-E-DTA---EKDEL    654
                     |.|||||.| |   :|      | | |..   |.|||
GRP78_YEAST      649 ITSKLYGGADG---SGAADYDDEDEDDDGDYFEHDEL    682

--------------------------------------------
Name 1: PP2AB_HUMAN
Name 2: PP2A_DROME

Serine/threonine-protein phosphatase 2A catalytic subunit beta isoform OS=Homo s
apiens GN=PPP2CB PE=1 SV=1
Serine/threonine-protein phosphatase PP2A OS=Drosophila melanogaster GN=mts PE=2
 SV=1

Needed for following interaction(s):
fly     genetic RASK_HUMAN      PP2AB_HUMAN     KRAS    PPP2CB  RAS1_DROME
PP2A_DROME

Length sequence 1:      309
Length sequence 2:      309
Sequence #1: PP2AB_HUMAN
Sequence #2: PP2A_DROME
Length #1: 309
Length #2: 309
Matrix: BLOSUM62
Gap open: 2.0
Gap extend: 1.0
Length: 309
Identity: 292/309 (94.50%)
Similarity: 303/309 (98.06%)
Gaps: 0/309 (0.00%)
Score: 1615.00

PP2AB_HUMAN        1 MDDKAFTKELDQWVEQLNECKQLNENQVRTLCEKAKEILTKESNVQEVRC     50
                     |:|||.||:||||:||||||.||.|.||||||:||||||:||||||||:|
PP2A_DROME         1 MEDKATTKDLDQWIEQLNECNQLTETQVRTLCDKAKEILSKESNVQEVKC     50

PP2AB_HUMAN       51 PVTVCGDVHGQFHDLMELFRIGGKSPDTNYLFMGDYVDRGYYSVETVTLL    100
                     ||||||||||||||||||||||||||||||||||||||||||||||||||
PP2A_DROME        51 PVTVCGDVHGQFHDLMELFRIGGKSPDTNYLFMGDYVDRGYYSVETVTLL    100

PP2AB_HUMAN      101 VALKVRYPERITILRGNHESRQITQVYGFYDECLRKYGNANVWKYFTDLF    150
                     |||||||.||||||||||||||||||||||||||||||||||||||||||
PP2A_DROME       101 VALKVRYRERITILRGNHESRQITQVYGFYDECLRKYGNANVWKYFTDLF    150

PP2AB_HUMAN      151 DYLPLTALVDGQIFCLHGGLSPSIDTLDHIRALDRLQEVPHEGPMCDLLW    200
                     |||||||||||||||||||||||||:||||||||||||||||||||||||
PP2A_DROME       151 DYLPLTALVDGQIFCLHGGLSPSIDSLDHIRALDRLQEVPHEGPMCDLLW    200

PP2AB_HUMAN      201 SDPDDRGGWGISPRGAGYTFGQDISETFNHANGLTLVSRAHQLVMEGYNW    250
                     |||||||||||||||||||||||||||||:.|||||||||||||||||||
PP2A_DROME       201 SDPDDRGGWGISPRGAGYTFGQDISETFNNTNGLTLVSRAHQLVMEGYNW    250

PP2AB_HUMAN      251 CHDRNVVTIFSAPNYCYRCGNQAAIMELDDTLKYSFLQFDPAPRRGEPHV    300
                     ||||||||||||||||||||||||:|||||:||:||||||||||||||||
PP2A_DROME       251 CHDRNVVTIFSAPNYCYRCGNQAALMELDDSLKFSFLQFDPAPRRGEPHV    300
```

```
PP2AB_HUMAN      301 TRRTPDYFL    309
                     |||||||||
PP2A_DROME       301 TRRTPDYFL    309

--------------------------------------------
Name 1: VPS25_HUMAN
Name 2: Q9U354_CAEEL

Vacuolar protein-sorting-associated protein 25 OS=Homo sapiens GN=VPS25 PE=1 SV=
1
Protein W02A11.2, confirmed by transcript evidence OS=Caenorhabditis elegans GN=
vps-25 PE=2 SV=1

Needed for following interaction(s):
worm    genetic RASK_HUMAN      VPS25_HUMAN     KRAS    VPS25   LET60_CAEEL
Q9U354_CAEEL

Length sequence 1:      176
Length sequence 2:      183
Sequence #1: VPS25_HUMAN
Sequence #2: Q9U354_CAEEL
Length #1: 184
Length #2: 184
Matrix: BLOSUM62
Gap open: 2.0
Gap extend: 1.0
Length: 184
Identity: 81/184 (44.02%)
Similarity: 120/184 (65.22%)
Gaps: 19/184 (10.33%)
Score: 459.00

VPS25_HUMAN        2 AMSFEWPWQYRFPPFFTLQPNVDTRQKQLAAWCSLVLSFCRLH-KQSSMT     50
                     |.:|:|||||.||||||:|.:::|:.|||.||..||:.:.: | |..|:.
Q9U354_CAEEL       8 ASAFKWPWQYDFPPFFTIQKSLNTKDKQLEAWARLVIDYAQ-HNKIYSLD     56

VPS25_HUMAN       51 VMEAQESPLFNNVKLQRKLPVESIQIVLE--ELRKKGNL-EWLDKSKSSF     97
                     :.||..|.||||.||.|:|..:.:..||:  | :||  | |:.|..::.|
Q9U354_CAEEL      57 IAEATTSELFNNQKLNRRLSTDGVNTVLQYLE-QKK--LIEFTDNGRTRF    103

VPS25_HUMAN       98 LIMWRRPEEWGKLIYQW-VSRSGQN---NSVFTLYELTNGEDTEDEEFHG    143
                     .|.||||:.|..:|||| |    :|   |:..||||:|:|:||.:|.||.
Q9U354_CAEEL     104 HIFWRRPDVWANMIYQWAV----ENAFINTPLTLYEITHGDDTTNESFHN    149

VPS25_HUMAN      144 LD-EATLLRALQALQQEHKAEIITV-SDGRGVKF    175
                     |: | .|::||..|:::.:|:::.: .|..||||
Q9U354_CAEEL     150 LERE-ILMKALTCLEEQRRAQLMNIGGDNEGVKF    182

--------------------------------------------
Name 1: RL17_HUMAN
Name 2: RL17B_YEAST

60S ribosomal protein L17 OS=Homo sapiens GN=RPL17 PE=1 SV=3
60S ribosomal protein L17-B OS=Saccharomyces cerevisiae (strain ATCC 204508 / S2
88c) GN=RPL17B PE=1 SV=2

Needed for following interaction(s):
yeast   genetic RASK_HUMAN      RL17_HUMAN      KRAS    RPL17   IRA2_YEAST
RL17B_YEAST

Length sequence 1:      184
Length sequence 2:      184
Sequence #1: RL17_HUMAN
Sequence #2: RL17B_YEAST
Length #1: 191
Length #2: 191
Matrix: BLOSUM62
Gap open: 2.0
Gap extend: 1.0
Length: 191
Identity: 104/191 (54.45%)
Similarity: 131/191 (68.59%)
Gaps: 16/191 (8.38%)
Score: 532.00
```

```
RL17_HUMAN         1 MVRYSLDPENPTKSCKSRGSNLRVHFKNTRETAQAIKGMHIRKATKYLKD     50
                     |.||.....||.||..:|||.|||.||||||||||.|..:.||.||| |
RL17B_YEAST        1 MARYGATSTNPAKSASARGSYLRVSFKNTRETAQAINGWELTKAQKYL-D     49

RL17_HUMAN        51 -VTL--QKQCVPFRRYNGGVGRCAQAKQWGWTQGRWPKKSAEFLLHMLKN     97
                      | |  |: .:||||:|..:||.||.|::|.|:.|||.||.:|:..:|:|
RL17B_YEAST       50 QV-LDHQR-AIPFRRFNSSIGRTAQGKEFGVTKARWPAKSVKFVQGLLQN     97

RL17_HUMAN        98 AESNAELKGLDVDSLVIEHIQVNKAPKMRRRTYRAHGRINPYMSSPCHIE    147
                     |.:|||.||||...|.:.|||||:|||.||||||||||||.|.|||.|||
RL17B_YEAST       98 AAANAEAKGLDATKLYVSHIQVNQAPKQRRRTYRAHGRINKYESSPSHIE    147

RL17_HUMAN       148 MILTEKEQIVPKPEEEVAQKK--KI-S-QK-KLKKQK-LMA    182
                     :::||||:.|.|    .|:||  :: | |: ::..|| :.|
RL17B_YEAST      148 LVVTEKEEAVAK----AAEKKVVRLTSRQRGRIAAQKRISA    184

--------------------------------------------
Name 1: MP2K1_HUMAN
Name 2: DSOR1_DROME

Dual specificity mitogen-activated protein kinase kinase 1 OS=Homo sapiens GN=MA
P2K1 PE=1 SV=2
Dual specificity mitogen-activated protein kinase kinase dSOR1 OS=Drosophila mel
anogaster GN=Dsor1 PE=2 SV=2

Needed for following interaction(s):
fly     genetic RASK_HUMAN      MP2K1_HUMAN     KRAS    MAP2K1  RAS1_DROME
DSOR1_DROME

Length sequence 1:      393
Length sequence 2:      396
Sequence #1: MP2K1_HUMAN
Sequence #2: DSOR1_DROME
Length #1: 396
Length #2: 396
Matrix: BLOSUM62
Gap open: 2.0
Gap extend: 1.0
Length: 396
Identity: 253/396 (63.89%)
Similarity: 307/396 (77.53%)
Gaps: 41/396 (10.35%)
Score: 1325.00

MP2K1_HUMAN        6 PTPIQLNP--APDG----SAVNGTSSAETNLEALQKKLEELEL-D-EQQR     47
                     |||    |  .|.|    |.: |  ..:|:::||.:.||.|:: | |  |
DSOR1_DROME       26 PTP----PFKTPSGTDTHSLL-G--KPKTSIDALTETLEGLDMGDTE--R     66

MP2K1_HUMAN       48 KRLEAFLTQKQKVGELKDDDFEKISELGAGNGGVVFKVSHKPSGLVMARK     97
                     ||::.||:||:|:|||.|:|.||:.|||:||||||.||.|..:.|:||||
DSOR1_DROME       67 KRIKMFLSQKEKIGELSDEDLEKLGELGSGNGGVVMKVRHTHTHLIMARK    116

MP2K1_HUMAN       98 LIHLEIKPAIRNQIIRELQVLHECNSPYIVGFYGAFYSDGEISICMEHMD    147
                     |||||:||||:.||:|||:||||||.|:|||||||||||||||||||:||
DSOR1_DROME      117 LIHLEVKPAIKKQILRELKVLHECNFPHIVGFYGAFYSDGEISICMEYMD    166

MP2K1_HUMAN      148 GGSLDQVLKKAGRIPEQILGKVSIAVIKGLTYLREKHKIMHRDVKPSNIL    197
                     |||||.:||:||||||.|||:::||:|||:|||:.|.|:||||||||||
DSOR1_DROME      167 GGSLDLILKRAGRIPESILGRITLAVLKGLSYLRDNHAIIHRDVKPSNIL    216

MP2K1_HUMAN      198 VNSRGEIKLCDFGVSGQLIDSMANSFVGTRSYMSPERLQGTHYSVQSDIW    247
                     |||.||||:|||||||||||||||||||||||||||||||||||||||||
DSOR1_DROME      217 VNSSGEIKICDFGVSGQLIDSMANSFVGTRSYMSPERLQGTHYSVQSDIW    266

MP2K1_HUMAN      248 SMGLSLVEMAVGRYPIPPPD-AKELELMFGCQVEGDAAETPPRPRTPGRP    296
                     |:|||||||:|.||||||: | .||.:|     .|.||     .: |:|
DSOR1_DROME      267 SLGLSLVEMAIGMYPIPPPNTA-TLESIF-----ADNAE-----ES-GQP    304

MP2K1_HUMAN      297 LSSYGMDSRP-PMAIFELLDYIVNEPPPKLPSGVFSLEFQDFVNKCLIKN    345
                       :   | .| .||||||||||||||||||...:||.||:|||:.||.|.
DSOR1_DROME      305 --T---D-EPRAMAIFELLDYIVNEPPPKLEHKIFSTEFKDFVDICLKKQ    348

MP2K1_HUMAN      346 PAERADLKQLMVHAFIKRSDAEEVDFAGWLCSTIGLNQPSTP---T    388
```

```
                     |.||||||.|:.|.:|::::.||||.:||:|.|:.| .||||    |
DSOR1_DROME      349 PDERADLKTLLSHPWIRKAELEEVDISGWVCKTMDL-PPSTPKRNT    393

--------------------------------------------
Name 1: RU2A_HUMAN
Name 2: RU2A_YEAST

U2 small nuclear ribonucleoprotein A' OS=Homo sapiens GN=SNRPA1 PE=1 SV=2
U2 small nuclear ribonucleoprotein A' OS=Saccharomyces cerevisiae (strain ATCC 2
04508 / S288c) GN=LEA1 PE=1 SV=1

Needed for following interaction(s):
yeast   genetic RASK_HUMAN      RU2A_HUMAN      KRAS    SNRPA1  IRA2_YEAST
RU2A_YEAST

Length sequence 1:      255
Length sequence 2:      238
Sequence #1: RU2A_HUMAN
Sequence #2: RU2A_YEAST
Length #1: 282
Length #2: 282
Matrix: BLOSUM62
Gap open: 2.0
Gap extend: 1.0
Length: 282
Identity: 93/282 (32.98%)
Similarity: 130/282 (46.10%)
Gaps: 96/282 (34.04%)
Score: 329.00

RU2A_HUMAN         1 MVKLTAEL-IEQAAQ-YT---NA---VRD-----RELDLR--GYKIP-VI     34
                     | |.|..: |: |.| |.   |.   | |     |:|.|.  ...:| .:
RU2A_YEAST         1 M-KFTPSIVID-APQYYVDHFNGKYNV-DKCVILRDLQLETDSESMPSSL     47

RU2A_HUMAN        35 ENLGATLDQFDA-I-DFSDNE-IRKLDGFP-LLRR--LKTLLV-NNNRIC     77
                     ::|  |  : .. | |.::|: |  :  .| |.||  :.|||: .|| |
RU2A_YEAST        48 KHL--T--K-PTHILDLTNNDLI--M--IPDLSRRDDIHTLLLGRNN-I-     86

RU2A_HUMAN        78 RIGEGLD-QALPCL-TE-LILTNNSL--VELGDLDPL--A--SLKSLTYL    118
                      : | :| :.|| : .: |.|:|||:  .|  ||..|  |  :||:|| |
RU2A_YEAST        87 -V-E-VDGRLLP-MNVQNLTLSNNSIRRFE--DLQRLRRAPRTLKNLT-L    129

RU2A_HUMAN       119 SILRNPVTNKKH---YRLYVIYK-VPQVRVLDFQKVKLKERQEAEKM-F-    162
                      | .|.|.   |   ||.:|: : ||.:..||||.|..:||:.|  | |
RU2A_YEAST       130 -I-GNQVC---HLANYREHVL-RLVPHLETLDFQNVTAEERKSA--MSFP    171

RU2A_HUMAN       163 K---GKR-G-AQLA-KDIARRSKT---FNPGAGLPTDKKKGGPSPGDVE-    202
                     :   |.. | ...| :|...|.||   .|    |...|.    :   ||
RU2A_YEAST       172 RQADGDTLGPVNTAIRDNGSRDKTMEIMN----LVVSKM----T---VER    210

RU2A_HUMAN       203 --AIKNAIANASTLAEVERL-KGLLQSGQIPG    231
                       .:|..:|.|::|.|:.|| | || ||   |
RU2A_YEAST       211 RNELKKQLAEATSLEEIARLEK-LL-SG---G    237

--------------------------------------------
Name 1: TAF6_HUMAN
Name 2: TAF6_YEAST

Transcription initiation factor TFIID subunit 6 OS=Homo sapiens GN=TAF6 PE=1 SV=
1
Transcription initiation factor TFIID subunit 6 OS=Saccharomyces cerevisiae (str
ain ATCC 204508 / S288c) GN=TAF6 PE=1 SV=1

Needed for following interaction(s):
yeast   genetic TAF6_HUMAN      BLM_HUMAN       TAF6    BLM     TAF6_YEAST
SGS1_YEAST

Length sequence 1:      677
Length sequence 2:      516
Sequence #1: TAF6_HUMAN
Sequence #2: TAF6_YEAST
Length #1: 608
Length #2: 608
Matrix: BLOSUM62
Gap open: 2.0
Gap extend: 1.0
Length: 608
Identity: 197/608 (32.40%)
Similarity: 290/608 (47.70%)
Gaps: 182/608 (29.93%)
Score: 791.00

TAF6_HUMAN         1 MAEEKKLKLSNTV-LPSESMKVVAESMGIAQIQEETCQLLT-DEVSYRIK     48
                     |:.:::   |.|: .|.:::|.||||:|:..|.::..:.|. | |.|||.
TAF6_YEAST         1 MSTQQQ---SYTIWSPQDTVKDVAESLGLENINDDVLKALAMD-VEYRIL     46

TAF6_HUMAN        49 EIAQDALKF-MHMGKRQKLTTSDIDYALK-LKNVEPLYGFH-AQEF---I     92
                     ||.:.|:|| .| .||..|||.|:..||: | |||||||:: ..|.   :
TAF6_YEAST        47 EIIEQAVKFKRH-SKRDVLTTDDVSKALRVL-NVEPLYGYYDGSEVNKAV     94

TAF6_HUMAN        93 PF-RF-ASGGGRELYFY-EEKEVDLSD-IINTPLPRVP-LDVCLKAHWLS    137
                     .| :. .| ||:.:| | :|:|||. | :||.|||:|| |.. ...|||:
TAF6_YEAST        95 SFSKVNTS-GGQSVY-YLDEEEVDF-DRLINEPLPQVPRLPT-FTTHWLA    140

TAF6_HUMAN       138 IEGCQPAIPENP---------PP----A---PKEQQKAEATEPLKS--AK    169
                     :||.||||.:||          ||    |   .......: | |:.|  |.
TAF6_YEAST       141 VEGVQPAIIQNPNLNDIRVSQPPFIRGAIVTALNDNSLQ-T-PVTSTTAS    188

TAF6_HUMAN       170 PGQEED-GP---LKG-K-GQGATTADGKGKEKKAPPLLEGAPLRLKPRSI    213
                       ....| |.   |.. | ||  .|       |.|         || :|
TAF6_YEAST       189 -ASVTDTGASQHLSNVKPGQ--NT------EVK--------PL-VK----    216

TAF6_HUMAN       214 HELSVEQQLYY-KEI-T-EACVGS-C-EA----KRAEALQSIATDPGLYQ    254
                     |.||.|.|:|: |.| | .|  .| . ||    |:| ||.|:.||.||:|
TAF6_YEAST       217 HVLSKELQIYFNKVISTLTA--KSQADEAAQHMKQA-ALTSLRTDSGLHQ    263

TAF6_HUMAN       255 MLPRFSTFISEGVRVNVVQN--NLALLIYLMRMVKALMDNPT-LYLEKYV    301
                     ::|.|..||:|    .:.||  :|.||..::.|:.:|:.| | ::|:.|:
TAF6_YEAST       264 LVPYFIQFIAE----QITQNLSDLQLLTTILEMIYSLLSN-TSIFLDPYI    308

TAF6_HUMAN       302 HELIPAVMTCIVSRQL--CLRP-DVDN----H-W-----ALRDFAARLVA    338
                     |.|:|:::|.:::::|    .| | |:    | :     ||||||.|:.
TAF6_YEAST       309 HSLMPSILTLLLAKKLGG--SPKD-DSPQEIHEFLERTNALRDFAASLLD    355

TAF6_HUMAN       339 QICKHFSTTTNNIQSRITKTFTKSWVD-EKTPWTTRYGSIAGLA--ELGH    385
                     .:.|.|.....:::.|:|:|..|:::| .:. :.|.||.:.|::  | |
TAF6_YEAST       356 YVLKKFPQAYKSLKPRVTRTLLKTFLDINRV-FGTYYGCLKGVSVLE-G-    402

TAF6_HUMAN       386 DVIKTLI--L---PRL--QQEG---ERIRSVL--D-GPVLSNI---D-RI    418
                     :.|:..:  |   .||  .:.|   :.|...|  | .|..:..   : :|
TAF6_YEAST       403 ESIRFFLGNLNNWARLVFNESGITLDNIEEHLNDDSNPTRTKFTKEETQI    452

TAF6_HUMAN       419 GADHVQSLLLKHCAPVLAK-LRPPPDNQDAYRAEFGSLGPLLCSQVVKAR    467
                     ..|.|.|.||     ||.| |   |   |.|  | |. |      :  |
TAF6_YEAST       453 LVDTVISALL-----VLKKDL---P---DLY--E-GK-G-----E--K--    478

TAF6_HUMAN       468 AQAALQAQQVNRTTLTITQPRPTLTLSQAPQPGPRTPG--LLKVPGSIAL    515
                              |     | .:.:..| |.:.   |. |.|  :|| ... |
TAF6_YEAST       479 ---------V-----T-DEDKEKL-LERC---GV-TIGFHILK-RDD-A-    505

TAF6_HUMAN       516 PVQTLVSA    523
                       :.|:||
TAF6_YEAST       506 --KELISA    511

--------------------------------------------
Name 1: LTK_HUMAN
Name 2: IRE1_YEAST

Leukocyte tyrosine kinase receptor OS=Homo sapiens GN=LTK PE=1 SV=3
Serine/threonine-protein kinase/endoribonuclease IRE1 OS=Saccharomyces cerevisia
e (strain ATCC 204508 / S288c) GN=IRE1 PE=1 SV=2

Needed for following interaction(s):
yeast   physical        PTTG1_HUMAN     LTK_HUMAN       PTTG1   LTK     SECU_YEA
ST      IRE1_YEAST

Length sequence 1:      864
Length sequence 2:      1115
```

```
Sequence #1: LTK_HUMAN
Sequence #2: IRE1_YEAST
Length #1: 1216
Length #2: 1216
Matrix: BLOSUM62
Gap open: 2.0
Gap extend: 1.0
Length: 1216
Identity: 299/1216 (24.59%)
Similarity: 452/1216 (37.17%)
Gaps: 546/1216 (44.90%)
Score: 881.00

LTK_HUMAN          7 LLVW-FGAAGAIL-CSSP-GS-----Q----E---T----F---L-R--S     31
                     |||. |   .:|: ||.| .|      |     |    |     |   : :  :
IRE1_YEAST        13 LLVCVF---SSIISCSIPLSSRTSRRQIVEDEVASTKKLNFNYGVDKNIN     59

LTK_HUMAN         32 SPLPLASPSPR--D--P--KVSA-P-PSILEPA-S-PLNSPG--TEGSWL     69
                     ||:| |   ||  :  |  |:|: | |::|..| : ..|..|  ...| :
IRE1_YEAST        60 SPIP-A---PRTTEGLPNMKLSSYPTPNLLNTADNRRANKKGRRAANS-I    104

LTK_HUMAN         70 FSTCGASGRHGP-TQTQC--DGAYAGTS-VVVT--V-GAAGQL-----R-    106
                      |.          | .:.:.  :   ...| :::.  | |  | |      |
IRE1_YEAST       105 -SV--------PYLENRSLNE---LSLSDILIAADVEG--G-LHAVDRRN    139

LTK_HUMAN        107 G-VQLWRV-P-G---------PG--Q-Y--L-ISAYGAAGGKGAKN--HL    136
                     | : :|.: | .            |.  : |  | |..:    |.|  |  :.
IRE1_YEAST       140 GHI-IWSIEPENFQPLIEIQEPSRLETYETLIIEPF----GDG--NIYYF    182

LTK_HUMAN        137 SRAH-G-------V--FVSAIFS-LGL---------G-----ESLY----    157
                     : || |         :  .||.  | |.|           |      |.:|
IRE1_YEAST       183 N-AHQGLQKLPLSIRQLVST--SPLHLKTNIVVNDSGKIVEDEKVYTGSM    229

LTK_HUMAN        158 --IL--VGQ-QGE--DAC-PG------GSPESQLV-CLGESRAV---E-E    188
                       |:  :.. .||  .|. ||       || :|  | |..|.: :   | |
IRE1_YEAST       230 RTIMYTINMLNGEIISAFGPGSKNGYFGS-QS--VDCSPEEK-IKLQECE    275

LTK_HUMAN        189 HAAMDG-S--E-GVPGSRRWAGGGGGGGATY-V-FRV-RAGELE-PL-L    229
                     :..:.| :  | |: .|  :        .||:| | :.. :...|: || |
IRE1_YEAST       276 NMIVIGKTIFELGI-HS--Y-------DGASYNVTYSTWQQNVLDVPLAL    315

LTK_HUMAN        230 V-AAGGGGRAYLRP-RDRGRTQASPEKLENRSEAPGSGGRGGAAGGGGGW    277
                     . .....|.. :.| ||:. ..|| : |:.|. |      |           |
IRE1_YEAST       316 QNTFSKDGMC-IAPFRDKS-LLAS-D-LDFRI-A-----R---------W    346

LTK_HUMAN        278 TSRAPS-PQ--AG-----RSLQ--EG--A-----EGG-QGCS--EAW--A    305
                     .|  |: |.  .|     ..|:  |.     .|. :..|  :.:  .
IRE1_YEAST       347 VS--PTFPGIIVGLFDVFNDLRTNENILVPHPFNPGDHESISSNKVYLDQ    394

LTK_HUMAN        306 T--LGW-A-AAGGFGGGGGAC-TAG-GGGGGYRGGDASETDNLW-ADG--    346
                     |  |.| | ::..|    .... :|.     ..|    || :|. | ...
IRE1_YEAST       395 TSNLSWFALSSQNF---PSLVESAPI---SRY----AS-SDR-WRVSSIF    432

LTK_HUMAN        347 EDGVSF------IHP--SSELFLQPLAVTENHGEVEIRRH-LNCSH--CP    385
                     ||...|       :|.  ::| : ..|  .||:   | :.: |:.:|  .|
IRE1_YEAST       433 EDETLFKNAIMGVHQIYNNE-Y-DHL--YENY---E-KTNSLDTTHKYPP    474

LTK_HUMAN        386 L--------RDC-QWQAEL-QLAECLCPEGMELA----V-DNVT--CMDL    418
                     |        .|. | ..|: .|.|.:.||.:| |     : :.::   :|
IRE1_YEAST       475 LMIDSSVDTTDLHQ-NNEMNSLKEYMSPEDLE-AYRKKIHEQISRE-LD-    520

LTK_HUMAN        419 HKPPGPLVL----MV-AVVATST-LSLL-MV-CGVL----IL----VKQK    452
                     .|....|:|     :| .::.|.. | || :: |.:|     ||     |...
IRE1_YEAST       521 EKNQNSLLLKFGSLVYRIIETGVFL-LLFLIFCAILQRFKILPPLYVLLS    569

LTK_HUMAN        453 KWQG-LQEMRLPSPELELSK-LR--TSA--IRTAPNPY--YC--QV---G    489
                     | .| :.|..:|.  :| || |.  :|:  : |  .|:  ..  ||   |
IRE1_YEAST       570 K-IGFMPEKEIPI--VE-SKSLNCPSSSENV-T--KPFDMKSGKQVVFEG    612

LTK_HUMAN        490 -L--G---------PA-----QSWPL--------------PPG----VTE    504
                      :  |           .|     :|..|                ..|    :..
IRE1_YEAST       613 AVNDGSLKSEKDNDDADEDDEKSLDLTTEKKKRKRGSRGGKKGRKSRIAN    662

LTK_HUMAN        505 VSPANV--TL--L----RALGHGAFGE-VYEGLVIGLPGDSSPLQVAIK-    544
```

```
                     : | |.  :|  |     :.||:|:.|. |::|   ...|  .|  ||:|
IRE1_YEAST       663 I-P-NFEQSLKNLVVSEKILGYGSSGTVVFQG---SFQG--RP--VAVKR    703

LTK_HUMAN        545 TLPELCSPQDELDFLME-ALII-SKFRHQNIVR--CVGLSLRATPR-L-I    588
                     .|.:.|    | : .||| .|:. |. .|.|::|  |    | ..|.| | |
IRE1_YEAST       704 MLIDFC---D-I-ALMEIKLLTESD-DHPNVIRYYC---S-ETTDRFLYI    743

LTK_HUMAN        589 LLEL--MSGGDM-KSFLRH-SRPHLG-QP--SPLVMRDLLQLAQDIAQG-    630
                     .|||  ::..|: :|  :: |..:|. |.  :| :  .||:  | ||.|
IRE1_YEAST       744 ALELCNLNLQDLVES--KNVSDENLKLQKEYNP-I--SLLR--Q-IASGV    785

LTK_HUMAN        631 CHYLEENH---FIHRDIAARNCLLS-----CA----G-PS-RVAKIGDFG    666
                     .| |    |    .||||:..:|.|:|      .|      | .: |:. |.|||
IRE1_YEAST       786 AH-L---HSLKIIHRDLKPQNILVSTSSRFTADQQTGAENLRIL-ISDFG    830

LTK_HUMAN        667 MAR--DIYRASYYRRGDRALL--P--VK-WMPPEAFLE---G----IFT-    701
                     :.:  |..::|:  |.:   |   |   .. |..|| .||   .     :.|
IRE1_YEAST       831 LCKKLDSGQSSF--RTN---LNNPSGTSGWRAPE-LLEESNNLQCQVETE    874

LTK_HUMAN        702 ---SK-T----DS-WS-FGV---L---LWEIFSLG----Y-M-----PYP    725
                        |: |     || :. | .    |    : :|||:|     | :      |:
IRE1_YEAST       875 HSSSRHTVVSSDSFYDPF-TKRRLTRSI-DIFSMGCVFYYILSKGKHPF-    921

LTK_HUMAN        726 G-RTNQEVLDFVVGG--GRMDPPRGCPGPVY-R--I--MT----QCWQHE    763
                     | :.::| .: ::.|   . :|..: |    :: |  |  .|     |...|:
IRE1_YEAST       922 GDKYSRE-SN-IIRGIFS-LDEMK-C---LHDRSLIAEATDLISQMIDHD    964

LTK_HUMAN        764 PEL-RPSFA-SILERLQYCTQDPDVLNSLLP----ME-LGPTPEEEGTSG    806
                     | | ||: | .:| |  :     |  |  ..|     :| |   .    : .|
IRE1_YEAST       965 P-LKRPT-AMKVL-R--H----P--L--FWPKSKKLEFL--L---K-VS-    994

LTK_HUMAN        807 LGNRSLECL--RPPQPQELSPEKL-KSW-GGS----PLGPWLSSGLKPL-    847
                       :| || :  |.| |..|     | | : .||     |.|.|  : :| .
IRE1_YEAST       995 --DR-LE-IENRDP-PSAL----LMK-FDAGSDFVIPSGDW--T-VK-FD   1030

LTK_HUMAN        848 KSRGLQPQNLWNPTYR    863
                     |:  .. .||  ..||
IRE1_YEAST      1031 KT--FM-DNL--ERYR   1041

--------------------------------------------
Name 1: FBXW7_HUMAN
Name 2: CDC4_YEAST

F-box/WD repeat-containing protein 7 OS=Homo sapiens GN=FBXW7 PE=1 SV=1
Cell division control protein 4 OS=Saccharomyces cerevisiae (strain ATCC 204508
/ S288c) GN=CDC4 PE=1 SV=2

Needed for following interaction(s):
yeast   genetic FBXW7_HUMAN     PTTG1_HUMAN     FBXW7   PTTG1   CDC4_YEAST
SECU_YEAST

Length sequence 1:      707
Length sequence 2:      779
Sequence #1: FBXW7_HUMAN
Sequence #2: CDC4_YEAST
Length #1: 851
Length #2: 851
Matrix: BLOSUM62
Gap open: 2.0
Gap extend: 1.0
Length: 851
Identity: 252/851 (29.61%)
Similarity: 396/851 (46.53%)
Gaps: 279/851 (32.78%)
Score: 1054.00

FBXW7_HUMAN        5 LLSVGSKRRR-T-GGSLRGNPSSSQ-VDEEQMNRVVEEE-QQQQLRQQEE     50
                     :.|.||.... | .|:.| |.|::: |:.|. .    ||: .:.| |::
CDC4_YEAST        26 IASSGSVTALVTAAGTHR-NSSTAKTVETED-G---EEDIDEYQ-RKR--     67

FBXW7_HUMAN       51 EHTARNGEVVGVEPRPGGQNDSQQGQLEENNNRFI-SVD-EDSSGNQEEQ     98
                       .|  |.  | |..| .::|.:  :::.:|::.: .|: : ::| ....
CDC4_YEAST        68 --AA--GS--G-ESTP-ERSDFK--RVKHDNHKTLHPVNLQ-NTG-AASV    105
```

```
FBXW7_HUMAN       99 EEDEEHAGEQD-EEDEEEEEMDQESDDFDQS-DDSSREDEHTHTNSVTNS    146
                     :.|..| ...| ..|  .|::       . .| ||.|...  : |.|| |
CDC4_YEAST       106 DNDGLH-NLTDISND--AEKL------L-MSVDDGSAAP--S-TLSV-N-    140

FBXW7_HUMAN      147 SSIVDLPVHQLSSPFYTKTTKMKRKLDHGSE----VRSFSLGKKPCKVSE    192
                     ..:..   |.:::|  | |.. ...:. ||:    |.|.:: ..|  :.|
CDC4_YEAST       141 MGVAS---HNVAAP--T-TVN-AATIT-GSDVSNNVNSATI-NNP--MEE    179

FBXW7_HUMAN      193 YTSTTGLVPCS--A-TP-TTFGDL-RAA----NGQG-----QQRRRITSV    228
                          |.:|.|  | :| || ..| :..    |...     :.:..|.|
CDC4_YEAST       180 -----GALPLSPTASSPGTT-TPLAKTTKTINNNNNIADLIESKDSIIS-    222

FBXW7_HUMAN      229 QPPTGLQEWL--KMFQSWSG--PE---K-LL----A-LD--ELID-SCEP    262
                       |    |:|  ::|.:.:.  |.   | ||    | :|  ||.| .
CDC4_YEAST       223 --P----EYLSDEIFSAINNNLPHAYFKNLLFRLVANMDRSELSDLG---    263

FBXW7_HUMAN      263 TQVKHMMQVIEPQFQRDFI-SLLPKELAL----YVLSFLEPKDLLQA---    304
                     |.:|       : ..:||.| | ||.|::|    | |.| |  |::.:
CDC4_YEAST       264 TLIK------D-NLKRDLITS-LPFEISLKIFNY-LQF-E--DIINSLGV    301

FBXW7_HUMAN      305 AQTCRYW-RILAEDNLLWREKCKEEGIDEPLHIKRRKVIKP-GFIHS-PW    351
                     :|.   | :|:.:...||  | |   :   | |. ...:.| || :| ..
CDC4_YEAST       302 SQN---WNKIIRKSTSLW--K-K---L---L-IS-ENFVSPKGF-NSLNL    336

FBXW7_HUMAN      352 K-S-AY--IRQHRIDTNWR-R-G--E----LK---SPK-V-----LKGHD    380
                     | | .|  :.| : |   | | .  |     ||   :|| |     |:||.
CDC4_YEAST       337 KLSQKYPKLSQ-Q-D---RLRLSFLENIFILKNWYNPKFVPQRTTLRGHM    381

FBXW7_HUMAN      381 DHVITCLQFCGNRIVSGSDDNTLKVWSAVTGK-CLRTLVGHTGGVWSSQM    429
                     ..|||||||..|.:::|:||..::|:.::..| .|: |.||.||||:  :
CDC4_YEAST       382 TSVITCLQFEDNYVITGADDKMIRVYDSINKKFLLQ-LSGHDGGVWA--L    428

FBXW7_HUMAN      430 R---DNIIISGSTDRTLKVWNAETGECIHTLY-GHTSTVRCMHLHE-K--    472
                     :   ..|::|||||||::||:.:.|.|.| :: ||.|||||:.:.| |
CDC4_YEAST       429 KYAHGGILVSGSTDRTVRVWDIKKGCCTH-VFKGHNSTVRCLDIVEYKNI    477

FBXW7_HUMAN      473 R-VVSGSRDATLRVWDI--ET-----GQ----CL--H---------VLM    498
                     : :|:||||.||.||.:  |:     |:    .|  |         ||.
CDC4_YEAST       478 KYIVTGSRDNTLHVWKLPKESSVPDHGEEHDYPLVFHTPEENPYFVGVLR    527

FBXW7_HUMAN      499 GHVAAVRCVQ-YDGRRVVSGAYD-FMVKVWD-PETETCLHTLQGHTNRVY    545
                     ||:|:||.|. : |..||||:|| .:: ||| .:.: ||:.|.|||:|:|
CDC4_YEAST       528 GHMASVRTVSGH-GNIVVSGSYDNTLI-VWDVAQMK-CLYILSGHTDRIY    574

FBXW7_HUMAN      546 S-LQFDGIH----VVSGSLDTSIRVWDVE----TG------N----C--I    574
                     | : :|  |    .:|.|:||:||:||:|    .|      |    |  |
CDC4_YEAST       575 STI-YD--HERKRCISASMDTTIRIWDLENIWNNGECSYATNSASPCAKI    621

FBXW7_HUMAN      575 ----HTLTGHQSLTSG-MELKDNILVSGNADSTVKIWDIKTGQCLQTLQG    619
                         :||.||.:|. | :.|.|..|||..||.:::.||  ...  .:
CDC4_YEAST       622 LGAMYTLQGHTALV-GLLRLSDKFLVSAAADGSIRGWD--AND--YS---    663

FBXW7_HUMAN      620 PNK---HQ---SAVTCLQF--NKNFVITSSDDGTVKLWDLKTGEFIR-NL    660
                      .|   |.   ||:|  .|  :.|.:::.|:: ...:::|::|:.:. |
CDC4_YEAST       664 -RKFSYHHTNLSAIT--TFYVSDNILVSGSEN-QFNIYNLRSGKLVHAN-    708

FBXW7_HUMAN      661 VTLESGGSGGVVWRIRASNTK---LVCAVGSRNGTEETKLL-VLDFDVDM    706
                     : |:....   :|.:   |.|   ||.|| .::|  :: .| :|||.
CDC4_YEAST       709 I-LKDADQ---IWSV---NFKGKTLVAAV-EKDG--QS-FLEILDFS---    744

FBXW7_HUMAN      707 K    707
                     |
CDC4_YEAST       745 K    745
```

Name 1: PKNX1_HUMAN
Name 2: UNC62_CAEEL

Homeobox protein PKNOX1 OS=Homo sapiens GN=PKNOX1 PE=1 SV=3
Homeobox protein unc-62 OS=Caenorhabditis elegans GN=unc-62 PE=1 SV=1

Needed for following interaction(s):
worm   genetic RASK_HUMAN   PKNX1_HUMAN   KRAS   PKNOX1 LET60_CAEEL
UNC62_CAEEL

Length sequence 1:   436
Length sequence 2:   564
Sequence #1: PKNX1_HUMAN
Sequence #2: UNC62_CAEEL
Length #1: 593
Length #2: 593
Matrix: BLOSUM62
Gap open: 2.0
Gap extend: 1.0
Length: 593
Identity: 202/593 (34.06%)
Similarity: 282/593 (47.55%)
Gaps: 218/593 (36.76%)
Score: 839.00

```
PKNX1_HUMAN        3 ATQTLSIDSYQ-DGQQMQVVTELK---TEQ--D-----P-NCS-EPDAEG     39
                     |.|. .|.:|. |...::  .| |   .||  |      | :.| :| |..
UNC62_CAEEL       17 AVQN-GISTYDLDTSSIK--RE-KRDHNEQFNDGYGPPPGSASADP-ASY     61

PKNX1_HUMAN       40 VSPP-------------PVESQTPM---DV------DKQAIYRHPLFPLL     67
                     ::.|             |. | |||   ::      ||::||.|||:|||
UNC62_CAEEL       62 IADPAAFYNLYTNMGGAPT-S-TPMMHHEMGEAMKRDKESIYAHPLYPLL    109

PKNX1_HUMAN       68 ALLFEKCE--QST-QG-S-EGTT------SASF-DVDIEN-FVR-KQEKE    103
                     .|||||||  .|| :. | :|:|      |||| | |: | ||| .||..
UNC62_CAEEL      110 VLLFEKCELATSTPRDTSRDGSTSSDVCSSASFKD-DL-NEFVRHTQENA    157

PKNX1_HUMAN      104 GKPFFCEDPETDNLMVKAIQVLRIHLLELEKVNELCKD-FCSRYIACLKT    152
                     .|.::..:|:.|.:|:::||:||.||||||||:||| | ||:||:.|||.
UNC62_CAEEL      158 DKQYYVPNPQLDQIMLQSIQMLRFHLLELEKVHELC-DNFCNRYVVCLKG    206

PKNX1_HUMAN      153 KM-----------NSE------TL--L--SGEP----GS-------PYSP    170
                     ||           :|:      ::  |  |..|    |:       ||.|
UNC62_CAEEL      207 KMPLDIVGDERASSSQPPMSPGSMGHLGHSSSPSMAGGATPMHYPPPYEP    256

PKNX1_HUMAN      171 VQSQQI-QSA-ITGTISP-QG------IV-VPASAL-------QQGNVAM    203
                      ||..: ::| :.|. .| :|      :. :.|:|.       |.|:..:
UNC62_CAEEL      257 -QSVPLPENAGVMGG-HPMEGSSMAYSMAGMAAAAASSSSSSNQAGDHPL    304

PKNX1_HUMAN      204 ATVAGGTVY------Q---PVTVVTP----QGQVVTQ--T-LS---P-GT    233
                     |.  |||::      |   |:.|.:|    .|. :.|  | ||   | |
UNC62_CAEEL      305 AN--GGTLHSTAGASQTLLPIAVSSPSTCSSGG-LRQDSTPLSGETPMG-    350

PKNX1_HUMAN      234 IRIQ-NSQLQLQLNQ--D-LSIL-HQDDG-------S---SKN-KRGVLP    267
                      ... || :. .:::  | .|:. ..|||       |   |:| ||.| |
UNC62_CAEEL      351 -HANGNS-MD-SISEAGDEFSVCGSNDDGRDSVLSDSANGSQNGKRKV-P    396

PKNX1_HUMAN      268 ----KHA-TNVMRSWLFQH-IGHPYPTEDEKKQIAAQTNLTLLQVNNWFI    311
                         |.| |. .|:||| | :.||||:|::|||:|.:|.||:||||||||
UNC62_CAEEL      397 KVFSKEAITK-FRAWLF-HNLTHPYPSEEQKKQLAKETGLTILQVNNWFI    444

PKNX1_HUMAN      312 NARRRILQPMLD-------S---S-CSETPKTKKKTAQNR----PVQRFW    346
                     ||||||:|||:|       |   : |    |.:::   ||    |...
UNC62_CAEEL      445 NARRRIVQPMIDQNNRAGRSGQMNVC----KNRRR---NRSEQSPGPS--    485

PKNX1_HUMAN      347 PDSIA-SGVA--QPPPSEL---T-M---SEGAVVTITTPVNMNVDSLQSL    386
                     |||.: || |  .|.||.|   | |   :| ..:..|.|..    ...|.
UNC62_CAEEL      486 PDSGSDSG-ANYSPDPSSLAASTAMPYPAE-FYMQRTMPYG----GFPSF    529

PKNX1_HUMAN      387 SSDGATLAVQQVMMAG-Q-SEDESVDS-TEE--DAGAL-APAH    423
                     ::. | :.....|| | | :  .:||: :::  |   | || |
UNC62_CAEEL      530 TNP-A-MPFMNPMM-GFQVA--PTVDALSQQWVD---LSAP-H    563
```

Name 1: MYO3B_HUMAN
Name 2: HSL1_YEAST

Myosin-IIIb OS=Homo sapiens GN=MYO3B PE=2 SV=4
Probable serine/threonine-protein kinase HSL1 OS=Saccharomyces cerevisiae (strai
n ATCC 204508 / S288c) GN=HSL1 PE=1 SV=1

Needed for following interaction(s):
yeast   genetic PTTG1_HUMAN   MYO3B_HUMAN   PTTG1   MYO3B   SECU_YEAST

```
HSL1_YEAST

Length sequence 1:      1341
Length sequence 2:      1518
Sequence #1: MYO3B_HUMAN
Sequence #2: HSL1_YEAST
Length #1: 1744
Length #2: 1744
Matrix: BLOSUM62
Gap open: 2.0
Gap extend: 1.0
Length: 1744
Identity: 457/1744 (26.20%)
Similarity: 713/1744 (40.88%)
Gaps: 673/1744 (38.59%)
Score: 1474.00

MYO3B_HUMAN        1 M-KHLYGLFHYNPMML-G-LE----S-------L--PDPT----------     24
                     | |    : :.||... | ||     |         |  ||.|
HSL1_YEAST        28 MAK----V-NSNPKRASGHLERVVQSVNDATKRLSQPDSTVSVATKSSKR     72

MYO3B_HUMAN       25 ---DT---WEIIETIGKGTYGKVYKVT-NKRDGSLAAVKILDP-----V-     61
                        ||   |::::|:|||:.|:| ::. |...|.|||:||: |      |
HSL1_YEAST        73 KSRDTVGPWKLGKTLGKGSSGRV-RLAKNMETGQLAAIKIV-PKKKAFVH    120

MYO3B_HUMAN       62 -SDMDEEIEAEY-------NILQFLP--------NH--PNVVKFYG----     89
                      |: :..:...|       |:..  |         ||  .|  . ||
HSL1_YEAST       121 CSN-NGTVPNSYSSSMVTSNVSS--PSIASREHSNHSQTN--P-YGIERE    164

MYO3B_HUMAN       90 -MFYK-ADHC-VGG--QLW-------LVLELCNGGSVTE-LV-KGLLRCG    125
                      :..| ..|. |..  ::|        ||||..:||.:.: || ||.|  .
HSL1_YEAST       165 IVIMKLISHTNVMALFEVWENKSELYLVLEYVDGGELFDYLVSKGKL--P    212

MYO3B_HUMAN      126 QRLDEAMISYILYGALL-GLQHLHN-NRIIHRDVKGNNILLTTEG-GVKL    172
                     :|  || |.|  :..:: |:.:.|: | |.|||:|..|:||..:. .:|:
HSL1_YEAST       213 ER--EA-IHY--FKQIVEGVSYCHSFN-ICHRDLKPENLLLDKKNRRIKI    256

MYO3B_HUMAN      173 VDFGVSA-QLTSTRLRRNTSVGTPFWMAPEVIACEQQYDSSYDARCDVWS    221
                     .|||::| :|.: :|.: ||.|:|.:.:||:: ..:.|    :....||||
HSL1_YEAST       257 ADFGMAALELPN-KLLK-TSCGSPHYASPEIV-MGRPY---HGGPSDVWS    300

MYO3B_HUMAN      222 LGIT--AIELGDGDPPLF-DMHPV-KTLFKIP--RNPPPTLLHPEKWCEE    265
                     .||.  |: | .|..| | |.: : |.|.|:.  :...|:.|..|  ..:
HSL1_YEAST       301 CGIVLFAL-L-TGHLP-FNDDN-IKKLLLKVQSGKYQMPSNLSSE--ARD    344

MYO3B_HUMAN      266 FNHFISQCLIKDFERRPSVT--HLLDHPFIK-----GVHGKVLFLQKQLA    308
                         .||:.|:.|.|:|  :|  .:|.||.||      .|: ||  |:| :.
HSL1_YEAST       345 ---LISKILVIDPEKR--ITTQEILKHPLIKKYDDLPVN-KV--LRK-MR    385

MYO3B_HUMAN      309 KVLQDQKHQNPVA--KTRHERMHTRRPYHVEDAEKYCLEDDLVNL----E    352
                     |   |    | :|  |:..: :|...  :|..:          :|.|    |
HSL1_YEAST       386 K---D----N-MARGKSNSD-LHLLN--NVSPS--------IVTLHSKGE    416

MYO3B_HUMAN      353 VLDEDTIIHQLQKRYADLLIYTYVGDI---LI-A--L-NPF--QNLSIYS    393
                     : || :|:..||     :| : : | :   || |  | .|.  :.|  .||
HSL1_YEAST       417 I-DE-SILRSLQ-----IL-W-H-G-VSRELITAKLLQKPMSEEKL-FYS    454

MYO3B_HUMAN      394 --PQF-SRLYHGV--------KR-A---S-NPPHI-FASADAAYQCMVT-    425
                       .|: .|  |.:          |: |   |  |.|.| :|| ..|..   |
HSL1_YEAST       455 LLLQYKQR--HSISLSSSSENKKSATESSVNEPRIEYAS-KTANN---TG    498

MYO3B_HUMAN      426 L-SK--DQCI-VI-SGESGSGKTESAHLIV-QH--LTFLGKAN---N-QT    463
                     | |:  |  : .: |.|..|..| |.   | |:  :|  | .|    | ..
HSL1_YEAST       499 LRSENND--VKTLHSLEIHSEDT-ST---VNQNNAIT--G-VNTEINAPV    539

MYO3B_HUMAN      464 LREKILQ--VNSL-------VEAFGNSCT---AI---NDNSSR-F-GKYL    496
                     |.:| .|  :|:|         .||  .:.|   ||    |.:||| | ..|
HSL1_YEAST       540 LAQK-SQFSINTLSQPESDKAEA--EAVTLPPAIPIFNASSSRIFRNSY-    585

MYO3B_HUMAN      497 EMMFTPTGVVMGARIS-EYL-LEKSRV-IKQA--AREKNFHIFYYIYAGL    541
                           |.:  .:| | ..| |..||: : .|  :|| .        :
HSL1_YEAST       586 ------TSI--SSR-SRRSLRLSNSRLSL-SASTSRE-T------V----    614

MYO3B_HUMAN      542 H-HQKKLSDFRLPEEK-PPRY-IADETGRVMH--DIT-S--KE-SYRRQF    582
```

```
                     | ::..|.  :||  | |.|| :: .  |.:|  ..| |  |. | |:..
HSL1_YEAST       615 HDNEMPLP--QLP--KSPSRYSLS-R--RAIHASPSTKSIHKSLS-RKNI    656

MYO3B_HUMAN      583 EA-IQHCFRII-GFTDKEVHSVYRILAGI----LNIGN--IEF-AAI-S-    621
                     .| :. ..|.: ....|  .|:|. |..|     ||: |  :.| ..: |
HSL1_YEAST       657 AATVA-ARRTLQNSASK--RSLYS-LQSISKRSLNL-NDLLVFDDPLPSK    701

MYO3B_HUMAN      622 ---SQHQTDKSEVPNA-EA------L--Q----NAAS-VLCISPEELQEA    654
                        |:: .:||| |:: |:      |  |     ||.. :|    || :|
HSL1_YEAST       702 KPASEN-VNKSE-PHSLESDSDFEILCDQILFGNALDRIL----EE-EE-    743

MYO3B_HUMAN      655 LTSHCVVTRGET---IIRANTVD-R-AAD---VRDAMS--KALYG-RLF-    692
                      .:.  ..| :|      |.|   | : :||   : ..:|  |...| . :
HSL1_YEAST       744 -DNE--KER-DTQRQ--RQN--DTKSSADTFTI-SGVSTNKENEGPE-YP    783

MYO3B_HUMAN      693 SWI-VNRINTL-LQPDENICSAGGGMNVGILDIFGFEN-FQRNSF--EQ-    736
                     :.| .|:.| : .:|.||: |   |:: . ..||..|| .. :|:  ||
HSL1_YEAST       784 TKIEKNQFN-MSYKPSENM-S---GLS-S-FPIFEKENTLS-SSYLEEQK    825

MYO3B_HUMAN      737 -----LCINIANEQIQYYFN----QHVFALE---QME-YQNEGID--A-V    770
                          |. :|.|.      ||     |....:|    |.| .|.:. |  : :
HSL1_YEAST       826 PKRAALS-DITNS-----FNKMNKQEGMRIEKKIQREQLQKKN-DRPSPL    868

MYO3B_HUMAN      771 -PVEYED---NR-PLLDMFLQ-KPLGLLALLD--EE-SRFP-QATDQTLV    810
                      |::::    |. | .|    | || . |: ||   .. |: | .:..::|:
HSL1_YEAST       869 KPIQHQELRVNSLP-ND---QGKP-S-LS-LDPRRNISQ-PVNSKVESLL    910

MYO3B_HUMAN      811 D--KF--EDNLRCKYFW-RPKGVELCFGI-QHYAG-K-VLYDASGV-LE-    850
                     .  ||  |.    ..: | ..:| .| | : :|... | |  .||.| :|
HSL1_YEAST       911 QGLKFKKEP---ASH-WTHERG-SL-F-MSEHVEDEKPV--KASDVSIES    951

MYO3B_HUMAN      851 ----------KNRDTLPADVVVVLRTSENKLLQQ-LFSIP---L-TK-T-    883
                               .:||  |:    ||  :|:..:|: :.|:|    | |. |
HSL1_YEAST       952 SYVPLTTVATSSRD--PS----VL--AESSTIQKPMLSLPSSFLNTSMTF    993

MYO3B_HUMAN      884 GNLAQTRA-----R-ITV---AS-S-SLPPHFSAGK--AKVDTLEVIRHP    920
                     .||:|..|      : ::|   .| | :: .| ...|  ||: :| .   |
HSL1_YEAST       994 KNLSQILADDGDDKHLSVPQNQSRSVAM-SH-PLRKQSAKI-SL-T---P   1036

MYO3B_HUMAN      921 EE--TTNM--KR-Q-TVASYFRYSLMDL--LSKMVVG-Q-P-H-F-VRCI    957
                     ..   ..|:  || | :..||.  | .||  :|.|... : | : | .:.|
HSL1_YEAST      1037 RSNLNANLSVKRNQGSPGSYL--S-NDLDGISDMTFAMEIPTNTFTAQAI   1083

MYO3B_HUMAN      958 K--PND-D--R-----EALQFSRERVL---AQL---R----S-TG-ILE-    984
                     :  .|| |  :      :|..|::|:|:    |.:    :     | |. |.:
HSL1_YEAST      1084 QLMNNDTDNNKINTSPKASSFTKEKVIKSAAYISKEKEPDNSDTNYIPDY   1133

MYO3B_HUMAN      985 TVSIRRQGYSHR-I-LFEEFVKRYYYLAFTAHQTPL--ASKES-C-VAIL   1028
                     |:.    ..|..: | :||:          | .:.:..|  :|.|| . .::.
HSL1_YEAST      1134 TIP---NTYDEKAINIFED--------A-PSDEGSLNTSSSESDSRASVH   1171

MYO3B_HUMAN     1029 EK--SRLD-----HWVL-GKT--KVFLKYYHVEQLNL--LLR----EVIG   1062
                     .|   | :|      : || ..|   :|.| |::  . |.  :.|     |::.
HSL1_YEAST      1172 RKAVS-IDTMATTN-VLTPATNVRVSL-YWN--N-NSSGIPRETTEEILS   1215

MYO3B_HUMAN     1063 RVVVL--Q--AYTKGWLGARRYKRVREKREKGAIAI-QSAWRGYDARRKF   1107
                     : :.|  :  :.|.  : .:|:...|..|:..|:.| ||        .:..|
HSL1_YEAST      1216 K-LRLSPENPSNTH--M-QKRFSSTRGSRDSNALGISQS------LQSMF   1255

MYO3B_HUMAN     1108 KKISNRRNESAAH-NQAGD--TSNQS-S--GP--HS--P---VAAGTR--   1142
                     |.:  ..::. .| :|| |  .|:.| |  .|  .|  |    |    |.
HSL1_YEAST      1256 KDL--EEDQD-GHTSQA-DILESSMSYSKRRPSEESVNPKQRV---TMLF   1298

MYO3B_HUMAN     1143 GSAEVQDCSEPGDHKVLRGSVHRRSHSQAESNNGR-TQTSSN--SPAVTE   1189
                     ...| :: |:     ||..|.: :..|::.:  | : ::.||.  .| |.|
HSL1_YEAST      1299 DEEE-EE-SK----KVGGGKI-KEEHTKLD--N-KISEESSQLVLP-VVE   1337

MYO3B_HUMAN     1190 K--NGHSQAQS--S--PK-GC-----DIFAGHAN-K-HSVSGT--DLLSS   1223
                     |  |.:: .::  |  || ..       |. |..:| : |     |  .:|.|
HSL1_YEAST      1338 KKENANN-TENNYSKIPKPSTIKVTKDT-AMESNTQTH----TKKPILKS   1381

MYO3B_HUMAN     1224 --RI-CHPAP--DQQGLSLWGAP--QKPGSENGL--AQK-HRTPRRRCQQ   1263
                       .: ...||  |::  : |...  |...|.|..  |.| |.|
HSL1_YEAST      1382 VQNVEVEEAPSSDKK--N-WFVKLFQNFSSHNNATKASKNHVT-------   1421
```

```
MYO3B_HUMAN     1264 PKMLSSPEDT-MY-YNQLN-GTLEYQGSKRKPRKLGQIKVL--D-----G   1303
                      . :|. :|. |. .|:.| .:::|| .|....|.|: ||:  |     |
HSL1_YEAST      1422 -N-ISF-DDAHMLTLNEFNKNSIDYQ-LKNLDHKFGR-KVVEYDCKFVKG   1466

MYO3B_HUMAN     1304 EDEY-YK-SL-S-P----VDCIPEENNSAHPSFFSSSSKG-DSF   1338
                       .: :| .: | |    |  |..:..|.| | .:||:|. :.|
HSL1_YEAST      1467 --NFKFKIKITSTPNASSV--ITVKKRSKH-S-NTSSNKAFEKF   1504

--------------------------------------------
```
